# The targeted cytosolic degradation of class I histone deacetylases is essential for efficient alphaherpesvirus replication

**DOI:** 10.7554/eLife.110309

**Published:** 2026-07-09

**Authors:** Sheng-Li Ming, Meng-Hua Du, Jia-Ming Yang, Ya-Di Guo, Jia-Jia Pan, Wei-Fei Lu, Jiang Wang, Lei Zeng, Bei-Bei Chu

**Affiliations:** 1 College of Veterinary Medicine, Henan Agricultural University Zhengzhou China; 2 Key Laboratory of Animal Biochemistry and Nutrition, Ministry of Agriculture and Rural Affairs Zhengzhou China; 3 Key Laboratory of Animal Growth and Development of Henan Province, Henan Agricultural University Zhengzhou China; 4 State Key Laboratory of Membrane Biology, School of Pharmaceutical Sciences, Tsinghua University Beijing China; 5 Ministry of Education Key Laboratory for Animal Pathogens and Biosafety Zhengzhou China; 6 International Joint Research Center of National Animal Immunology, Henan Agricultural University Zhengzhou, Henan Province China; The University of Texas Southwestern Medical Center United States; The University of Texas Southwestern Medical Center United States

**Keywords:** alphaherpesviruses, HDACs, MDM2, ubiquitination, DNA damage response, Viruses

## Abstract

Viral infection triggers a robust DNA damage response (DDR), reshaping the host chromatin landscape to facilitate viral replication. Here, we uncover a novel mechanism by which alphaherpesviruses exploit the DDR pathway. We demonstrated that herpes simplex virus 1 (HSV-1) and pseudorabies virus (PRV) induced selective degradation of class I histone deacetylases (HDAC1/2), leading to histone hyperacetylation and subsequent DDR activation. Strikingly, viral infection promoted nuclear export of HDAC1/2, followed by MDM2-mediated K63-linked polyubiquitination and proteasomal degradation in the cytoplasm. Pharmacological inhibition of either DDR signaling or HDAC1/2 nuclear export significantly affected viral replication in vitro and in vivo. Our findings reveal a unique viral strategy to hijack host epigenetic regulation for efficient replication, and identify potential therapeutic targets for alphaherpesvirus infections.

## Introduction

HSV-1 is a widespread neurotropic virus that infects more than two-thirds of the global population under the age of 50 (James et al., 2020). After initial infection of mucosal epithelia and neuronal tissues, HSV-1 establishes lifelong latency within sensory ganglia (Feng et al., 2023). Clinically, HSV-1 infection can present as oral or genital lesions and, in severe instances, may progress to herpes simplex encephalitis—a potentially fatal inflammation of the brain (DiSabato et al., 2016; Modi et al., 2017). Emerging evidence suggests a plausible association between HSV-1 and neurodegenerative conditions such as Alzheimer’s disease, linking chronic neuroinflammation and neuronal impairment to persistent viral infection (Aïd and Bosetti, 2011; Aktas et al., 2007). To sustain lifelong infection, HSV-1 employs sophisticated strategies to evade host immune defenses and maintain latent reservoirs.

Epigenetic regulation plays a critical role in governing HSV-1 latency and reactivation (Anna, 2008; Schang et al., 2021). During latency, the virus coopts host histone deacetylases (HDACs) to enforce viral genomic silencing through chromatin condensation (Du et al., 2010). Although HDAC1 and HDAC2 are central to chromatin stability and DNA damage response (DDR), their roles during alphaherpesvirus infection appear complex and context-dependent (Fang et al., 2013). While these enzymes contribute to viral repression during latency (Zhou et al., 2013), their potential involvement in promoting lytic replication—whether through degradation or functional inactivation—has remained inadequately explored.

Histone acetylation, a pivotal epigenetic modification that modulates chromatin accessibility and transcriptional activity, is dynamically regulated by histone acetyltransferases (HATs), which add acetyl groups to activate gene expression, and HDACs, which remove these groups to enforce repression (Shvedunova and Akhtar, 2022; Lomonte et al., 2004). The HDAC family is categorized into four classes: Class I (HDAC1, 2, 3, 8), Class IIa/b, Class III (sirtuins), and Class IV (HDAC11) (Herbein and Wendling, 2010). Among these, Class I HDACs—particularly HDAC1 and HDAC2—are indispensable for chromatin remodeling, genomic integrity, and immune modulation (Chang et al., 2004; Marié et al., 2018; Nusinzon and Horvath, 2003; Guise et al., 2013). These enzymes contribute to host antiviral defense by fine-tuning the expression of immune-related genes. Nevertheless, the mechanisms through which HSV-1 overcomes this transcriptional repression remain incompletely understood (Guise et al., 2013).

HDAC1 serves as a key regulator of chromatin dynamics and innate immune signaling, interfacing with critical pathways such as NF-κB, JAK-STAT, and Toll-like receptor cascades to shape antiviral responses (Ray et al., 2008; Li et al., 2024; Marques et al., 2025; Shao et al., 2025). For example, in lung epithelial cells, HDAC1 facilitates STAT1 phosphorylation and enhances interferon-stimulated gene activation, thereby restricting influenza A virus replication (Nagesh and Husain, 2016). This underscores the dual function of HDAC1 in both epigenetic control and immune regulation. However, the specific strategies used by HSV-1 to manipulate HDAC1/2—particularly through ubiquitin-mediated degradation—to circumvent host immunity and enhance viral replication have not been clearly defined.

This study reveals that HSV-1 induces the degradation of HDAC1/2 via an MDM2-dependent ubiquitination mechanism, resulting in elevated histone acetylation, chromatin relaxation, and activation of DNA damage response pathways that collectively enhance viral replication. These findings offer novel insights into the epigenetic subversion strategies employed by HSV-1 and underscore the therapeutic potential of targeting the MDM2–HDAC axis in the treatment of herpesvirus infections.

## Results

### HSV-1 infection promotes HDAC1/2 degradation and histone hyperacetylation

**Figure 1.**
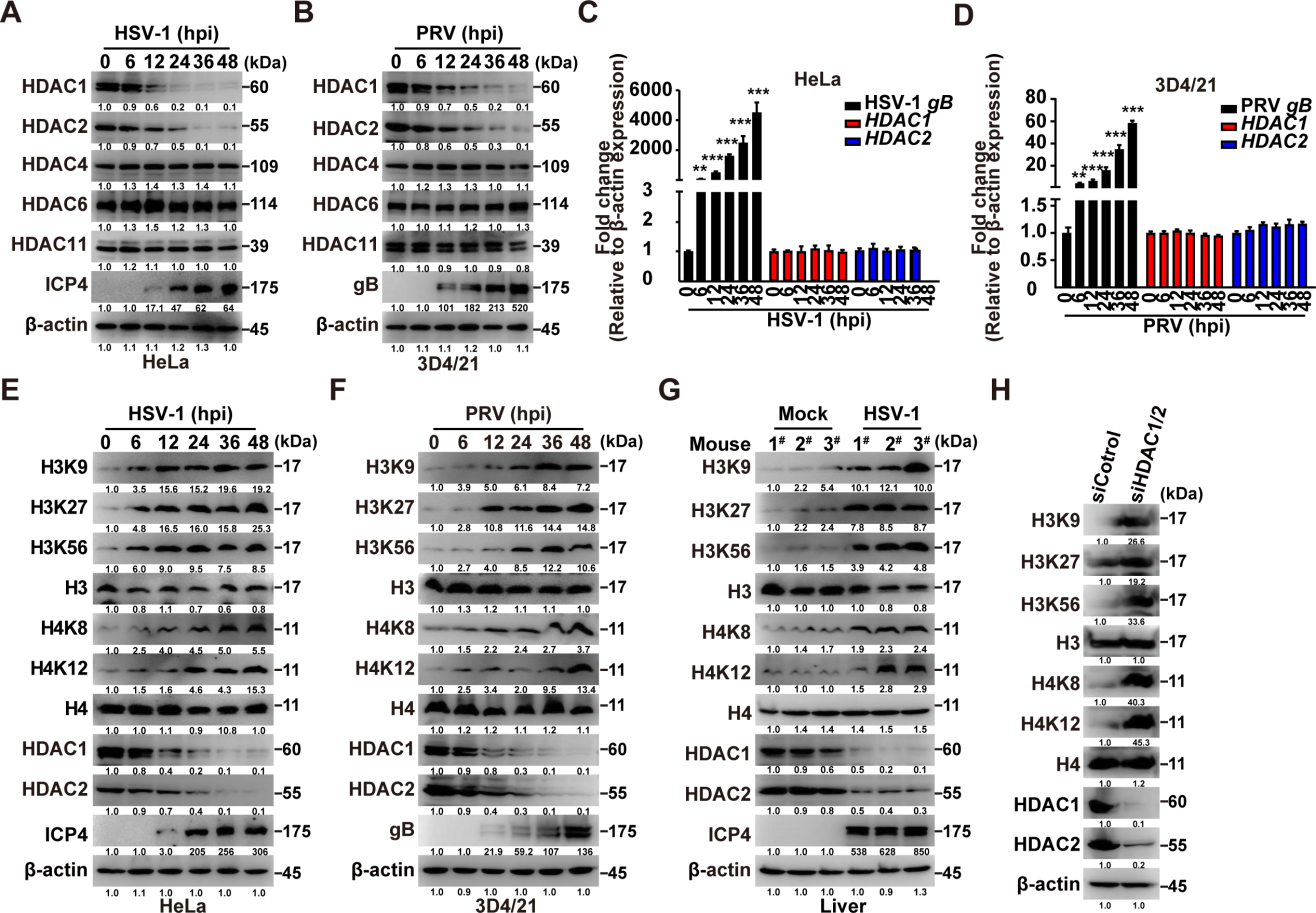
Viral infection induces degradation of class I HDACs and hyperacetylation of histones H3 and H4. (**A**) Immunoblotting analysis of the indicated HDACs in HeLa cells infected with HSV-1 (MOI = 1) for the indicated time points. (**B**) Immunoblotting analysis of the indicated HDACs in 3D4/21 cells infected with PRV-QXX (MOI = 1) for the indicated time points. (**C, D**) qRT-PCR analysis of *HDAC1* and *HDAC2* mRNA levels, normalized to β-actin expression, in HeLa cells infected with HSV-1 (**C**) or 3D4/21 cells infected with PRV-QXX (**D**) (MOI = 1). Statistical significance of the different time points in comparison with the control (0 h) is shown in the table (mean ± SD; n=3; **p<0.01, ***p<0.001). (**E**) Immunoblotting of the indicated proteins in HeLa cells infected with HSV-1 (MOI = 1) for the indicated time points. (**F**) Immunoblotting of the indicated proteins in 3D4/21 cells infected with PRV-QXX (MOI = 1) for the indicated time points. (**G**) Immunoblotting analysis of the indicated proteins in liver tissues from mice mock-infected or infected with HSV-1 (1×10⁶ pfu per mouse) at 5 days post-infection (n=3). (**H**) Immunoblotting of the indicated proteins in HeLa cells transfected with siControl or siHDAC1/2. Note: The numerical values beneath each lane in the western blot images represent the relative abundance of the corresponding protein bands, as quantified by densitometric analysis and normalized to the signal intensity of the first lane, which serves as the control group. Figure 1—source data 1.PDF file containing original western blots for Figure 1A, B, E–H, indicating the relevant bands and treatments. Figure 1—source data 2.Original files for western blot analysis displayed in Figure 1A, B, E–H.

Post-translational modifications of histones play a critical role in regulating chromatin remodeling and gene expression. HDACs, a highly conserved enzyme family (Leong and Husain, 2024), catalyze the removal of acetyl groups from histones, thereby promoting chromatin condensation and transcriptional repression. To investigate the effect of HSV-1 infection on HDAC expression and histone acetylation, we examined the protein levels of key HDAC isoforms following viral infection. Immunoblotting analysis revealed a marked decrease in HDAC1 and HDAC2 protein levels in cells infected with either HSV-1 or PRV, whereas the expression of HDAC4, HDAC6, and HDAC11 remained unchanged (Figure 1A and B). In contrast, qRT-PCR results showed no alterations in *HDAC1* and *HDAC2* mRNA levels (Figure 1C and D), suggesting that their depletion is regulated at the post-translational level.

To assess the functional impact of HDAC1/2 reduction, we examined global histone acetylation patterns. Site-specific immunoblotting demonstrated elevated acetylation at multiple lysine residues, including H3K9, H3K27, H3K56, H4K8, and H4K12, in HSV-1-infected HeLa cells (Figure 1E). A similar hyperacetylation profile was observed in PRV-infected 3D4/21 cells and in murine liver tissues infected with HSV-1 (Figure 1F and G). Next, we assessed the effect of HDAC1/2 knockdown on global histone acetylation and observed significantly elevated acetylation at multiple evolutionarily conserved lysine residues—including H3K9, H3K27, H3K56, H4K8, and H4K12—consistent with genome-wide histone hyperacetylation (Figure 1H). Collectively, these orthogonal lines of evidence—viral infection-induced degradation, cross-species conservation, and knockdown-mediated phenocopy—support the conclusion that HSV-1-mediated depletion of class I HDACs drives genome-wide histone hyperacetylation, establishing a chromatin environment permissive for viral transcription and replication.

### HSV-1 infection induces DDR through HDAC1/2 depletion and histone hyperacetylation

**Figure 2.**
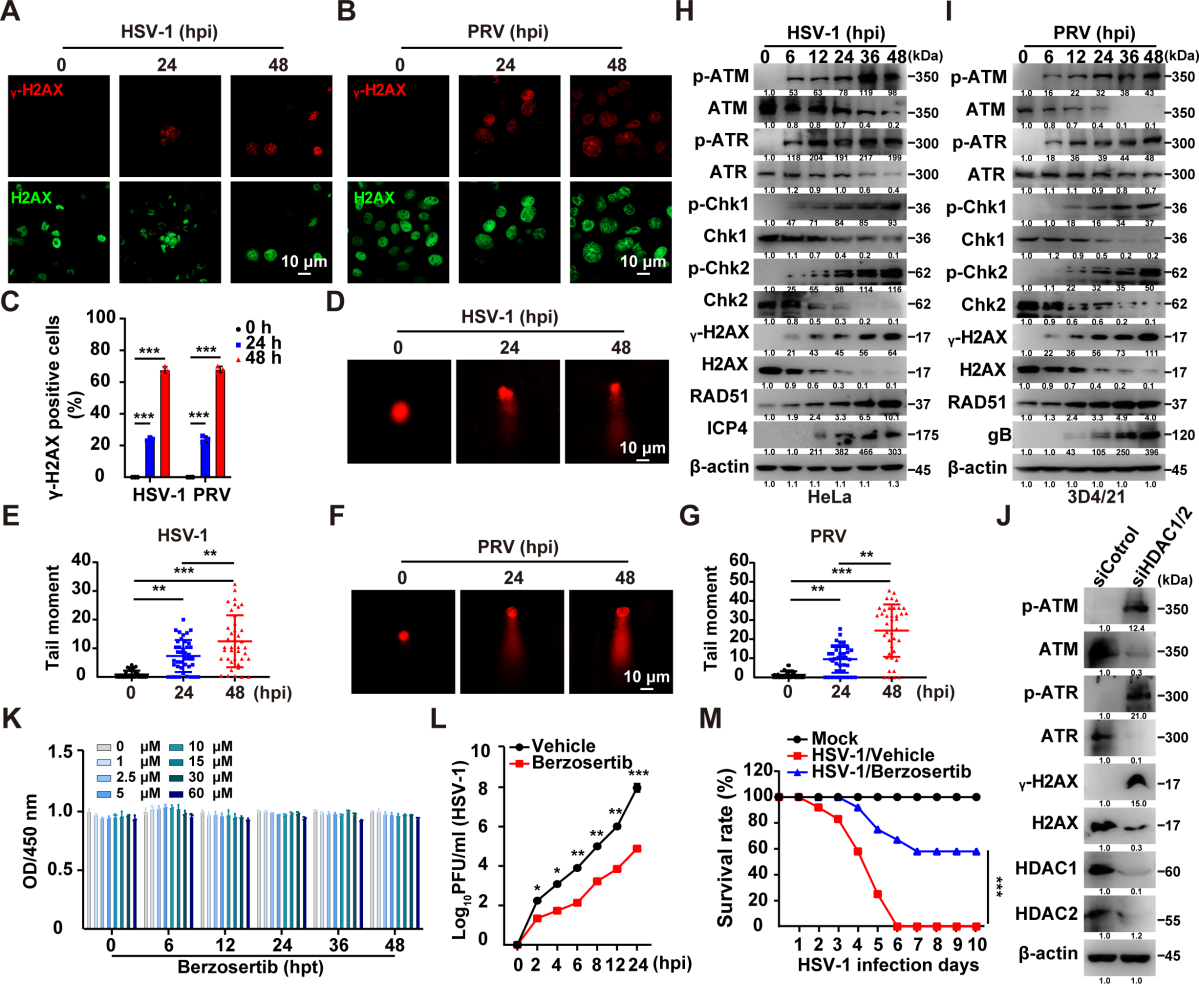
Viral infection triggers chromatin dysfunction and DDR activation via HDAC degradation. (A–C) Immunofluorescence staining of γ-H2AX (red) and H2AX (green) in HSV-1-infected HeLa cells (**A**) or PRV-infected 3D4/21 cells (**B**) (MOI = 1). Panel (C) presents the quantitative analysis of γ-H2AX foci intensity or nuclear fluorescence signal derived from panels (A) and (B). Scale bars: 10 μm. Statistical significance of the different time points in comparison with the control (0 h) is shown in the table (mean ± SD; n=100 cells/group; ***p<0.001). (**D, E**) Comet assay assessing DNA damage in HeLa cells infected with HSV-1 (MOI = 1). Scale bars: 10 μm. Statistical significance of the different time points in comparison with the control (0 h) is shown in the table (mean ± SD; n=40 cells/group; **p<0.01, ***p<0.001). (**F, G**) Comet assay assessing DNA damage in 3D4/21 cells infected with PRV (MOI = 1) at the indicated time points. Statistical significance of the different time points in comparison with the control (0 h) is shown in the table (mean ± SD; n=40 cells/group; **p<0.01, ***p<0.001). (**H, I**) Immunoblotting analysis of DDR markers (p-ATM, p-ATR, p-Chk1, p-Chk2, γ-H2AX) and viral ICP4/EP0 in HSV-1-infected HeLa cells (**G**) or PRV-QXX-infected 3D4/21 cells (**H**) (MOI = 1). (**J**) Immunoblotting analysis of DDR markers (p-ATM, p-ATR, γ-H2AX) and HDAC1/HDAC2 in HeLa cells transfected with siHDAC1/2. (**K**) HeLa cells were treated with vehicle and berzosertib (0–60 μM) for 0–48 h. Cell proliferation was analyzed by CCK-8 assay. Statistical significance of the different time points in comparison with the control (0 μM) is shown in the table (mean ± SD; ns, no significance). (**L**) Viral titers in HSV-1-infected HeLa cells (MOI = 2) treated with berzosertib (50 nM) or vehicle. t=0 h defined as time of medium replacement post-adsorption. Statistical significance of the different time points in comparison with the control (vehicle) is shown in the table (mean ± SD; n=3. *p<0.05, **p<0.01, ***p<0.001). (**M**) Survival curves of HSV-1-infected mice (1×10⁶ PFU/mouse) treated with berzosertib (20 mg/kg) or vehicle. Statistical significance of berzosertib treatment (10-day regimen) versus vehicle control in HSV-1-infected groups is summarized in the table (mean ± SD; n=12/group, ***p<0.001). Note: The numerical values beneath each lane in the western blot images represent the relative abundance of the corresponding protein bands, as quantified by densitometric analysis and normalized to the signal intensity of the first lane, which serves as the control group. Figure 2—source data 1.PDF file containing original western blots for Figure 2H–J, indicating the relevant bands and treatments. Figure 2—source data 2.Original files for western blot analysis displayed in Figure 2H–J.

Given that excessive histone acetylation can destabilize chromatin structure, we sought to elucidate the mechanism by which this epigenetic modification activates the DDR pathway. Accumulating evidence underscores the pivotal role of histone modifications in maintaining genomic stability, with dynamic changes in chromatin architecture being intimately associated with DDR initiation. To determine whether HSV-1 infection induces DDR activation, we evaluated the phosphorylation of γ-H2AX, a well-established marker of DNA double-strand breaks. Immunofluorescence microscopy revealed a significant increase in γ-H2AX foci in cells infected with HSV-1 or PRV (Figure 2A–C). Comet assays (single-cell gel electrophoresis) quantify DNA fragmentation, with an increased tail moment reflecting elevated levels of DNA damage—findings that further corroborate enhanced DNA fragmentation under these experimental conditions (Figure 2D–G). Given the central role of ATM/ATR signaling in the DDR, we proceeded to examine the activation of key checkpoint kinases. Immunoblotting analysis confirmed phosphorylation of ATM, ATR, Chk1, Chk2, H2AX, and RAD51 in HSV-1-infected HeLa cells and 3D4/21 cells (Figure 2H and I). Subsequently, we assessed the effect of HDAC1 knockdown on the DDR pathway and found that HDAC1-depleted HeLa cells exhibited reduced phosphorylation of ATM, ATR, and H2AX (Figure 2J), indicating that HDAC1 depletion is sufficient to potentiate DDR signaling. Together, these data demonstrate that HSV-1 infection induces a coordinated, ATM/ATR-dependent DDR, and that HDAC1 loss represents a functionally relevant upstream event contributing to this response.

Next, we pharmacologically validated the functional relevance of ATR in HSV-1 replication using berzosertib, a selective and clinically advanced ATR inhibitor. CCK-8 assays confirmed that the concentration of berzosertib used (100 nM) did not significantly affect HeLa cell viability or proliferation over 48 h (Figure 2K), yet it markedly reduced viral titers in plaque assays (Figure 2L). Collectively, these findings demonstrate that HSV-1 infection activates the DDR, likely through degradation of HDAC1 and HDAC2 and the resulting histone hyperacetylation. This virus-triggered DDR activation highlights the profound disruption of host chromatin homeostasis and genomic integrity by HSV-1.

### HSV-1 promotes the ubiquitin-proteasome degradation of HDAC1/2

**Figure 3.**
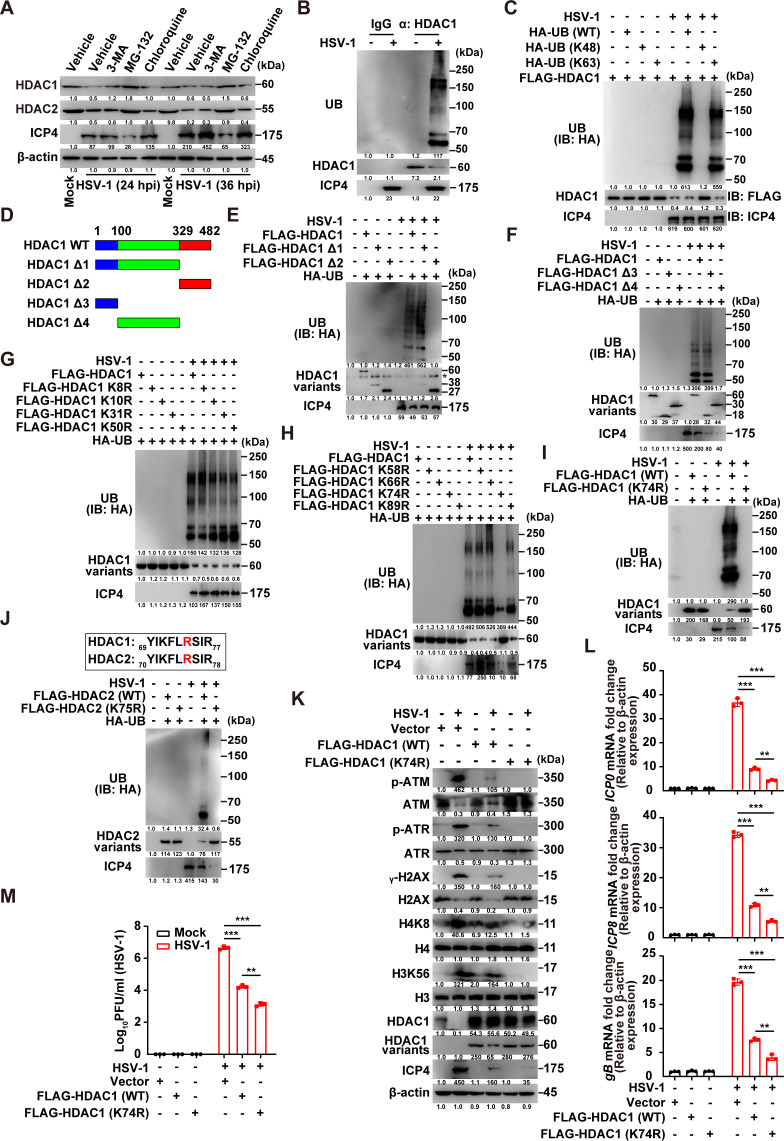
Viral infection induces K63-linked ubiquitination of HDAC1/2. (**A**) Immunoblotting analysis of the indicated proteins in HeLa cells either mock-infected or infected with HSV-1 (MOI = 1), treated with vehicle, 3-MA (10 μM), MG-132 (10 μM), or chloroquine (10 μM) for the indicated times. (**B**) Ubiquitination assay of HDAC1 in HeLa cells either mock-infected or infected with HSV-1 (MOI = 1) at 24 hpi. (**C**) Ubiquitination assay of FLAG-HDAC1 in HeLa cells transfected with HA-ubiquitin variants (WT, K48, K63), and either mock-infected or infected with HSV-1 (MOI = 1) at 24 hpi. (**D**) Schematic representation of HDAC1 deletion mutants. (E–I) Ubiquitination assays of FLAG-HDAC1 mutant variants in HeLa cells either mock-infected or infected with HSV-1 (MOI = 1) at 24 hpi. (**J**) Ubiquitination assays of FLAG-HDAC2 variants (WT and K75R) in HeLa cells either mock-infected or infected with HSV-1 (MOI = 1) at 24 hpi. (**K**) Immunoblotting analysis of the indicated proteins in HeLa cells transfected with empty vector, FLAG-HDAC1 (wild-type), or FLAG-HDAC1 (K74R), followed by mock-infected or infected with HSV-1 (MOI = 1) at 24 hpi. (**L**) qRT-PCR analysis of HSV-1 *ICP0*, *ICP8,* and *gB* mRNA levels, normalized to β-actin expression, in HeLa cells transfected with empty vector, FLAG-HDAC1 (wild-type), or FLAG-HDAC1 (K74R), followed by mock-infected or infected with HSV-1 (MOI = 1) at 24 hpi. Statistical significance was determined: (i) relative to the empty-vector control group, and (ii) between cells transfected with FLAG-HDAC1 versus those transfected with FLAG-HDAC1 (K74R), as summarized in the table (mean ± SD; n=3; ns >0.05, *p<0.05, **p<0.01, ***p<0.001). (**M**) Viral titer analysis in HeLa cells transfected with FLAG-HDAC1 or FLAG-HDAC1 (K74R), and either mock-infected or infected with HSV-1 (MOI = 1) at 24 hpi. Statistical significance was determined: (i) relative to the empty-vector control group and (ii) between cells transfected with FLAG-HDAC1 versus those transfected with FLAG-HDAC1 (K74R), as summarized in the table (mean ± SD; n=3; **p<0.01, ***p<0.001). Note: The numerical values beneath each lane in the western blot images represent the relative abundance of the corresponding protein bands, as quantified by densitometric analysis and normalized to the signal intensity of the first lane, which serves as the control group. Figure 3—source data 1.PDF file containing original western blots for Figure 3A–C, E–K, indicating the relevant bands and treatments. Figure 3—source data 2.Original files for western blot analysis displayed in Figure 3A–C, E–K.

To elucidate the mechanism responsible for HDAC1/2 degradation, we focused on their post-translational regulation. Since HDAC1/2 mRNA levels were unchanged after infection, we hypothesized that the reduction in HDAC1/2 protein is mediated through enhanced proteolytic degradation. To identify the relevant degradation pathway, HSV-1-infected HeLa cells were treated with either the proteasome inhibitor MG-132 or autophagy inhibitors (3-MA and chloroquine). Immunoblotting analysis indicated that only MG-132 rescued HDAC1/2 protein levels (Figure 3A), confirming that degradation occurs primarily via the proteasomal pathway.

To examine whether HSV-1 infection induced ubiquitination of HDAC1, endogenous immunoprecipitation assays were performed, which revealed increased ubiquitination of HDAC1 following infection (Figure 3B). Subsequent ubiquitination assays using wild-type (WT), K48-only, and K63-only ubiquitin mutants demonstrated that HDAC1 undergoes K63-linked—but not K48-linked—polyubiquitination (Figure 3C). Using a series of HDAC1 truncation mutants, we mapped the ubiquitination site to the N-terminal region (amino acids 1–100) (Figure 3D–F). Site-directed mutagenesis further identified lysine 74 (K74) as the critical residue required for ubiquitination (Figure 3G–I). Sequence alignment showed that HDAC2 K75 corresponds to HDAC1 K74 (Figure 3J), and a K75R mutation in HDAC2 similarly abolished ubiquitination (Figure 3J).

To further validate the role of HDAC1 K74, we first overexpressed HDAC1 WT and HDAC1 K74R and assessed phosphorylation of ATM, ATR, and H2AX; results showed that HDAC1 K74R overexpression suppressed HSV-1-induced DDR signaling (Figure 3K). Furthermore, qRT-PCR analysis revealed that overexpression of both HDAC1 WT and HDAC1 K74R inhibited HSV-1 *ICP0*, *ICP8,* and *gB* expression, but HDAC1 WT exerted a significantly stronger inhibitory effect (Figure 3L). Consistently, HDAC1 K74R overexpression reduced viral titers in plaque assays (Figure 3M). Collectively, these data demonstrate that HDAC1 K74 acetylation status modulates its capacity to restrain HSV-1 replication, likely by fine-tuning its regulatory influence on DDR signaling and viral gene expression.

### MDM2 functions as the E3 ligase mediating HDAC1/2 ubiquitination and degradation

**Figure 4.**
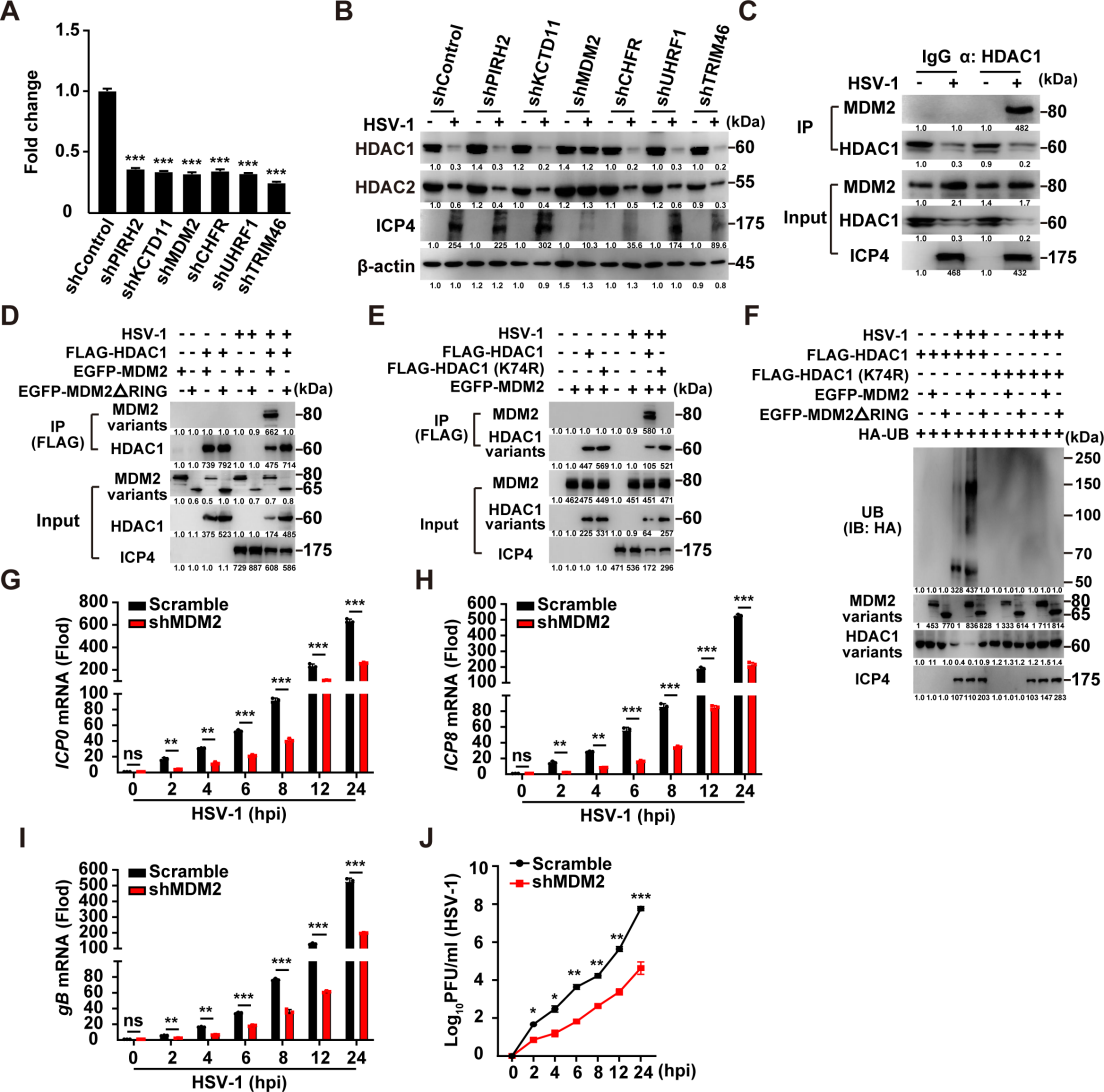
MDM2 mediates viral-induced HDAC1/2 degradation via K74/K75 ubiquitination. (**A**) qRT-PCR analysis validation of E3 ligase knockdown efficiency (shPIRH2, shKCTD11, shMDM2, shCHFR, shUHRF1, shTRIM46) in HeLa cells. Statistical significance of shRNA-mediated knockdown relative to the scramble control was assessed, and the results are summarized in the table (mean ± SD; n=3; ***p<0.001). (**B**) Immunoblotting analysis of HDAC1/2 stability in E3 ligase-knockdown HeLa cells infected with HSV-1 (MOI = 1). (**C**) Co-IP assay of endogenous HDAC1-MDM2 interaction in HSV-1-infected HeLa cells (MOI = 1; 24 hpi). (**D**) Co-IP assay of FLAG-HDAC1 with EGFP-MDM2 (WT or ΔRING) in HSV-1-infected HeLa cells (MOI = 1; 24 hpi). (**E**) Co-IP assay of FLAG-HDAC1 (WT or K74R) with EGFP-MDM2 in HSV-1-infected HeLa cells (MOI = 1; 24 hpi). (**F**) Ubiquitination assays of FLAG-HDAC1 (WT or K74R) co-expressed with EGFP-MDM2 (WT or ΔRING) in HSV-1-infected HeLa cells (MOI = 1; 24 hpi). (G–I) qRT-PCR analysis of HSV-1 *ICP0 (***H**), *ICP8 (***I**), and *gB (***J**) mRNA levels, normalized to β-actin expression, in Scramble and shMDM2 HeLa cells, and either mock-infected or infected with HSV-1 (MOI = 1) at 24 hpi. Statistical significance of shMDM2-mediated knockdown relative to the scramble control was assessed at each time point and is summarized in the table (mean ± SD; n=3; ns >0.05, *p<0.05, **p<0.01, ***p<0.001). (**J**) Viral titer analysis in Scramble and shMDM2 HeLa cells, either mock-infected or infected with HSV-1 (MOI = 2) at 24 hpi. t=0 h defined as time of medium replacement post-adsorption. Statistical significance of shMDM2-mediated knockdown relative to the scramble control was assessed at each time point and is summarized in the table (mean ± SD; n=3; **p<0.01, ***p<0.001). Note: The numerical values beneath each lane in the western blot images represent the relative abundance of the corresponding protein bands, as quantified by densitometric analysis and normalized to the signal intensity of the first lane, which serves as the control group. Figure 4—source data 1.PDF file containing original western blots for Figure 4B–F, indicating the relevant bands and treatments. Figure 4—source data 2.Original files for western blot analysis displayed in Figure 4B–F.

Having established the occurrence of K63-linked ubiquitination (Figure 3), we next sought to identify the E3 ubiquitin ligase responsible. An RNAi screen targeting candidate E3 ligases revealed MDM2 as the principal mediator of HDAC1/2 degradation. Knockdown of MDM2—but not of PIRH2, KCTD11, CHFR, UHRF1, or TRIM46—effectively prevented HDAC1/2 depletion (Figure 4A and B). Co-IP assay further confirmed an enhanced interaction between HDAC1 and MDM2 following HSV-1 infection (Figure 4C).

To characterize this interaction, we focused on the RING finger domain of MDM2, which is essential for its E3 ligase activity. Co-IP analysis demonstrated that HSV-1 infection promoted binding between FLAG-HDAC1 and EGFP-MDM2, but not with a RING-deleted mutant (EGFP-MDM2∆RING) (Figure 4D), indicating that the interaction was RING domain-dependent. To identify the region within HDAC1 required for binding, HeLa cells were co-transfected with EGFP-MDM2 and either FLAG-HDAC1 or the ubiquitination-resistant mutant FLAG-HDAC1 K74R. Infection with HSV-1 failed to induce binding between EGFP-MDM2 and the K74R mutant (Figure 4E), underscoring the essential role of lysine 74 in mediating MDM2-dependent ubiquitination.

Ubiquitination assay further revealed that overexpression of EGFP-MDM2∆RING did not enhance HSV-1-induced ubiquitination or degradation of HDAC1 (Figure 4F). Similarly, the FLAG-HDAC1 K74R mutant remained resistant to ubiquitination under all experimental conditions (Figure 4F). To further validate the role of MDM2, we performed MDM2 knockdown in infected cells; qRT-PCR analysis showed that MDM2 knockdown significantly suppressed HSV-1 *ICP0*, *ICP8*, and *gB* mRNA transcription (Figure 4G–I), and reduced viral titers in plaque assays (Figure 4J). Collectively, these results demonstrate that HSV-1 induces HDAC1/2 degradation through MDM2-mediated K63-linked ubiquitination.

### HSV-1 triggers cytoplasmic translocation of HDAC1 to facilitate its degradation

**Figure 5.**
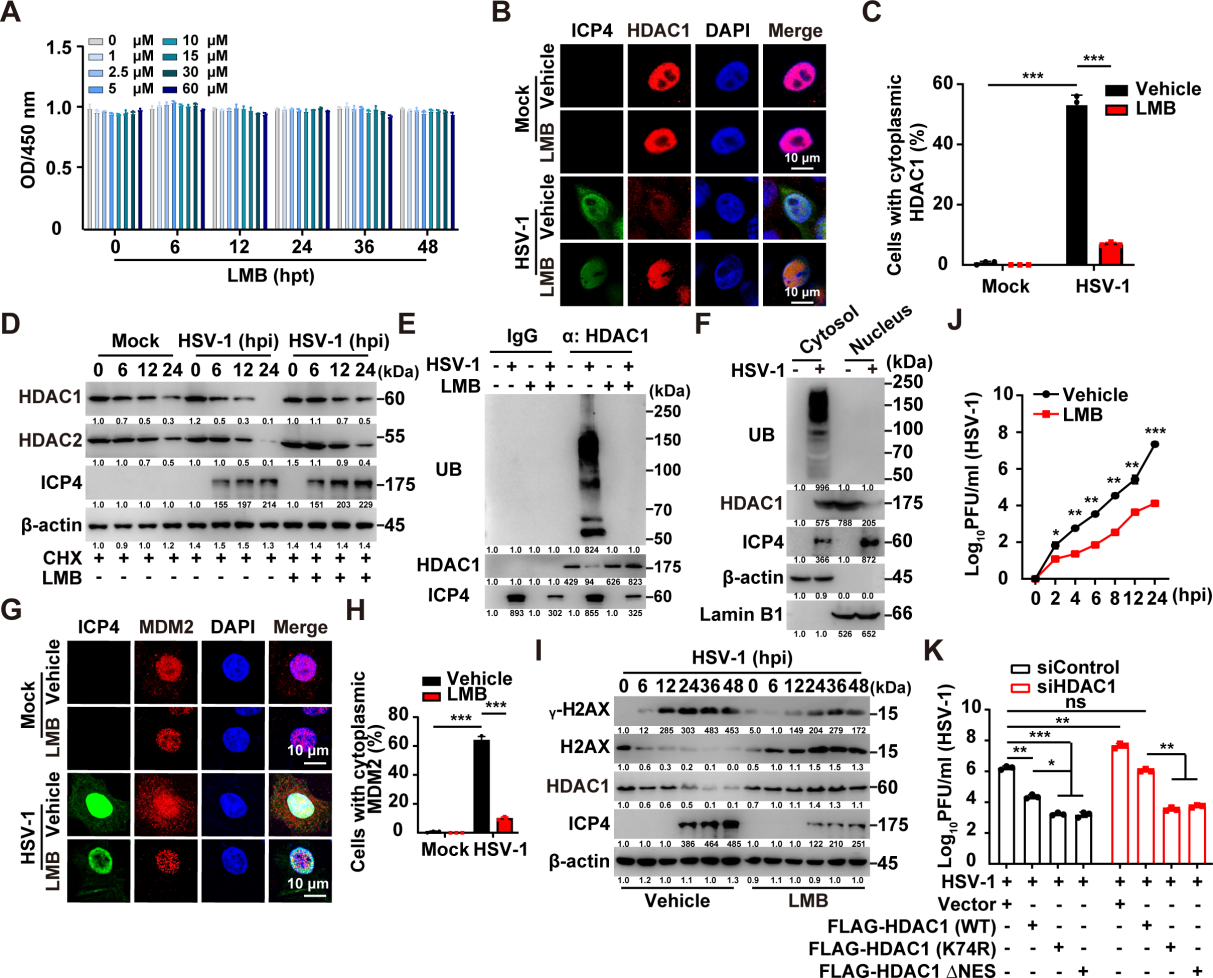
Cytoplasmic translocation enables MDM2-mediated HDAC1 degradation. (**A**) HeLa cells were treated with vehicle and LMB (0–60 μM) for 0–48 h. Cell proliferation was analyzed by CCK-8 assay. ns, no significance. (**B, C**) Immunofluorescence staining of HDAC1 (red) and viral ICP4 (green) in HSV-1-infected HeLa cells (MOI = 1)±leptomycin B (LMB; 10 nM; 24 h). Scale bar: 10 μm. (**C**) shows the quantitative analysis of (**B**), Statistical significance was determined for: (i) HSV-1 infection versus no infection in the absence of LMB; and (ii) HSV-1 infection with LMB treatment versus vehicle control, as summarized in the table (mean ± SD; n=100; ***p<0.001). (**D**) CHX chase assay measuring HDAC1/2 degradation kinetics in HSV-1-infected HeLa cells (MOI = 1)±LMB (10 μM). (**E**) Ubiquitination assay of HDAC1 in HSV-1-infected HeLa cells (MOI = 1)±LMB (10 μM; 24 h). (**F**) Immunoblotting analysis of ubiquitination in HDAC1 from cytosolic and nuclear fractions of HeLa cells infected with HSV-1. (**G, H**) Immunofluorescence analysis of MDM2 (red) and ICP4 (green) in HSV-1-infected HeLa cells (MOI = 1)±LMB (10 μM; 24 h). Scale bar: 10 μm. (**H**) shows the quantitative analysis of (**G**), Statistical significance was determined for: (i) HSV-1 infection versus no infection in the absence of LMB; and (ii) HSV-1 infection with LMB treatment versus vehicle control, as summarized in the table (mean ± SD; n=100; ***p<0.001). (**I**) Immunoblotting analysis of DDR markers (γ-H2AX) and HDAC1/2 in HSV-1-infected HeLa cells (MOI = 1)±LMB (10 μM). (**J**) Viral titers in HSV-1-infected HeLa cells (MOI = 2)±LMB (10 μM). t=0 h defined as time of medium replacement post-adsorption. Statistical significance of LMB treatment versus vehicle control in HSV-1-infected groups is summarized in the table (mean ± SD; n=3; *p<0.05, **p<0.01, ***p<0.001). (**K**) Viral titer analysis in HeLa cells transfected with siHDAC1 transfected with empty vector, FLAG-HDAC1, FLAG-HDAC1 (K74R), or FLAG-HDAC1 ∆NES, infected with HSV-1 (MOI = 1) at 24 hpi. Statistical significance was determined: (i) relative to the empty-vector control group; and (ii) among cells transfected with FLAG-HDAC1, FLAG-HDAC1 (K74R), and FLAG-HDAC1 ∆NES, as summarized in the table (mean ± SD; n=3; **p<0.01, ***p<0.001). Note: The numerical values beneath each lane in the western blot images represent the relative abundance of the corresponding protein bands, as quantified by densitometric analysis and normalized to the signal intensity of the first lane, which serves as the control group. Figure 5—source data 1.PDF file containing original western blots for Figure 5D–F, I, indicating the relevant bands and treatments. Figure 5—source data 2.Original files for western blot analysis displayed in Figure 5D–F, I.

To elucidate the mechanisms by which HSV-1 induces HDAC degradation, we systematically analyzed the subcellular localization of HDAC1. In mock-infected HeLa cells, HDAC1 was predominantly nuclear; however, HSV-1 infection triggered a pronounced translocation of HDAC1 to the cytoplasm (Figure 5B). This redistribution was completely abolished by treatment with leptomycin B (LMB), a specific inhibitor of CRM1-mediated nuclear export (Figure 5B). At the same time, CCK-8 assay showed that LMB had no significant effect on cell proliferation (Figure 5A).

To determine whether translocation is functionally linked to degradation, we conducted cycloheximide (CHX) chase assays. Degradation of both HDAC1 and HDAC2 began approximately 12 h after CHX treatment and was markedly accelerated by HSV-1 infection (Figure 5D). Importantly, LMB treatment restored degradation kinetics to levels comparable to those in mock-infected cells (Figure 5B). Moreover, under LMB treatment, HSV-1 was unable to induce ubiquitination of endogenous HDAC1 (Figure 5E). To further validate the subcellular site of HDAC1 ubiquitination, we performed nucleocytoplasmic fractionation followed by immunoblotting; results showed that HSV-1-induced ubiquitination of endogenous HDAC1 occurs specifically in the cytoplasm (Figure 5F).

We further demonstrated that nuclear export and subsequent proteasomal degradation of HDAC1 are strictly dependent on the E3 ubiquitin ligase MDM2. HSV-1 infection induces CRM1-dependent ubiquitination of endogenous HDAC1, thereby establishing CRM1-mediated nuclear export as an essential prerequisite for MDM2-catalyzed HDAC1 degradation (Figure 5G and H). Consistent with this, immunoblotting analysis revealed that LMB significantly suppressed HSV-1-induced phosphorylation of p53 and γH2AX (Figure 5I). Notably, LMB treatment-by inhibiting CRM1-dependent nuclear export and thereby preventing HDAC1 degradation—markedly impaired HSV-1 replication (Figure 5F). To rigorously test whether HDAC1 nuclear export is the critical, rate-limiting step enabling its ubiquitination and degradation, we employed NESmapper to identify a canonical nuclear export signal (NES) in HDAC1 (residues 207–216). We then generated an HDAC1 ΔNES deletion mutant and performed functional rescue experiments in HDAC1-knockdown cells by reconstituting expression of HDAC1 WT, the ubiquitination-deficient mutant HDAC1 K74R, or FLAG-tagged HDAC1 ΔNES. Only HDAC1 WT fully restored the pro-viral phenotype associated with HDAC1 depletion; in contrast, neither HDAC1 K74R nor HDAC1 ΔNES rescued viral replication, and both exerted potent antiviral activity—significantly suppressing HSV-1 proliferation relative to controls (Figure 5K). Collectively, these findings establish that HSV-1 co-opts the CRM1 nuclear export machinery to translocate HDAC1 to the cytoplasm, where it becomes accessible to MDM2 for ubiquitination and subsequent proteasomal degradation, thereby modulating host antiviral defense.

## Discussion

**Figure 6.**
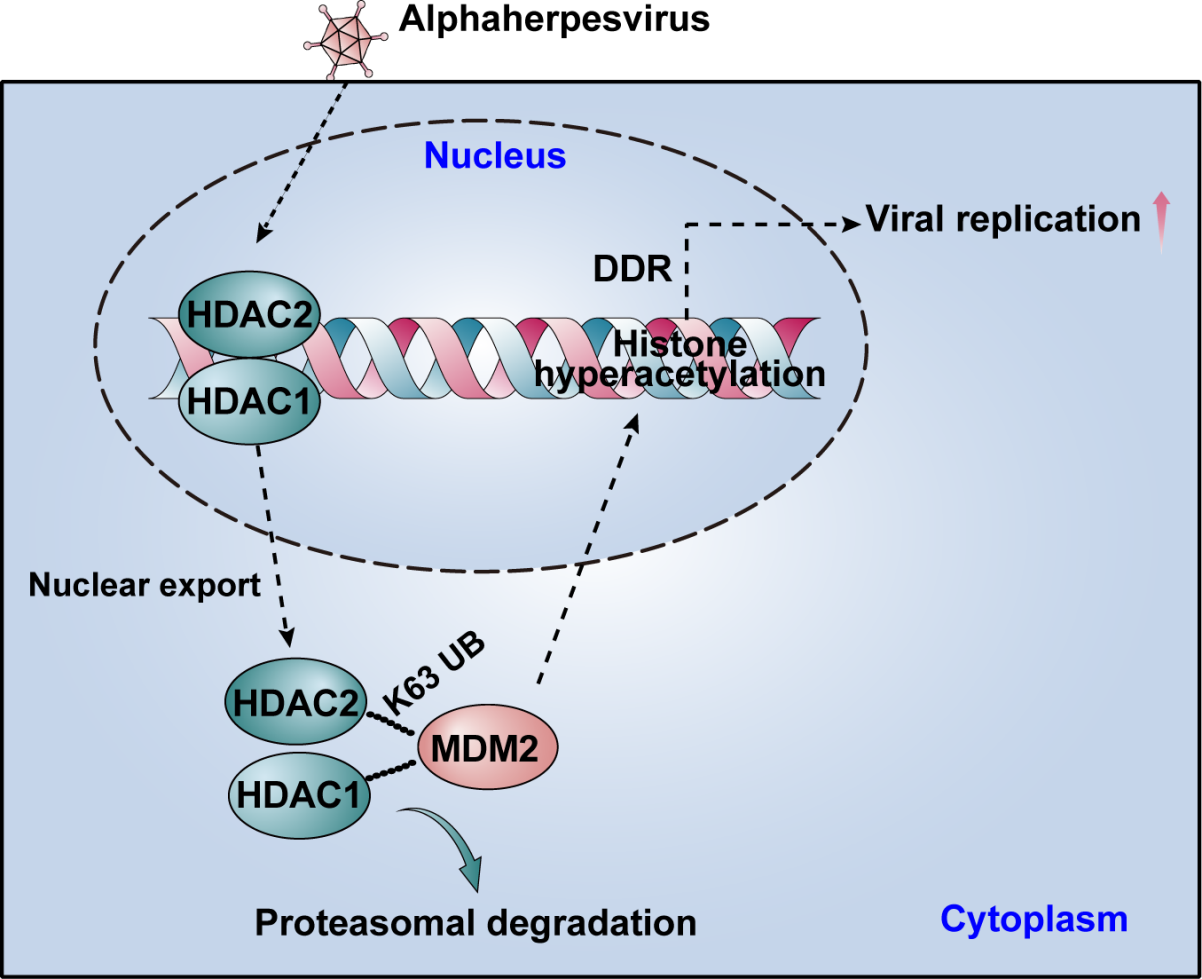
A schematic model showing the targeted cytosolic degradation of class I histone deacetylases is essential for efficient alphaherpesvirus replication.

HSV-1 manipulates host cellular pathways to facilitate its replication, latency, and reactivation (Roizman and Whitley, 2013). In this study, we demonstrate that alphaherpesvirus targets class I histone deacetylases HDAC1 and HDAC2 for MDM2-mediated K63-linked polyubiquitination and subsequent proteasomal degradation. This degradation induces histone hyperacetylation, chromatin relaxation, and enhanced viral replication—revealing a mechanism through which alphaherpesvirus reprograms the host epigenetic landscape to optimize viral replication (Figure 6).

In contrast, HSV-1 actively targets HDAC1 and HDAC2 for proteasomal degradation to facilitate lytic replication—a strategy that stands in marked contrast to human cytomegalovirus (HCMV), which relies on HDAC activity to maintain latency (Murphy et al., 2002). This divergence underscores a fundamental difference in epigenetic regulation among herpesviruses. Our data demonstrate that HSV-1-mediated HDAC1/2 degradation initiates as early as 4–6 h post-infection, coinciding with the onset of immediate-early gene expression and preceding peak viral DNA synthesis. These findings support a context-dependent, dual functional role for HDAC1/2: their enzymatic activity helps enforce transcriptional silencing during latency, whereas their timely removal is essential for chromatin remodeling and efficient lytic gene expression.

We identified MDM2 (Honda et al., 1997) as the principal E3 ubiquitin ligase responsible for ubiquitinating HDAC1 at lysine 74 and HDAC2 at lysine 75, leading to their proteasomal degradation. This mechanism is distinct from other E3 ligases, including PIRH2 (Daks et al., 2022) and TRIM46 (Tantai et al., 2022), and critically depends on the RING domain of MDM2 (Wade et al., 2010). While K63-linked ubiquitination is typically associated with non-proteolytic functions, our work establishes its novel role in mediating HDAC1/2 degradation—uncovering a previously unrecognized viral pathogenesis pathway. Targeting this axis using MDM2 inhibitors or inhibiting nuclear export of HDAC1/2 significantly restricts viral replication, suggesting that host-directed antiviral strategies may help overcome resistance to direct-acting agents (Wade et al., 2010).

Depletion of HDAC1/2 increases acetylation at histone residues associated with open chromatin and active transcription. Similar chromatin remodeling has been observed in other viral infections (Lieberman, 2016), underscoring its conserved role in viral gene regulation. Our results are consistent across multiple cell types, including HeLa, 3D4/21, and murine liver, emphasizing the central importance of HDAC1/2 suppression in HSV-1 biology. Notably, HDAC1/2 degradation activates DDR, as indicated by increased γ-H2AX foci and phosphorylation of ATM, ATR, Chk1, and Chk2. This aligns with previous reports that HSV-1 exploits DDR signaling to enhance replication (Mertens and Knipe, 2021; Anacker et al., 2014; Shah and O’Shea, 2015). Importantly, HDAC1/2 undergo CRM1-dependent nuclear export prior to degradation—a process inhibited by leptomycin B—highlighting the essential role of subcellular localization in viral manipulation of host proteins.

HSV-1-mediated HDAC1/2 degradation likely supports viral replication through multiple mechanisms, including evasion of nuclear antiviral sensors and stimulation of viral gene expression. Targeting the MDM2–HDAC1/2 axis represents a promising antiviral strategy. MDM2 inhibitors reduce HSV-1 titers (Ming et al., 2024), suggesting that modulating ubiquitination pathways could complement existing therapies. Beyond herpesviruses, the MDM2–HDAC axis may be exploited by other DNA viruses that manipulate chromatin and DDR (Lieberman, 2016), indicating a possible common host vulnerability. However, broad HDAC inhibition may unintentionally enhance viral replication, underscoring the need for targeted therapeutic approaches (Cody et al., 2014).

This study primarily employed in vitro models (HeLa cells), which lack the neuronal environment essential for studying HSV-1 latency. Future work should validate these findings in primary neurons and trigeminal ganglia (Deng et al., 2024). Additional studies are also needed to determine whether HDAC1/2 degradation occurs during reactivation or is limited to lytic infection (Anna, 2008), and to explore the interplay between HDAC degradation, viral tegument proteins, and host stress responses. In vivo evaluation of inhibitors targeting the MDM2–HDAC1/2 pathway will be crucial for assessing their therapeutic potential.

## Materials and methods

### Mice

Female C57BL/6J mice (6–8 weeks old) were purchased from the Experimental Animal Center of Zhengzhou University (Zhengzhou, China) and housed in specific-pathogen-free facilities under controlled environmental conditions, including a 12 h light–dark cycle and a temperature of 22°C. For in vivo infection studies, mice were anesthetized with isoflurane prior to intranasal inoculation with HSV-1, at a dose of 1×10⁶ PFU per mouse in a total volume of 30 µL PBS. Clinical signs—including ruffled fur, hunched posture, reduced mobility, and body weight loss 15%—were monitored daily. On day 15 post-infection, mice were humanely euthanized by intravenous injection of sodium pentobarbital (90 mg/kg), following the ‘Guidelines for the Euthanasia of Laboratory Animals’. Immediately thereafter, tissues—including liver, spleen, lung, brain, and draining cervical lymph nodes—and serum were harvested under sterile conditions. All tissues were snap-frozen in liquid nitrogen within 60 s of excision and stored at –80°C until further analysis. Samples were processed for western blotting. All procedures were in accordance with ethical guidelines.

### Reagents and plasmids

Reagents were sourced as follows: Leptomycin B (HY-16909), MG-132 (HY-13259), 3-MA (HY-19312), cycloheximide (HY-12320), and chloroquine (HY-17589A) were purchased from MedChemExpress; berzosertib (S7102) was purchased from Selleck; DMSO (W387520) was purchased from Sigma-Aldrich. Unless otherwise specified, all inhibitors were added at 1 h post-infection (hpi), after completion of viral adsorption and entry, to avoid interference with early viral processes. Antibodies against CHK1 (25887-1-AP), CHK2 (13954-1-AP), RAD51 (14961-1-AP), β-actin (66009-1-lg), P53 (10442-1-AP), HDAC1 (10197-1-AP), HDAC2 (12922-3-AP), HDAC4 (17449-1-AP), HDAC6 (12834-1-AP), HDAC11 (67949-1-Ig), and EGFP (50430-2-AP) were purchased from Proteintech; antibodies against p-P53 (9286), p-ATM (13050), ATM (2873), ATR (13934), p-ATR (2853), p-CHK1 (12302), p-CHK2 (2197), γ-H2AX (80312), H3 (4499), H4K8ac (2594), H4K12ac (13944), H4 (13919), H2AX (7631), H3K9ac (4658), H3K27ac (8173), UB (20326), and MDM2 (86934) were purchased from Cell Signaling Technology; H3K56ac antibody (07–677) was purchased from Millipore; ICP4 antibody (ab6514) was purchased from Abcam; ICP0 antibody (sc-53070) was purchased from Santa Cruz Biotechnology; FLAG antibody (F7425) was purchased from Sigma-Aldrich; HA antibody (A00169) was purchased from GenScript.

*HDAC1* and *HDAC2* coding sequences were amplified from HEK293 cell cDNA and cloned into p3×FLAG-CMV-10 to generate FLAG-tagged expression constructs. MDM2 coding sequence was amplified and inserted into pEGFP-C1 to produce EGFP-MDM2. Site-directed mutagenesis was performed using the QuikChange Site-Directed Mutagenesis Kit (Agilent Technologies, 200523) per the manufacturer’s instructions to generate: FLAG-HDAC1 K8R, FLAG-HDAC1 K10R, FLAG-HDAC1 K31R, FLAG-HDAC1 K50R, FLAG-HDAC1 K58R, FLAG-HDAC1 K66R, FLAG-HDAC1 K74R, FLAG-HDAC1 K89R, FLAG-HDAC2 K75R, FLAG-HDAC1 (aa 1–329), FLAG-HDAC1 (aa 329–482), FLAG-HDAC1 (aa 1–100), FLAG-HDAC1 (aa 100–329), and EGFP-MDM2∆RING (aa 1–435). FLAG-HDAC1 ΔNES (residues 207–216 deleted), in which the deletion encompasses the CRM1-dependent nuclear export signal as predicted by NESmapper. HA-tagged ubiquitin constructs [HA-UB (WT), HA-UB (K48), HA-UB (K63)] were provided by Dr. Bo Zhong (College of Life Sciences, Wuhan University, China).

### Cells

Cell lines (HeLa/ATCC CL-82, Vero/ATCC CL-81, 3D4/21/ATCC CRL-2843, HEK293T/ATCC CRL-11268) were cultured in DMEM 10566-016 or RPMI 1640 (Gibco, 11965092) supplemented with 10% fetal bovine serum (FBS; Gibco, 10099141C), 1% penicillin/streptomycin, and 1% L-glutamine (B540732, Sangon) at 37°C under 5% CO₂ in a humidified incubator. All cell lines were authenticated by short tandem repeat (STR) profiling performed by the American Type Culture Collection (ATCC), and routinely tested for mycoplasma contamination using PCR-based detection—results were consistently negative. For transient transfections, DNA constructs were delivered to HeLa and HEK293T cells using Lipofectamine 3000 (Invitrogen, L3000001) per the manufacturer’s protocol.

To generate shRNA-mediated knockdown cell lines, HEK293T cells were co-transfected with control or gene-specific shRNA (Supplementary file 1), packaging plasmid psPAX2, and envelope plasmid pMD2.G. Post-transfection (6 h), medium was replaced, and lentiviral particles were harvested at 48 h. Parental cells were infected with viral supernatant and selected with puromycin to establish stable knockdown lines.

To generate siRNA-mediated knockdown cell lines, HeLa cells were transfected with either a non-targeting control siRNA or gene-specific siRNAs (Supplementary file 1) using Lipofectamine RNAiMAX (Invitrogen, 13778030). Transfection was performed at a confluency of approximately 30%, and target gene and protein expression levels were assessed 48 h post-transfection.

### Viruses

HSV-1 strain F (provided by Dr. Chun-Fu Zheng, University of Calgary, Canada) and PRV-QXX were propagated per established protocols (Wang et al., 2020). Viral titers were determined by plaque assays in Vero cells. For in vitro infections, cells were infected with HSV-1 or PRV-QXX at an MOI of 1. For in vivo studies, mice were intranasally inoculated with HSV-1 (1×10⁶ pfu/mouse).

### Immunoblotting, ubiquitination, and Co-immunoprecipitation (Co-IP)

For immunoblotting, cells were lysed in RIPA buffer (50 mM Tris-HCl [pH 8.0], 150 mM NaCl, 1% Triton X-100, 1% sodium deoxycholate, 0.1% SDS, 2 mM MgCl_₂_) supplemented with protease/phosphatase inhibitors. Proteins were resolved by SDS-PAGE, transferred to PVDF membranes, and blocked with 5% nonfat milk in TBST (1 h, room temperature [RT]). Membranes were incubated with primary antibodies (overnight, 4°C), then HRP-conjugated secondary antibodies (1 h, RT). Signals were detected using Laminate Crescendo Western HRP Substrate (Millipore, WBLUR0500) on a GE AI600 imager.

For ubiquitination assay, cells were lysed in IP buffer (50 mM Tris-HCl [pH 7.4], 150 mM NaCl, 1% NP-40, 1% sodium deoxycholate, 5 mM EDTA, 5 mM EGTA) and clarified (16,000×*g*, 10 min, 4°C). Subsequently, 900 μL aliquots were incubated with 40 μL of a 1:1 slurry of Sepharose beads conjugated to anti-HDAC1 or anti-FLAG mouse monoclonal antibodies (4°C, 4 h). Beads were washed four times with IP buffer, eluted in SDS sample buffer (10 min, boiling), and analyzed by immunoblotting.

For Co-IP assay, cells were lysed in Co-IP buffer (50 mM Tris-HCl [pH 7.4], 150 mM NaCl, 1% NP-40, 5 mM EDTA, 5 mM EGTA) and clarified (16,000×*g*, 10 min, 4°C). Aliquots (900 μL) were incubated with 40 μL of a 1:1 slurry of Sepharose beads conjugated to IgG (GE Healthcare, AI600) or anti-FLAG mouse monoclonal antibody (4°C, 4 h). Beads were washed three times with Co-IP buffer, eluted in SDS sample buffer (10 min, boiling), and analyzed by immunoblotting.

### Immunofluorescence assay

Cells were fixed in 4% paraformaldehyde at RT for 20 min on coverslips in 12-well plates. Following three washes with PBS, cells were permeabilized with 0.2% Triton X-100 for 20 min and subsequently blocked with 10% FBS. Specific primary antibodies, diluted in 10% FBS, were then applied to the cells and incubated for 1 h at RT. After three PBS washes, cells were exposed to the appropriate secondary antibodies, also diluted in 10% FBS, for 1 h at RT. Nuclei were stained with DAPI for 5 min at RT, mounted using Prolong Diamond (Invitrogen, P36970), and visualized using a Zeiss LSM 800 confocal microscope.

### Comet assay

Cells were seeded in 6-well plates and treated accordingly. Frosted microscope slides were coated with 0.5% normal melting point agarose. A mixture of 10 μL DMEM containing around 10,000 cells and 75 μL of 0.7% low melting point agarose was layered onto the pre-coated agarose. An additional 75 μL of 0.7% low melting point agarose formed a third layer. The slides were lysed in a buffer containing 10 mM Tris-HCl, pH 10.0, 2.5 M NaCl, 100 mM Na2EDTA, 1% Triton X-100, and 10% DMSO for 2 h at 4°C. Post-lysis, the slides were treated with an electrophoresis solution (300 mM NaOH, 1 mM Na2EDTA, pH >13) for 40 mi, electrophoresed at 20 V (∼300 mA) for 25 min, and neutralized with 0.4 mM Tris-HCl (pH 7.5). Subsequently, cells were stained with propidium iodide (5 μg/mL) and examined using a Zeiss LSM 800 confocal microscope. DNA damage was assessed based on tail moment using CometScore software.

### qRT-PCR analysis

Total RNA was isolated using TRIzol reagent (TaKaRa, 9108) and reverse-transcribed into cDNA with the PrimeScript RT reagent kit (TaKaRa, RR047A). qRT-PCR was performed in triplicate using SYBR Premix Ex Taq (TaKaRa, RR820A) according to the manufacturer’s protocol. Expression levels were normalized to *β-actin* as an internal reference. Melting curve analysis confirmed amplification specificity by verifying single-product formation in all reactions. Relative gene expression was calculated using the 2^˗ΔΔCt^ method. Primer sequences are provided in Supplementary file 1.

### Plaque assay

Vero cells (seeded in six-well plates) were grown to confluence, infected with serially diluted viruses (10⁻¹–10⁻⁷; 1 h, 37°C), and washed with PBS to remove residual inoculum. DMEM containing 1% methylcellulose (4 mL/well) was added, and cells were incubated for 4–5 days. Post-incubation, cells were fixed with 4% paraformaldehyde (15 min), stained with 1% crystal violet (30 min), and plaques were quantified.

### Statistical analysis

Statistical analyses were performed using GraphPad Prism 8 software. Comparisons between two groups were evaluated using a two-tailed Student’s *t*-test. Significance was defined as p<0.05. Data are presented as mean ± SD of three independent experiments. Kaplan–Meier survival curves were generated and analyzed for mouse survival assessment.

### Declaration of generative AI and AI-assisted technologies in the writing process

This study affirms that neither generative artificial intelligence nor any other AI-assisted technologies were employed in the preparation of this manuscript.

## Data Availability

The data that support the findings of this study are openly available in "Mendeley Data, V3" at https://doi.org/10.17632/yg5fgtvxzk.3. The following dataset was generated: MingS-L 2025The targeted cytosolic degradation of class I histone deacetylases is essential for efficient alphaherpesvirus replicationMendeley Data10.17632/yg5fgtvxzk.3PMC1334938042423462

## Additional files

Supplementary file 1. List of shRNAs and primers used in this study.

MDAR checklist
