# Supplementary figures and images for: The targeted cytosolic degradation of class I histone deacetylases is essential for efficient alphaherpesvirus replication (part 1 of 2)
Source: eLife. 2026 Jul 9;15:RP110309. doi: 10.7554/eLife.110309 (PMC13349380; doi:10.7554/eLife.110309)

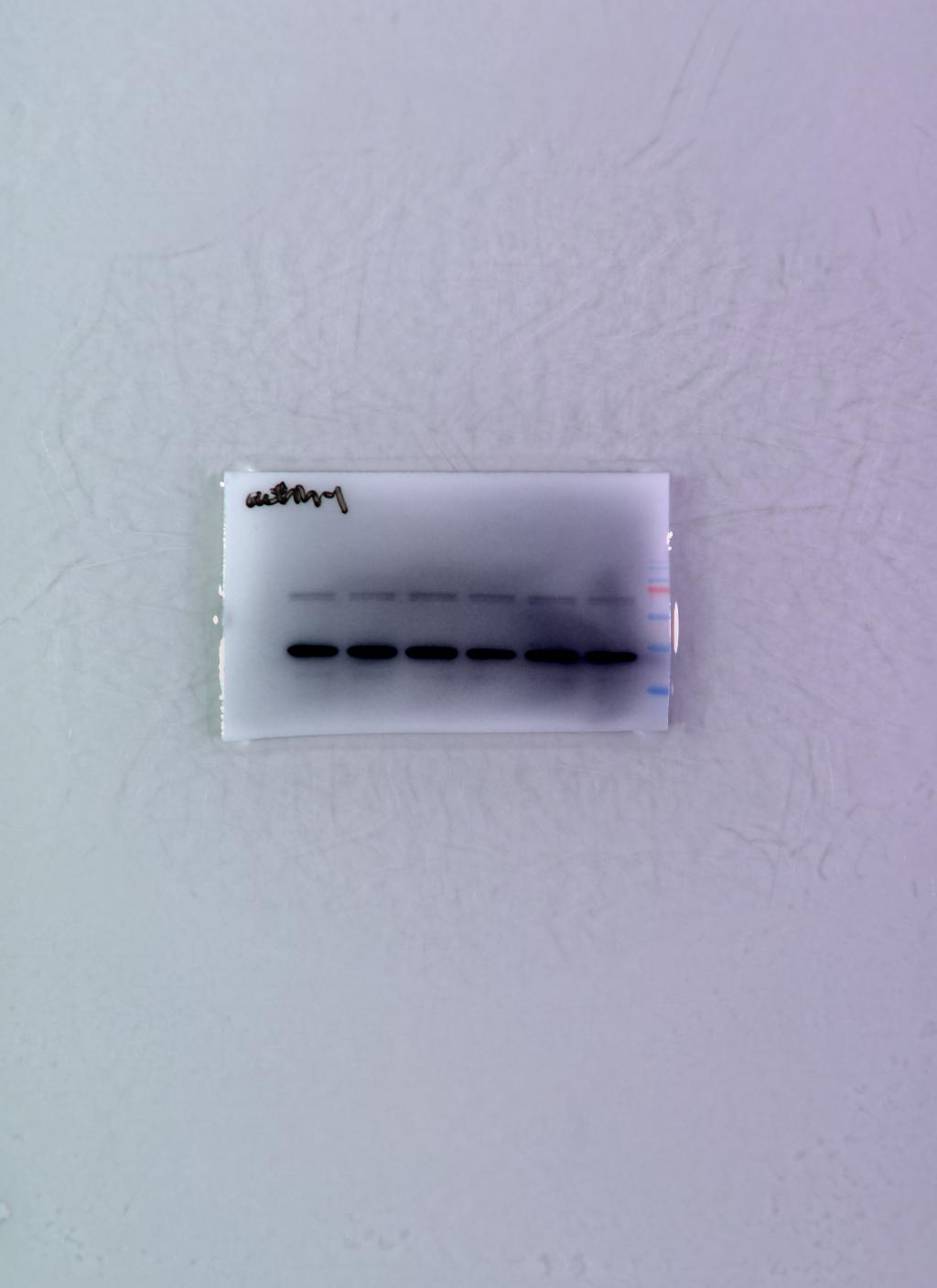

Supplement: Figure 1—source data 2. [file elife-110309-fig1-data2.zip › Figure 1-Source Data 2/ACTIN 1-1 2023.11.25_23.47.30_Ch+Marker.jpg]

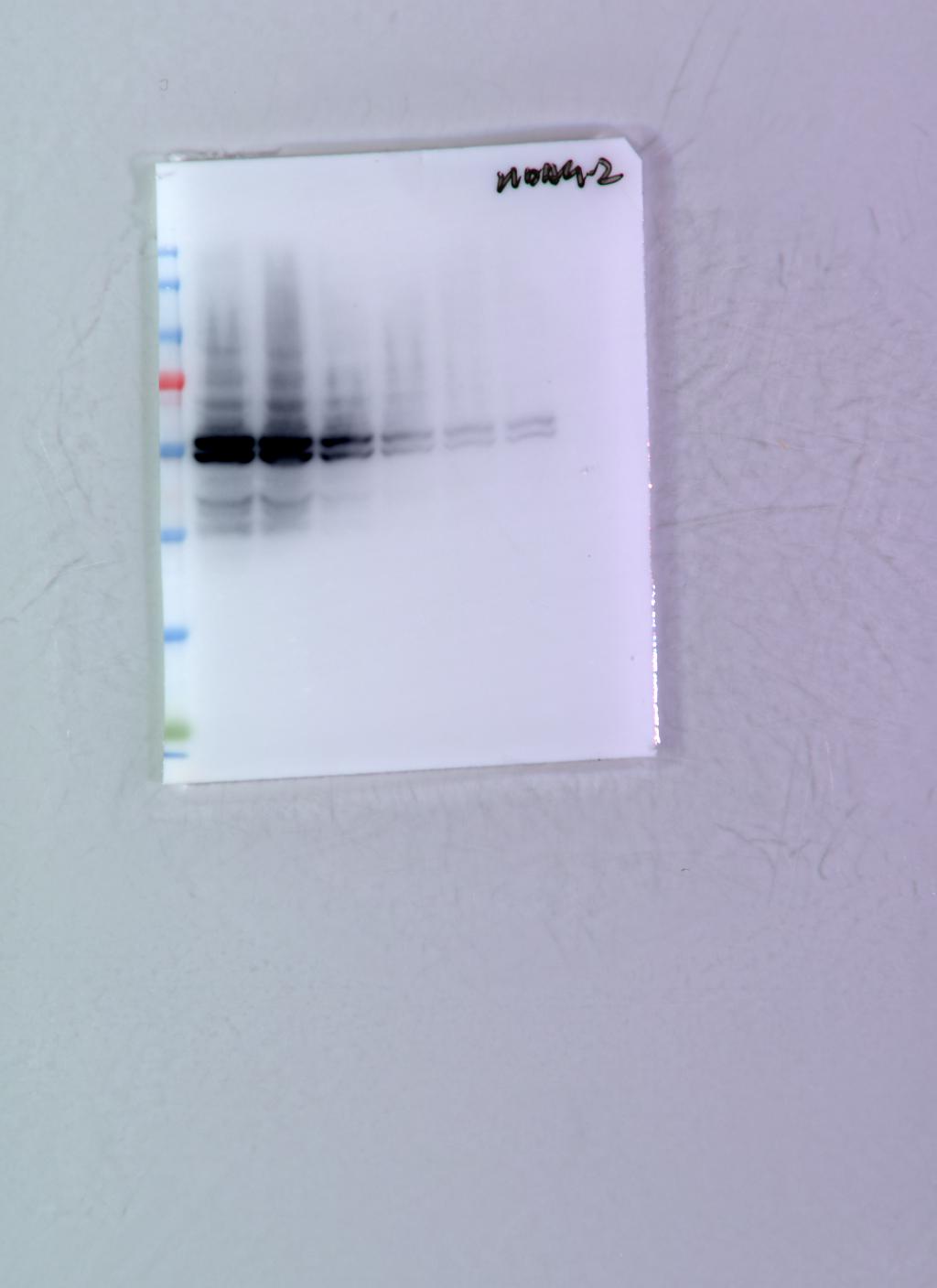

Supplement: Figure 1—source data 2. [file elife-110309-fig1-data2.zip › Figure 1-Source Data 2/HDAC1 4-0 2023.11.26_11.28.14_Ch+Marker.jpg]

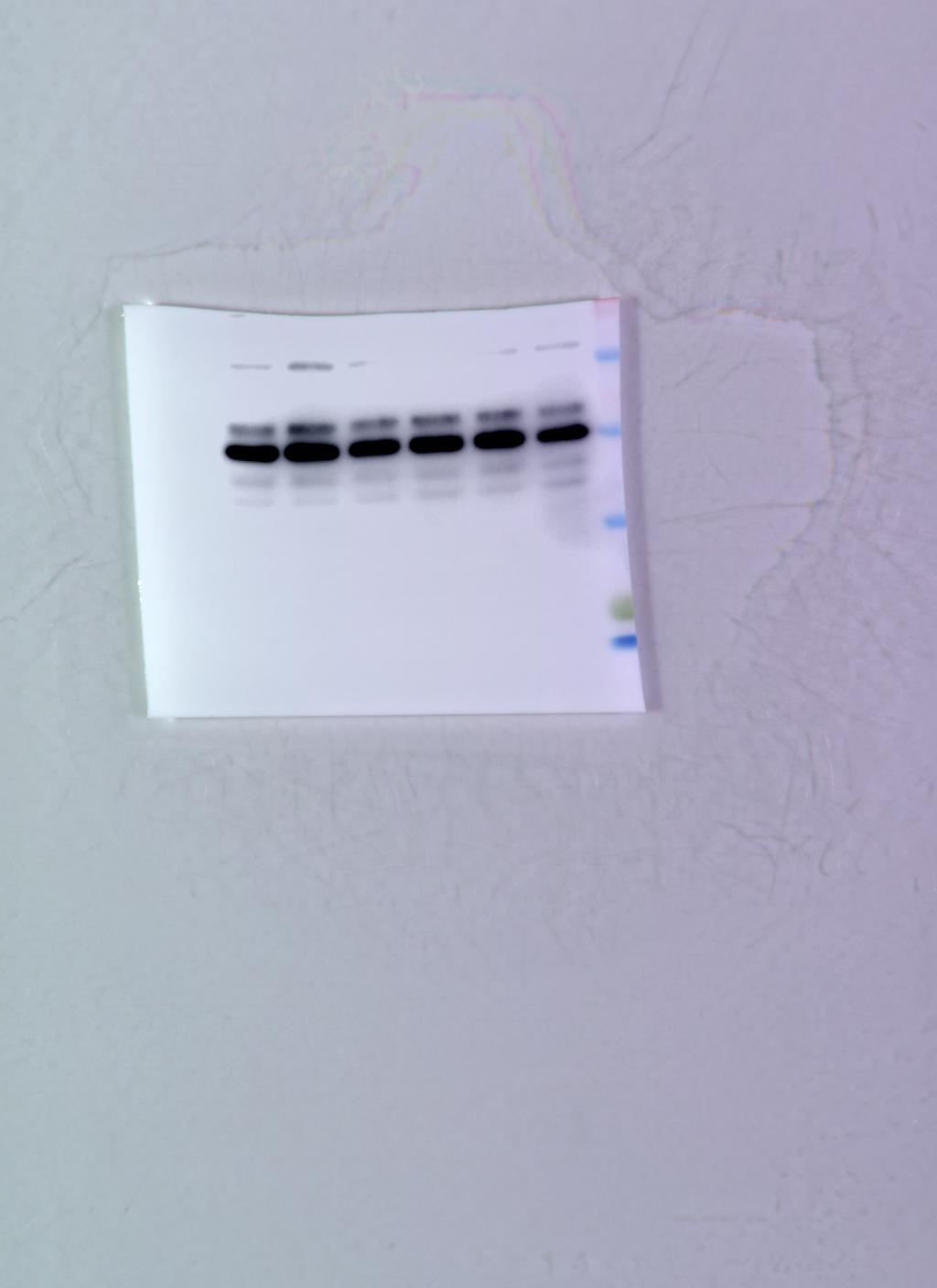

Supplement: Figure 1—source data 2. [file elife-110309-fig1-data2.zip › Figure 1-Source Data 2/HDAC11 1-2 2023.11.25_23.57.52_Ch+Marker.jpg]

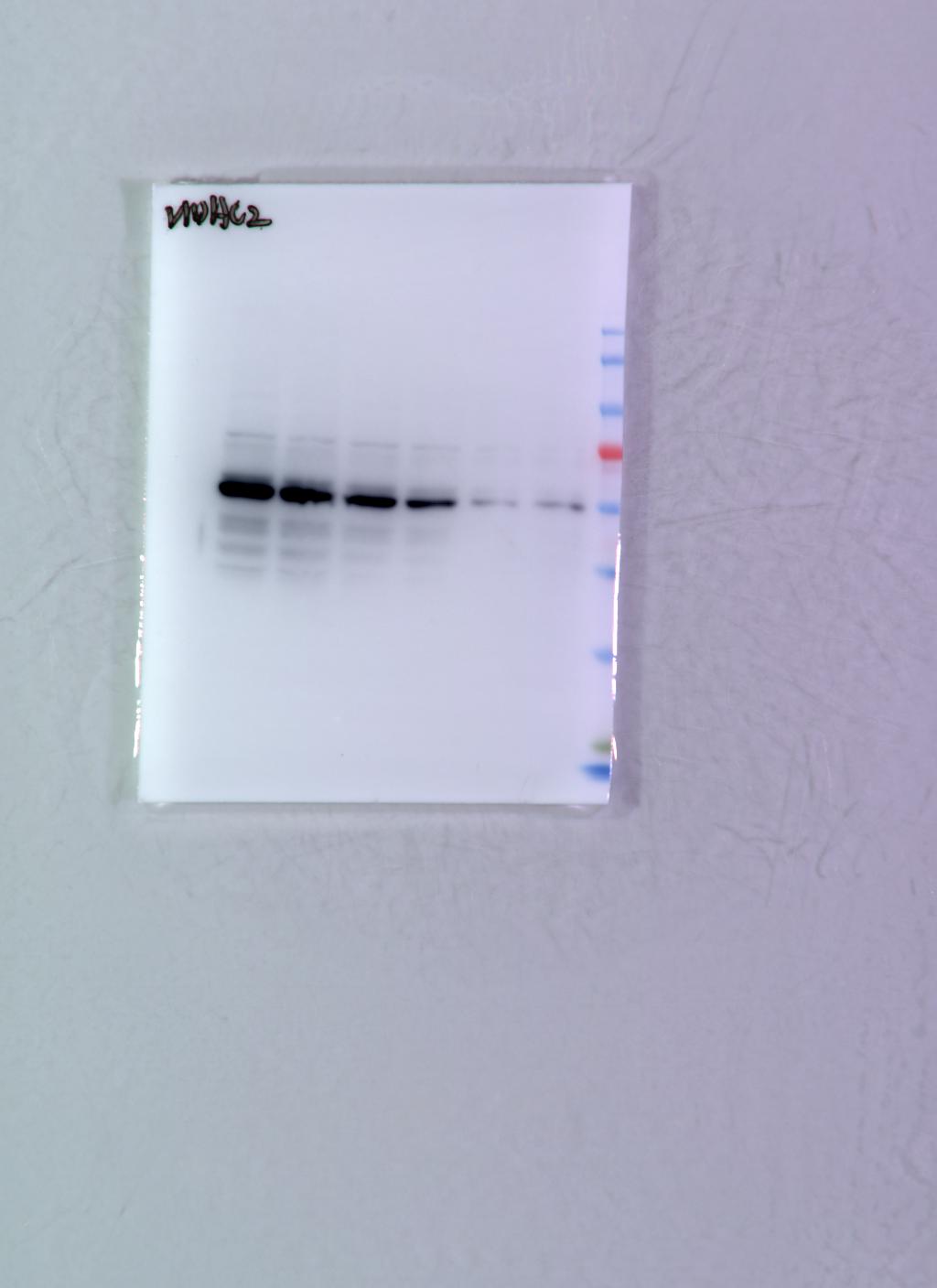

Supplement: Figure 1—source data 2. [file elife-110309-fig1-data2.zip › Figure 1-Source Data 2/HDAC2 1-3 2023.11.25_23.11.20_Ch+Marker.jpg]

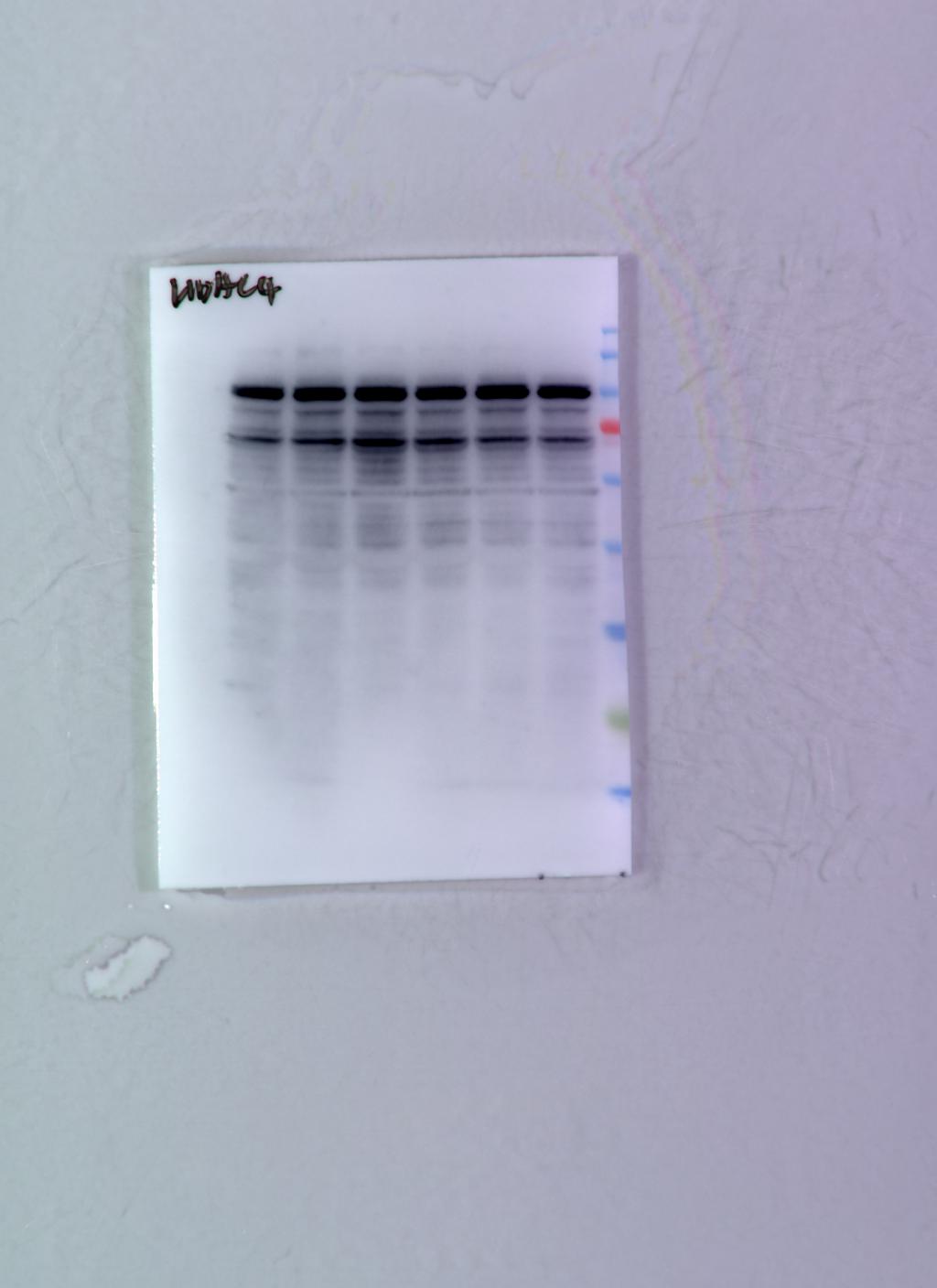

Supplement: Figure 1—source data 2. [file elife-110309-fig1-data2.zip › Figure 1-Source Data 2/HDAC4 1-3 2023.11.25_21.40.22_Ch+Marker.jpg]

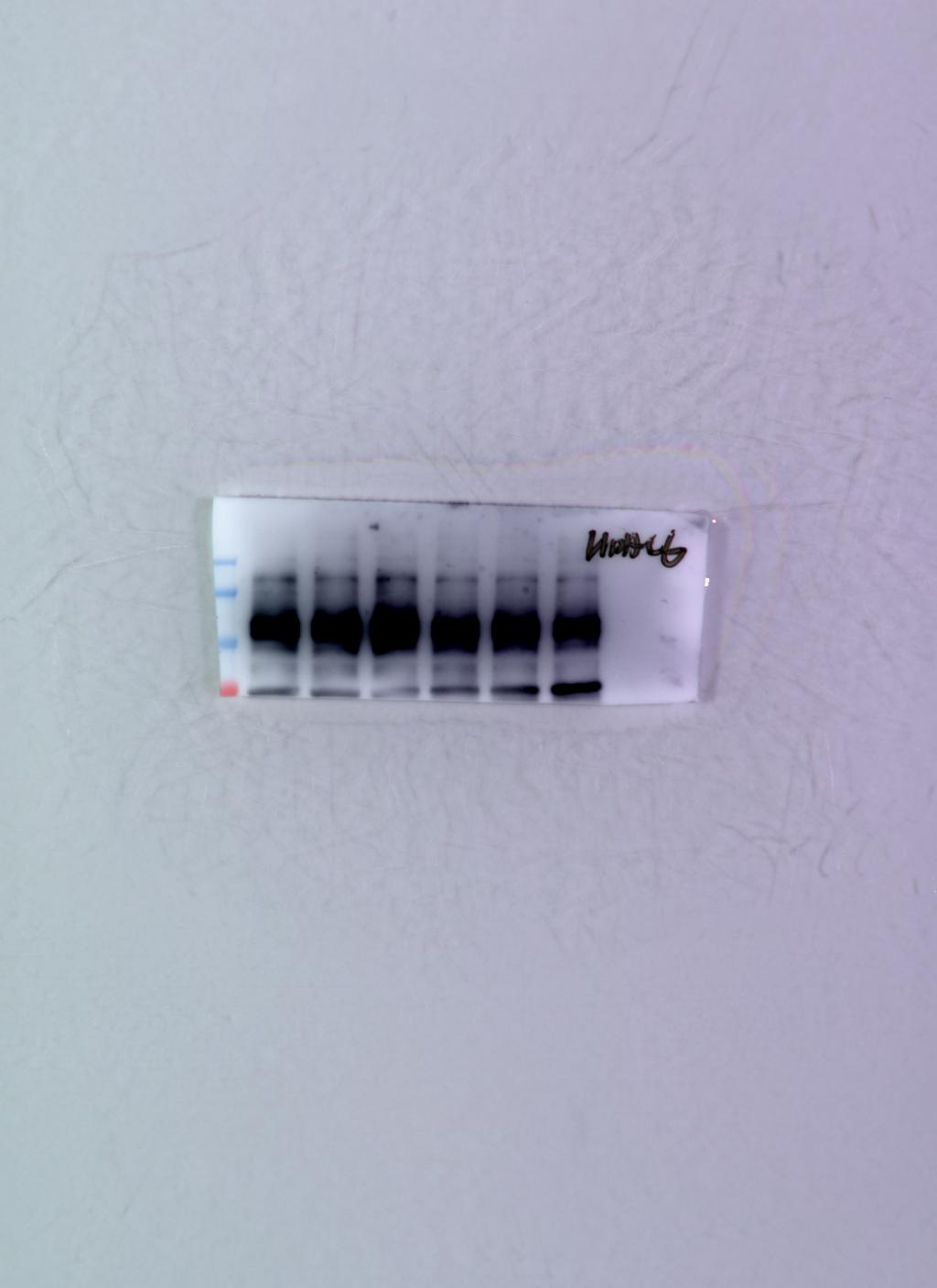

Supplement: Figure 1—source data 2. [file elife-110309-fig1-data2.zip › Figure 1-Source Data 2/HDAC6 2-4 2023.11.25_22.56.56_Ch+Marker.jpg]

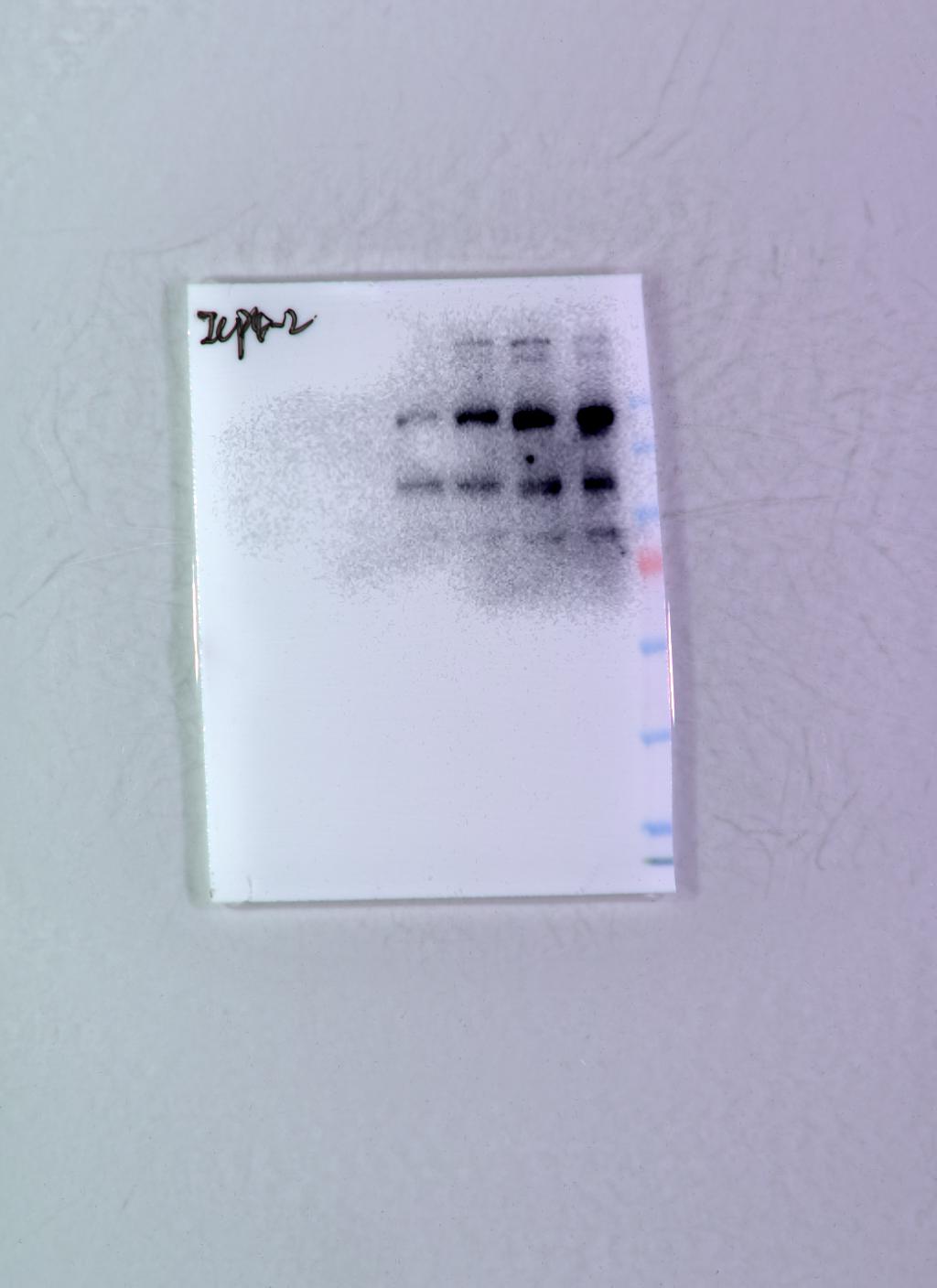

Supplement: Figure 1—source data 2. [file elife-110309-fig1-data2.zip › Figure 1-Source Data 2/ICP4 1-2 2026.03.24_20.55.30_Ch+Marker.jpg]

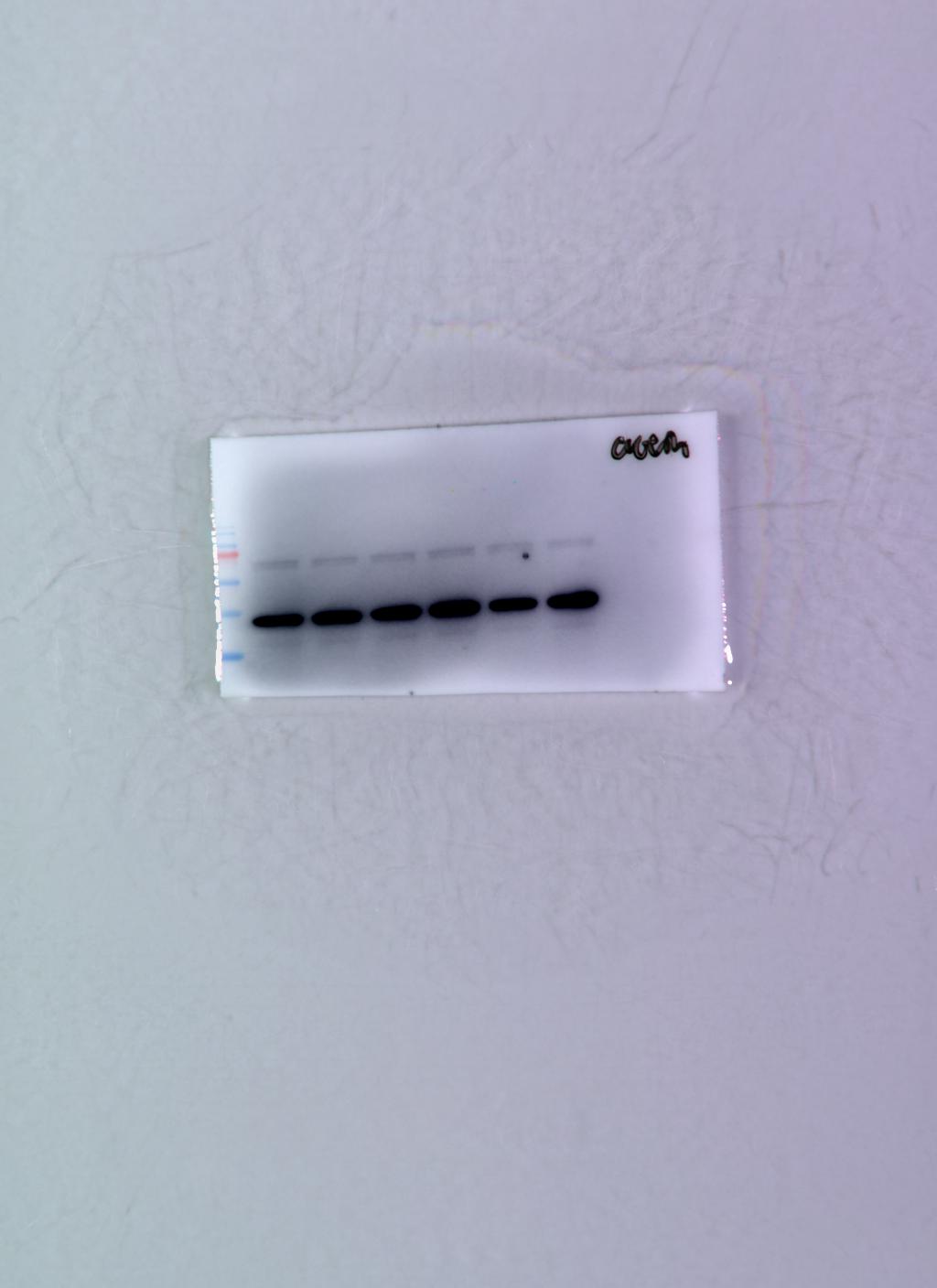

Supplement: Figure 1—source data 2. [file elife-110309-fig1-data2.zip › Figure 1-Source Data 4/ACTIN 0-3 2023.11.25_23.32.52_Ch+Marker.jpg]

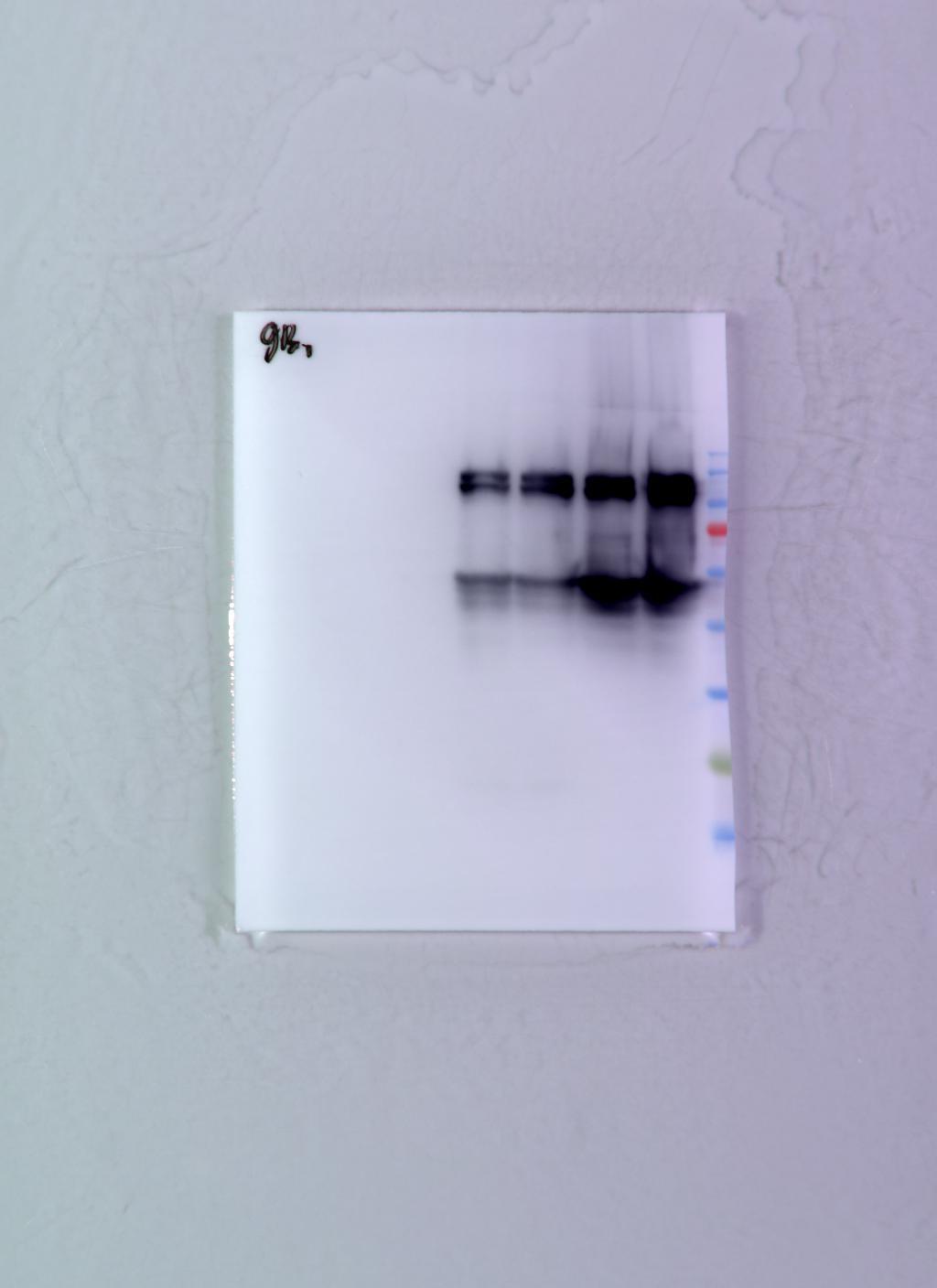

Supplement: Figure 1—source data 2. [file elife-110309-fig1-data2.zip › Figure 1-Source Data 4/GB 0-6 2023.12.03_17.41.02_Ch+Marker.jpg]

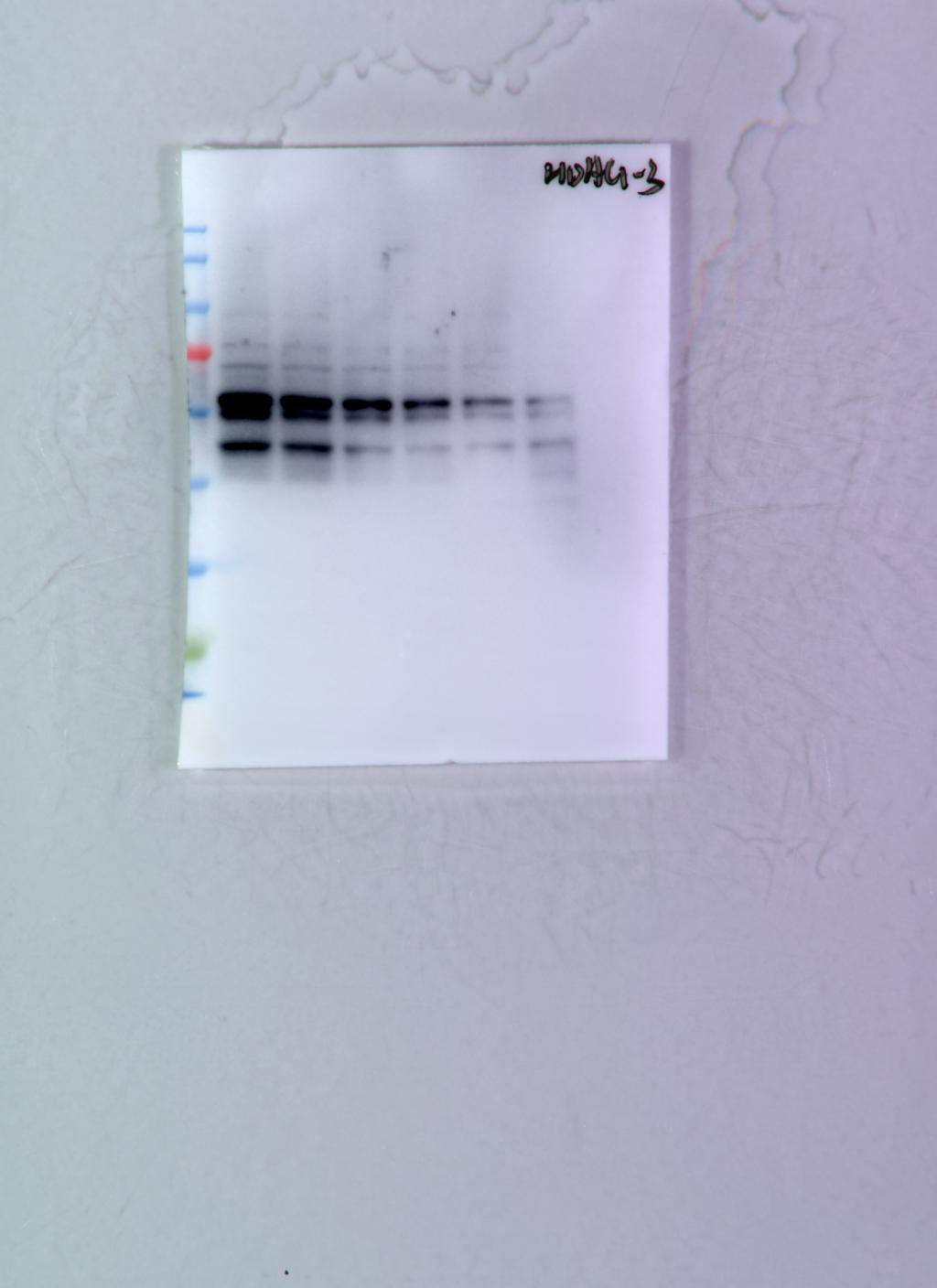

Supplement: Figure 1—source data 2. [file elife-110309-fig1-data2.zip › Figure 1-Source Data 4/hdac1 2-0 2023.11.25_21.07.43_Ch+Marker.jpg]

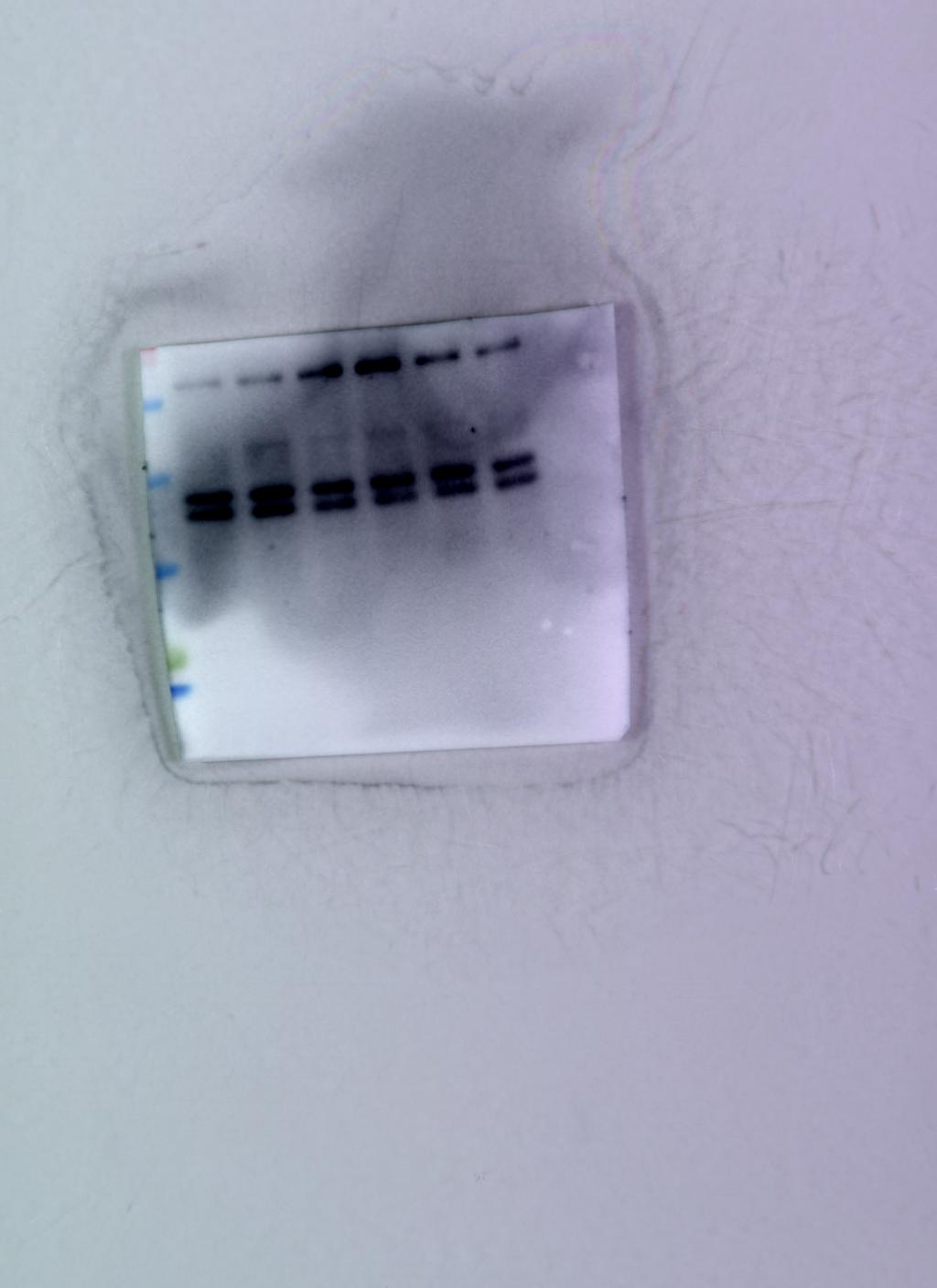

Supplement: Figure 1—source data 2. [file elife-110309-fig1-data2.zip › Figure 1-Source Data 4/HDAC11 2-1 2023.11.26_00.08.15_Ch+Marker.jpg]

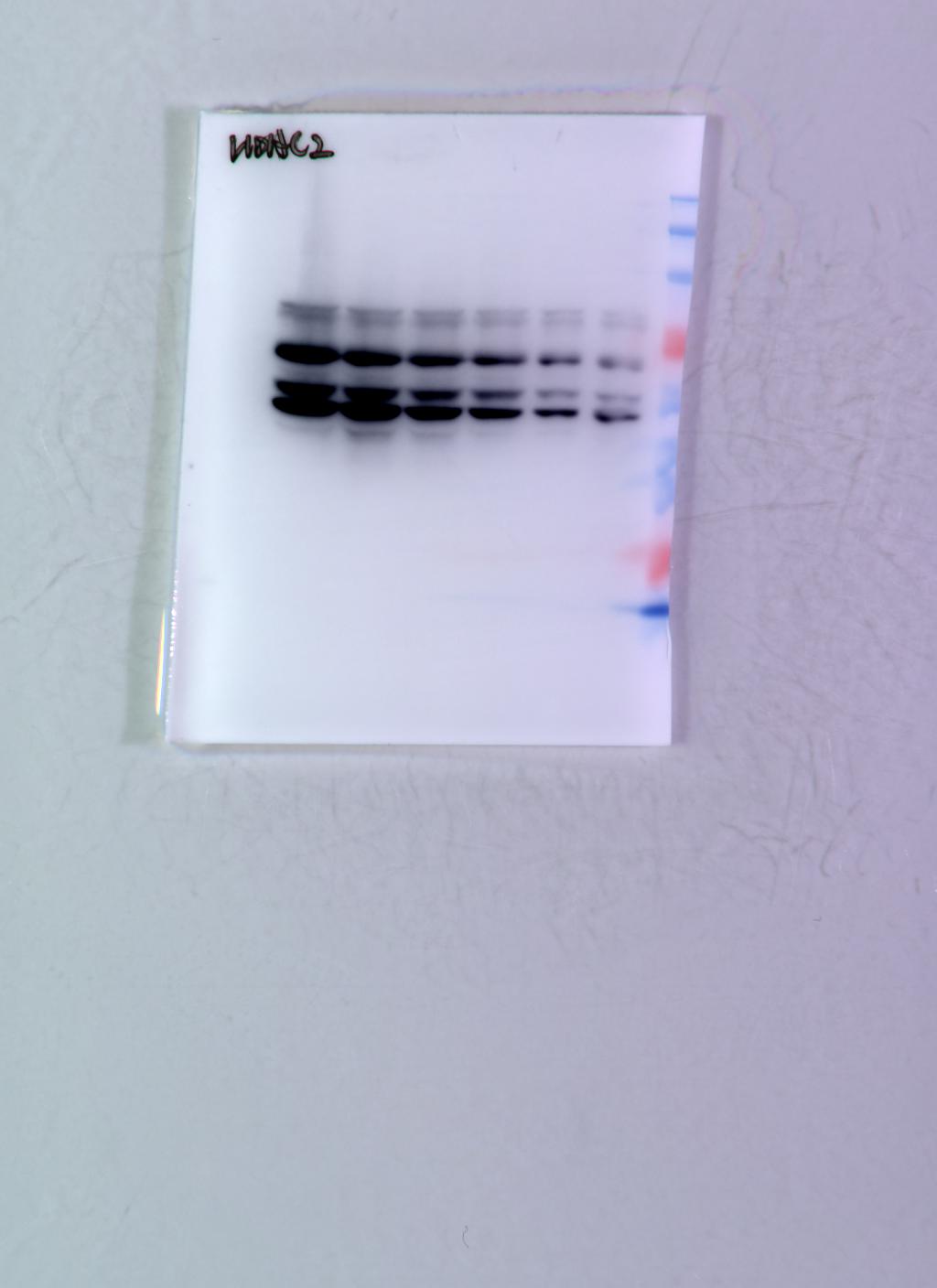

Supplement: Figure 1—source data 2. [file elife-110309-fig1-data2.zip › Figure 1-Source Data 4/HDAC2 0-2 2023.12.10_11.47.11_Ch+Marker.jpg]

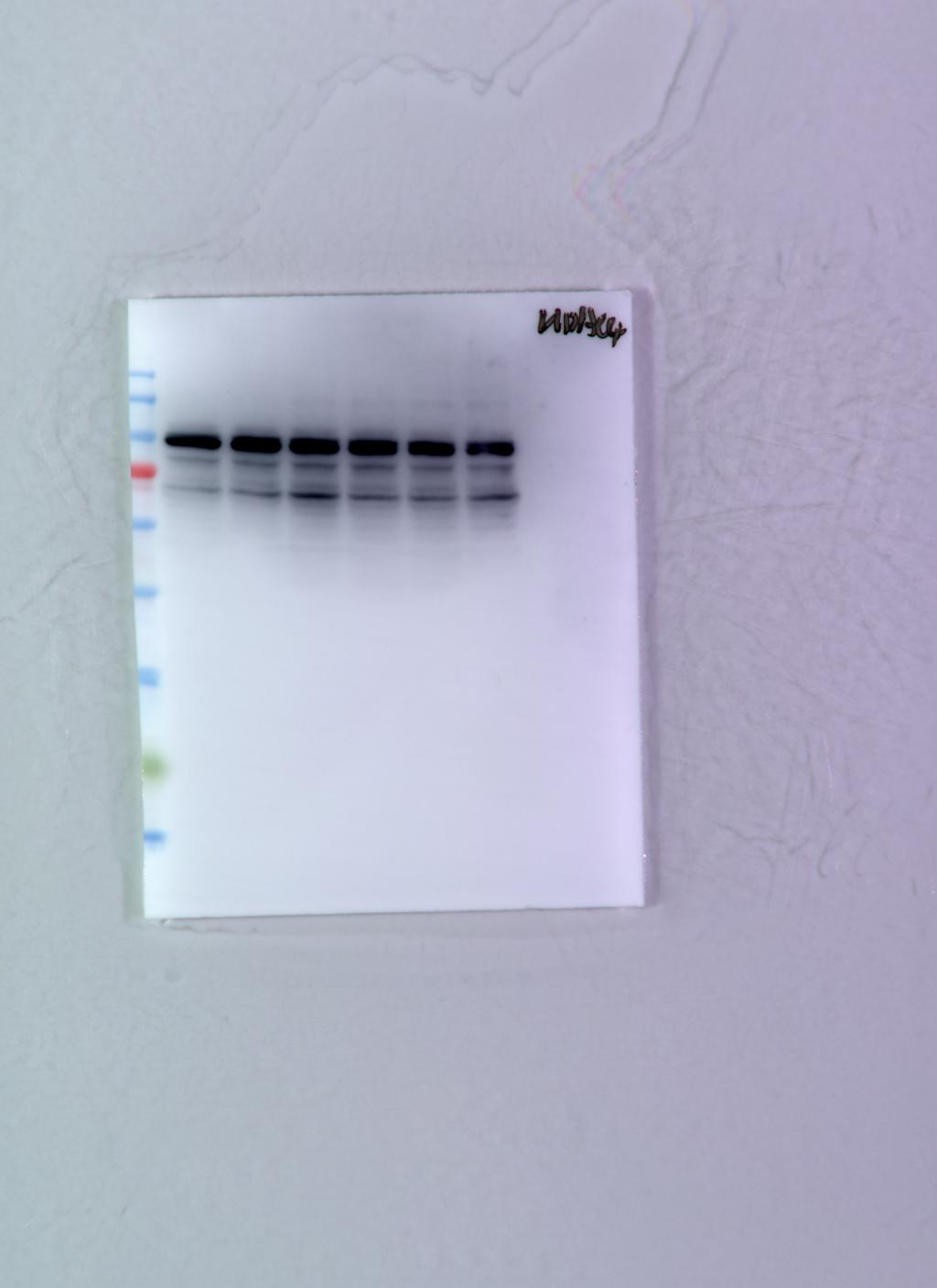

Supplement: Figure 1—source data 2. [file elife-110309-fig1-data2.zip › Figure 1-Source Data 4/HDAC4 2-4 2023.11.25_21.52.59_Ch+Marker.jpg]

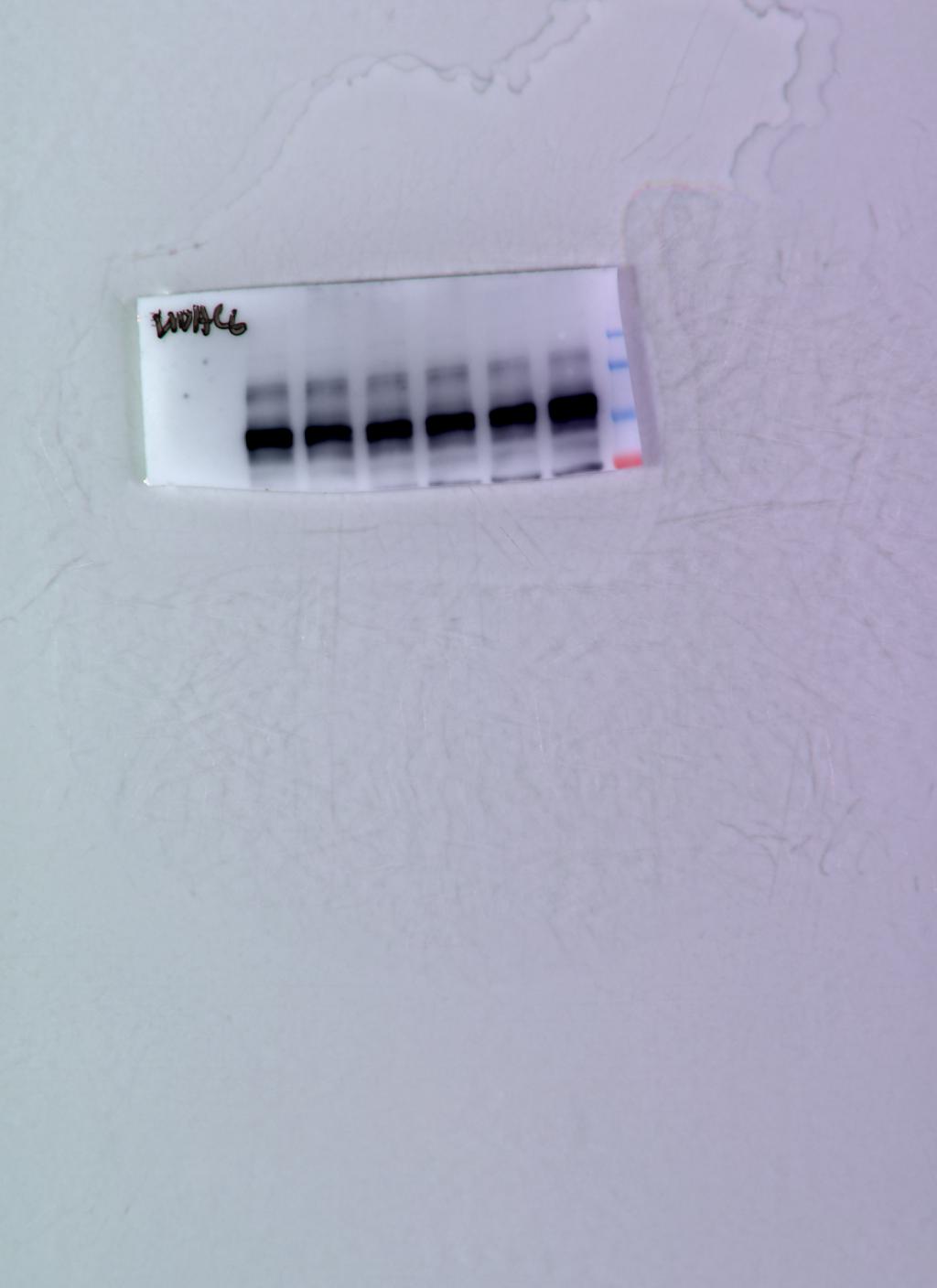

Supplement: Figure 1—source data 2. [file elife-110309-fig1-data2.zip › Figure 1-Source Data 4/HDAC6 1-4 2023.11.25_22.42.46_Ch+Marker.jpg]

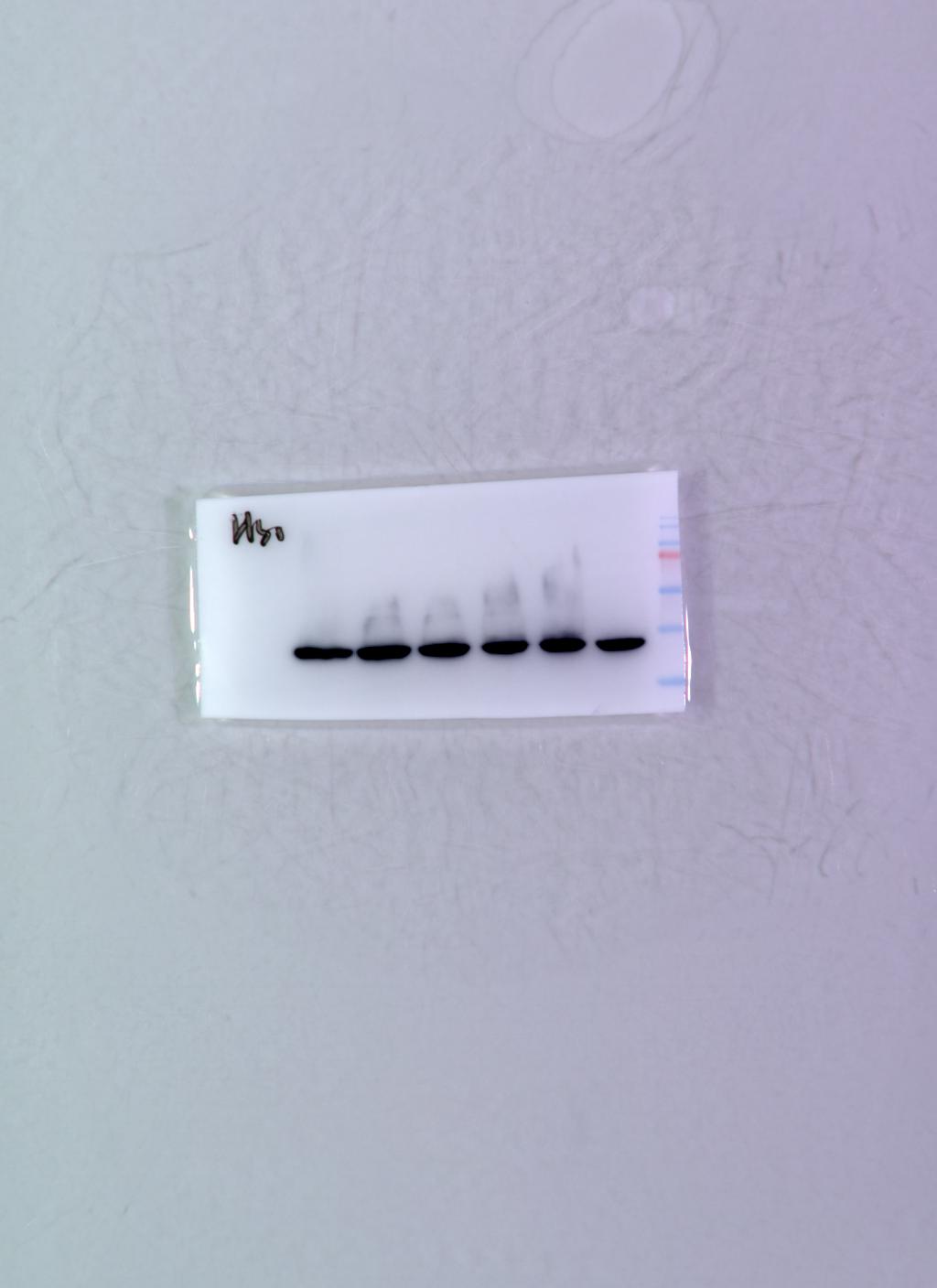

Supplement: Figure 1—source data 2. [file elife-110309-fig1-data2.zip › Figure 1-Source Data 6/ACTIN 3-1 2023.11.26_11.54.32_Ch+Marker.jpg]

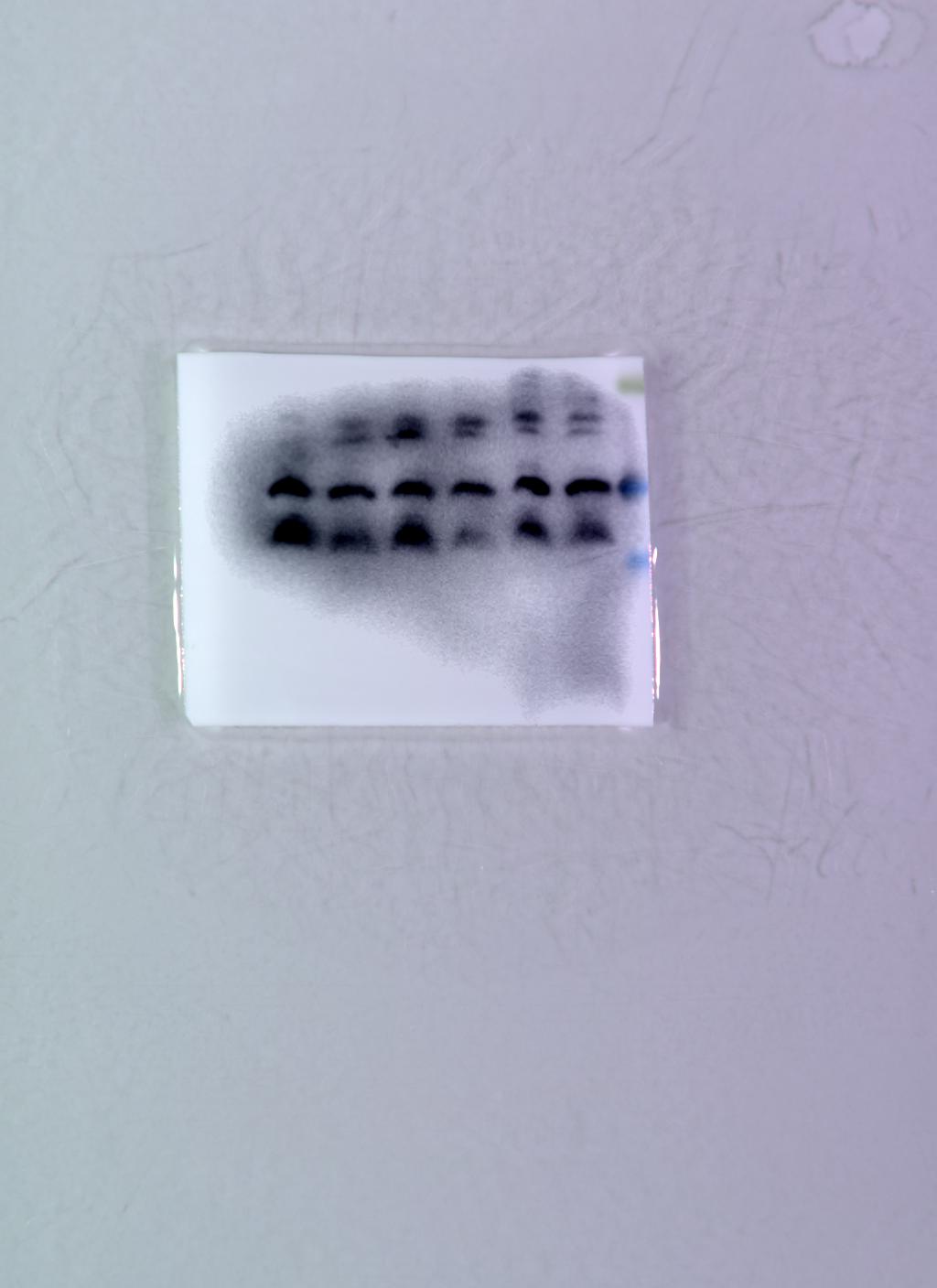

Supplement: Figure 1—source data 2. [file elife-110309-fig1-data2.zip › Figure 1-Source Data 6/H3 4-2 2023.11.26_12.18.19_Ch+Marker.jpg]

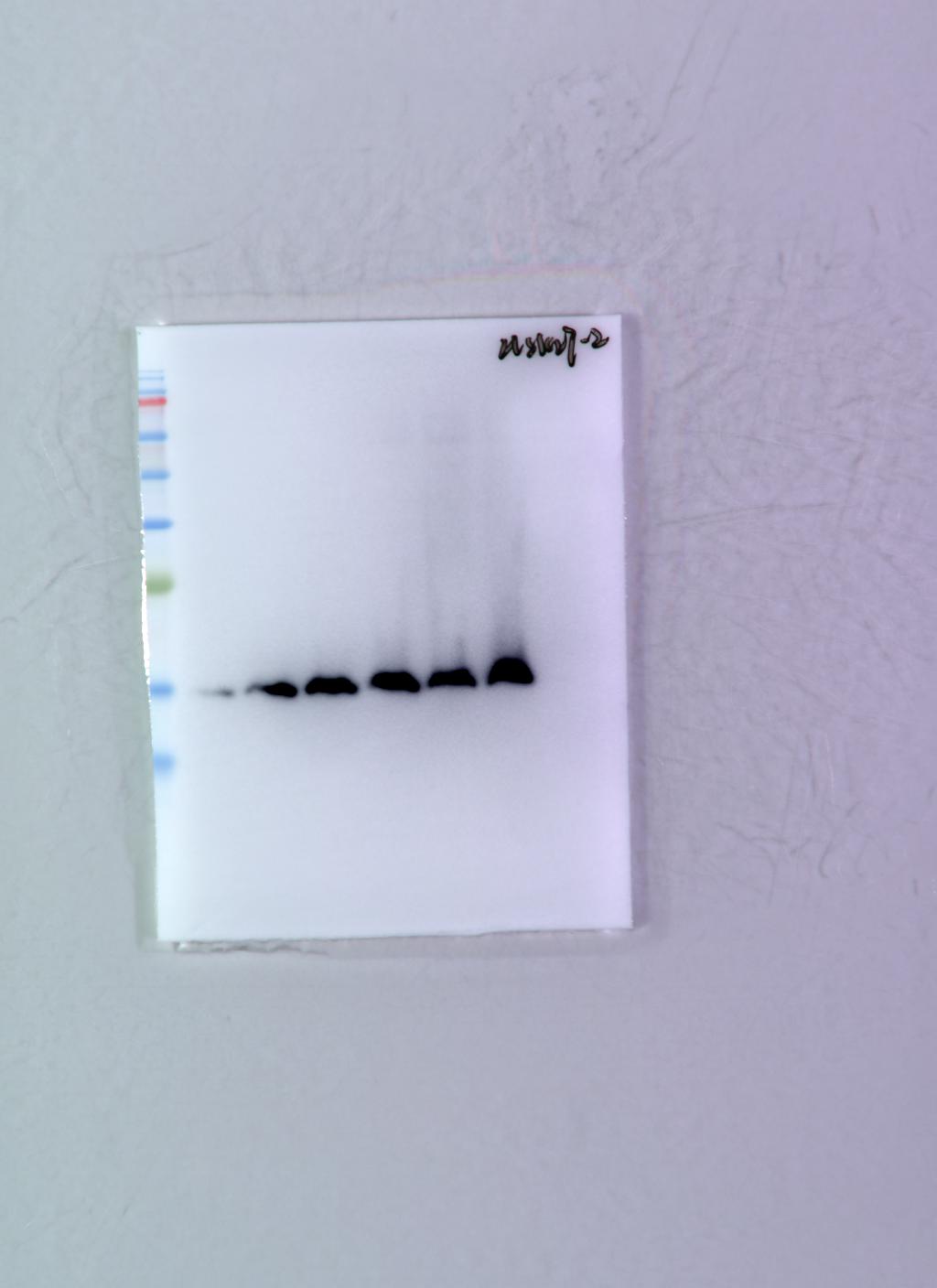

Supplement: Figure 1—source data 2. [file elife-110309-fig1-data2.zip › Figure 1-Source Data 6/H3K27 1-1 2023.11.26_10.28.19_Ch+Marker.jpg]

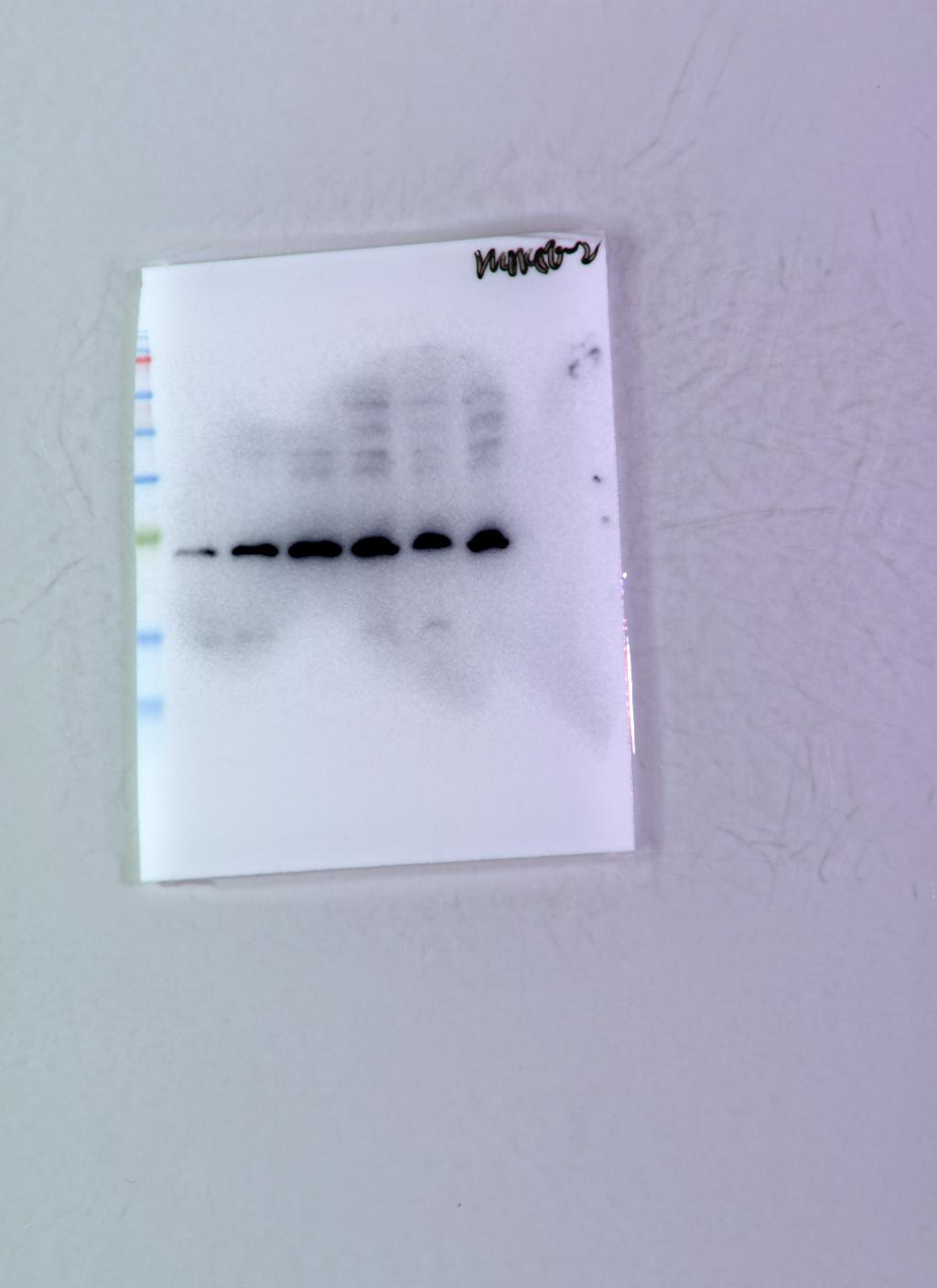

Supplement: Figure 1—source data 2. [file elife-110309-fig1-data2.zip › Figure 1-Source Data 6/H3K56 4-1 2023.11.26_11.45.58_Ch+Marker.jpg]

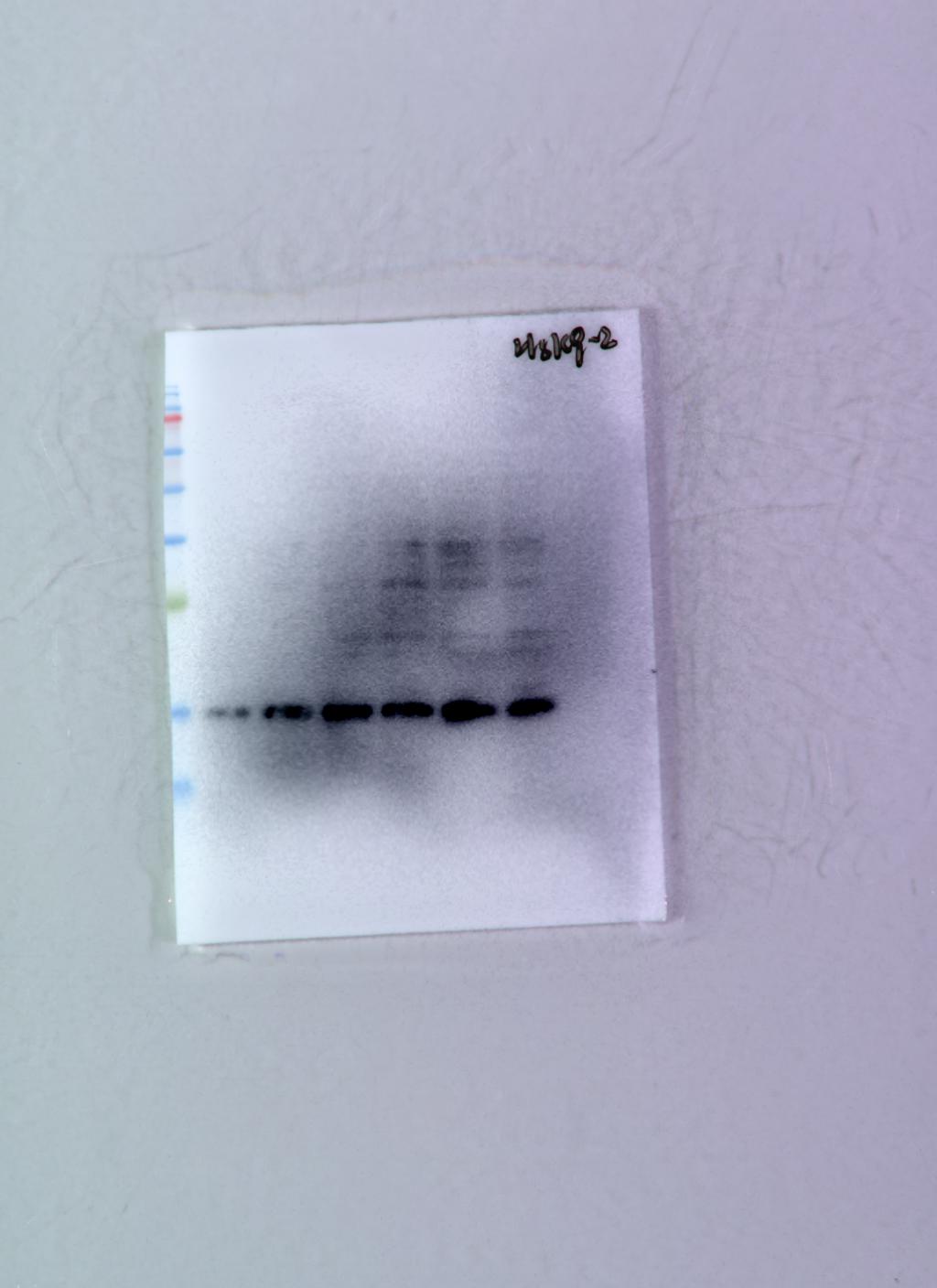

Supplement: Figure 1—source data 2. [file elife-110309-fig1-data2.zip › Figure 1-Source Data 6/H3K9 2-3 2023.11.26_10.39.38_Ch+Marker.jpg]

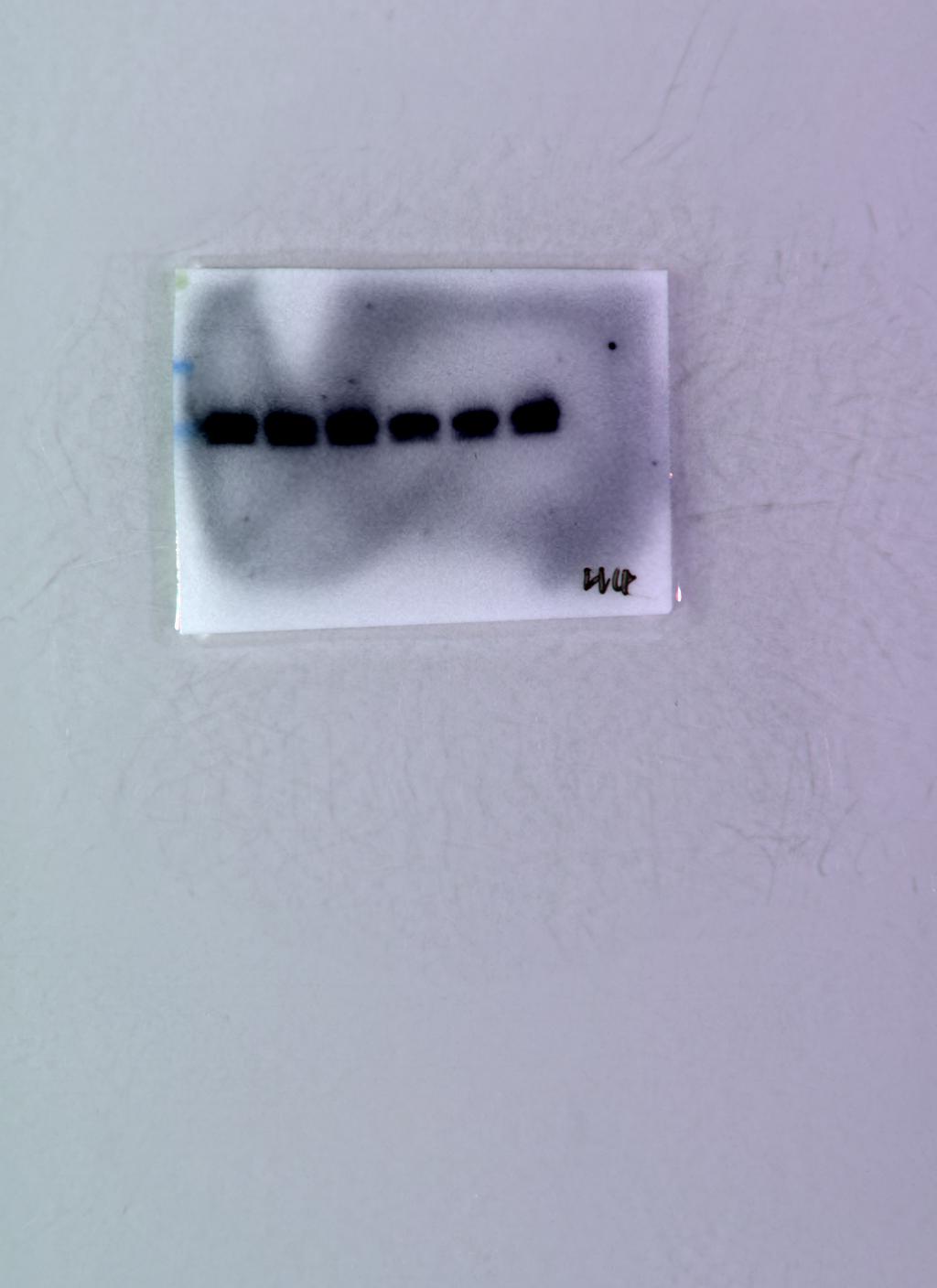

Supplement: Figure 1—source data 2. [file elife-110309-fig1-data2.zip › Figure 1-Source Data 6/H4 0-3 2023.11.25_22.13.00_Ch+Marker.jpg]

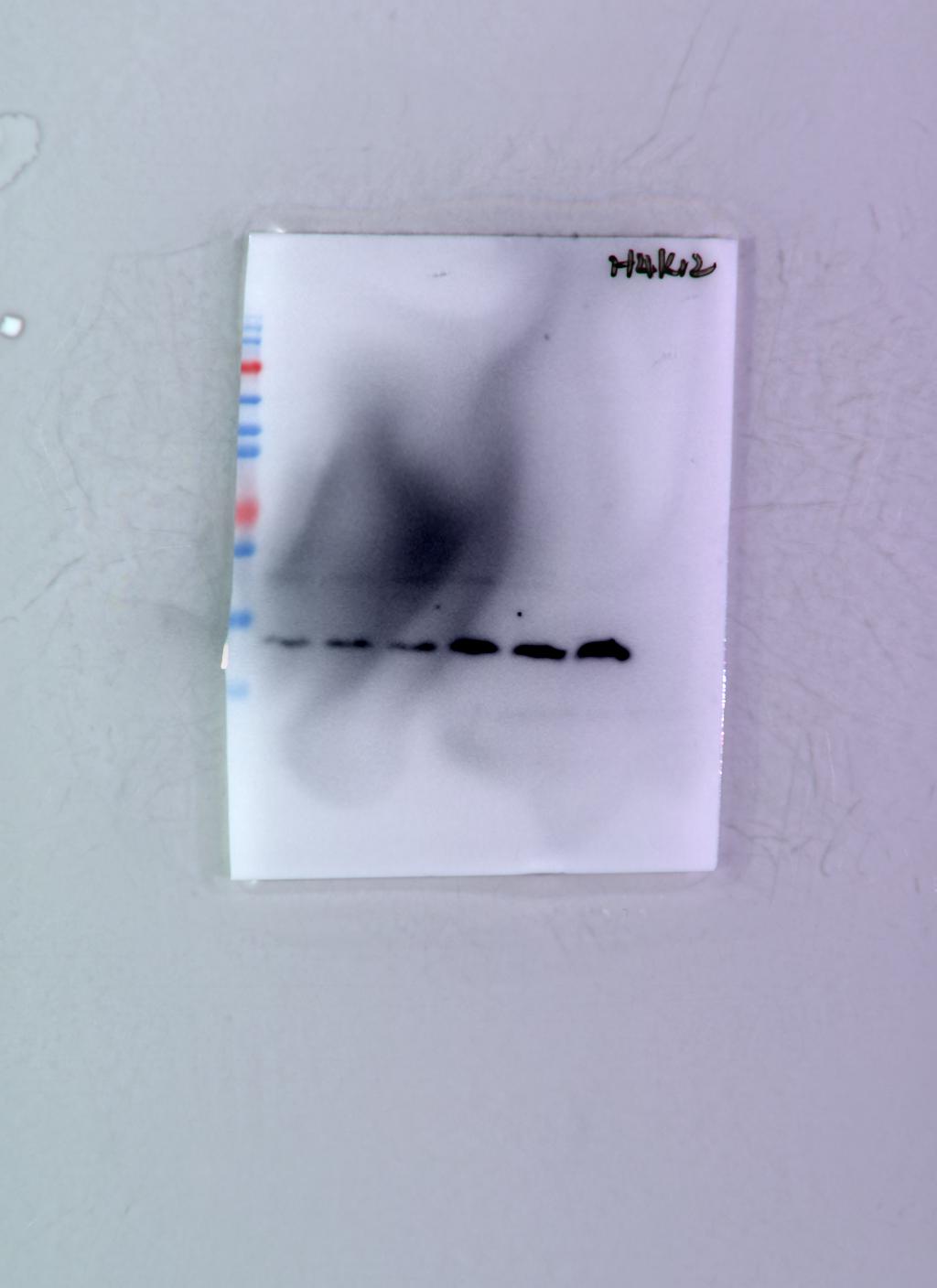

Supplement: Figure 1—source data 2. [file elife-110309-fig1-data2.zip › Figure 1-Source Data 6/H4K12 0-2 2023.12.10_12.23.40_Ch+Marker.jpg]

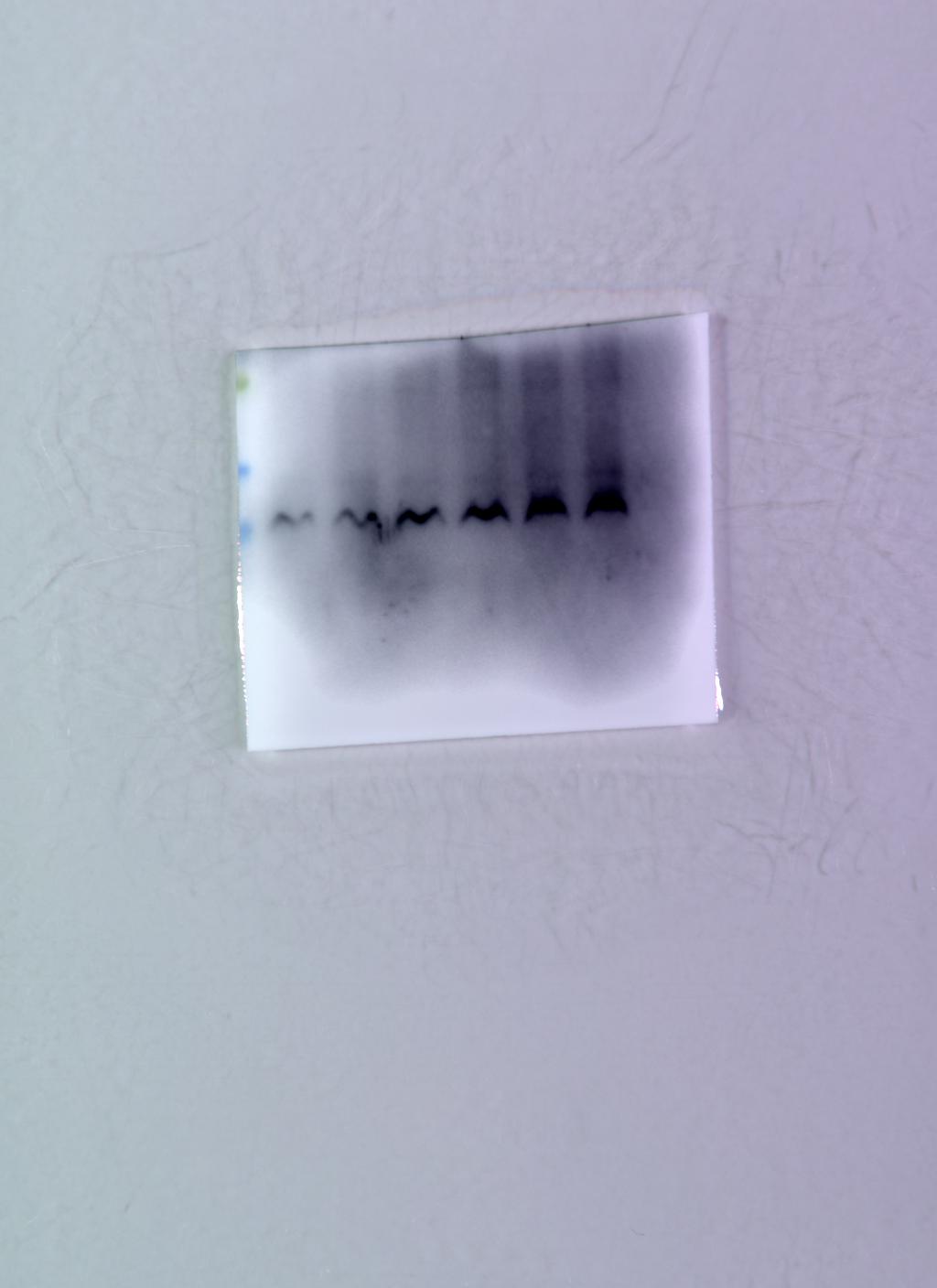

Supplement: Figure 1—source data 2. [file elife-110309-fig1-data2.zip › Figure 1-Source Data 6/H4K8 0-4 2023.11.25_22.23.03_Ch+Marker.jpg]

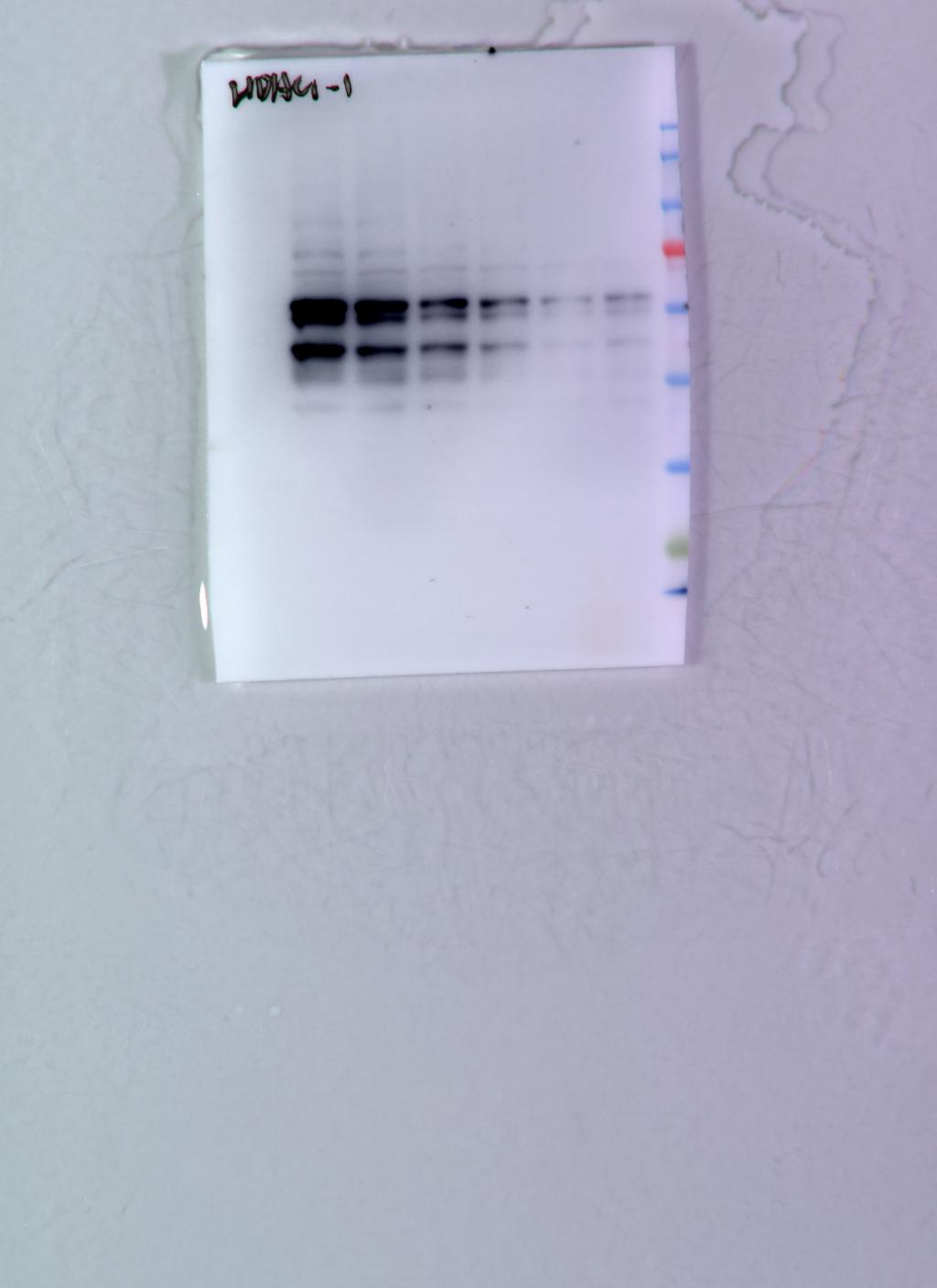

Supplement: Figure 1—source data 2. [file elife-110309-fig1-data2.zip › Figure 1-Source Data 6/hdac1 1-2 2023.11.25_20.57.30_Ch+Marker.jpg]

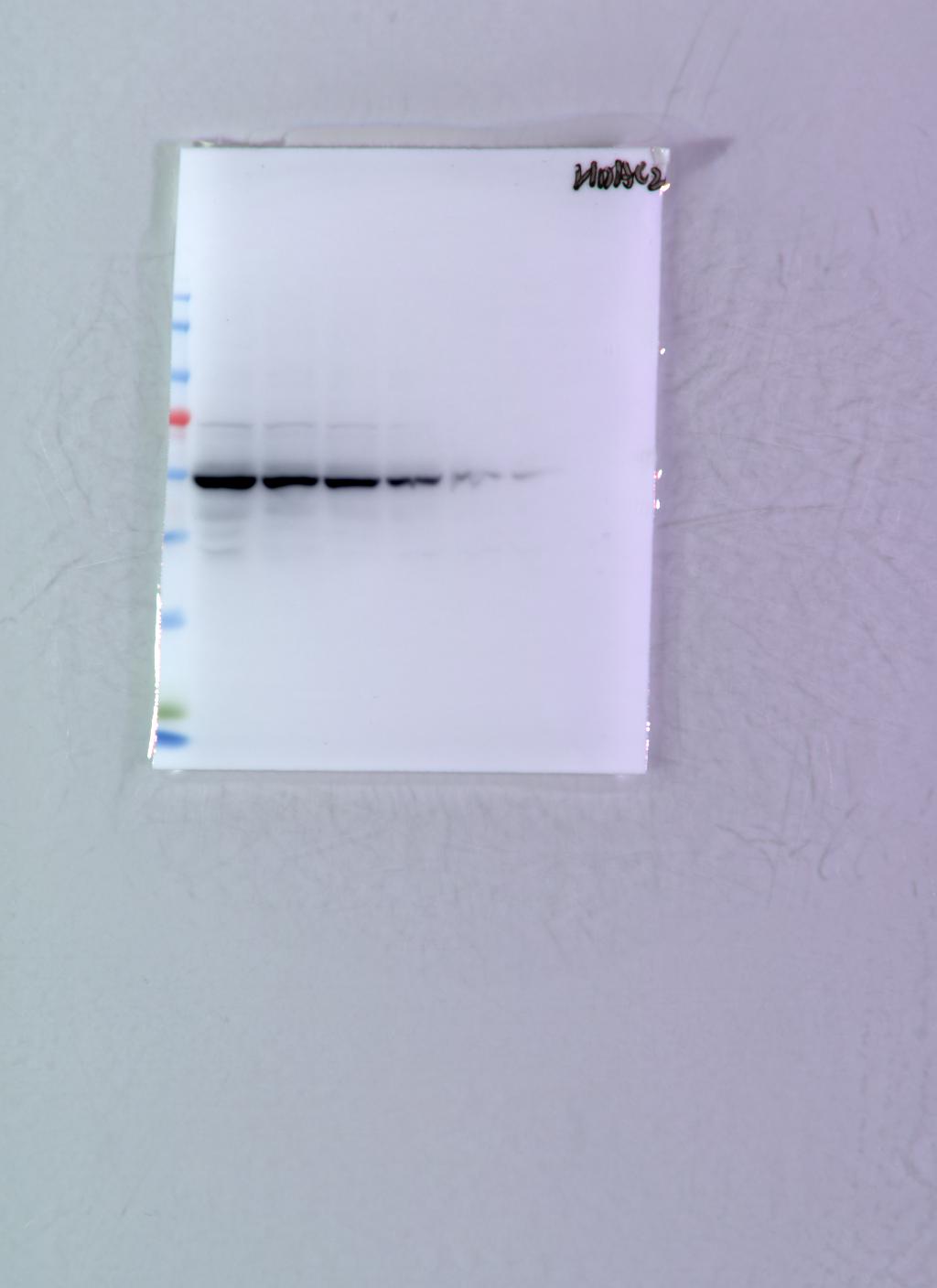

Supplement: Figure 1—source data 2. [file elife-110309-fig1-data2.zip › Figure 1-Source Data 6/HDAC2 0-1 2023.11.25_23.07.59_Ch+Marker.jpg]

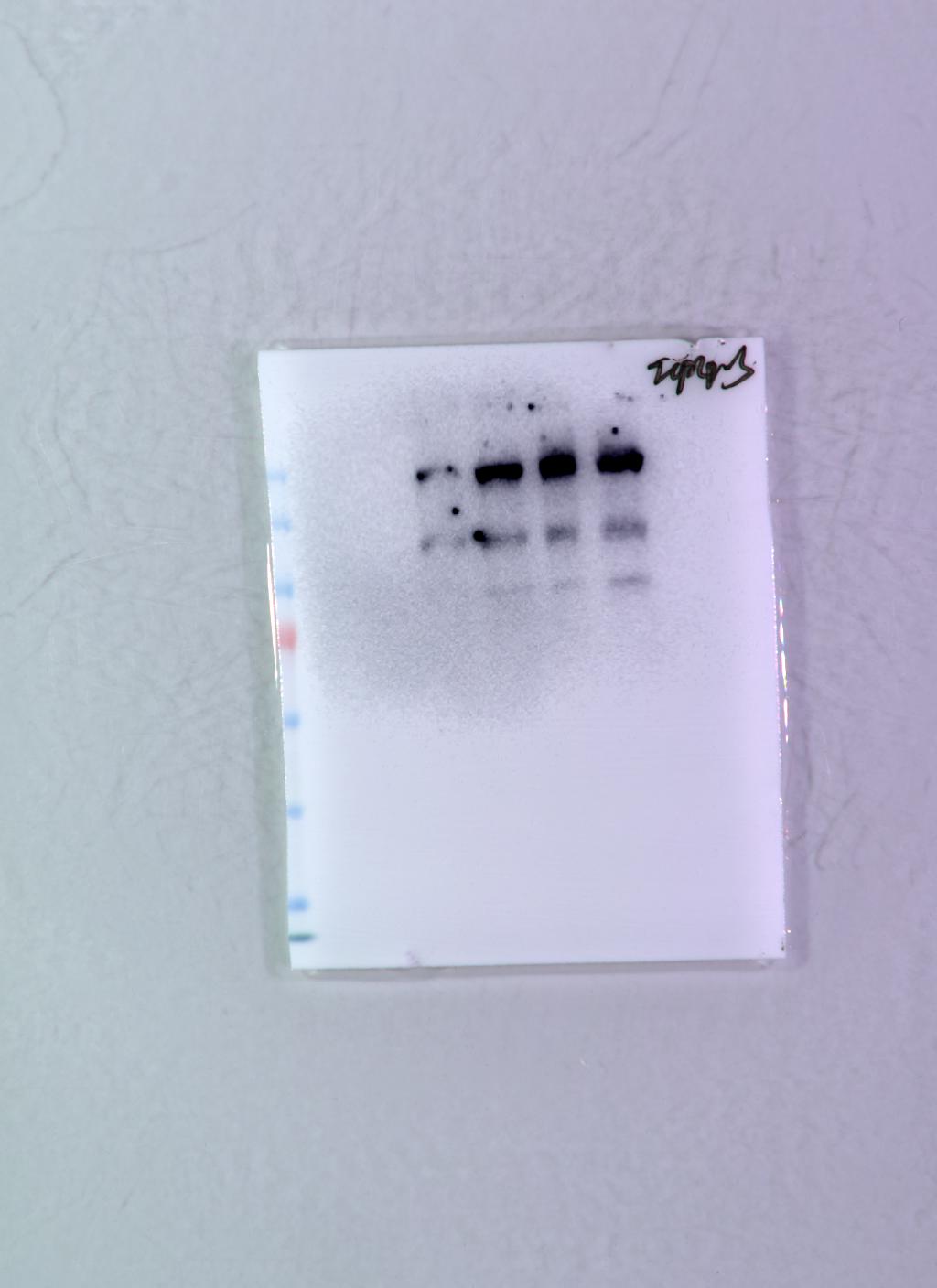

Supplement: Figure 1—source data 2. [file elife-110309-fig1-data2.zip › Figure 1-Source Data 6/ICP4 2-0 2026.03.24_21.00.27_Ch+Marker.jpg]

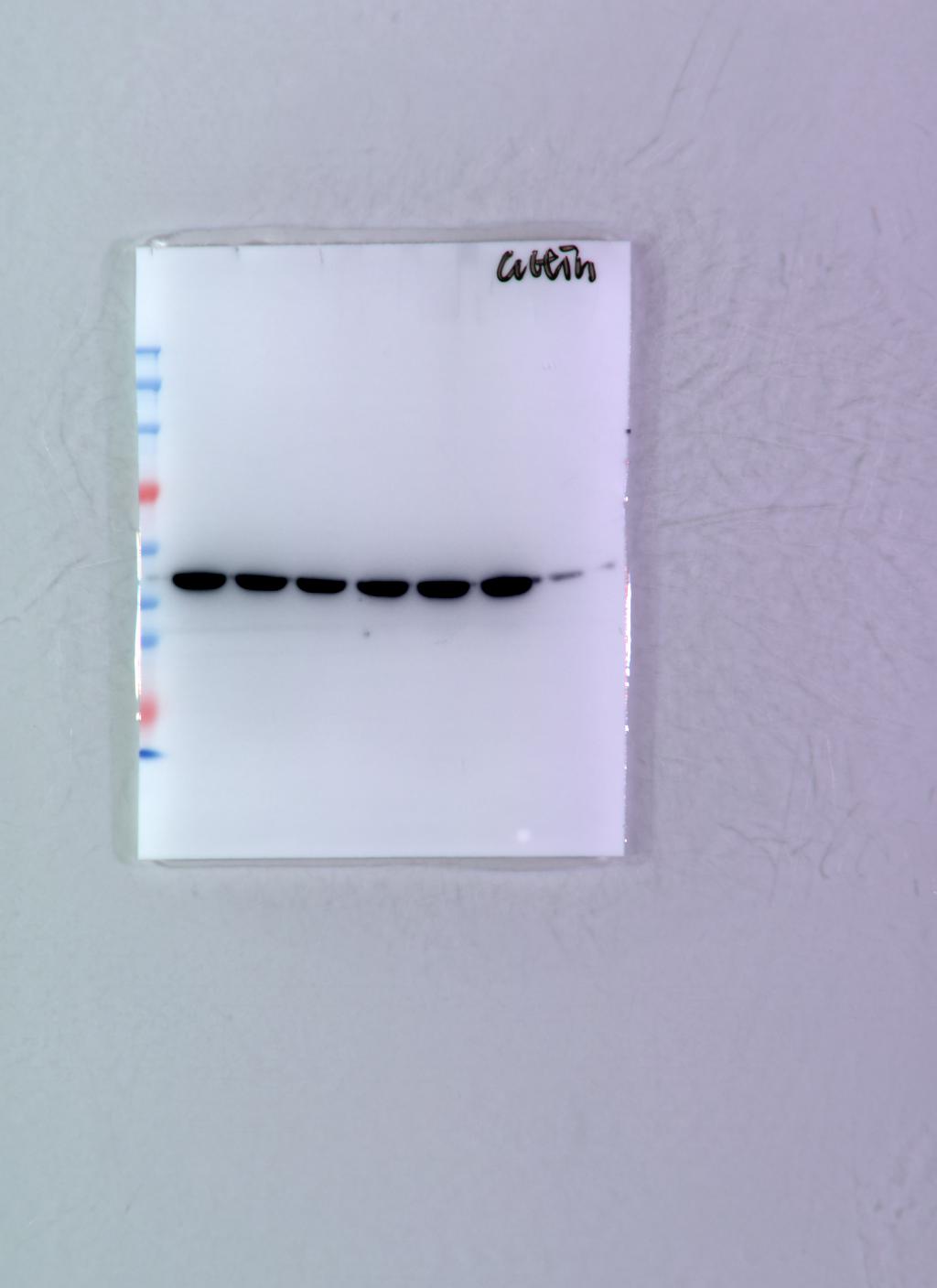

Supplement: Figure 1—source data 2. [file elife-110309-fig1-data2.zip › Figure 1-Source Data 8/ACTIN 0-3 2023.12.10_12.45.54_Ch+Marker.jpg]

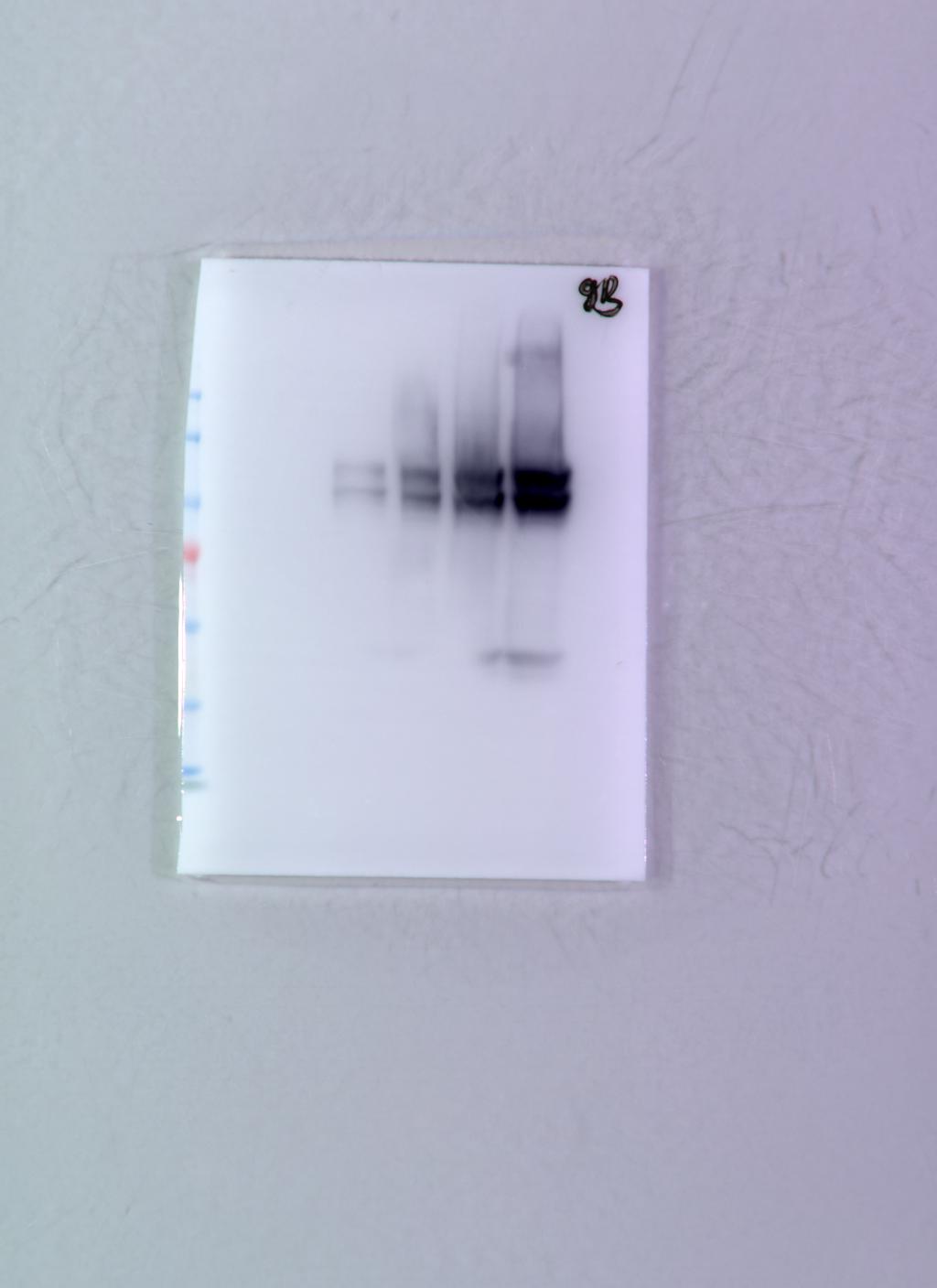

Supplement: Figure 1—source data 2. [file elife-110309-fig1-data2.zip › Figure 1-Source Data 8/GB 0-1 2023.12.02_12.44.50_Ch+Marker.jpg]

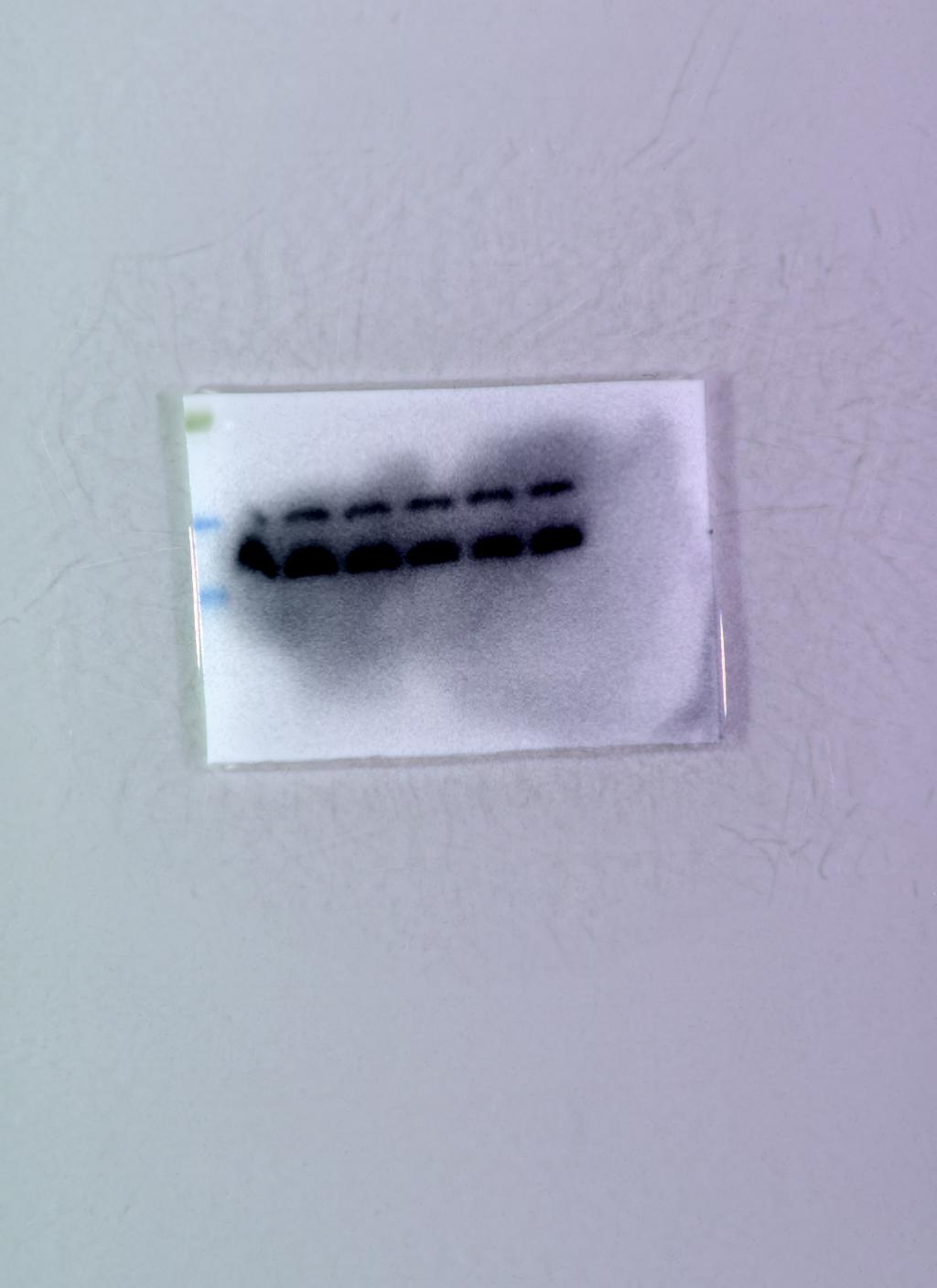

Supplement: Figure 1—source data 2. [file elife-110309-fig1-data2.zip › Figure 1-Source Data 8/H3 3-2 2023.11.26_12.04.47_Ch+Marker.jpg]

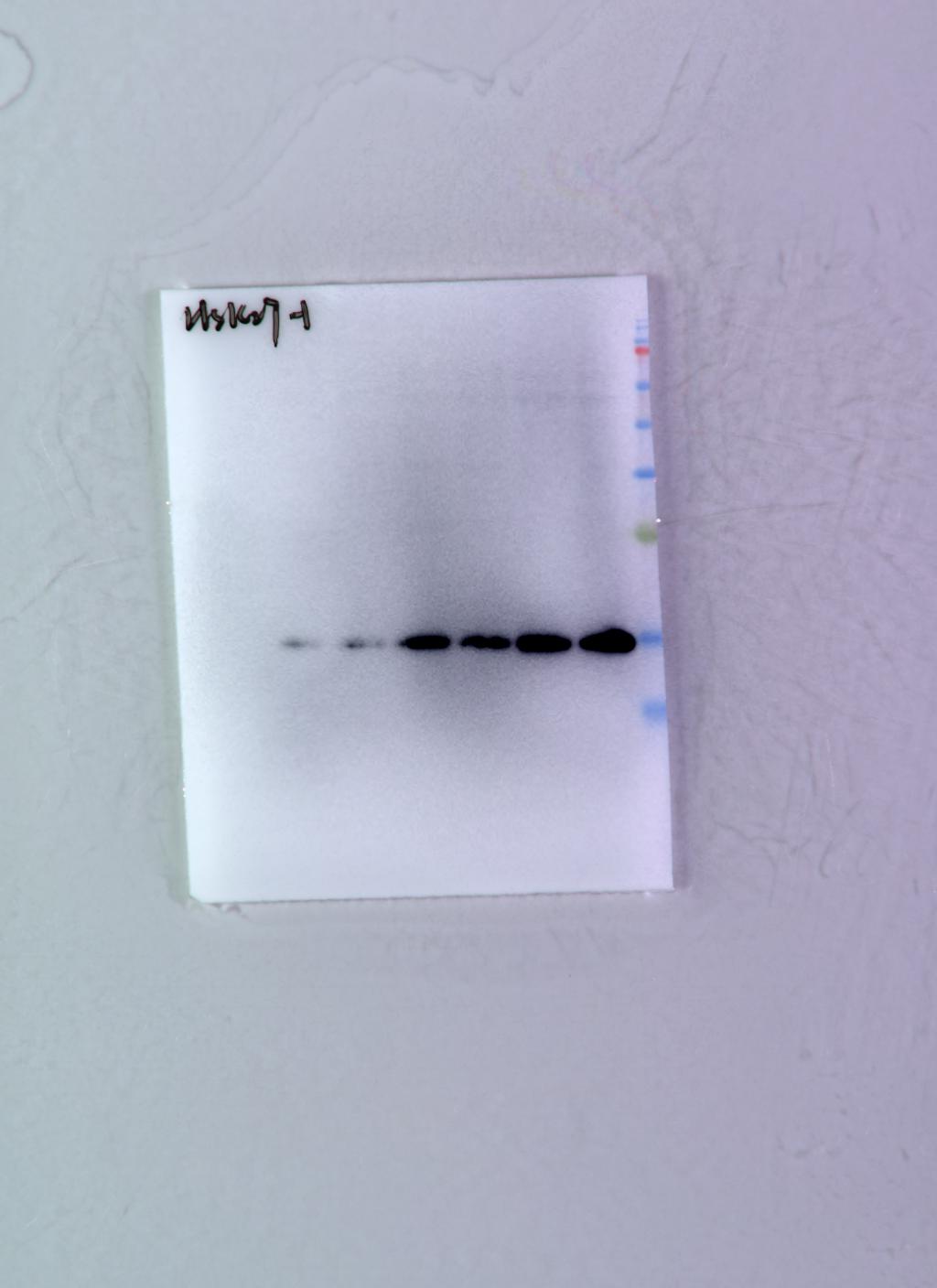

Supplement: Figure 1—source data 2. [file elife-110309-fig1-data2.zip › Figure 1-Source Data 8/H3K27 0-7 2023.11.26_10.22.20_Ch+Marker.jpg]

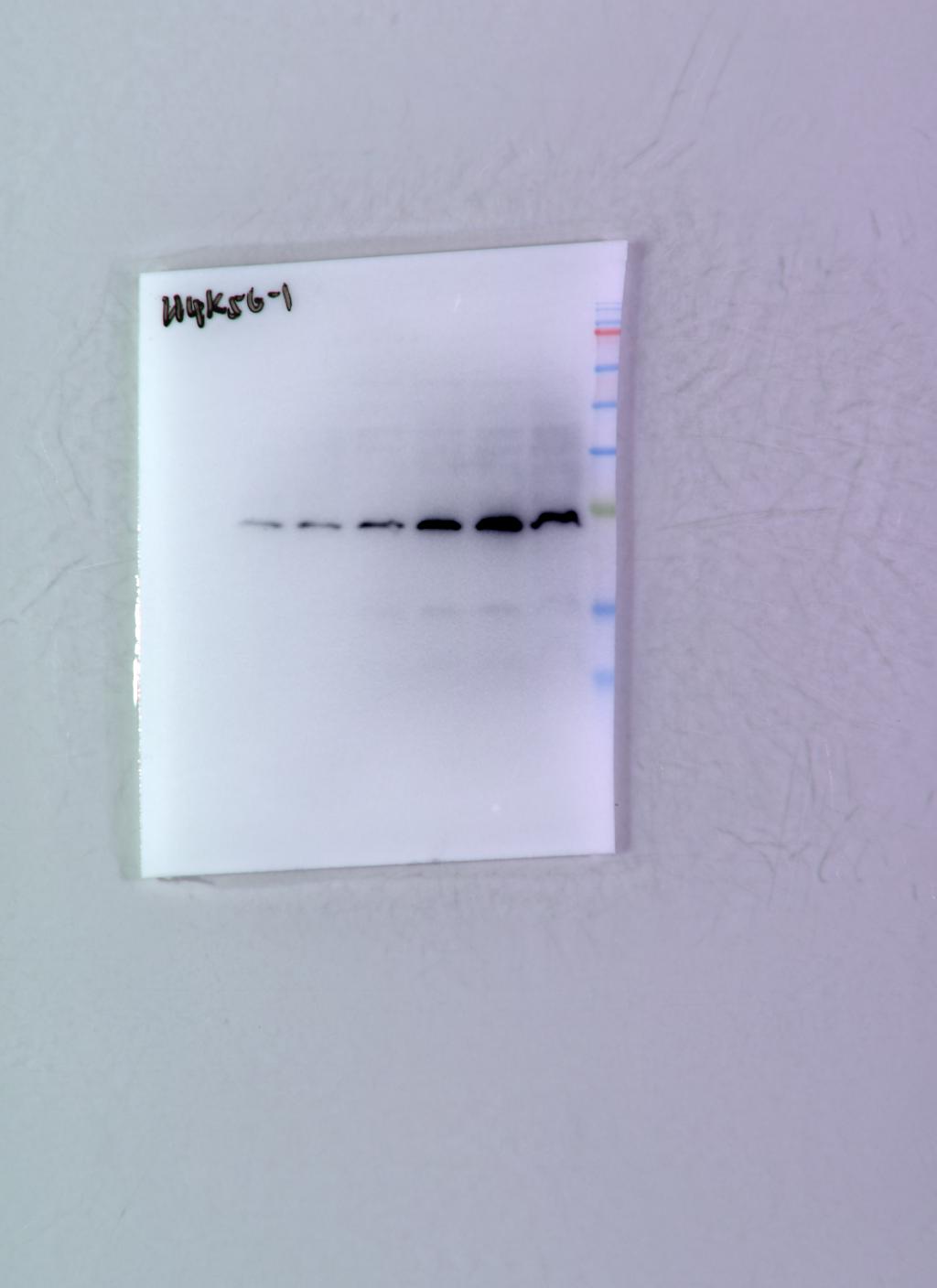

Supplement: Figure 1—source data 2. [file elife-110309-fig1-data2.zip › Figure 1-Source Data 8/H3K56 3-3 2023.11.26_11.42.48_Ch+Marker.jpg]

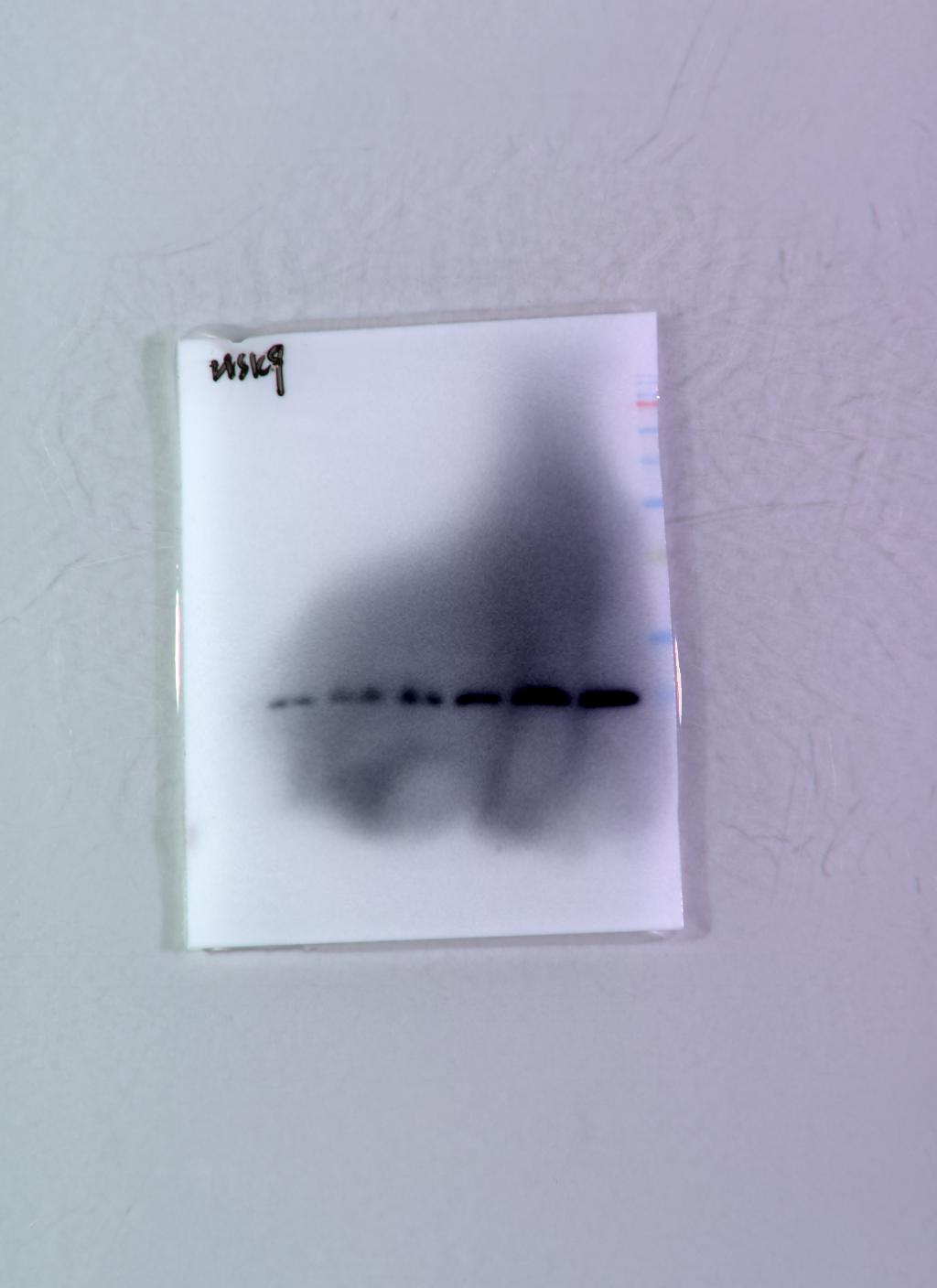

Supplement: Figure 1—source data 2. [file elife-110309-fig1-data2.zip › Figure 1-Source Data 8/H3K9 5-1 2023.12.02_13.37.59_Ch+Marker.jpg]

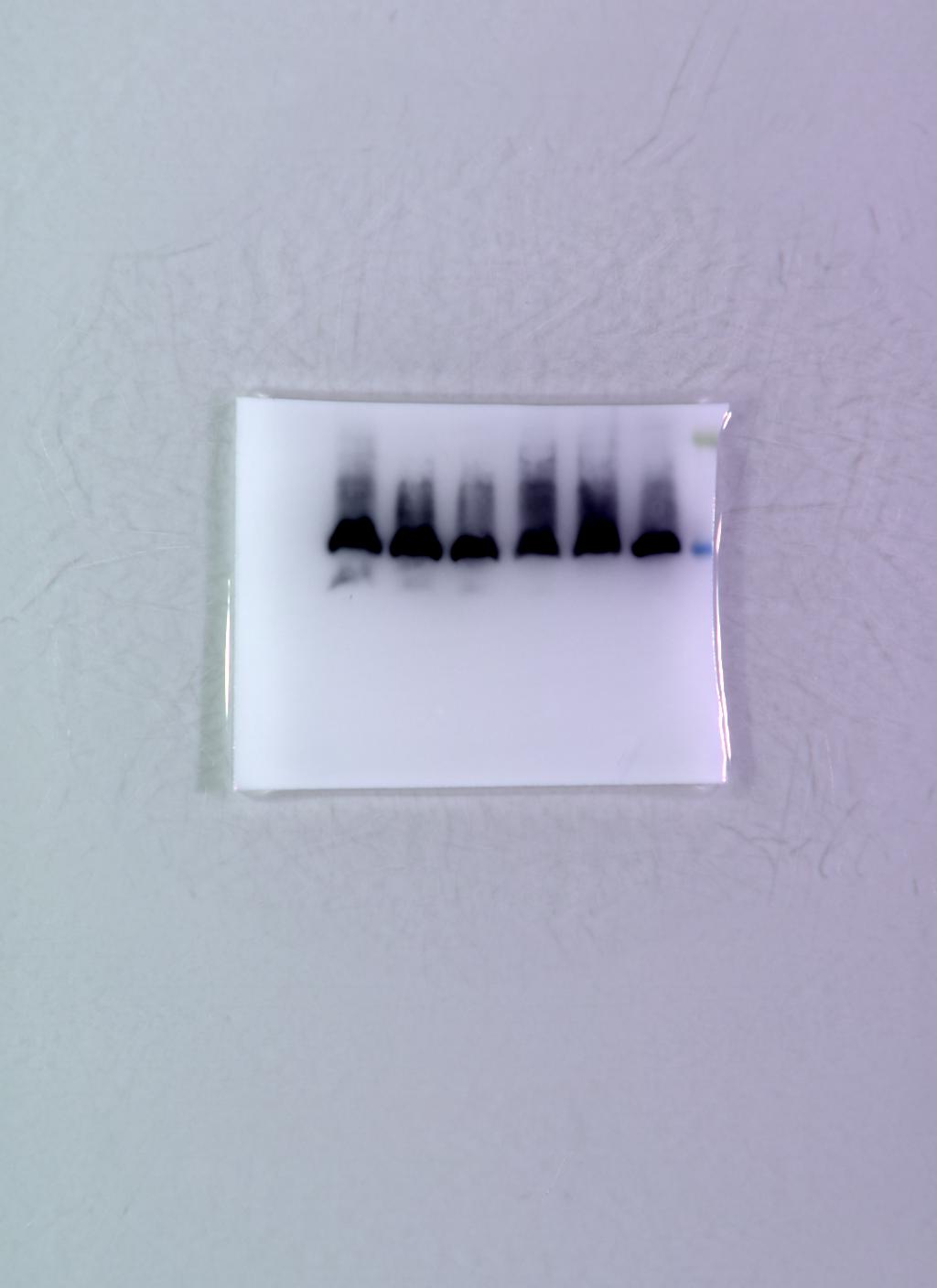

Supplement: Figure 1—source data 2. [file elife-110309-fig1-data2.zip › Figure 1-Source Data 8/H4 3-4 2023.11.26_12.28.18_Ch+Marker.jpg]

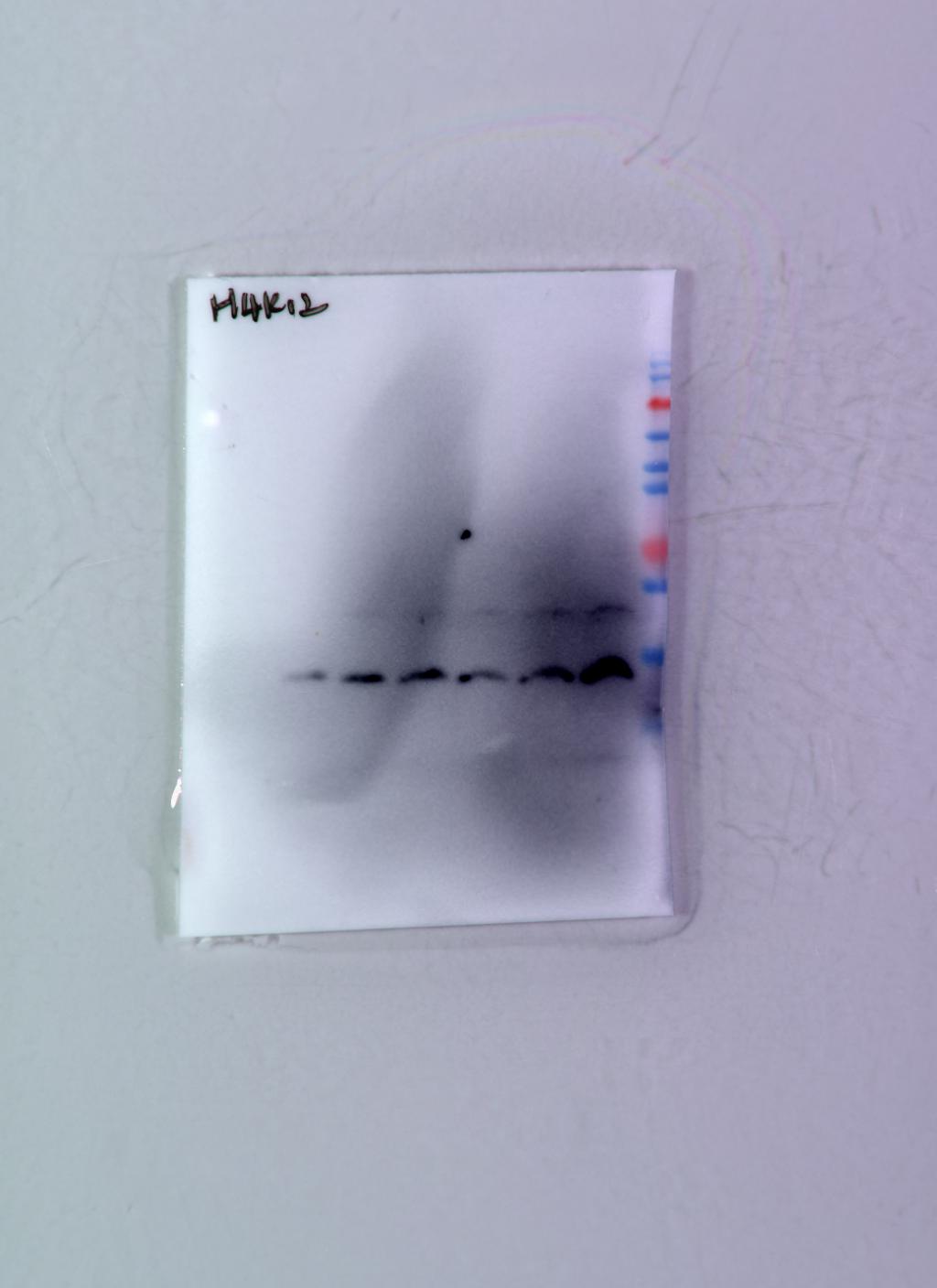

Supplement: Figure 1—source data 2. [file elife-110309-fig1-data2.zip › Figure 1-Source Data 8/H4K12 0-1 2023.12.10_12.30.36_Ch+Marker.jpg]

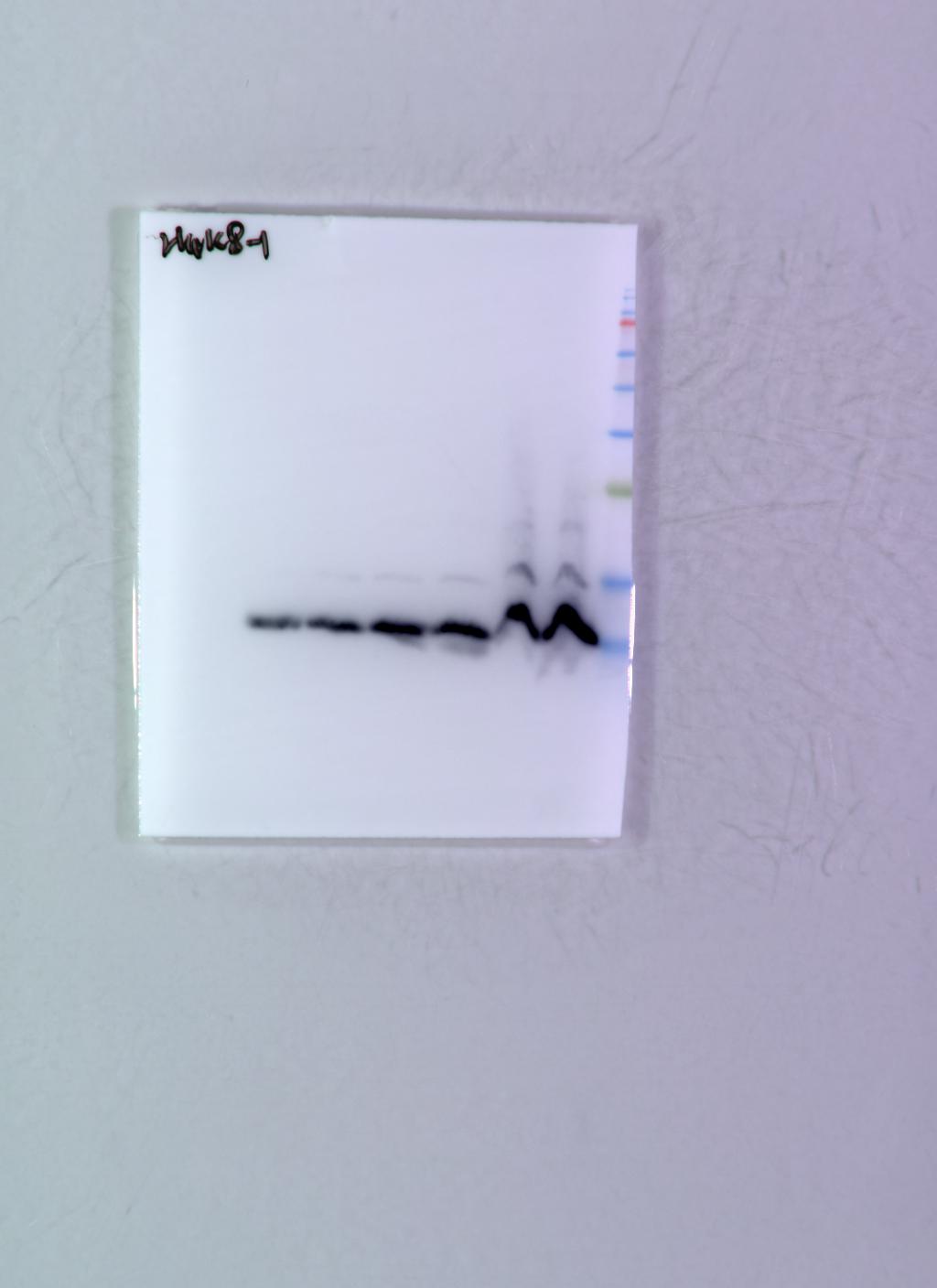

Supplement: Figure 1—source data 2. [file elife-110309-fig1-data2.zip › Figure 1-Source Data 8/H4K8 3-3 2023.11.26_11.37.06_Ch+Marker.jpg]

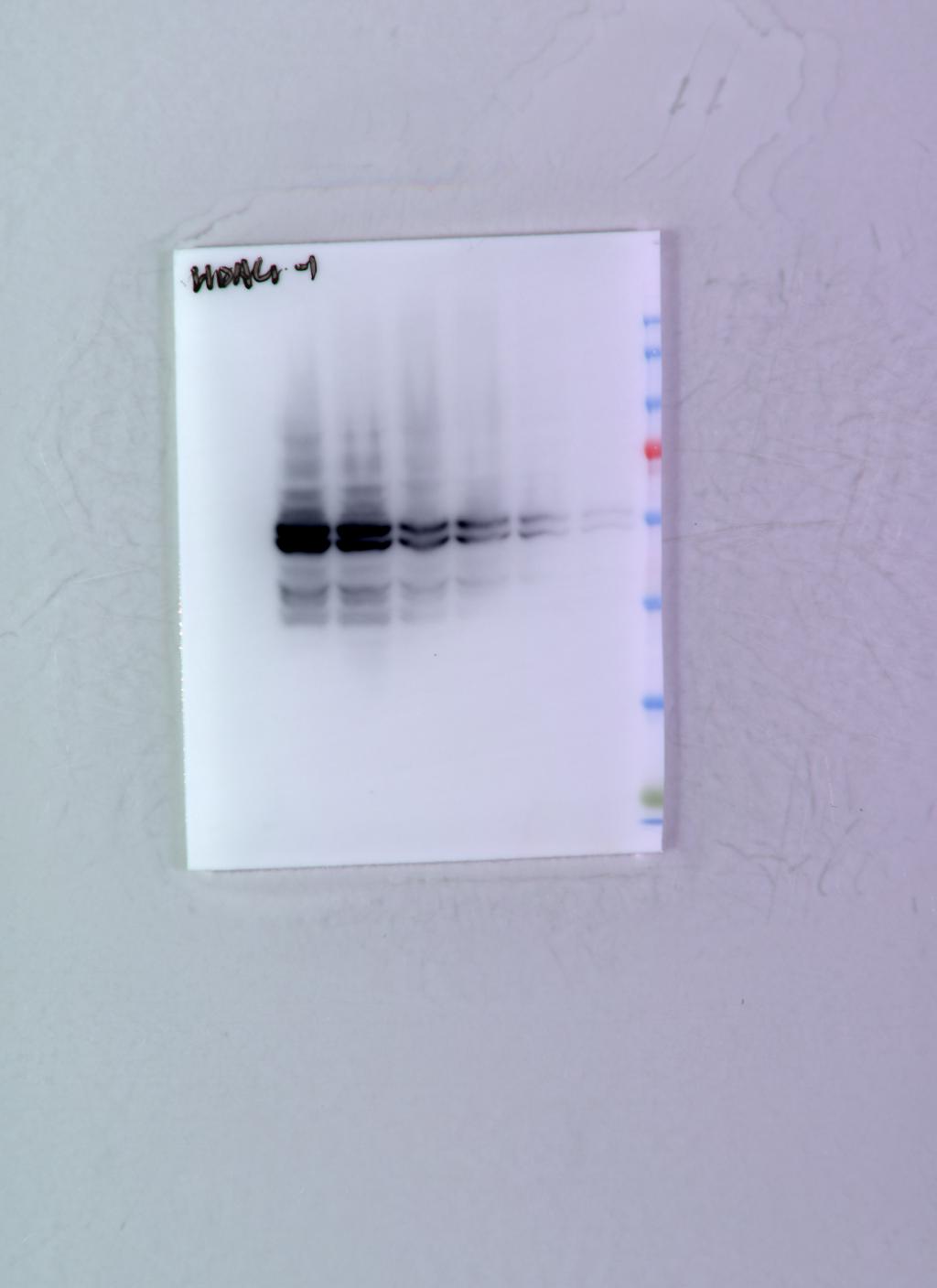

Supplement: Figure 1—source data 2. [file elife-110309-fig1-data2.zip › Figure 1-Source Data 8/HDAC1 3-1 2023.11.26_11.25.29_Ch+Marker.jpg]

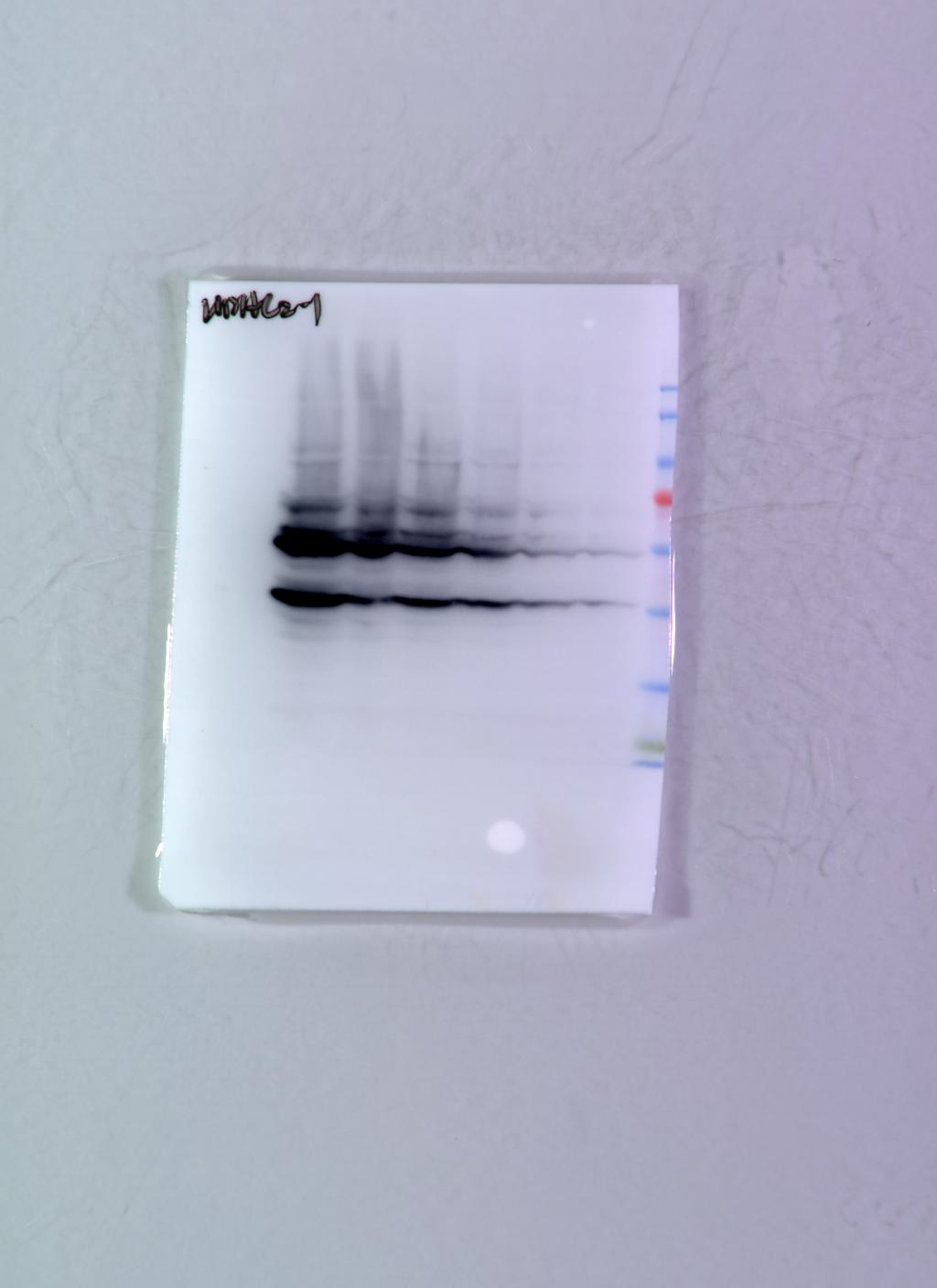

Supplement: Figure 1—source data 2. [file elife-110309-fig1-data2.zip › Figure 1-Source Data 8/HDAC2 3-3 2023.11.26_11.09.46_Ch+Marker.jpg]

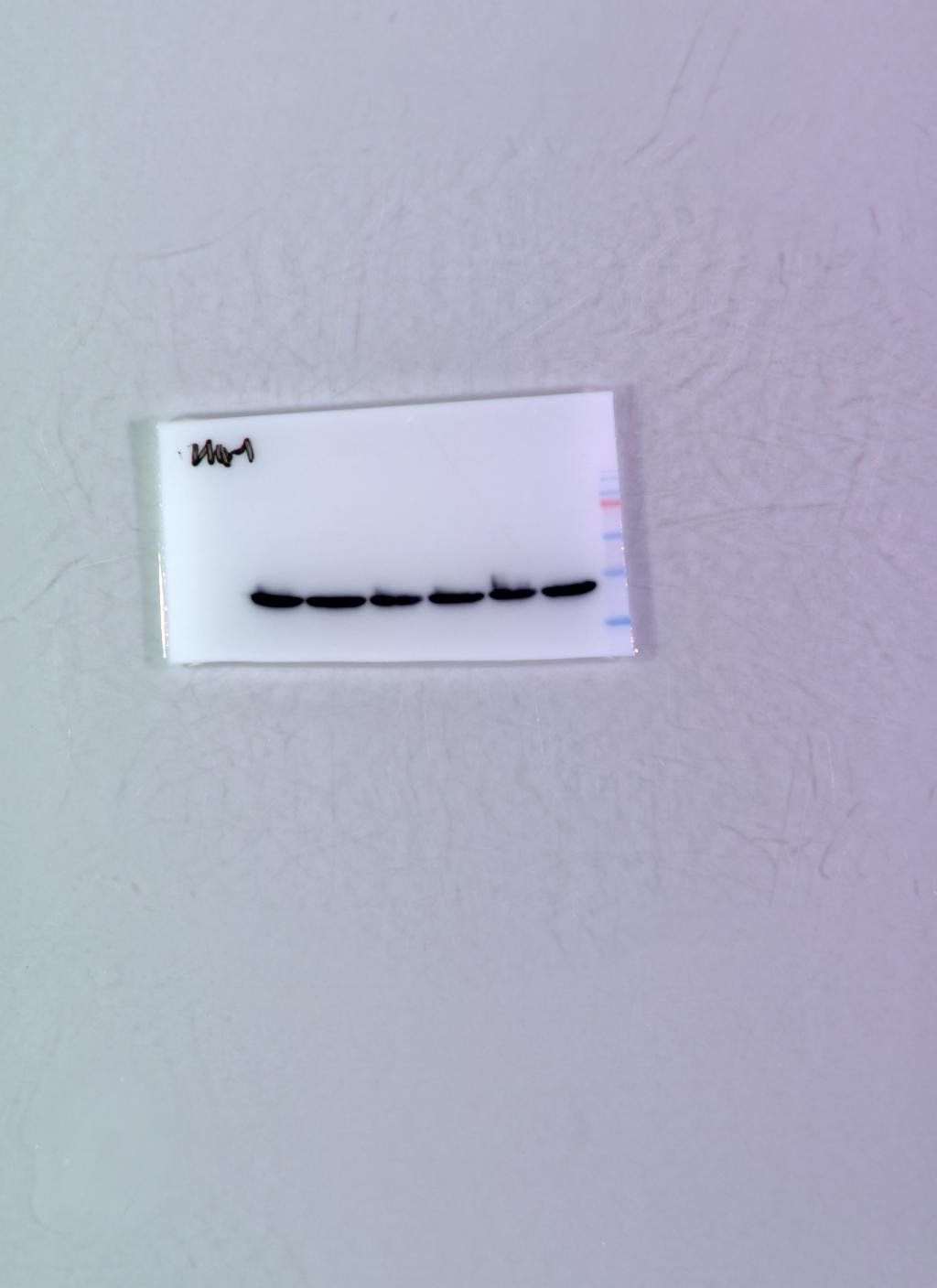

Supplement: Figure 1—source data 2. [file elife-110309-fig1-data2.zip › Figure 1-Source Data 10/ACTIN 4-1 2023.11.26_11.57.26_Ch+Marker.jpg]

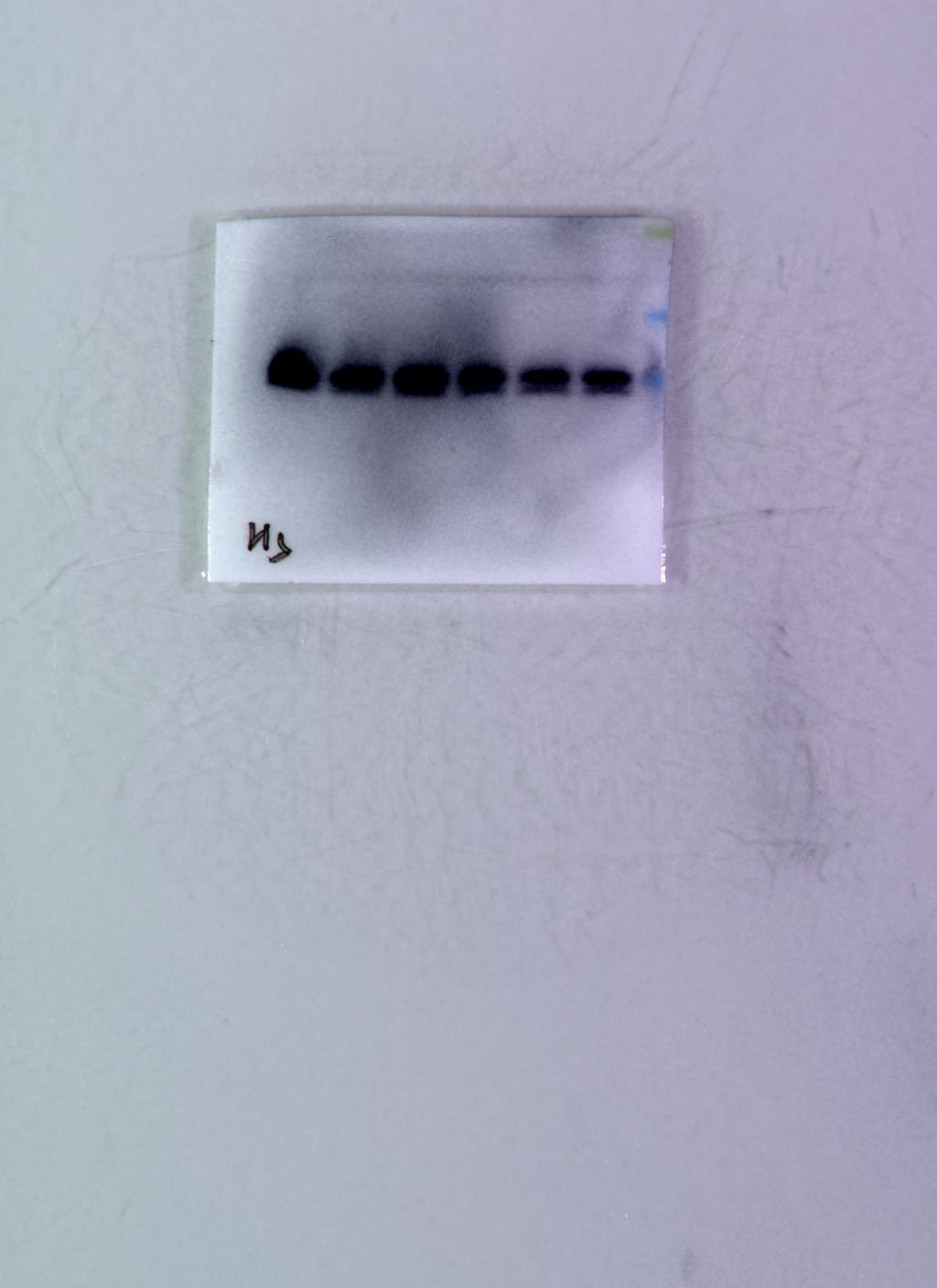

Supplement: Figure 1—source data 2. [file elife-110309-fig1-data2.zip › Figure 1-Source Data 10/H3 0-2 2023.11.25_22.07.03_Ch+Marker.jpg]

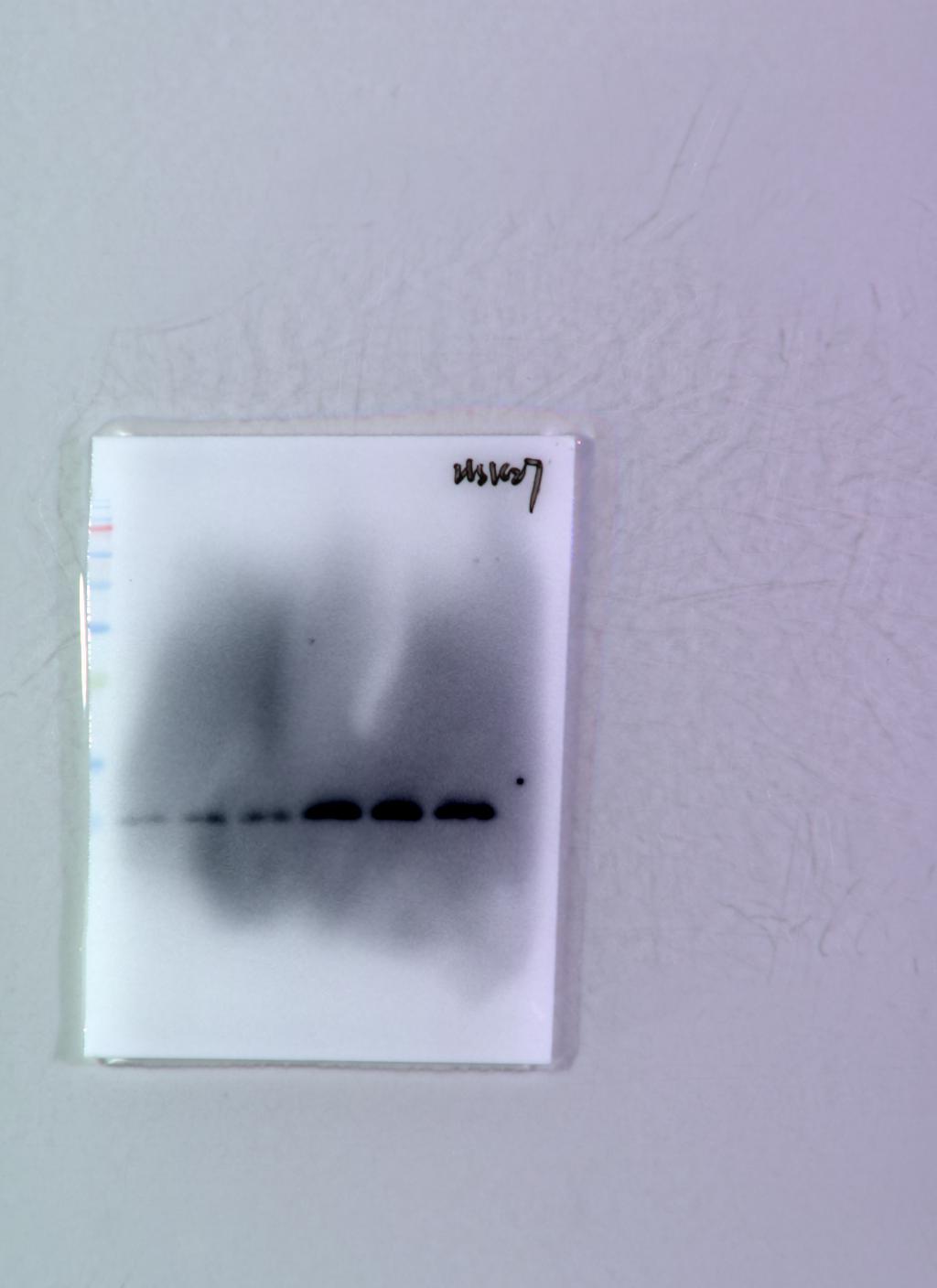

Supplement: Figure 1—source data 2. [file elife-110309-fig1-data2.zip › Figure 1-Source Data 10/H3K27 5-0 2023.12.02_13.31.28_Ch+Marker.jpg]

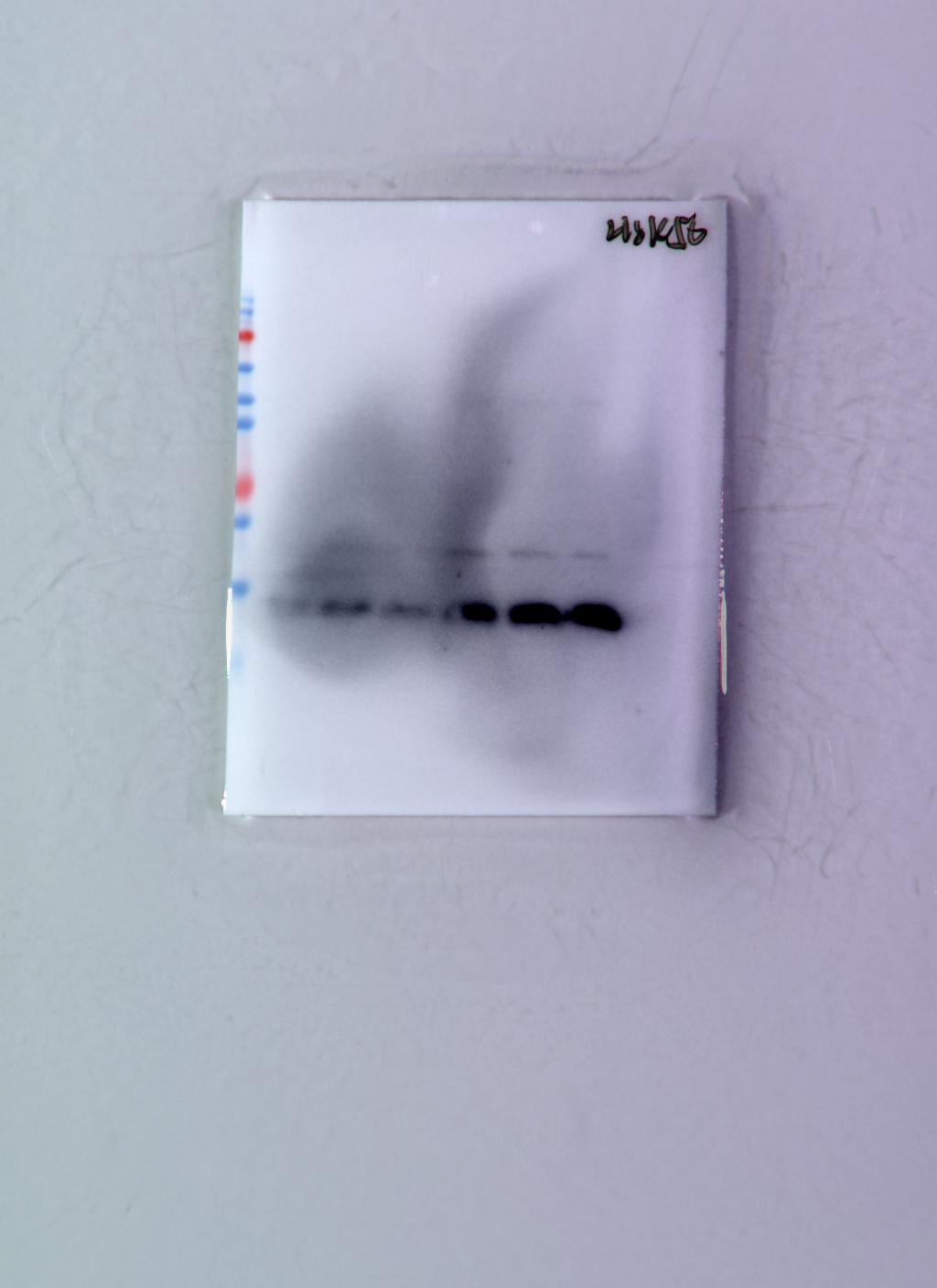

Supplement: Figure 1—source data 2. [file elife-110309-fig1-data2.zip › Figure 1-Source Data 10/H3K56 0-5 2023.12.10_12.05.13_Ch+Marker.jpg]

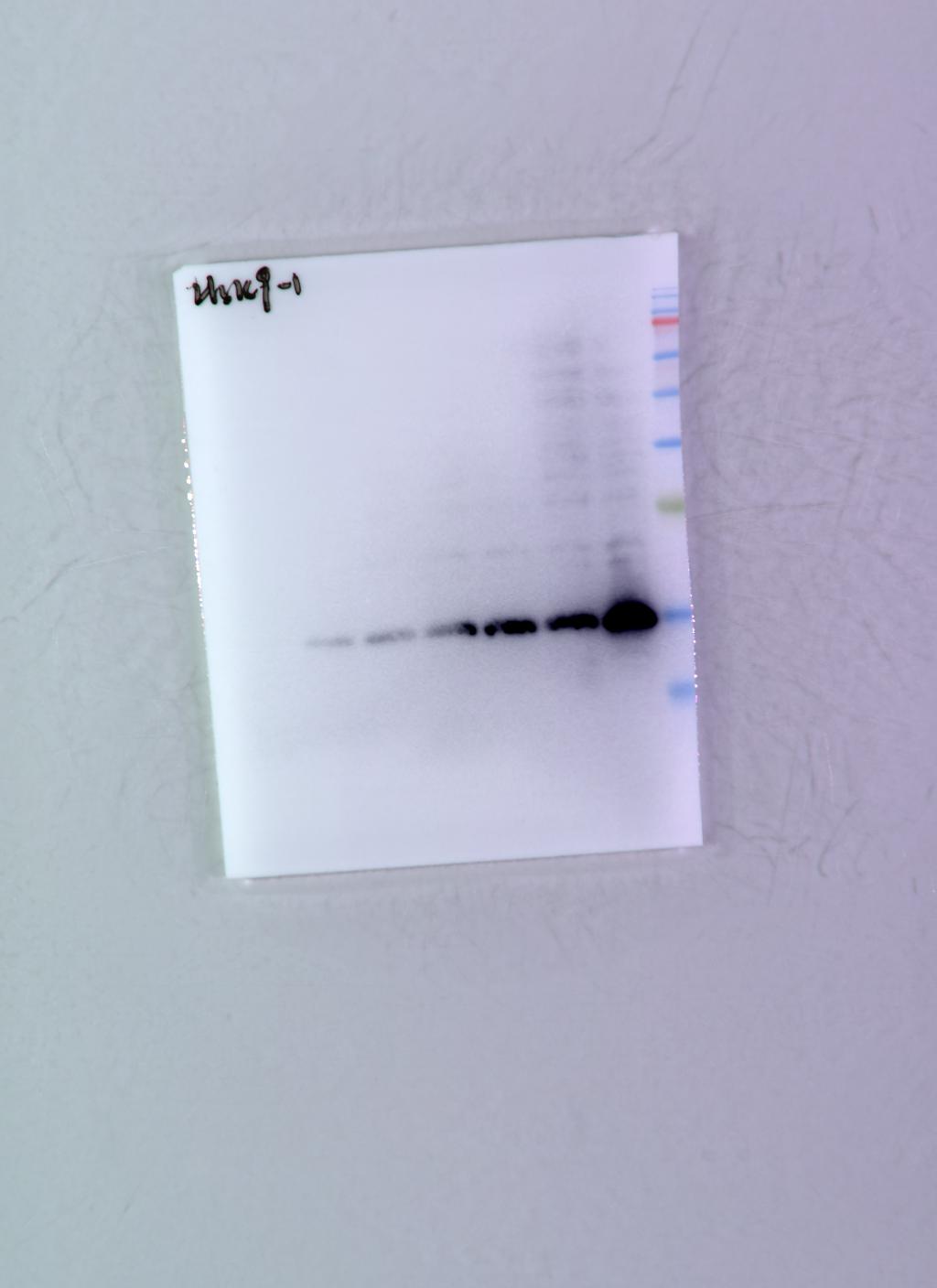

Supplement: Figure 1—source data 2. [file elife-110309-fig1-data2.zip › Figure 1-Source Data 10/H3K9 1-4 2023.11.26_10.44.23_Ch+Marker.jpg]

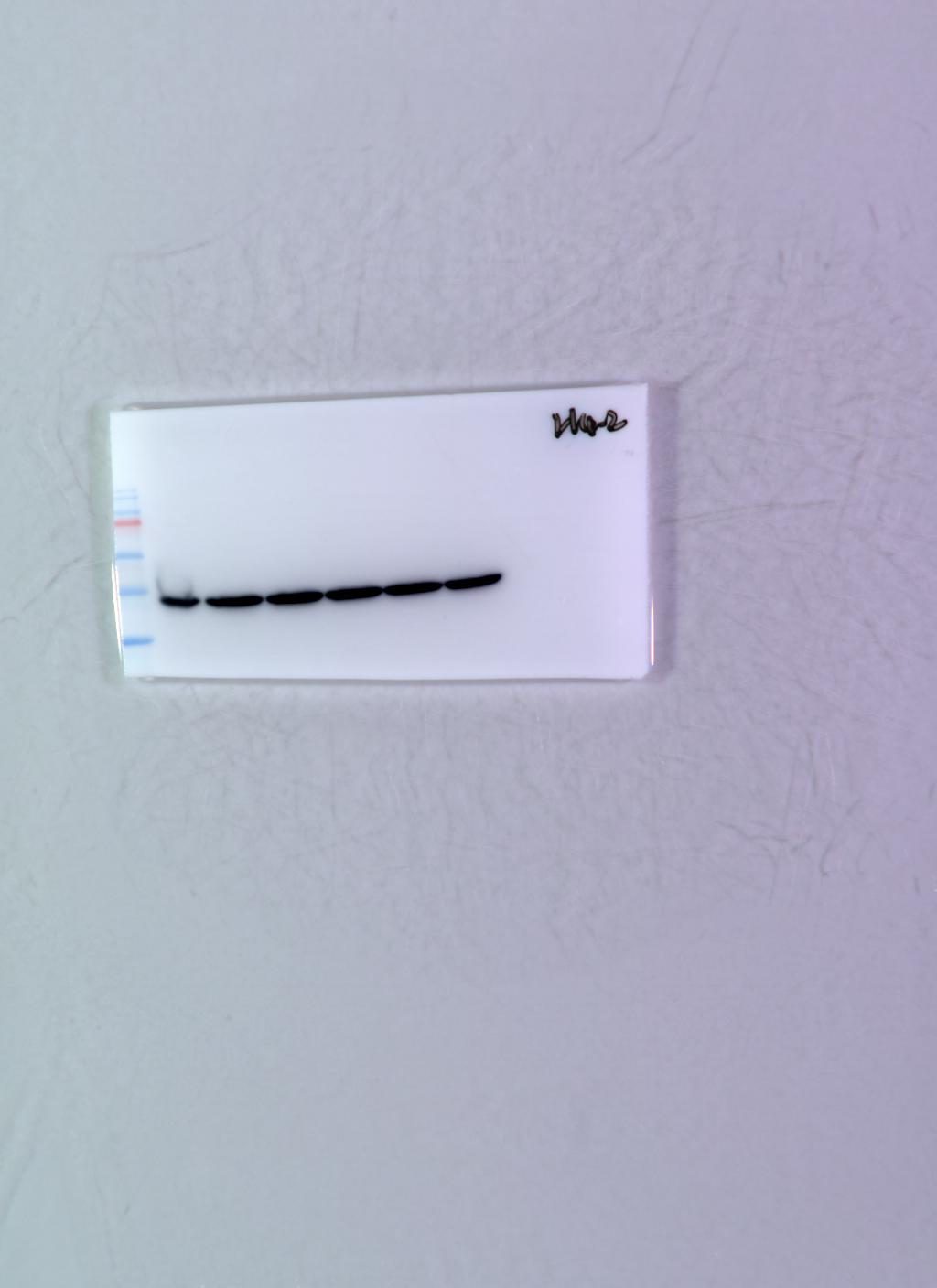

Supplement: Figure 1—source data 2. [file elife-110309-fig1-data2.zip › Figure 1-Source Data 10/H4 5-0 2023.11.26_11.59.51_Ch+Marker.jpg]

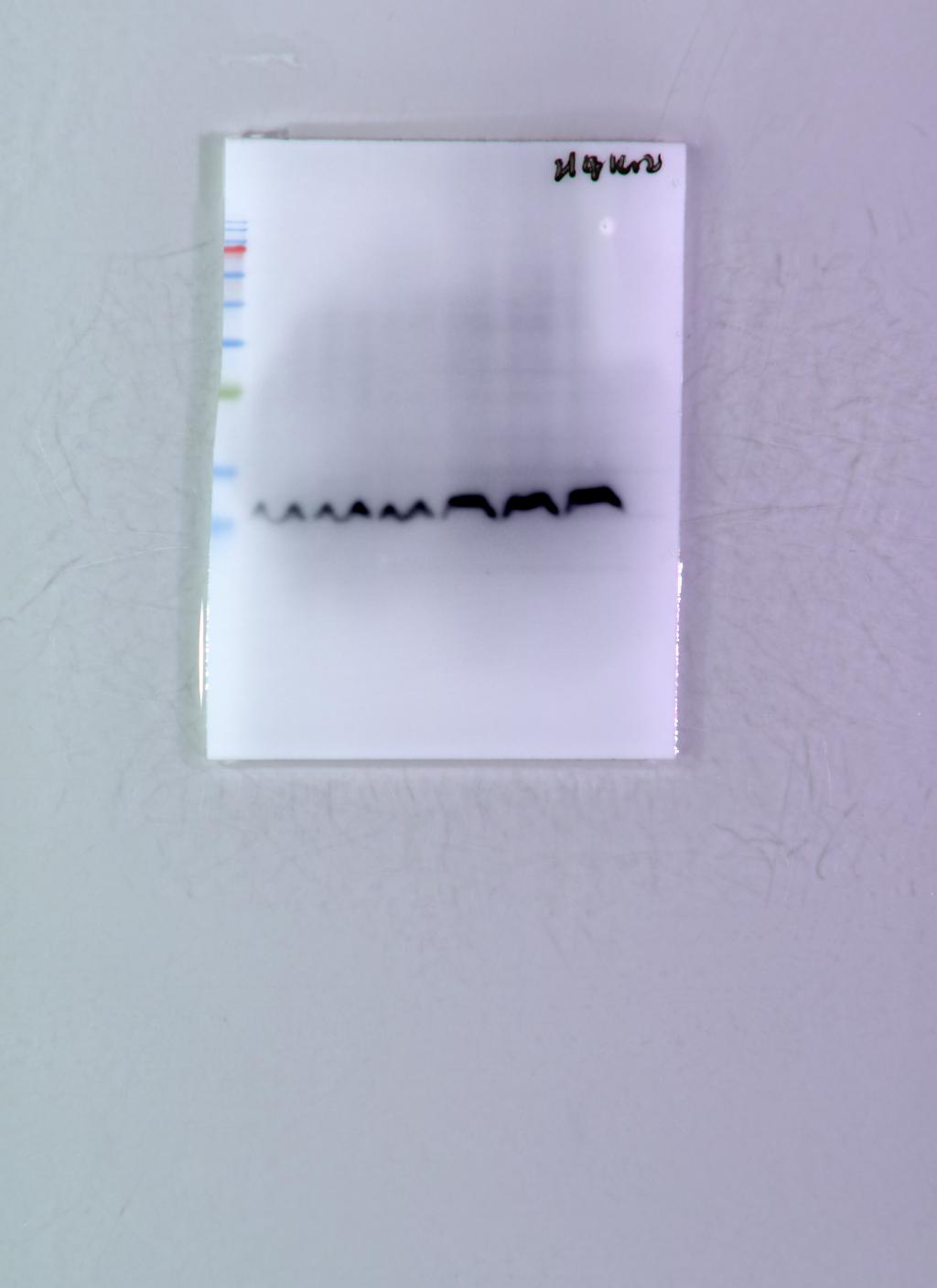

Supplement: Figure 1—source data 2. [file elife-110309-fig1-data2.zip › Figure 1-Source Data 10/H4K12 M 0-2 2023.11.26_00.17.10_Ch+Marker.jpg]

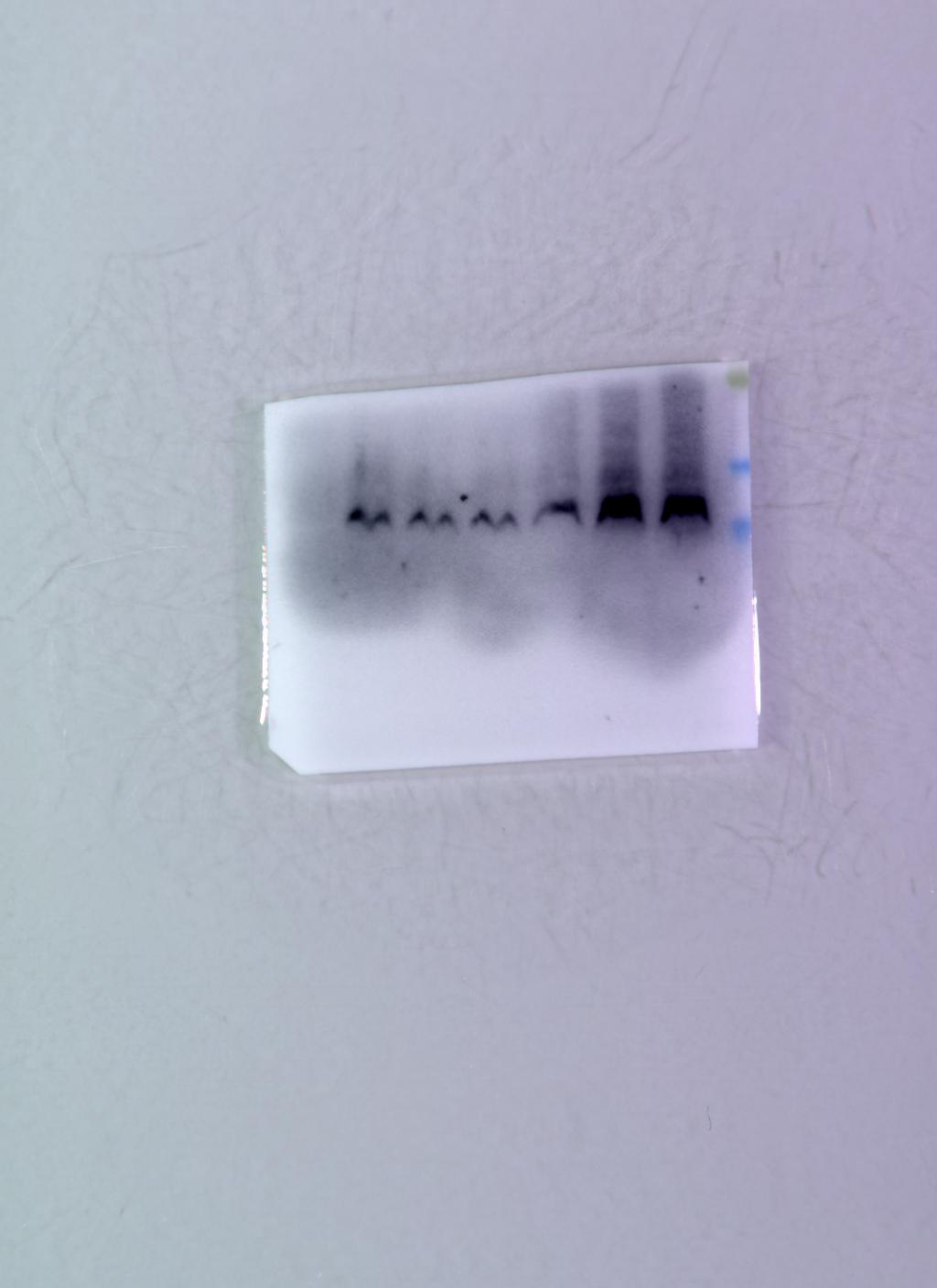

Supplement: Figure 1—source data 2. [file elife-110309-fig1-data2.zip › Figure 1-Source Data 10/H4K8 M 0-2 2023.11.25_22.25.53_Ch+Marker.jpg]

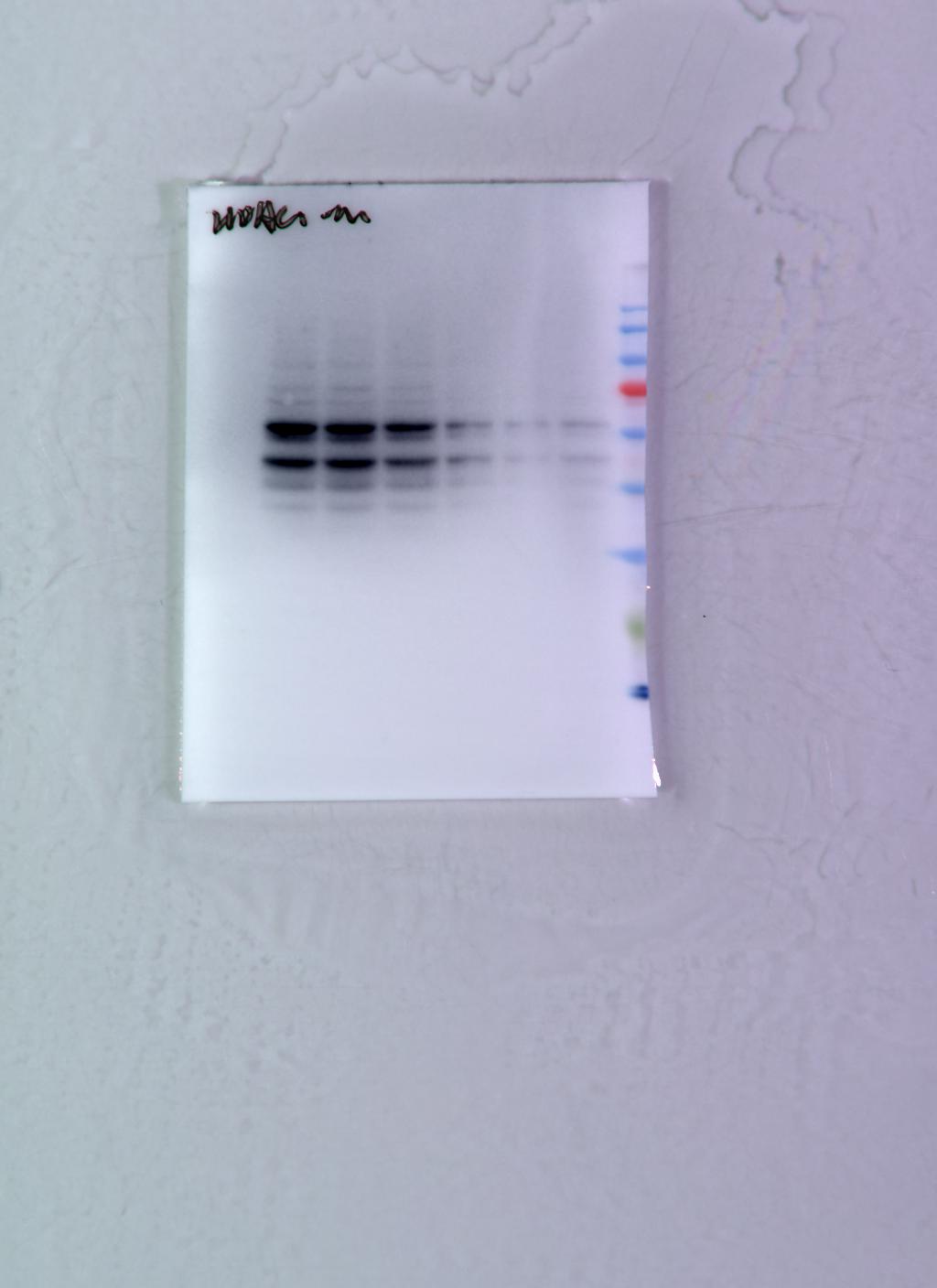

Supplement: Figure 1—source data 2. [file elife-110309-fig1-data2.zip › Figure 1-Source Data 10/hdac1 m 0-4 2023.11.25_20.45.10_Ch+Marker.jpg]

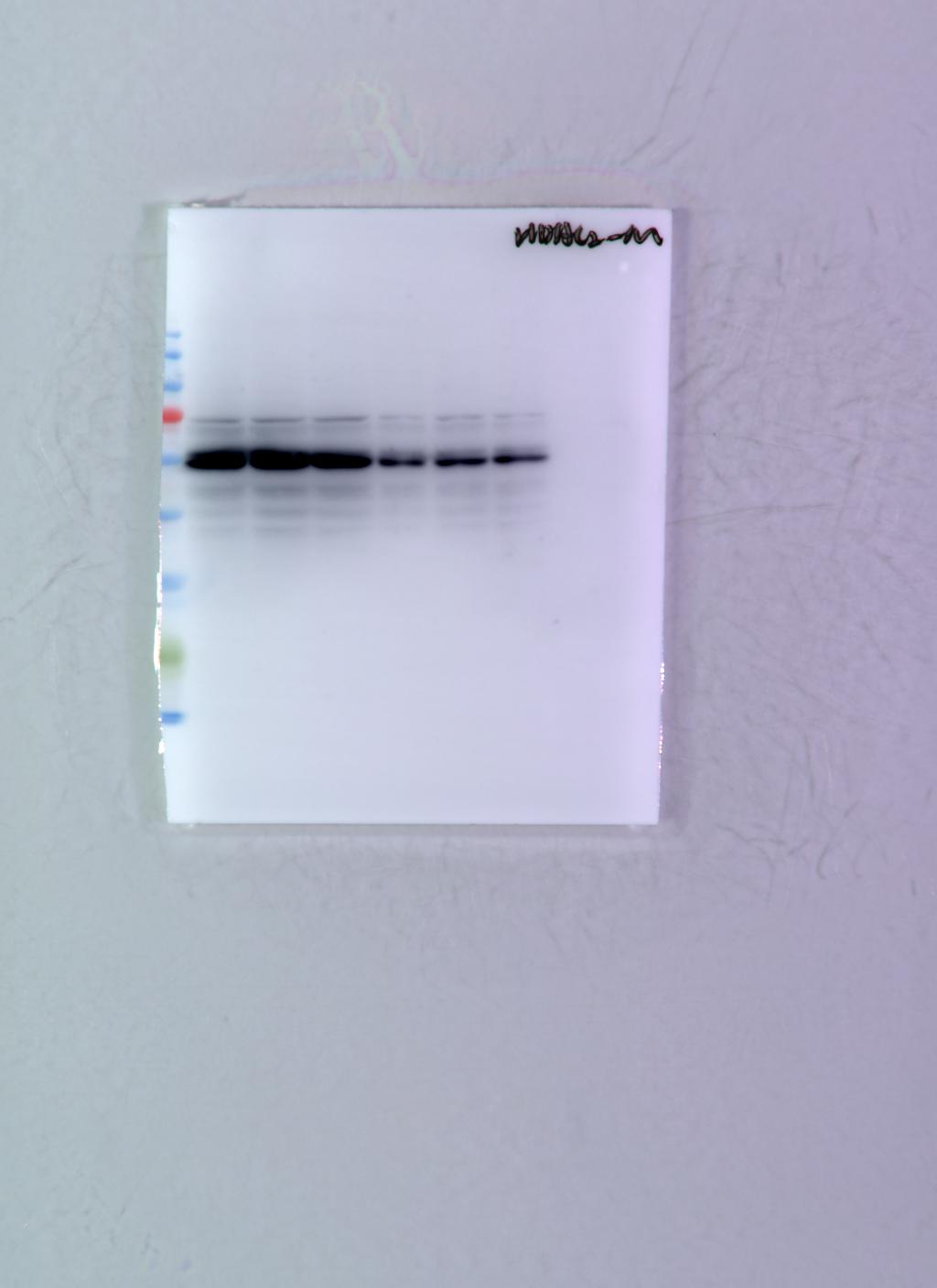

Supplement: Figure 1—source data 2. [file elife-110309-fig1-data2.zip › Figure 1-Source Data 10/HDAC2 M 1-0 2023.11.25_22.59.30_Ch+Marker.jpg]

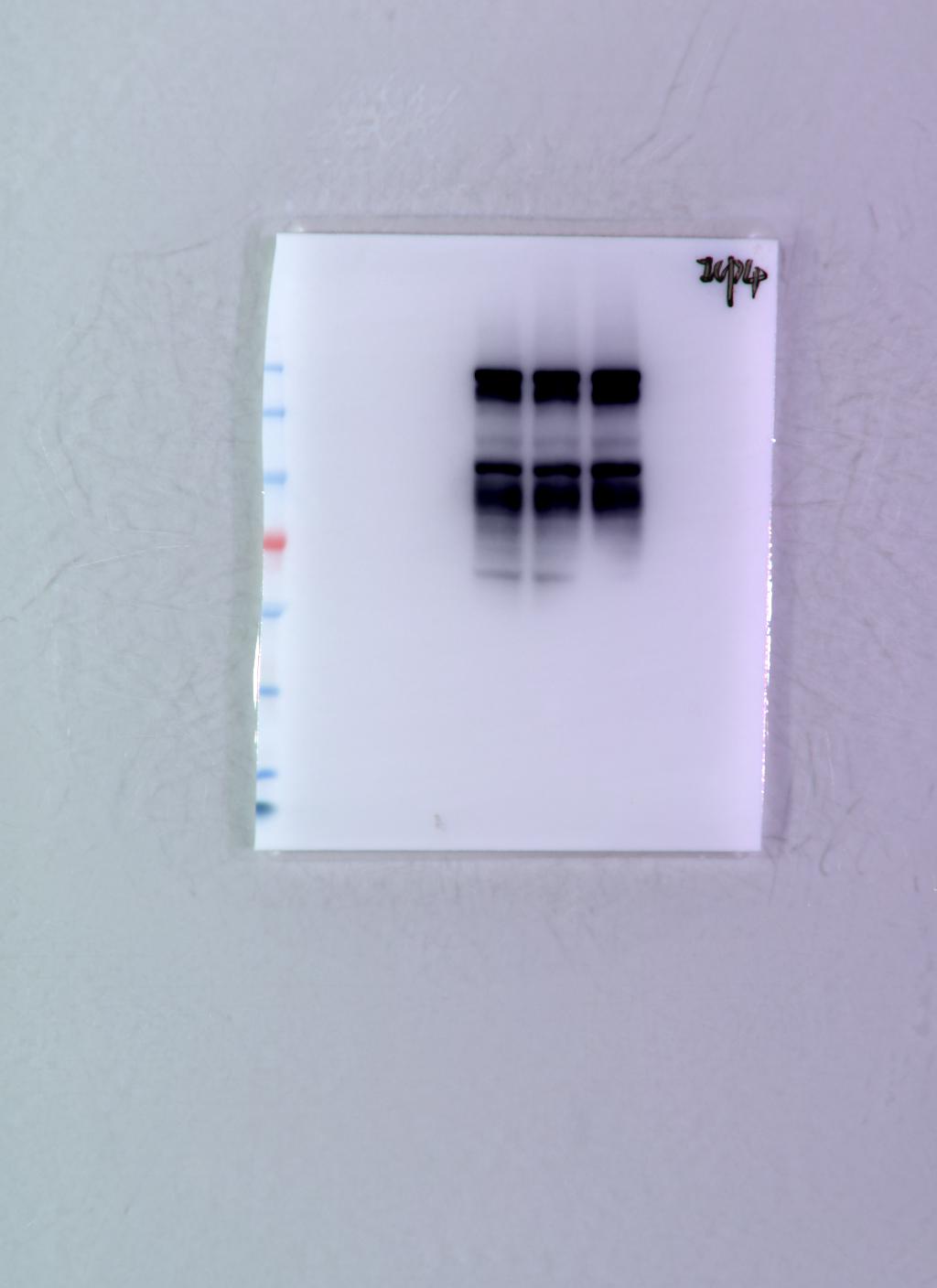

Supplement: Figure 1—source data 2. [file elife-110309-fig1-data2.zip › Figure 1-Source Data 10/ICP4 M 1-1 2023.11.25_23.15.19_Ch+Marker.jpg]

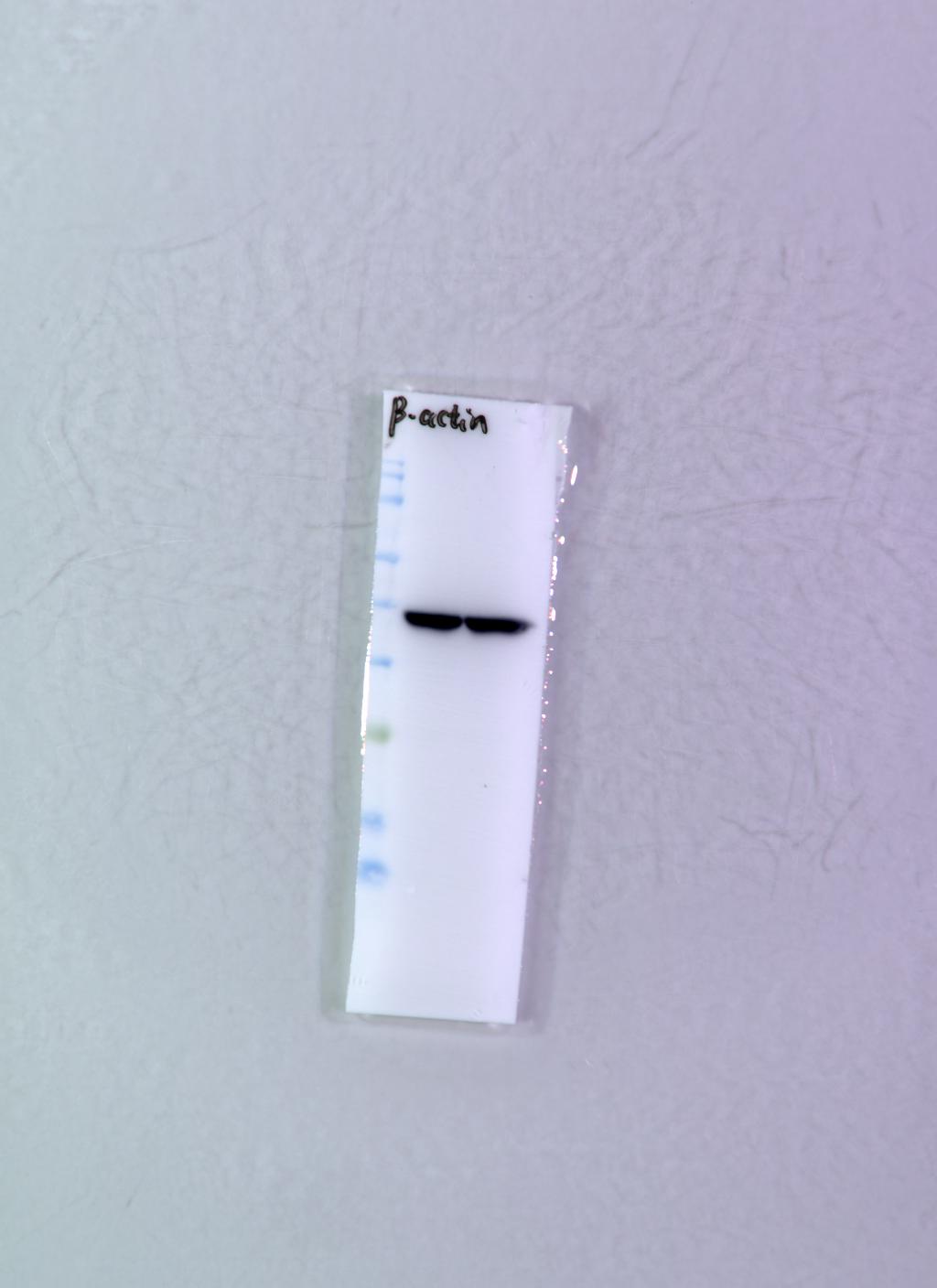

Supplement: Figure 1—source data 2. [file elife-110309-fig1-data2.zip › Figure 1-Source Data 12/ACTIN 0-3 2026.04.07_09.39.15_Ch+Marker.jpg]

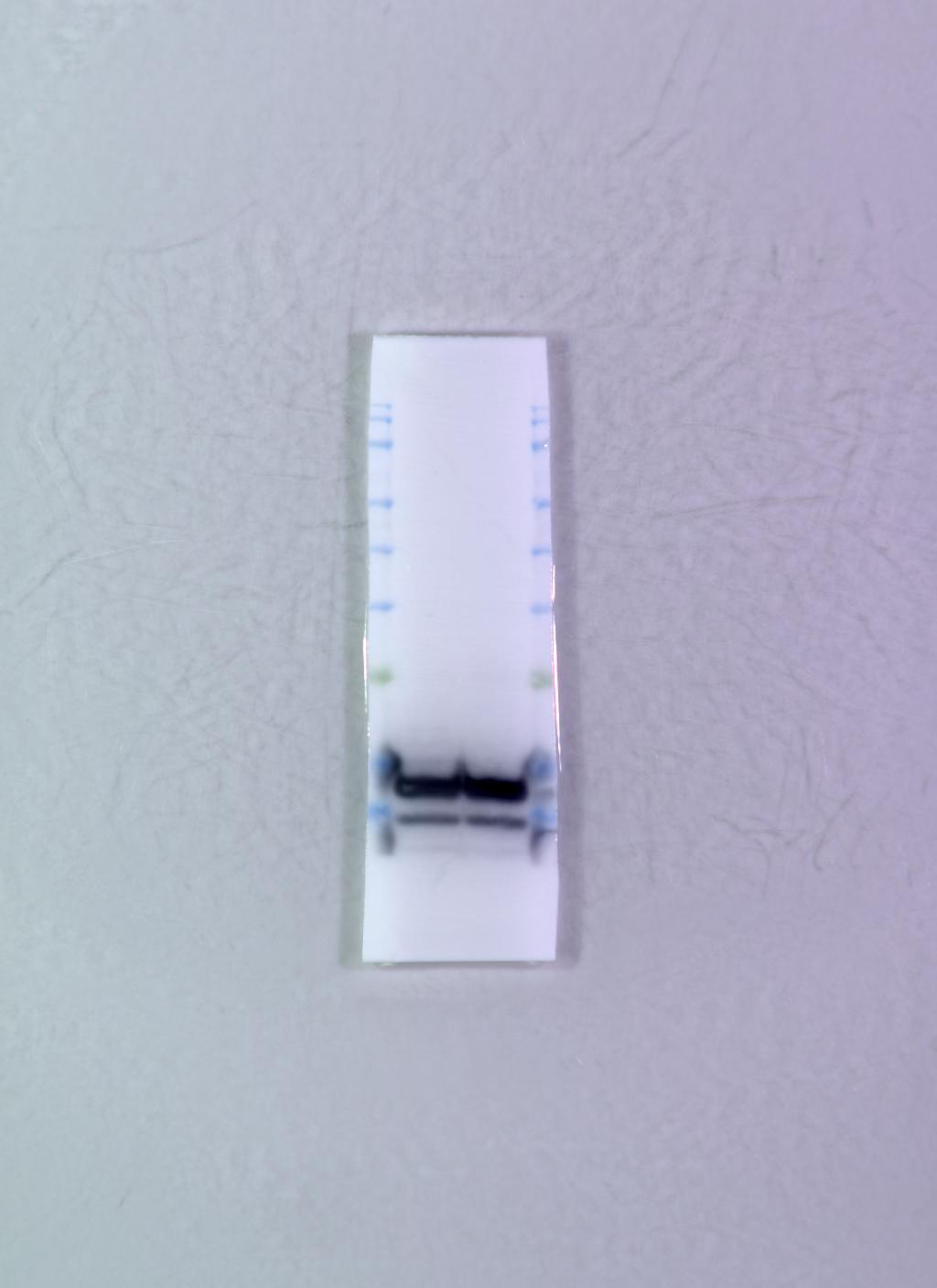

Supplement: Figure 1—source data 2. [file elife-110309-fig1-data2.zip › Figure 1-Source Data 12/H3 0-3 2026.04.07_10.17.11_Ch+Marker.jpg]

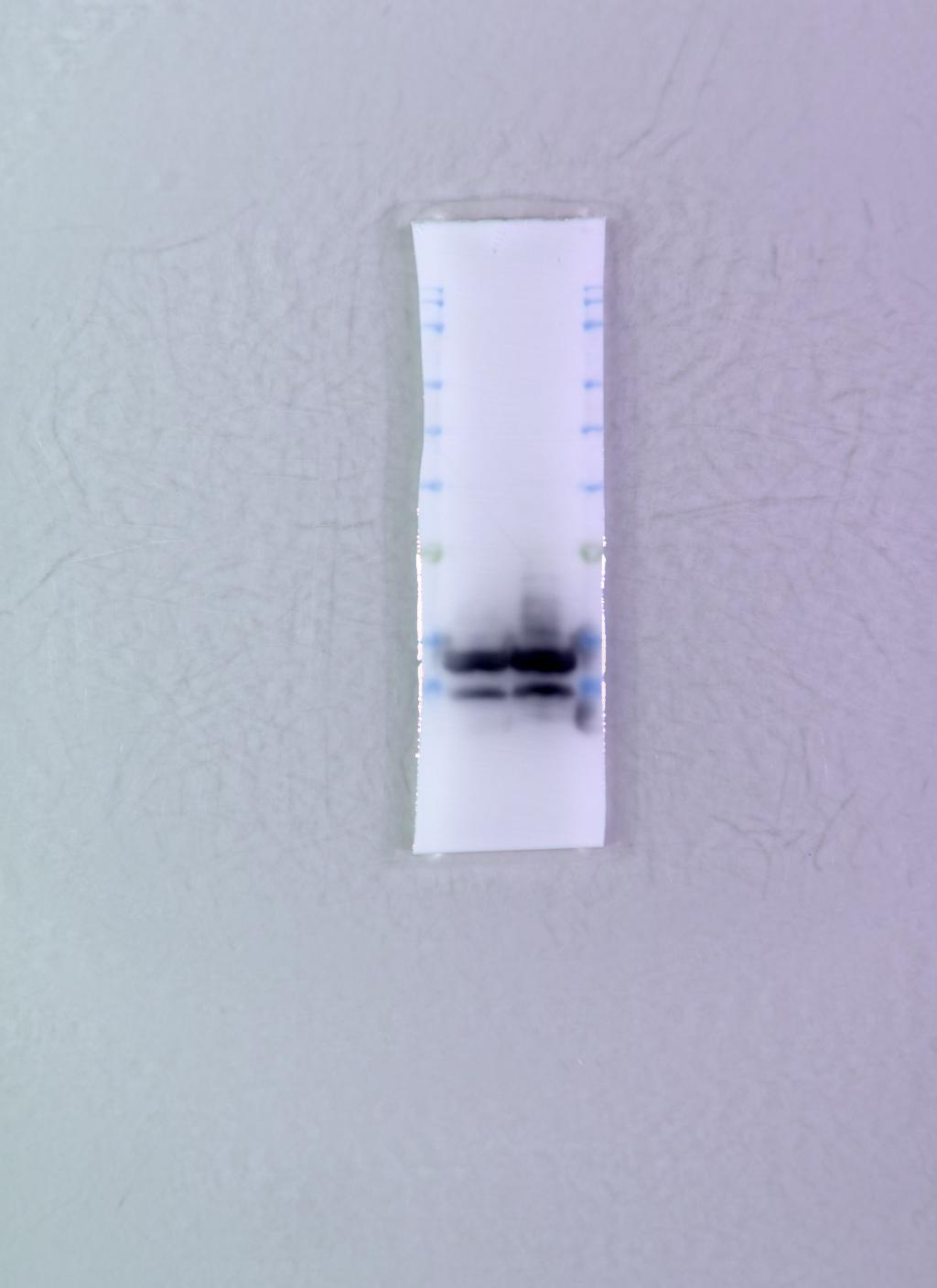

Supplement: Figure 1—source data 2. [file elife-110309-fig1-data2.zip › Figure 1-Source Data 12/H3K27 0-6 2026.04.07_10.14.25_Ch+Marker.jpg]

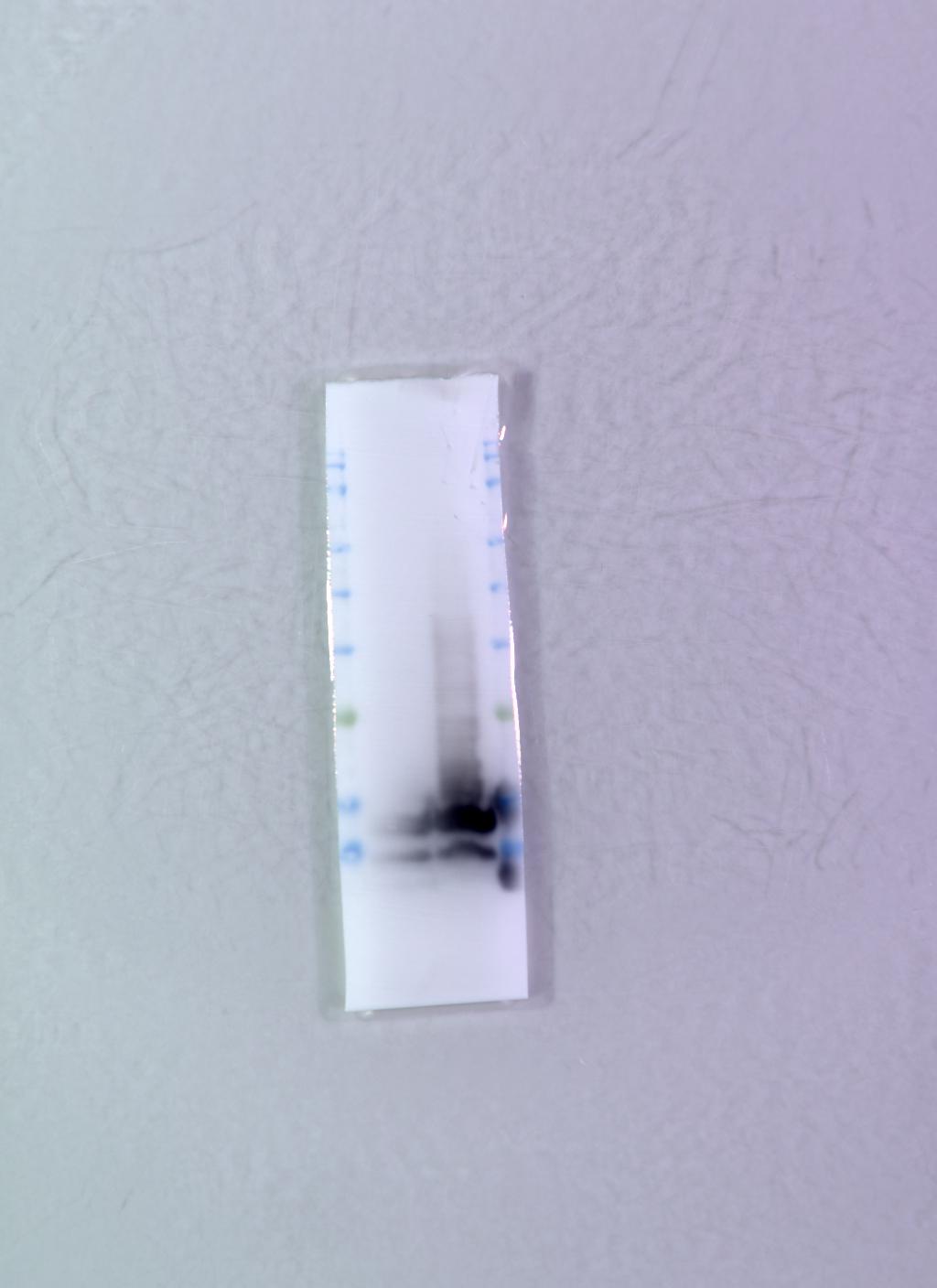

Supplement: Figure 1—source data 2. [file elife-110309-fig1-data2.zip › Figure 1-Source Data 12/H3K56 0-3 2026.04.07_10.22.09_Ch+Marker.jpg]

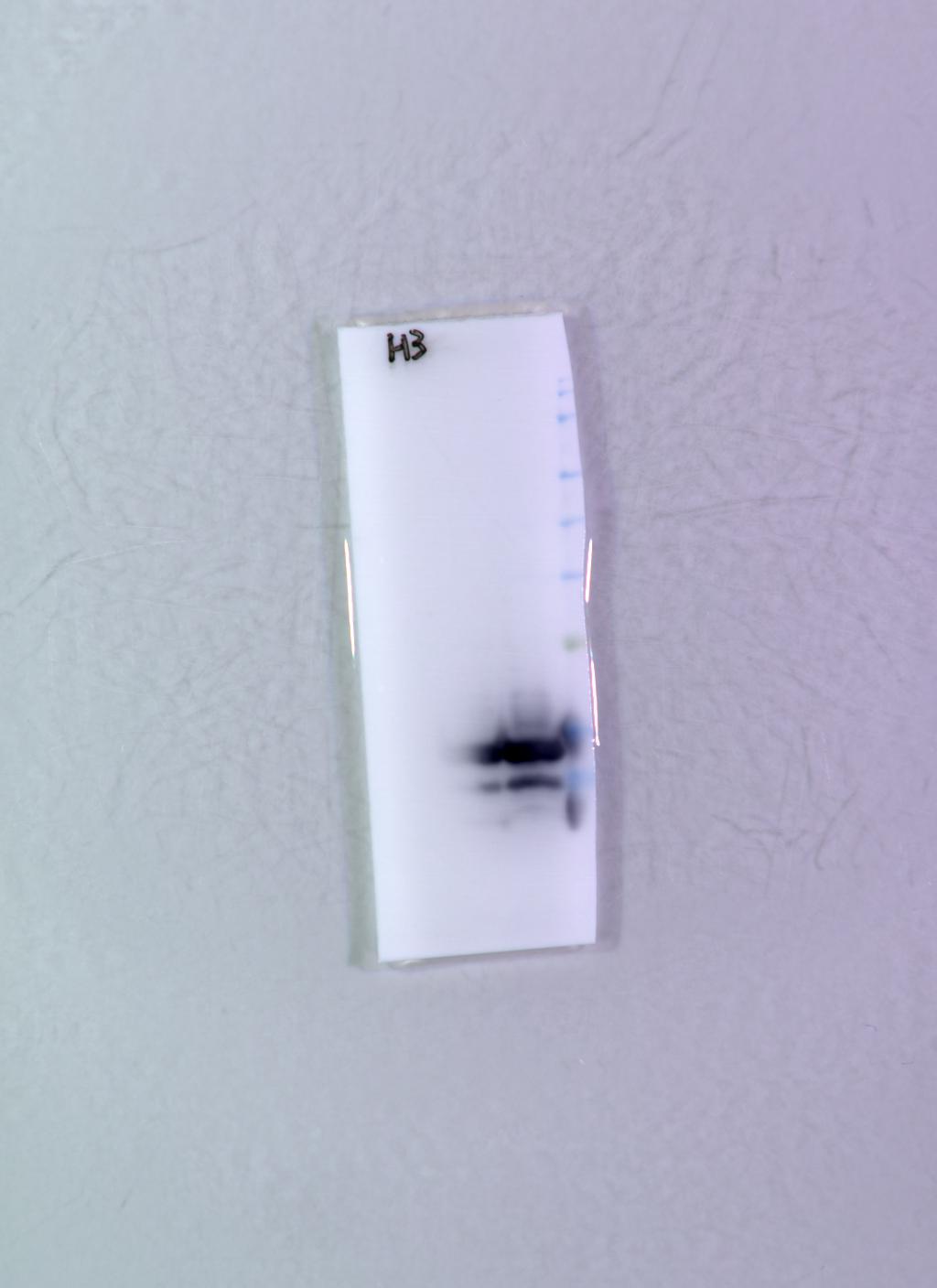

Supplement: Figure 1—source data 2. [file elife-110309-fig1-data2.zip › Figure 1-Source Data 12/H3K9 0-2 2026.04.07_10.08.06_Ch+Marker.jpg]

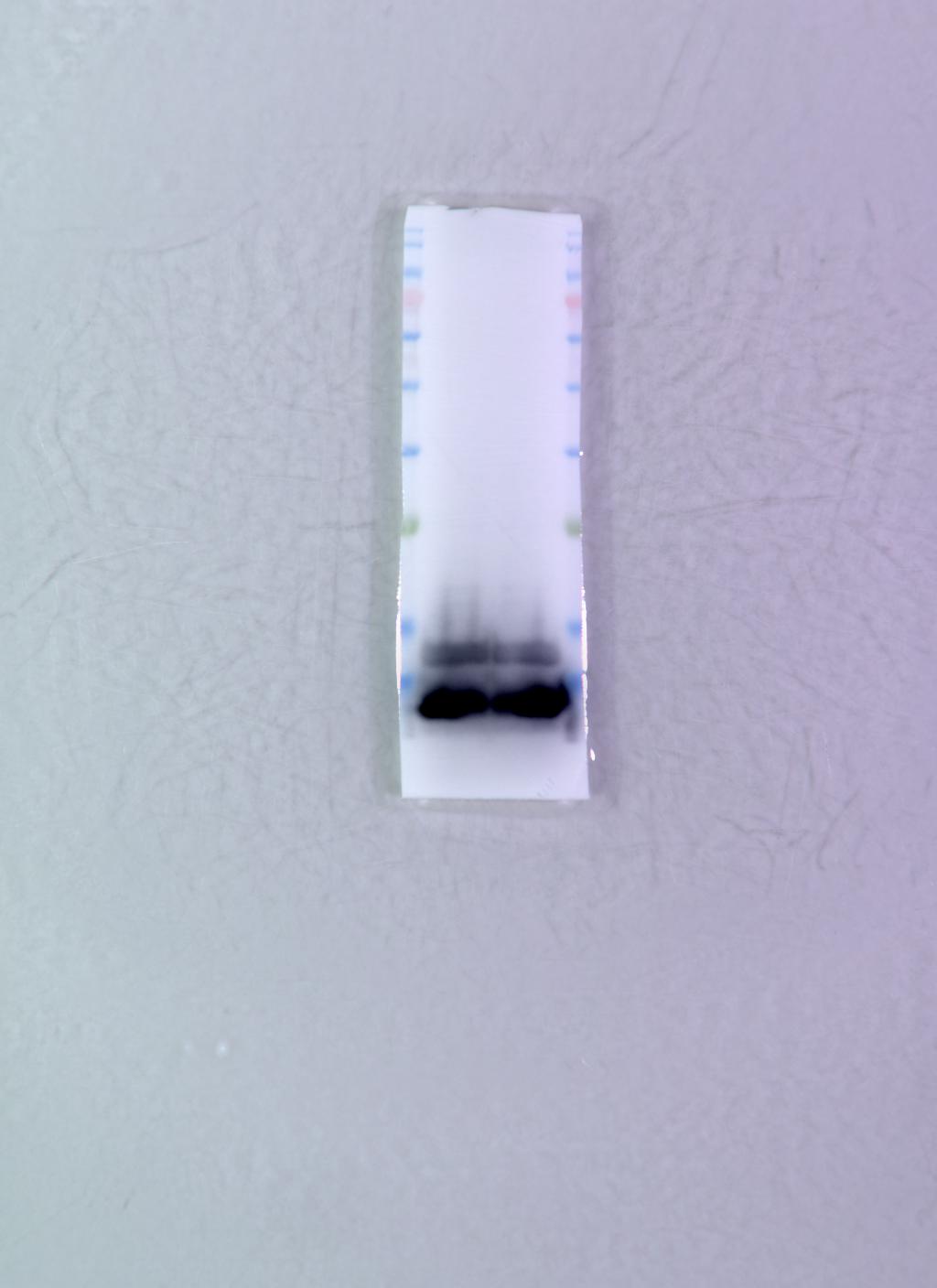

Supplement: Figure 1—source data 2. [file elife-110309-fig1-data2.zip › Figure 1-Source Data 12/H4 0-3 2026.04.07_09.59.40_Ch+Marker.jpg]

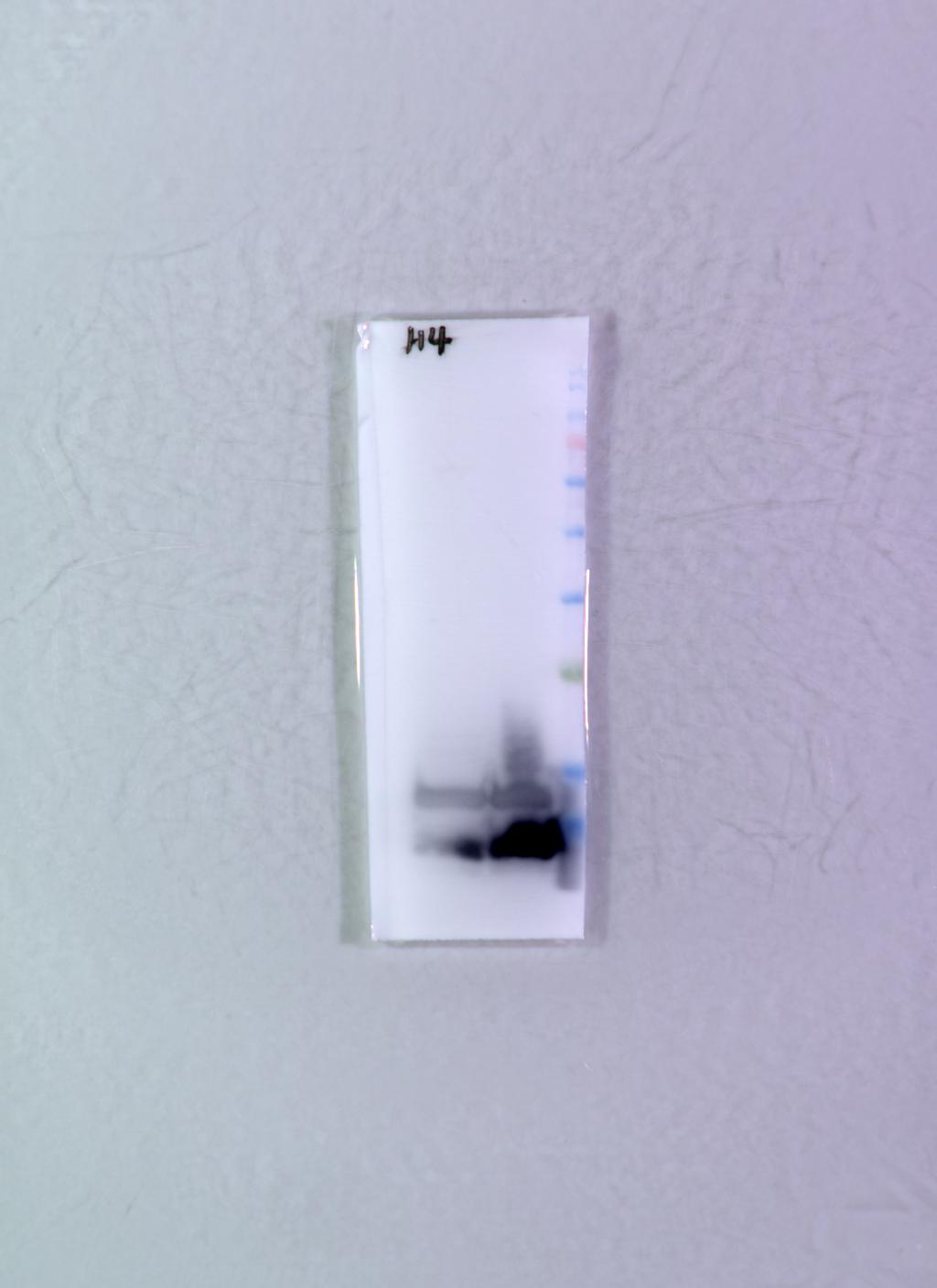

Supplement: Figure 1—source data 2. [file elife-110309-fig1-data2.zip › Figure 1-Source Data 12/H4K12 0-3 2026.04.07_10.02.13_Ch+Marker.jpg]

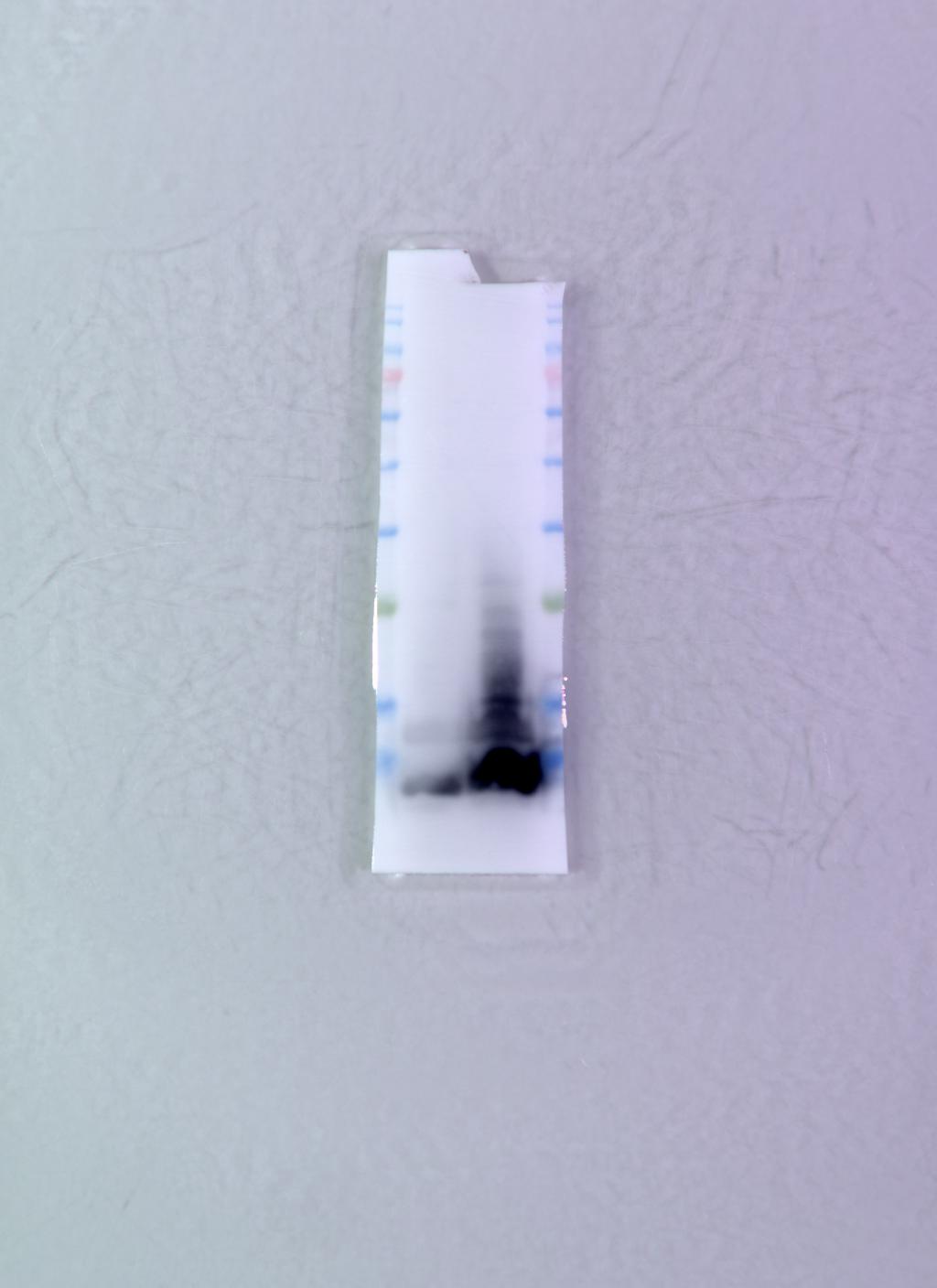

Supplement: Figure 1—source data 2. [file elife-110309-fig1-data2.zip › Figure 1-Source Data 12/H4K18 0-1 2026.04.07_09.55.04_Ch+Marker.jpg]

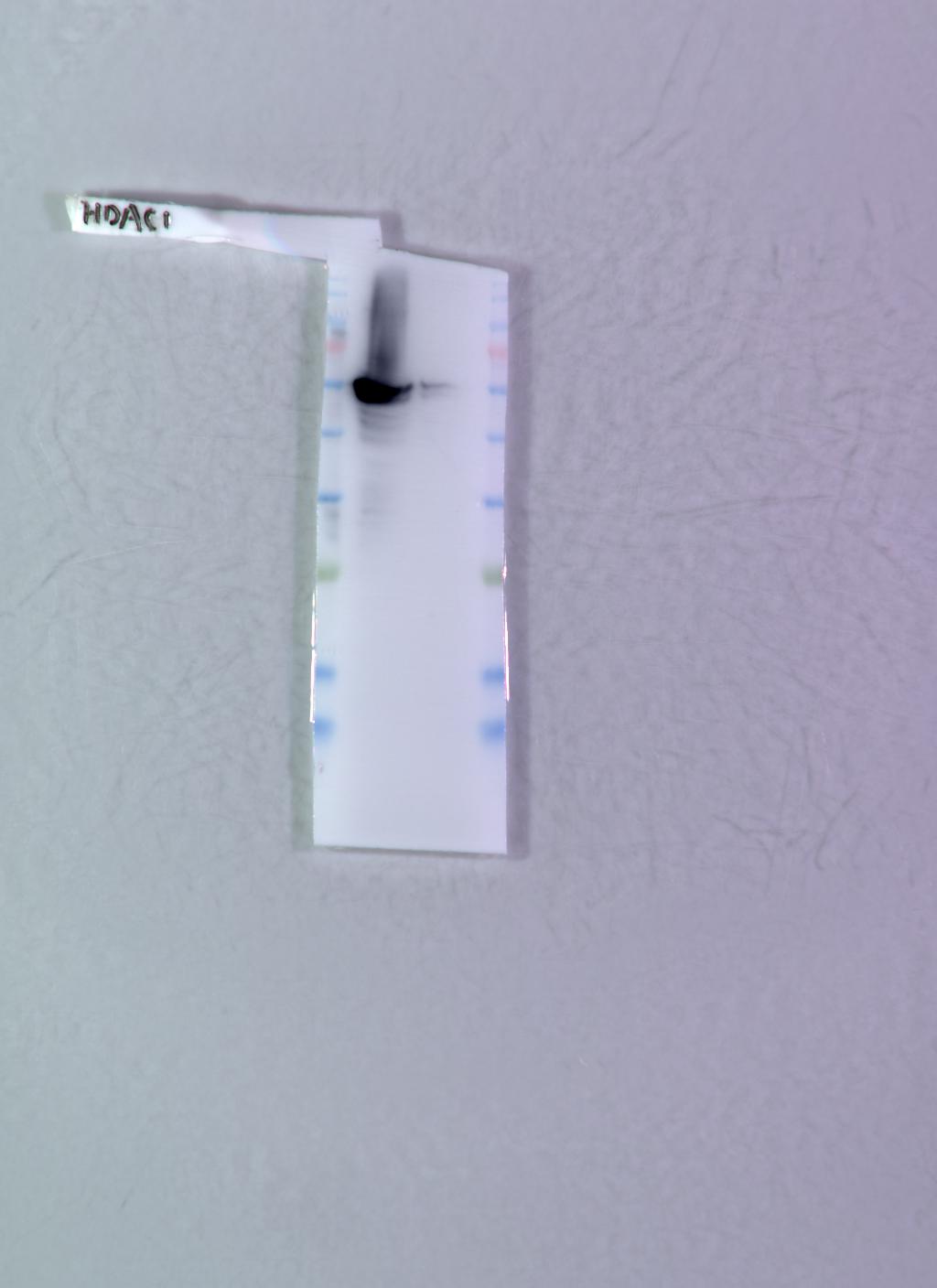

Supplement: Figure 1—source data 2. [file elife-110309-fig1-data2.zip › Figure 1-Source Data 12/HDAC1 0-2 2026.04.07_09.46.17_Ch+Marker.jpg]

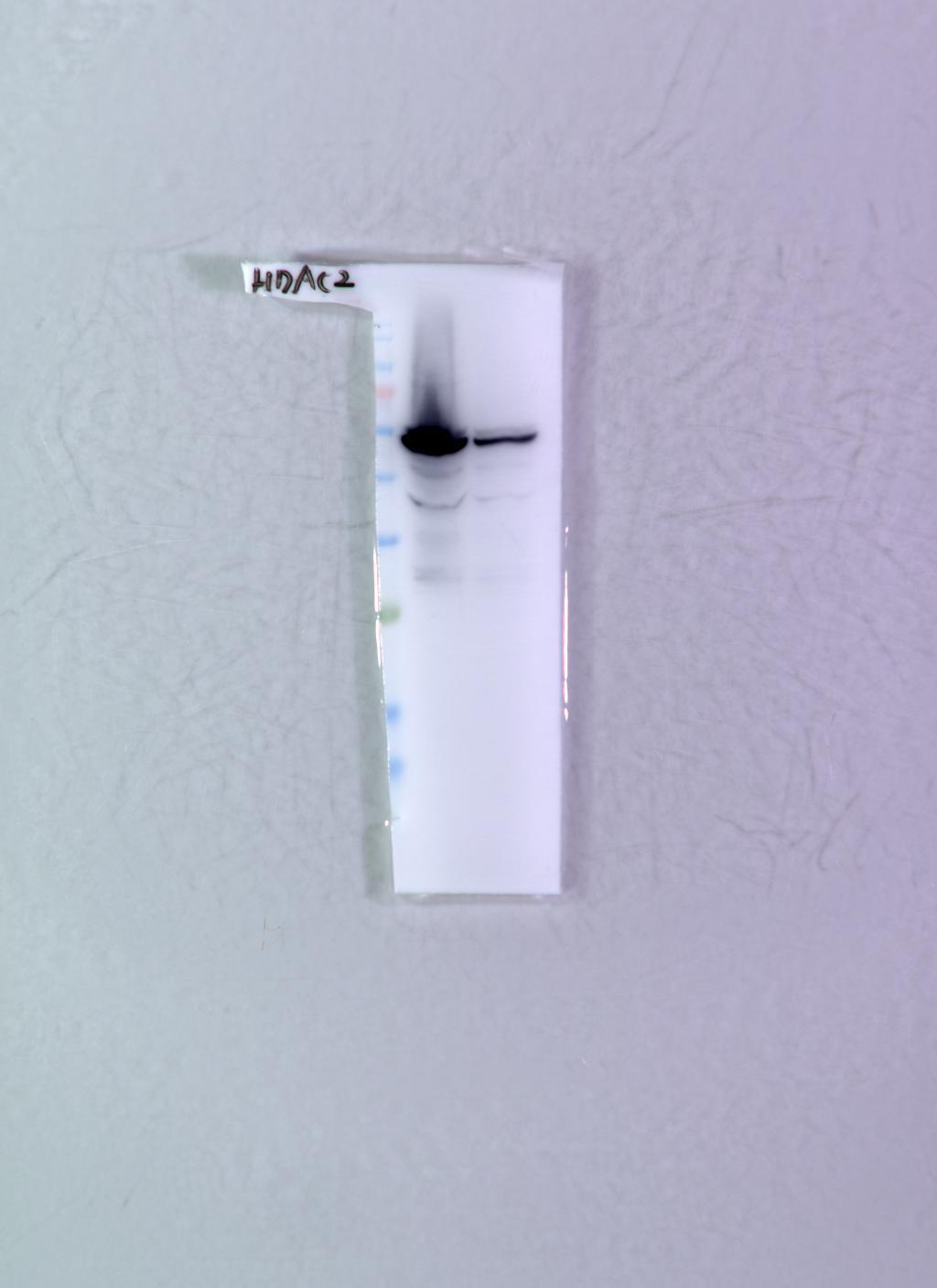

Supplement: Figure 1—source data 2. [file elife-110309-fig1-data2.zip › Figure 1-Source Data 12/HDAC2 0-3 2026.04.07_09.43.37_Ch+Marker.jpg]

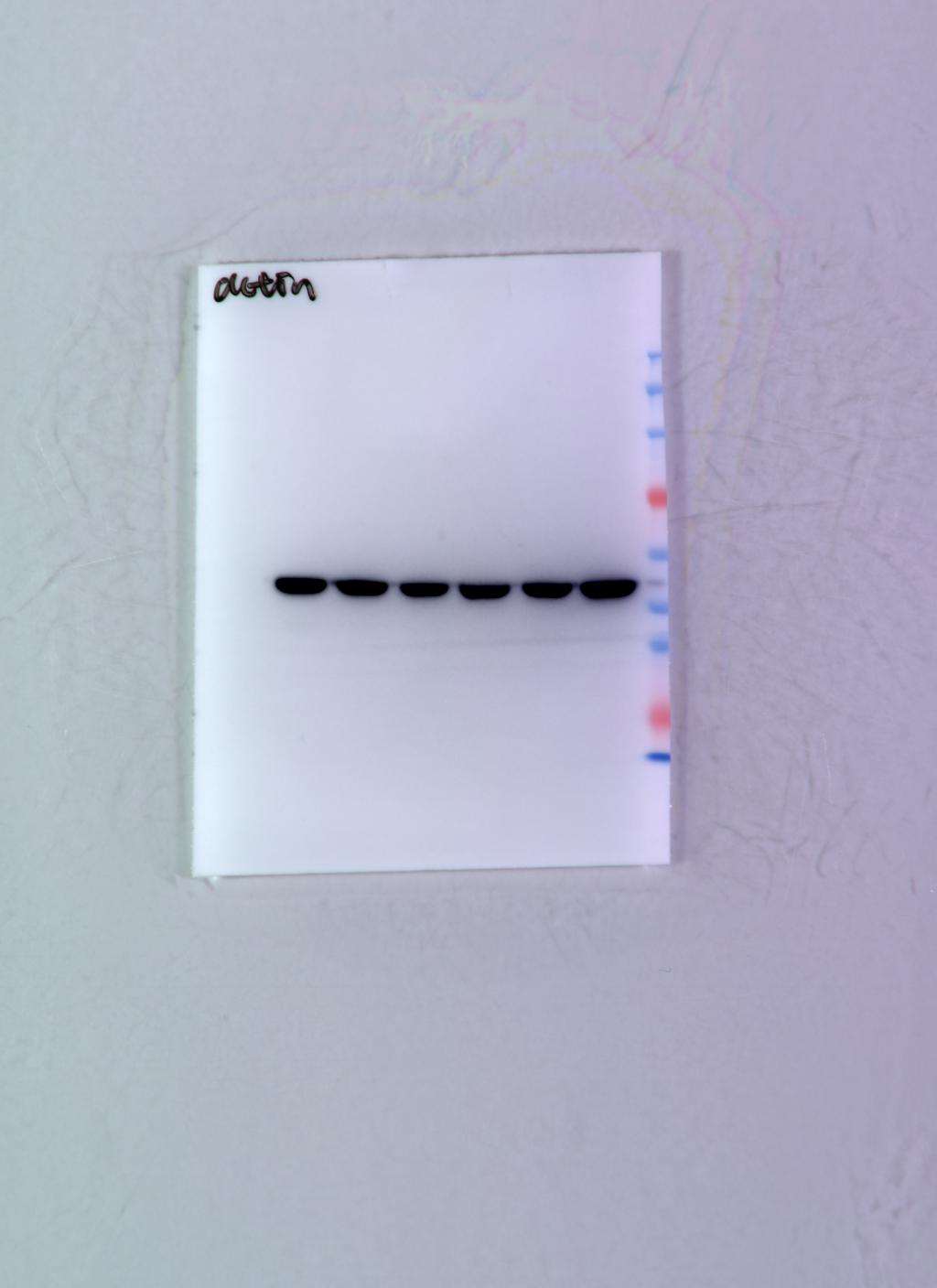

Supplement: Figure 2—source data 2. [file elife-110309-fig2-data2.zip › Figure 2-Source Data 2/ACTIN 1-2 2023.12.10_12.51.21_Ch+Marker.jpg]

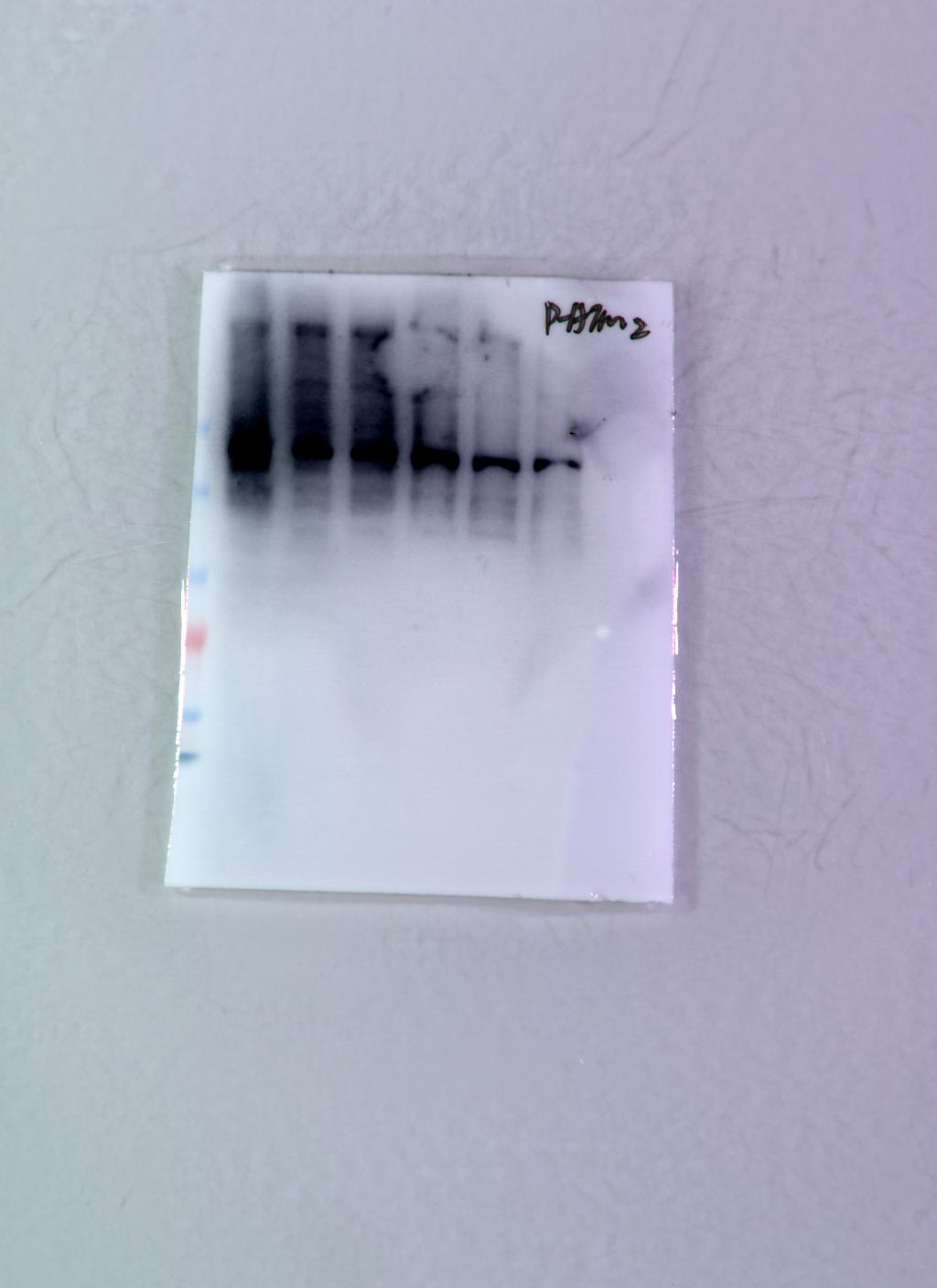

Supplement: Figure 2—source data 2. [file elife-110309-fig2-data2.zip › Figure 2-Source Data 2/ATM 4-3 2026.03.24_20.05.07_Ch+Marker.jpg]

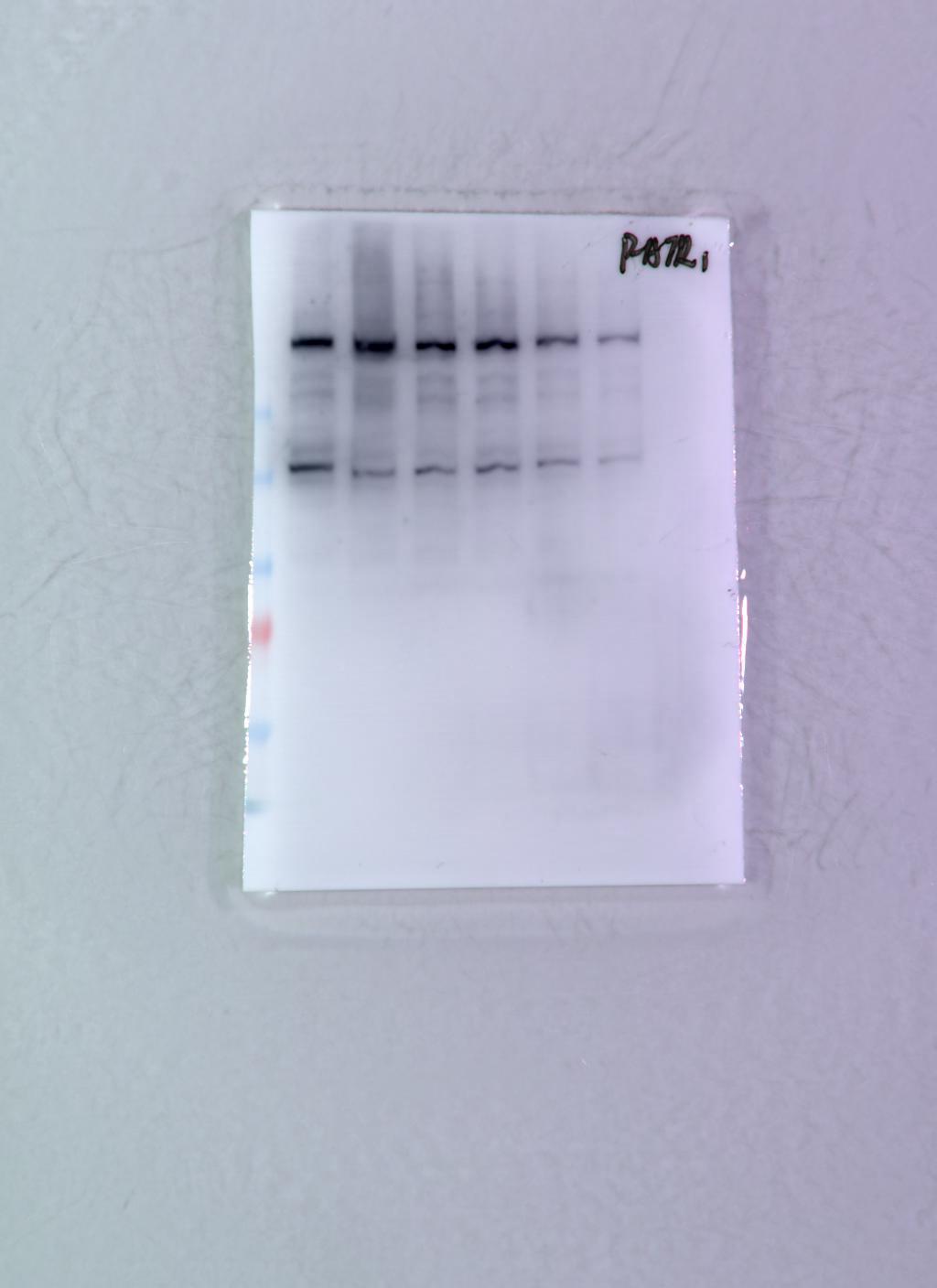

Supplement: Figure 2—source data 2. [file elife-110309-fig2-data2.zip › Figure 2-Source Data 2/ATR 0-6 2026.03.24_19.15.11_Ch+Marker.jpg]

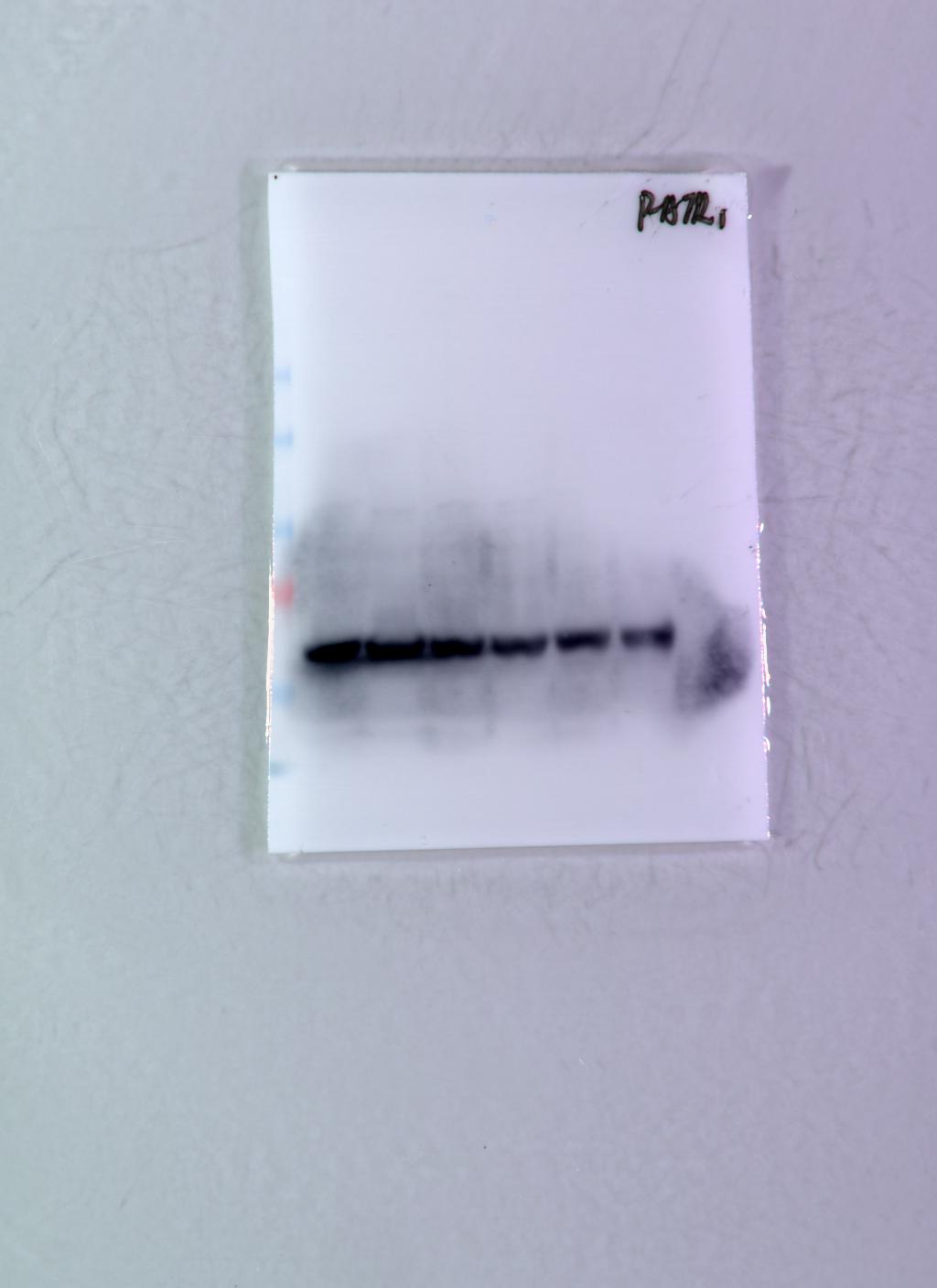

Supplement: Figure 2—source data 2. [file elife-110309-fig2-data2.zip › Figure 2-Source Data 2/CHK1-2 2026.03.25_17.01.35_Ch+Marker.jpg]

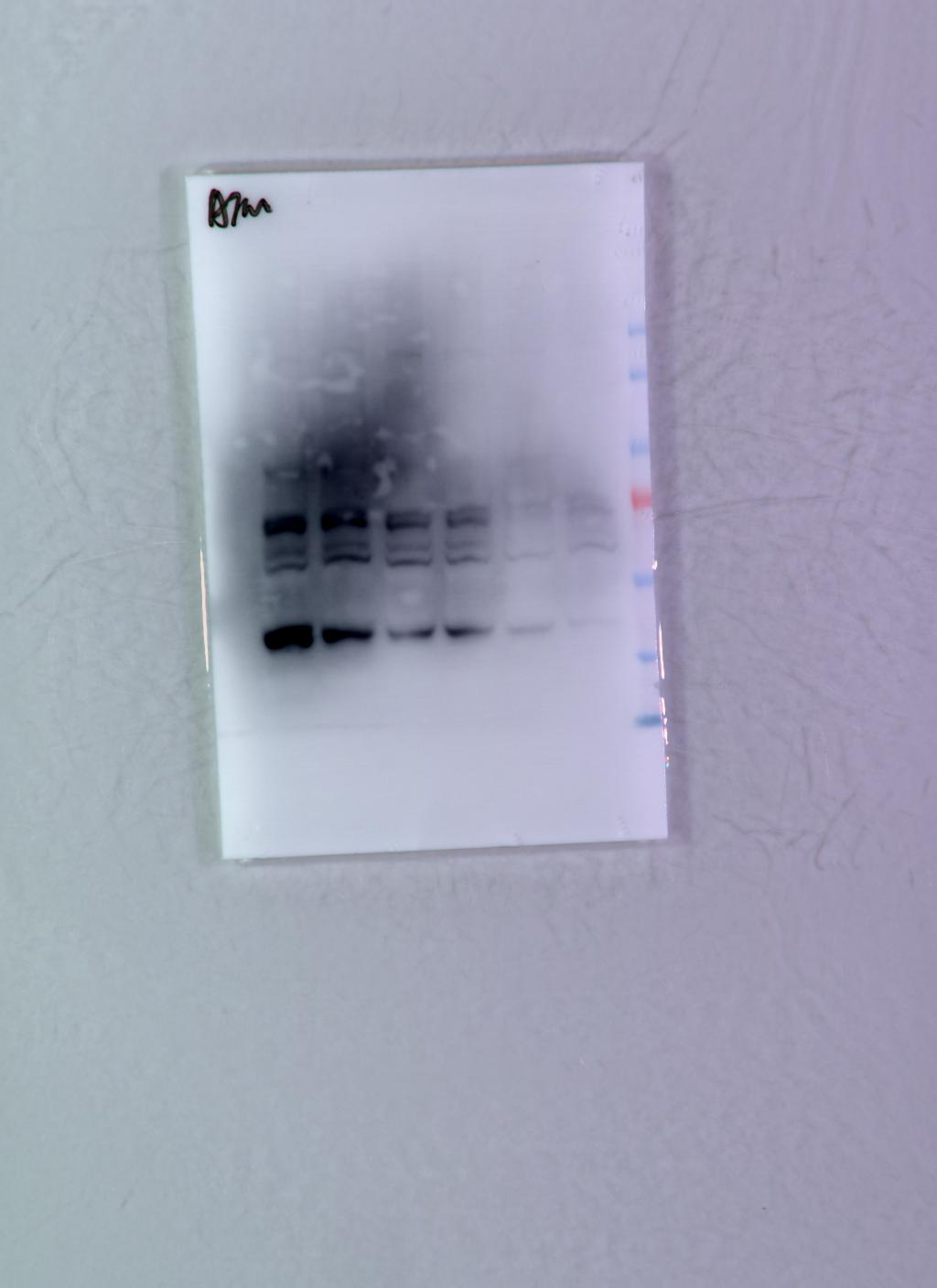

Supplement: Figure 2—source data 2. [file elife-110309-fig2-data2.zip › Figure 2-Source Data 2/CHK2-1 2026.03.25_16.57.55_Ch+Marker.jpg]

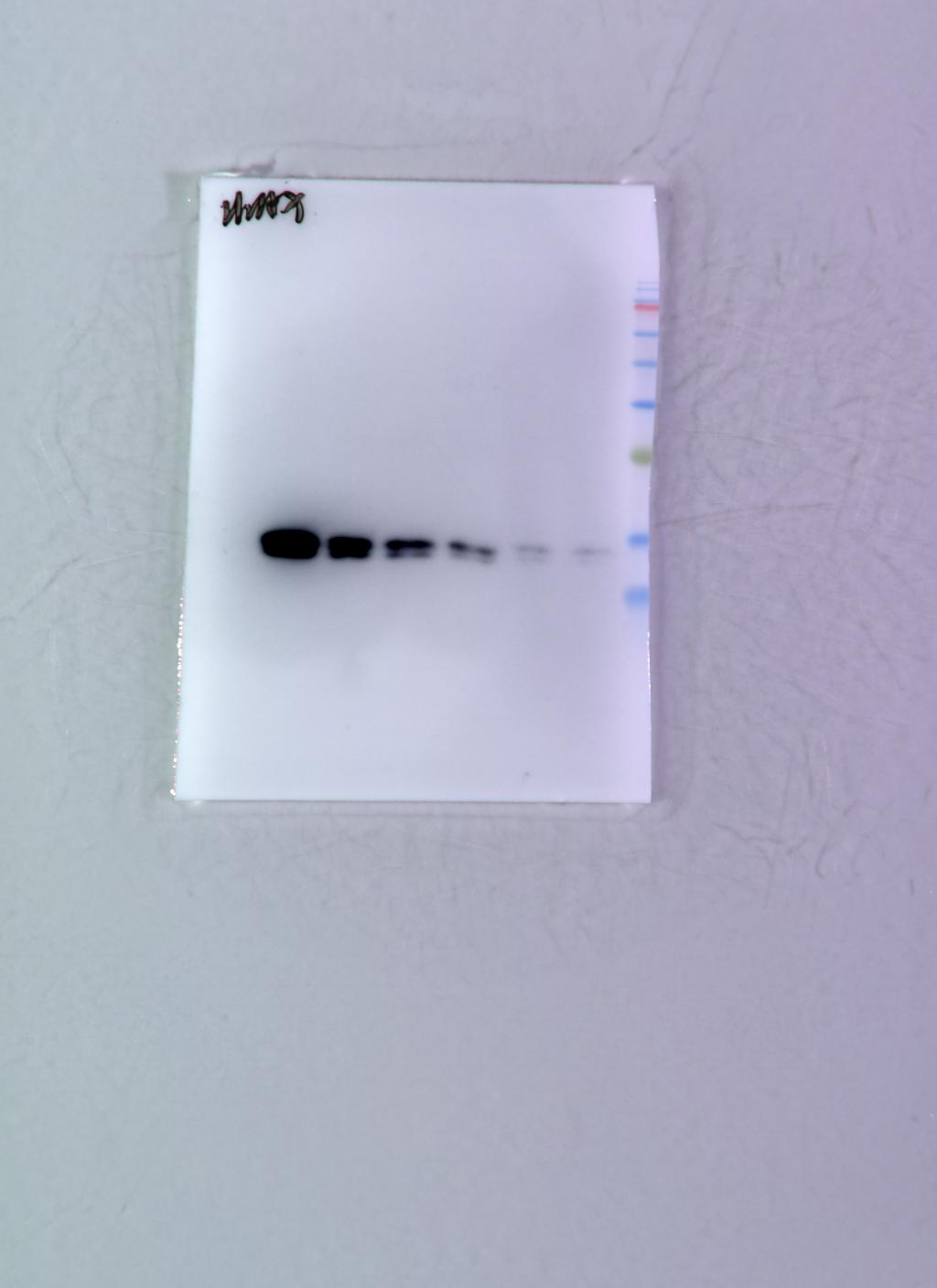

Supplement: Figure 2—source data 2. [file elife-110309-fig2-data2.zip › Figure 2-Source Data 2/h2ax 1-2 2023.11.25_21.15.59_Ch+Marker.jpg]

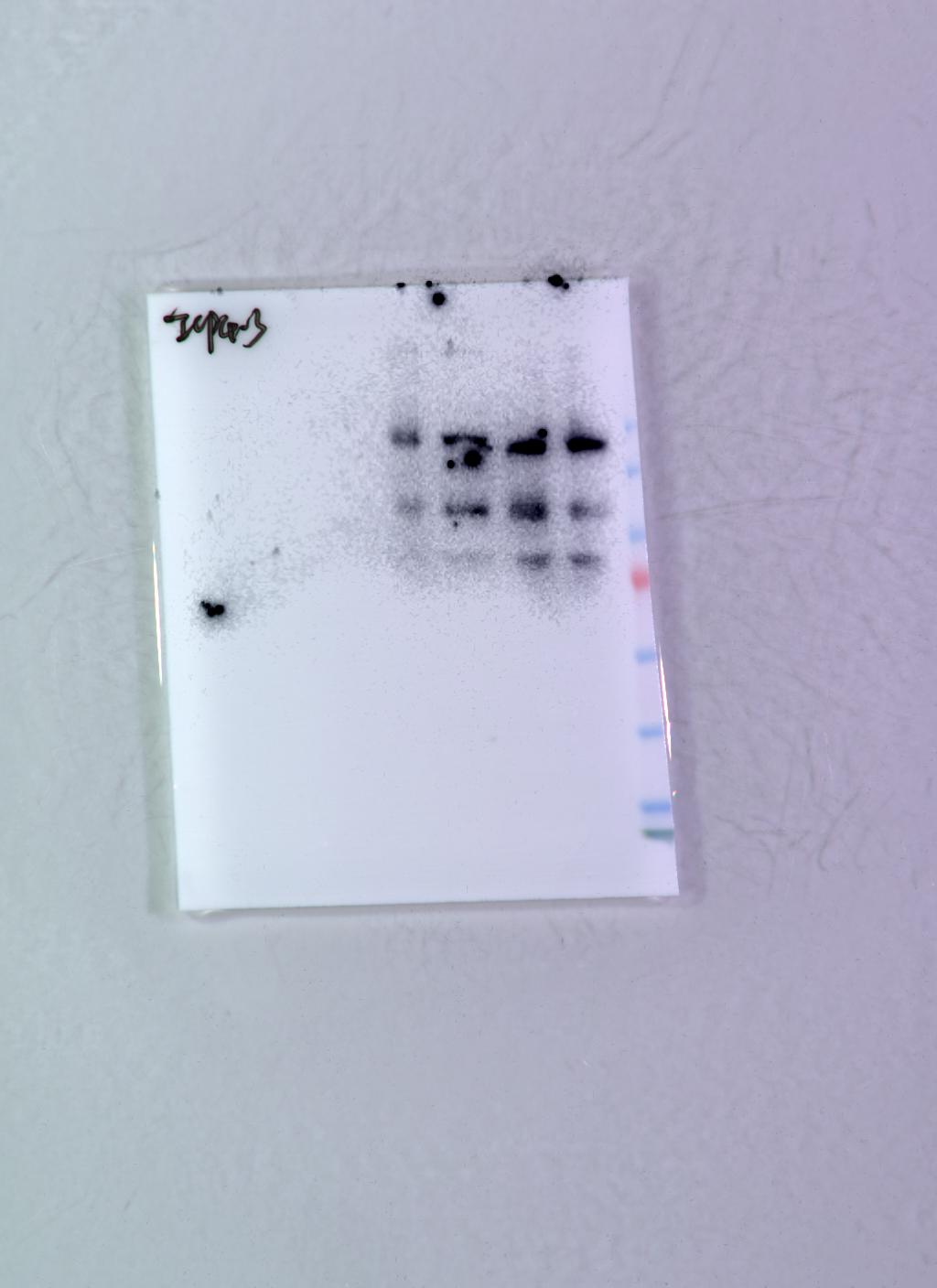

Supplement: Figure 2—source data 2. [file elife-110309-fig2-data2.zip › Figure 2-Source Data 2/ICP4 4-0 2026.03.24_21.09.09_Ch+Marker.jpg]

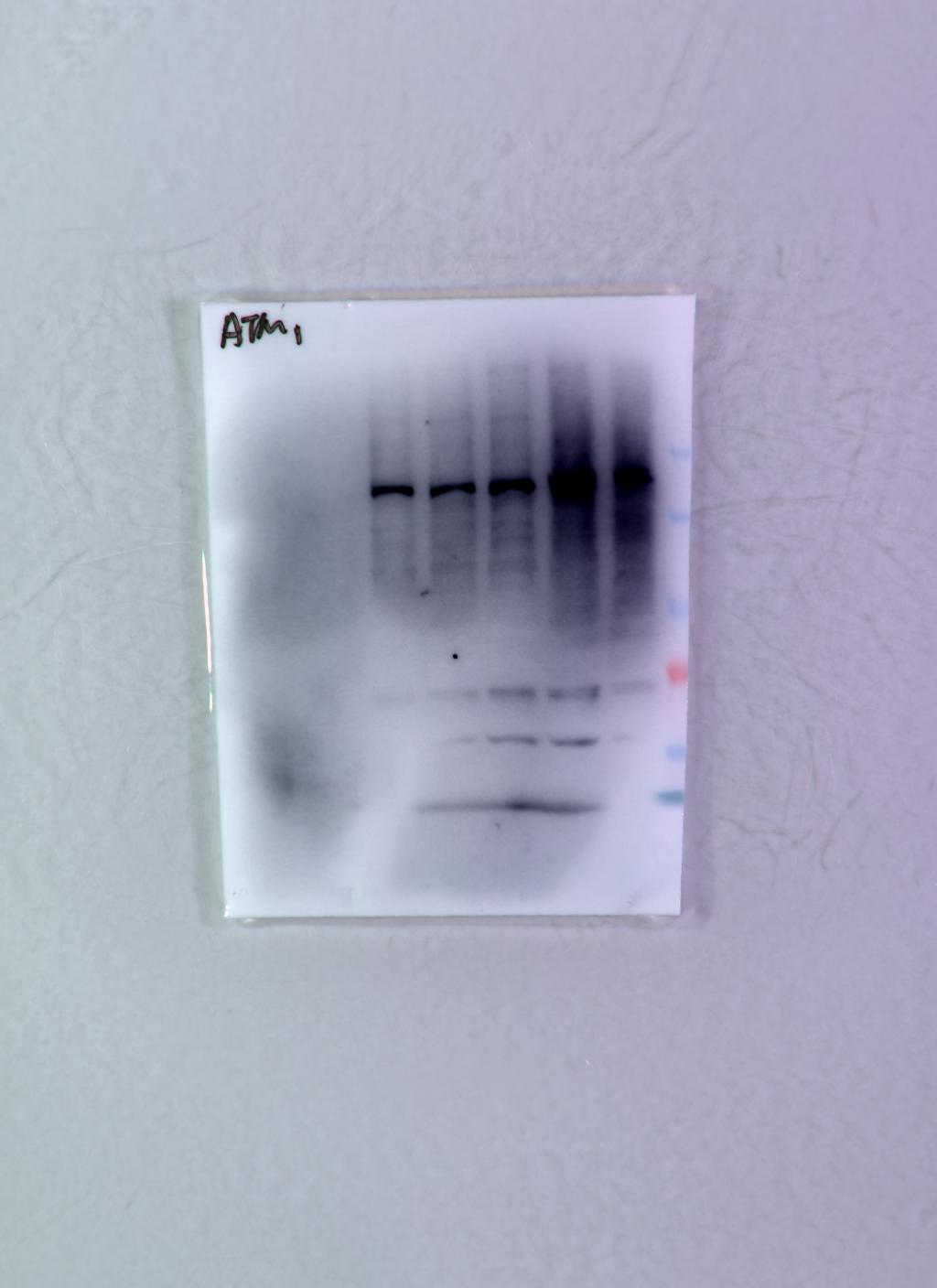

Supplement: Figure 2—source data 2. [file elife-110309-fig2-data2.zip › Figure 2-Source Data 2/P-ATM 4-0 2026.03.24_18.12.54_Ch+Marker.jpg]

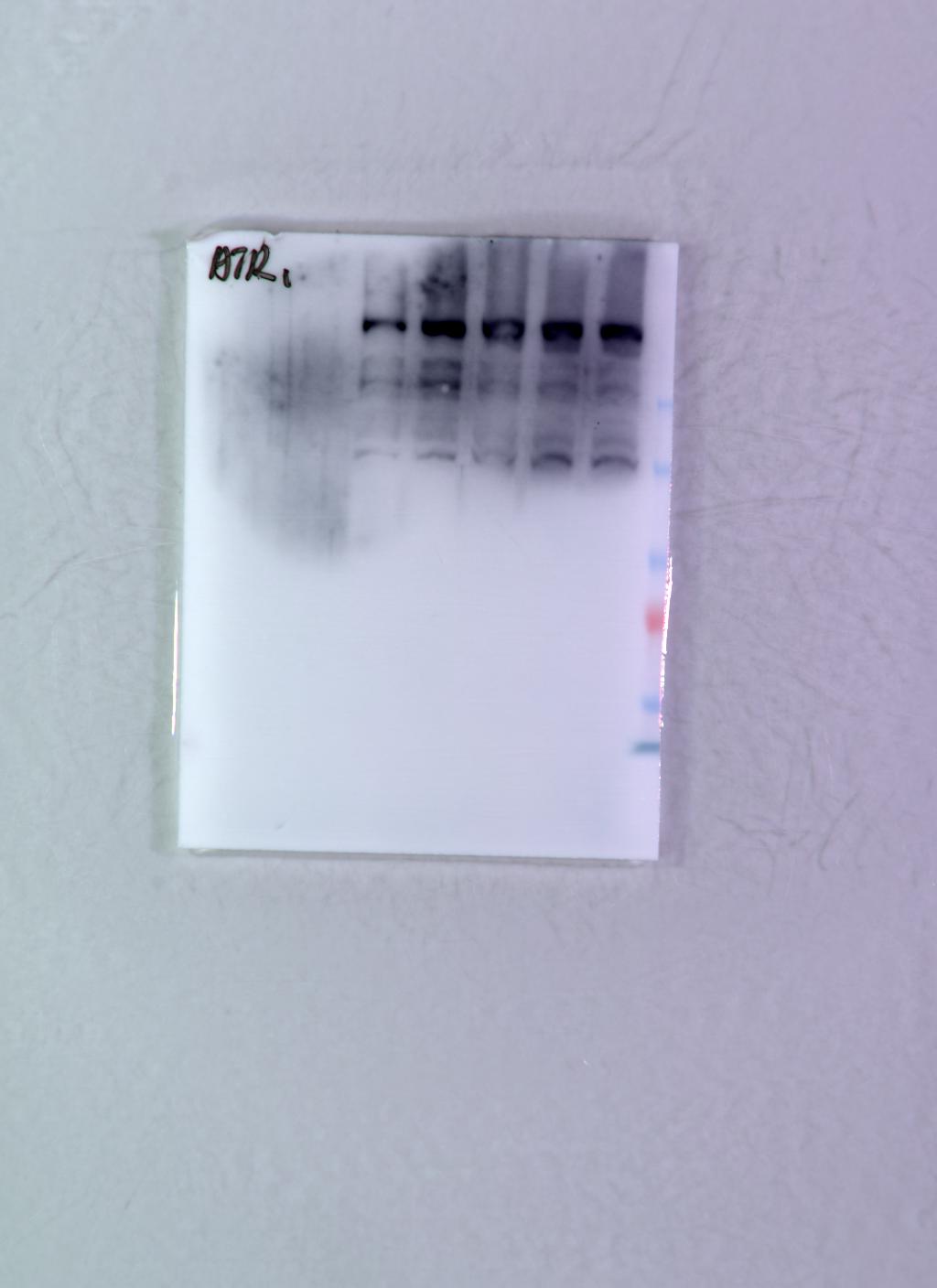

Supplement: Figure 2—source data 2. [file elife-110309-fig2-data2.zip › Figure 2-Source Data 2/P-ATR 1-2 2026.03.24_19.22.54_Ch+Marker.jpg]

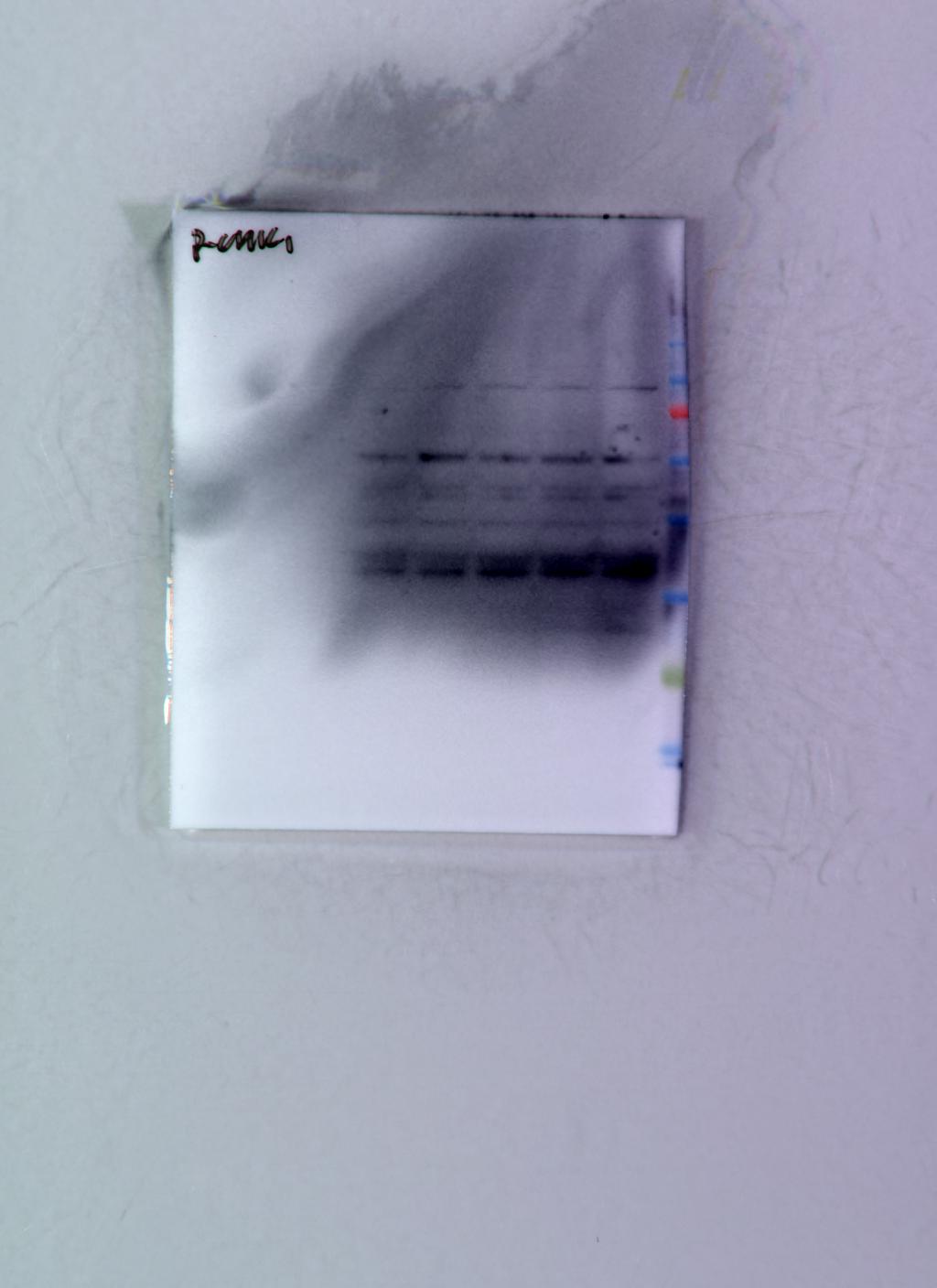

Supplement: Figure 2—source data 2. [file elife-110309-fig2-data2.zip › Figure 2-Source Data 2/P-CHK1 2-0 2023.12.03_18.55.38_Ch+Marker.jpg]

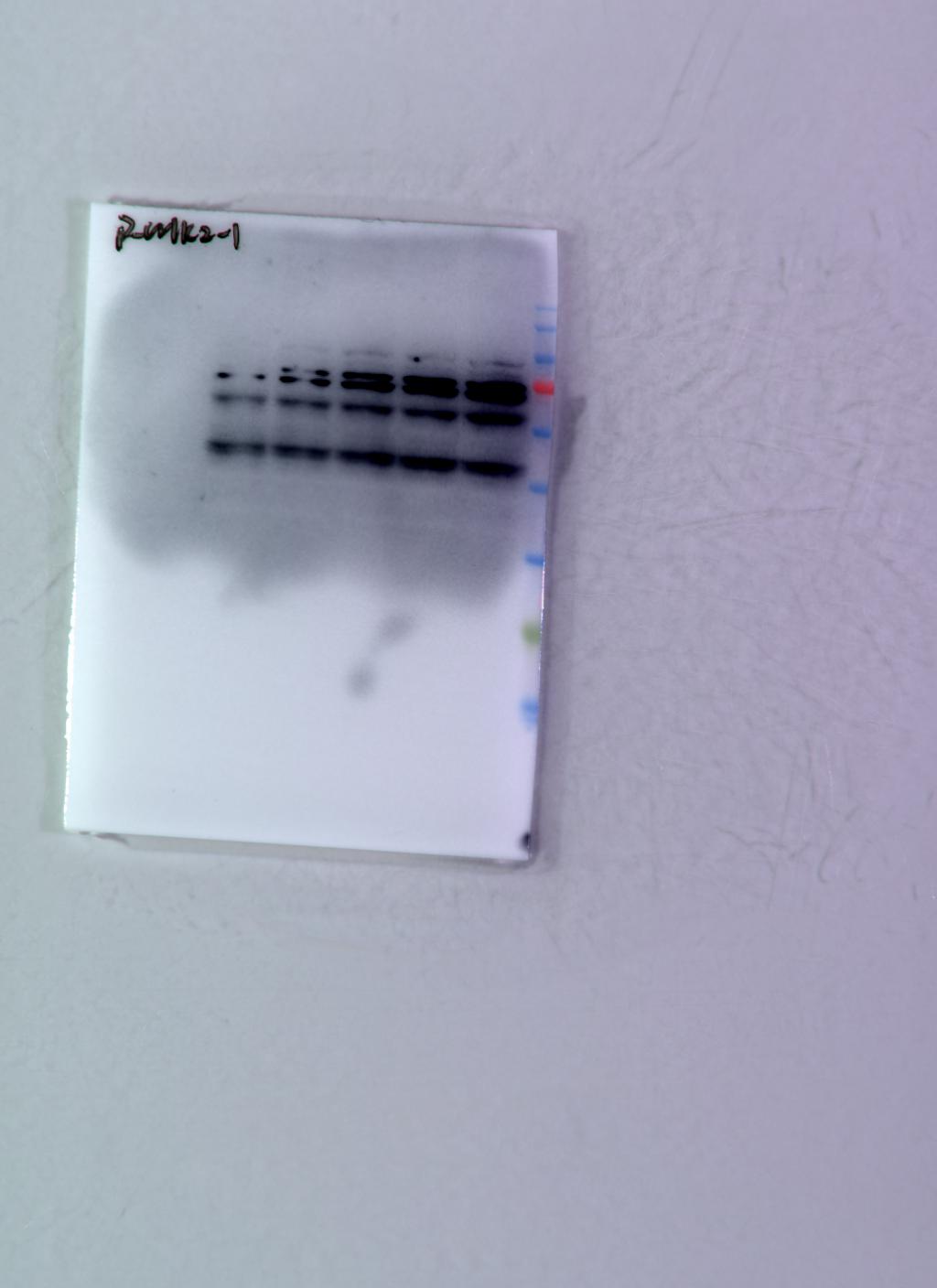

Supplement: Figure 2—source data 2. [file elife-110309-fig2-data2.zip › Figure 2-Source Data 2/P-CHK2 2-2 2023.12.03_19.49.31_Ch+Marker.jpg]

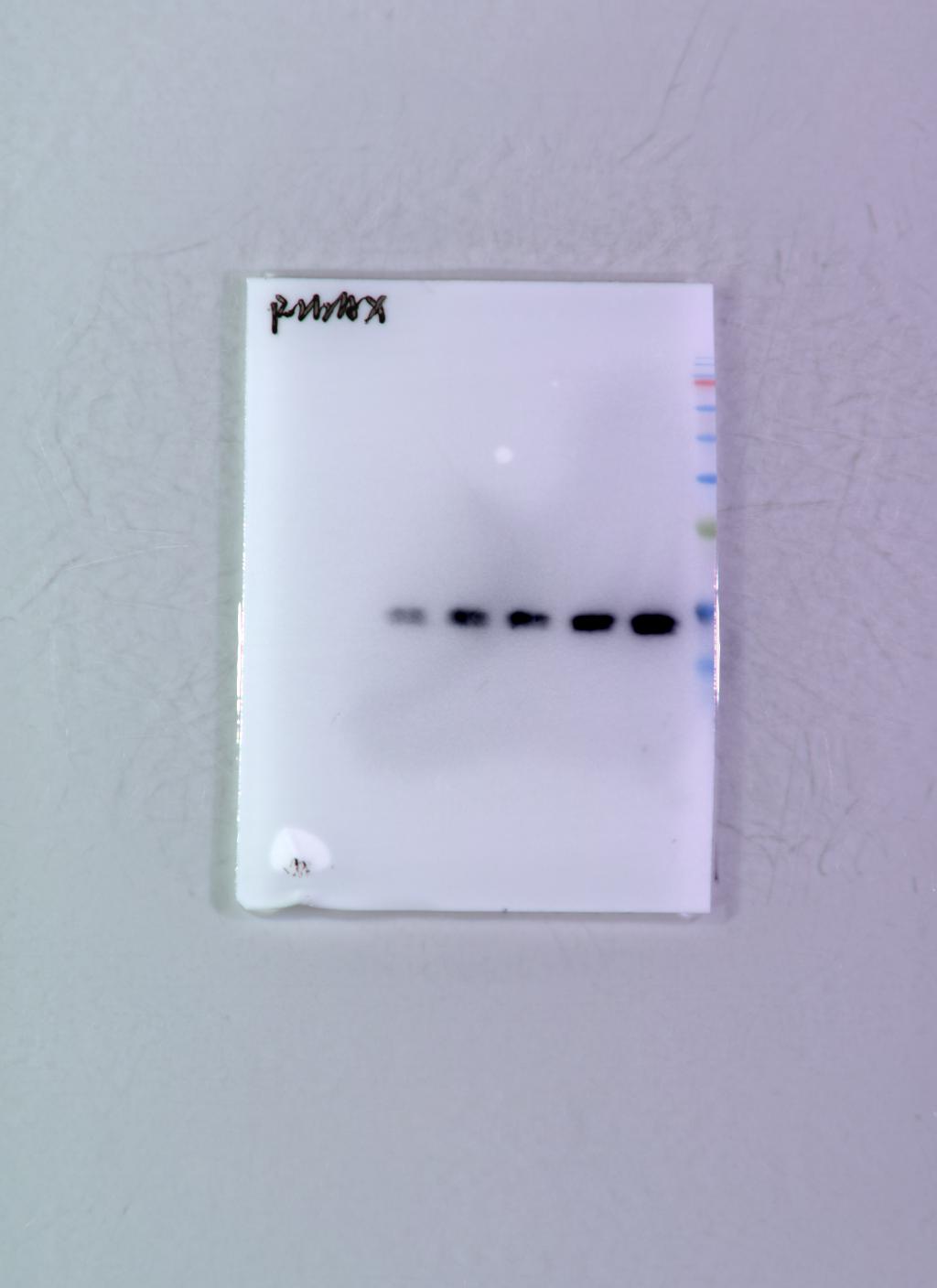

Supplement: Figure 2—source data 2. [file elife-110309-fig2-data2.zip › Figure 2-Source Data 2/p-h2ax 2-3 2023.11.25_21.32.22_Ch+Marker.jpg]

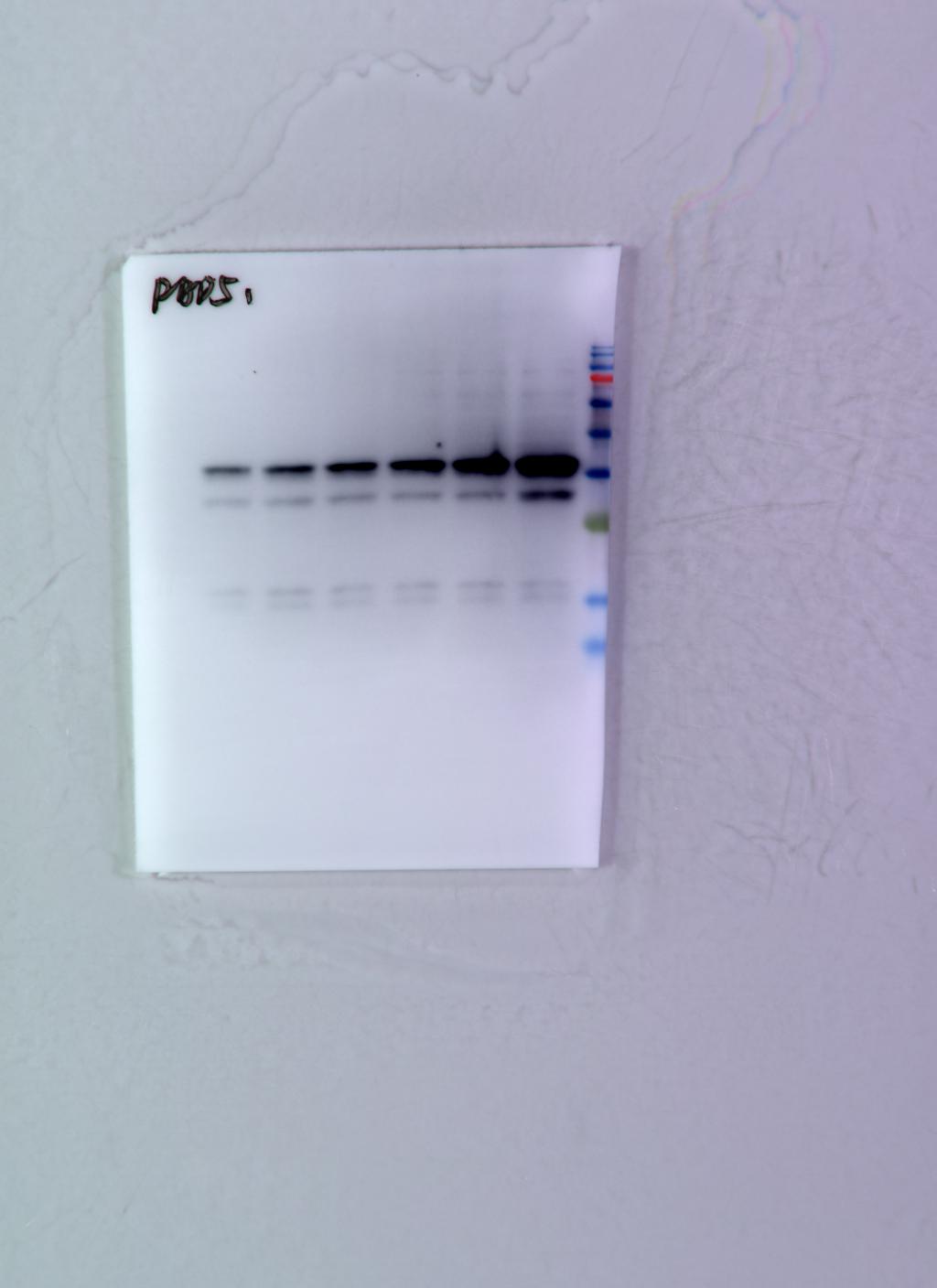

Supplement: Figure 2—source data 2. [file elife-110309-fig2-data2.zip › Figure 2-Source Data 2/RAD51 1-1 2023.12.03_18.08.35_Ch+Marker.jpg]

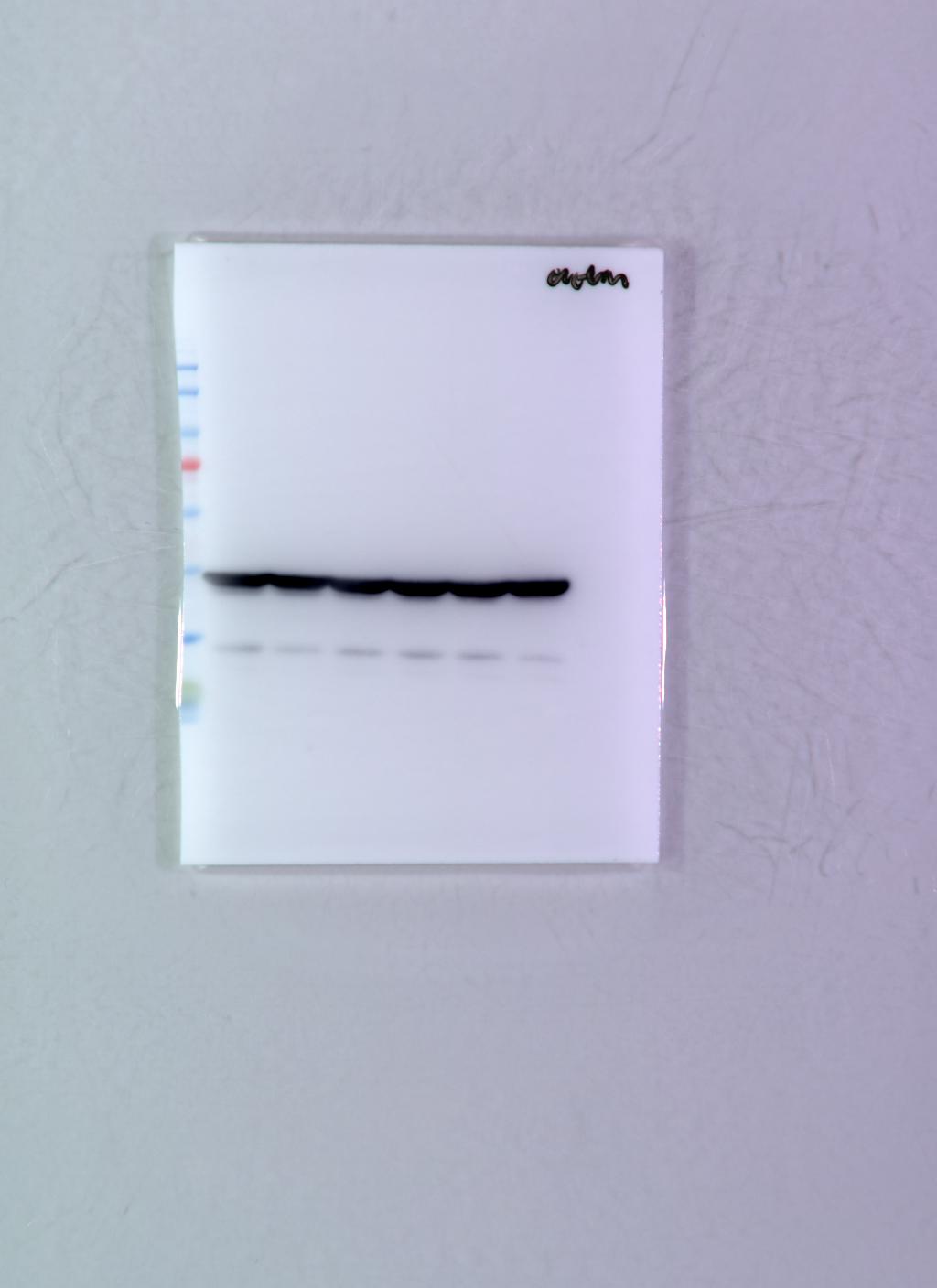

Supplement: Figure 2—source data 2. [file elife-110309-fig2-data2.zip › Figure 2-Source Data 4/ACTIN 1-0 2023.12.02_12.11.57_Ch+Marker.jpg]

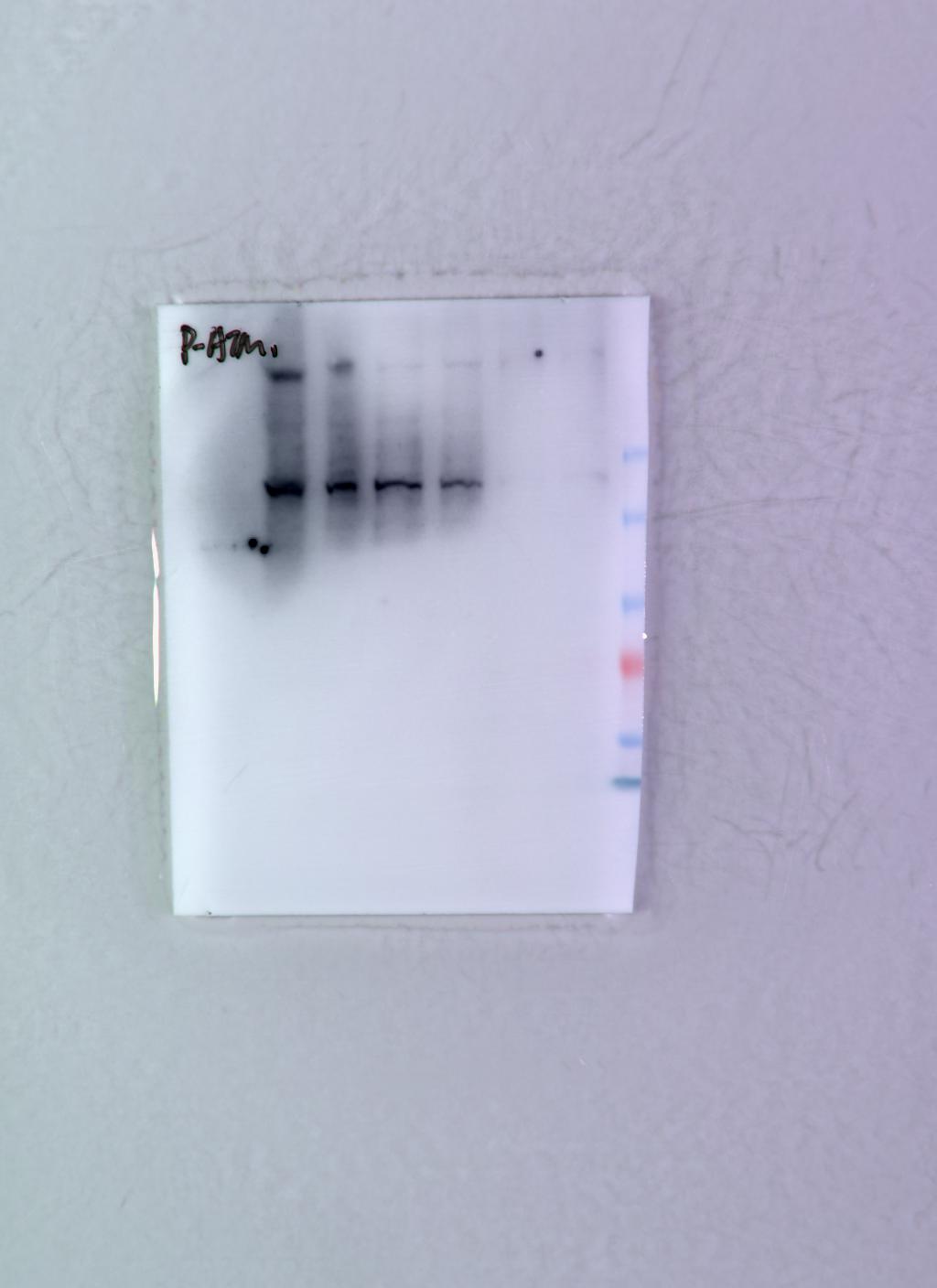

Supplement: Figure 2—source data 2. [file elife-110309-fig2-data2.zip › Figure 2-Source Data 4/ATM 5-1 2026.03.24_20.10.38_Ch+Marker.jpg]

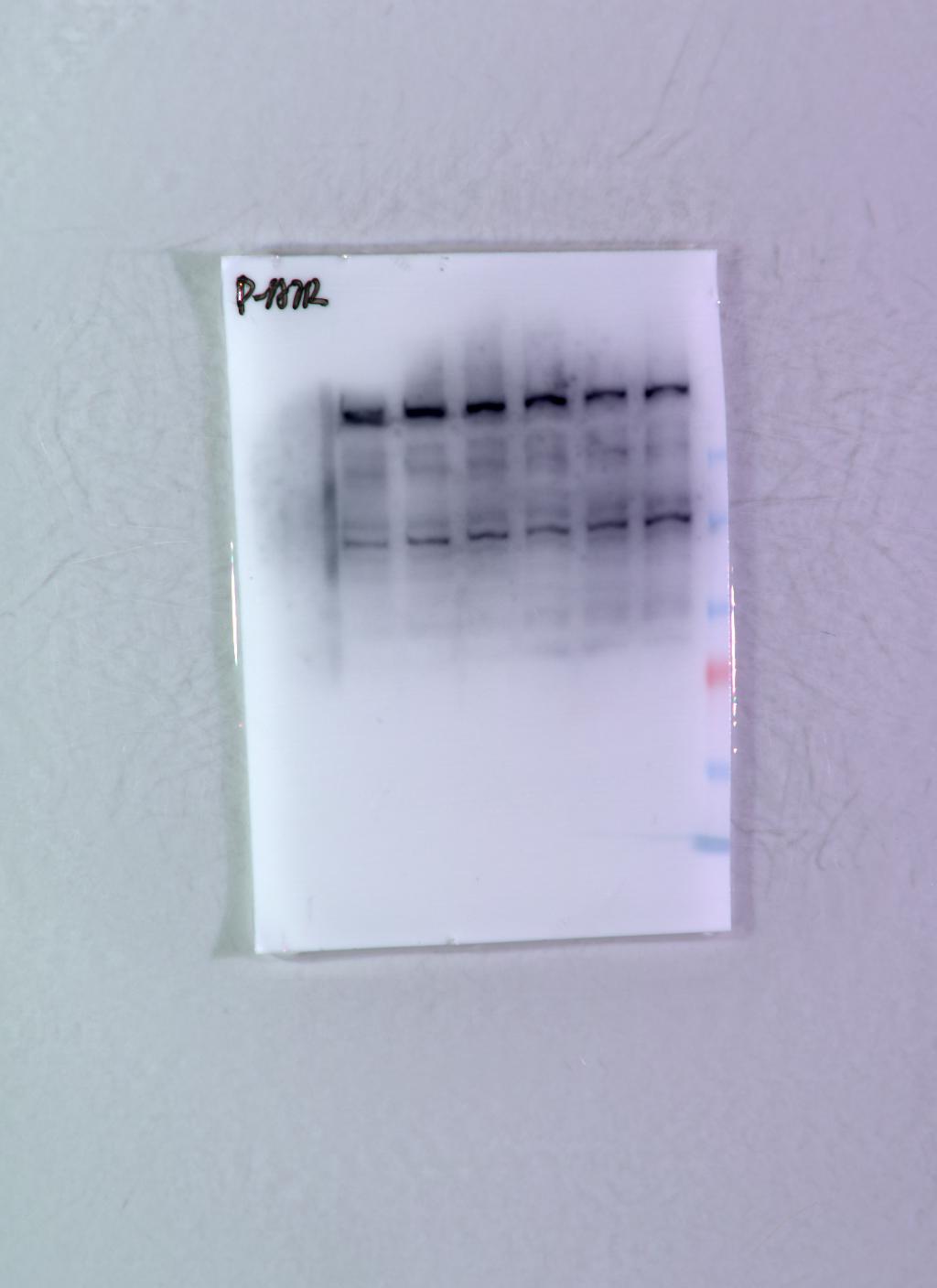

Supplement: Figure 2—source data 2. [file elife-110309-fig2-data2.zip › Figure 2-Source Data 4/ATR 1-3 2026.03.24_19.08.21_Ch+Marker.jpg]

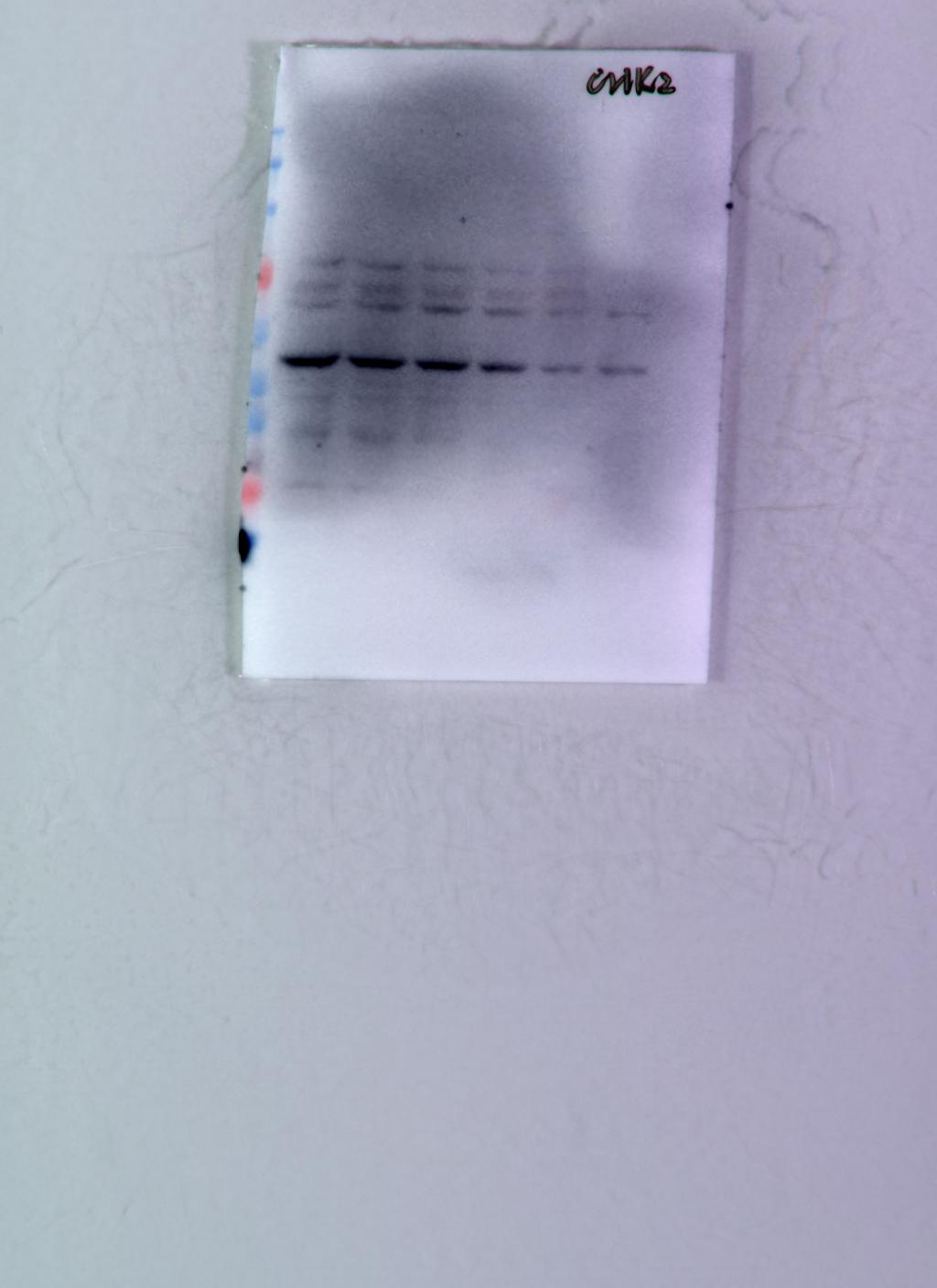

Supplement: Figure 2—source data 2. [file elife-110309-fig2-data2.zip › Figure 2-Source Data 4/CHK1 0-5 2023.12.10_11.56.51_Ch+Marker.jpg]

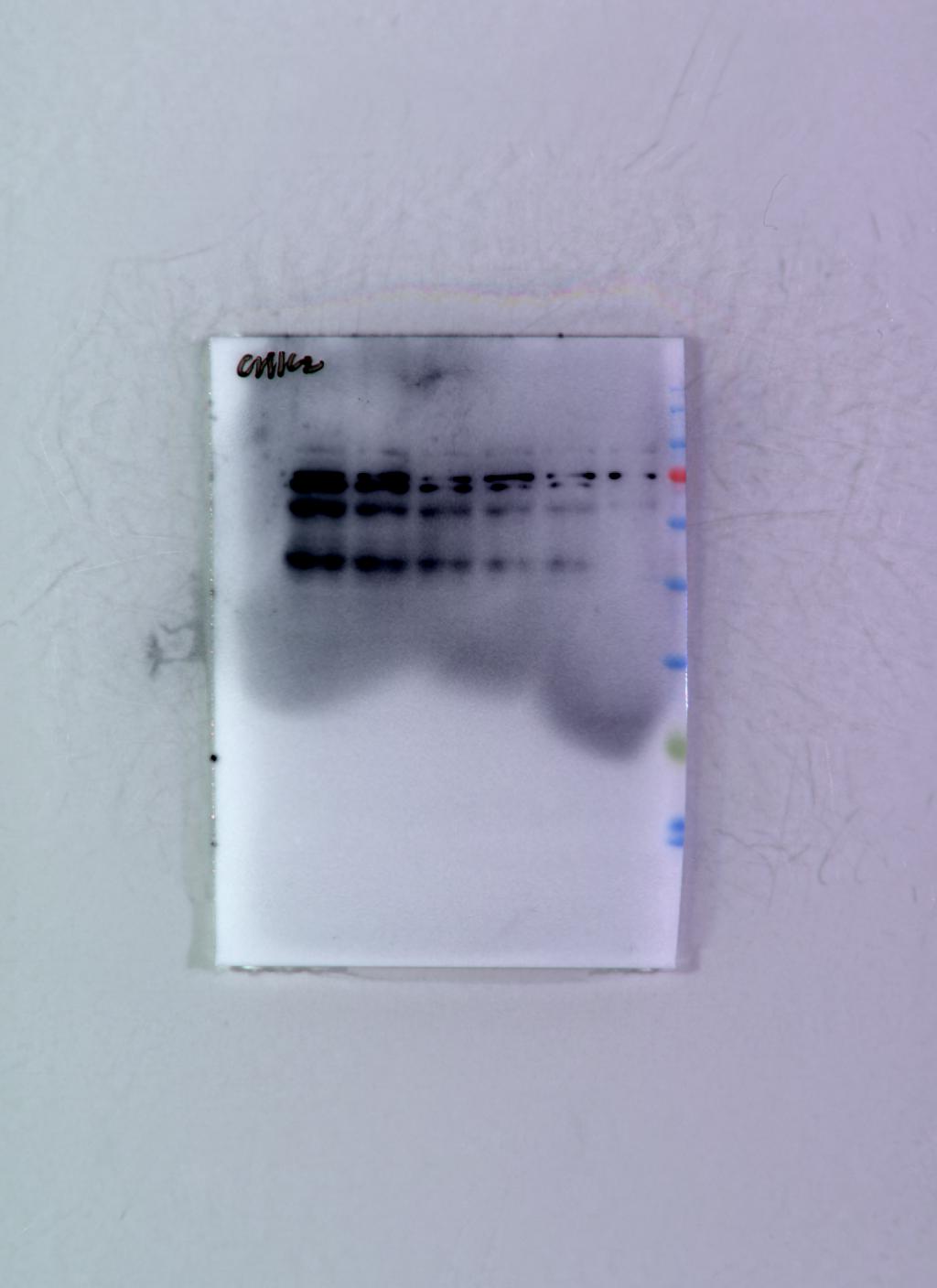

Supplement: Figure 2—source data 2. [file elife-110309-fig2-data2.zip › Figure 2-Source Data 4/CHK2 2-0 2023.12.03_20.05.41_Ch+Marker.jpg]

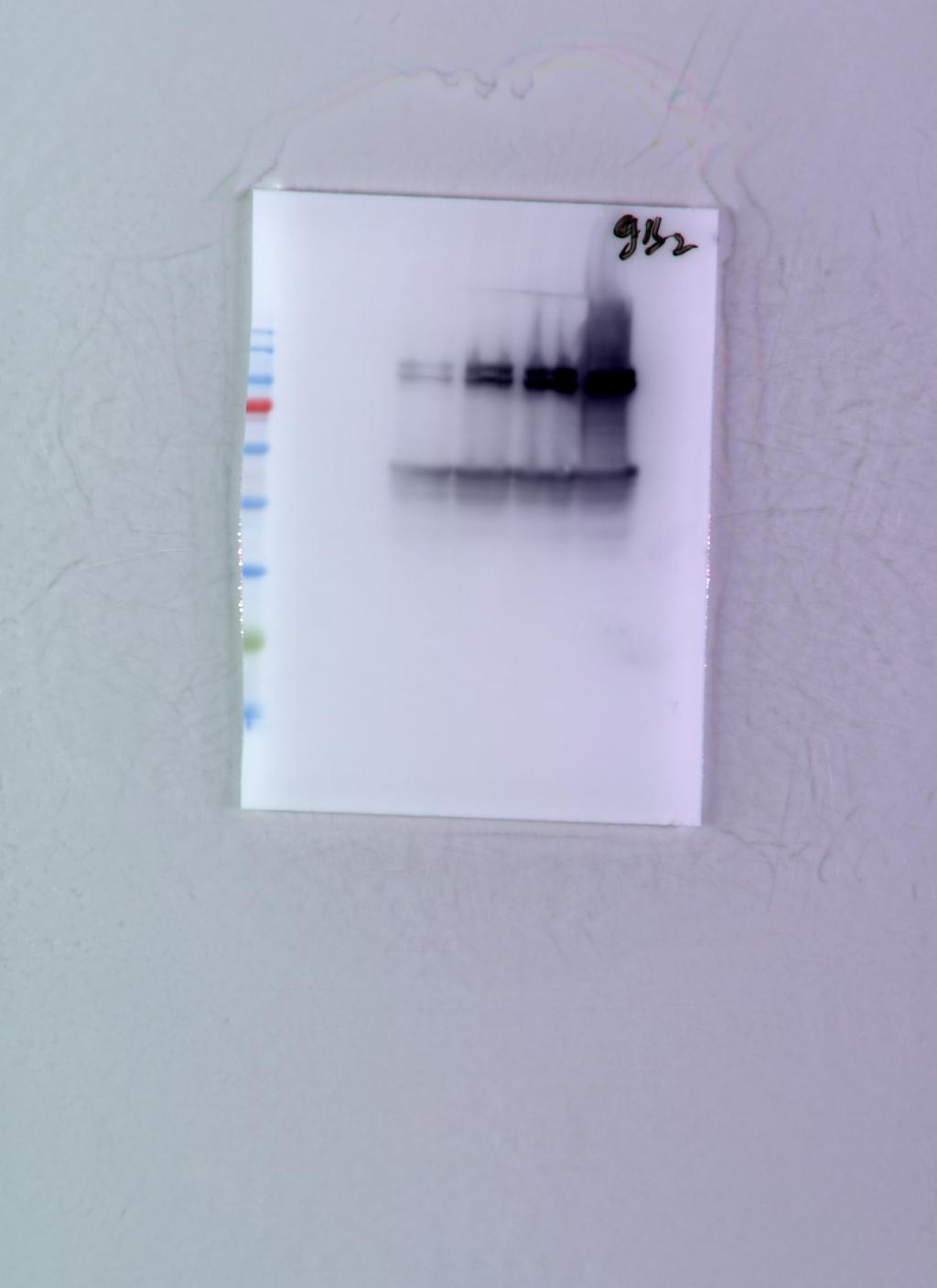

Supplement: Figure 2—source data 2. [file elife-110309-fig2-data2.zip › Figure 2-Source Data 4/GB 1-3 2023.12.03_17.46.57_Ch+Marker.jpg]

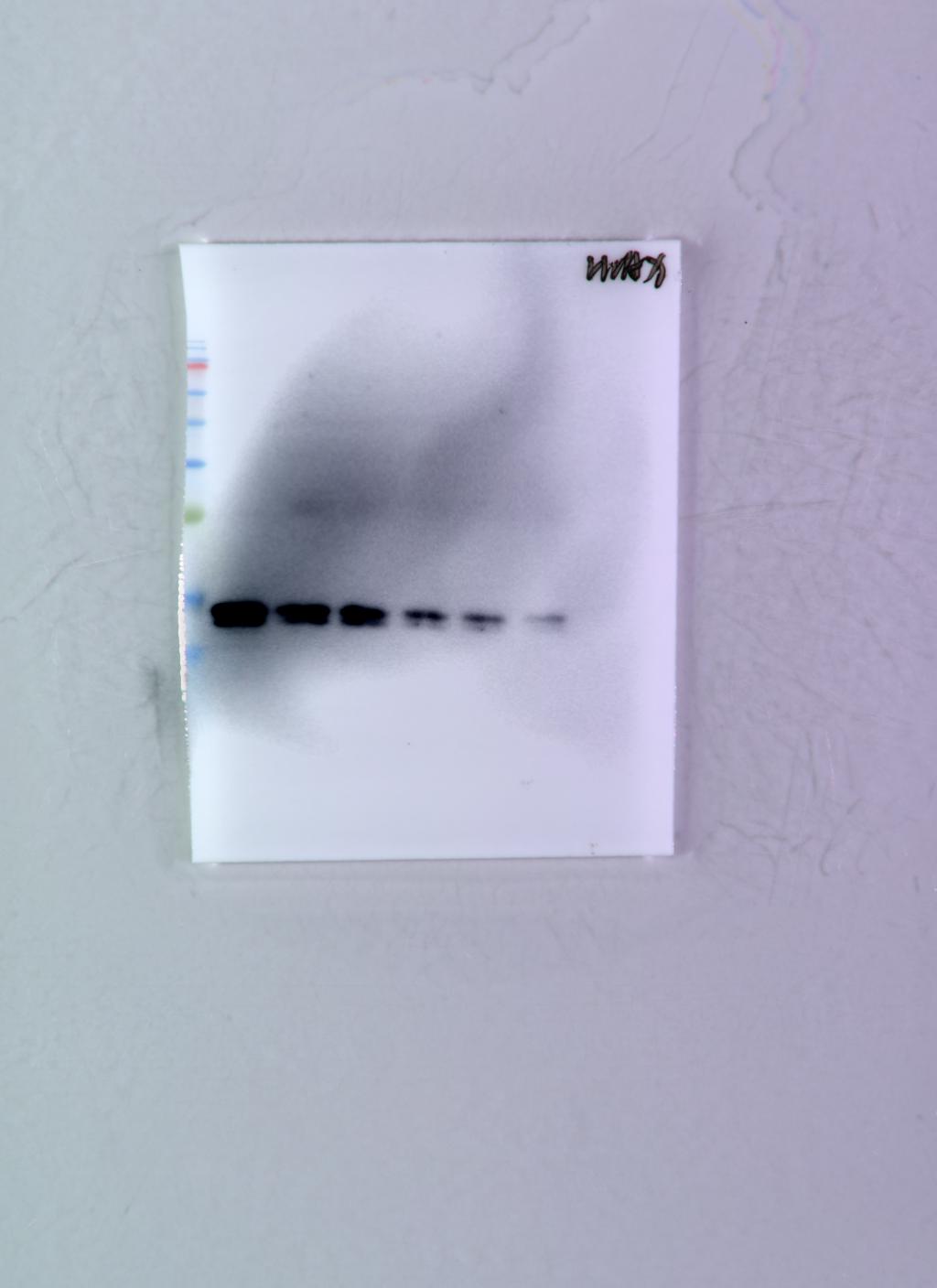

Supplement: Figure 2—source data 2. [file elife-110309-fig2-data2.zip › Figure 2-Source Data 4/h2ax 2-3 2023.11.25_21.25.39_Ch+Marker.jpg]

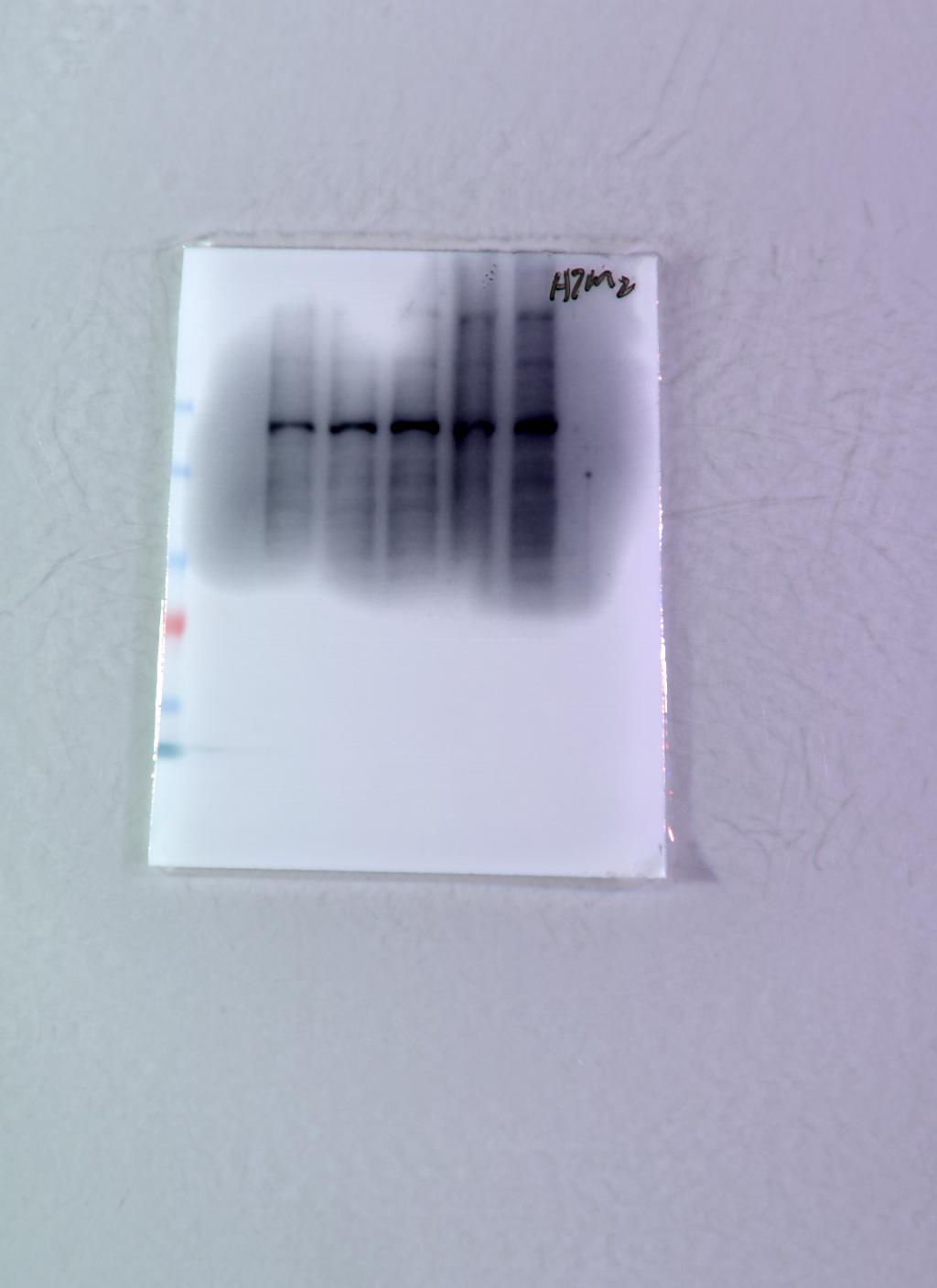

Supplement: Figure 2—source data 2. [file elife-110309-fig2-data2.zip › Figure 2-Source Data 4/P-ATM 3-2 2026.03.24_18.09.25_Ch+Marker.jpg]

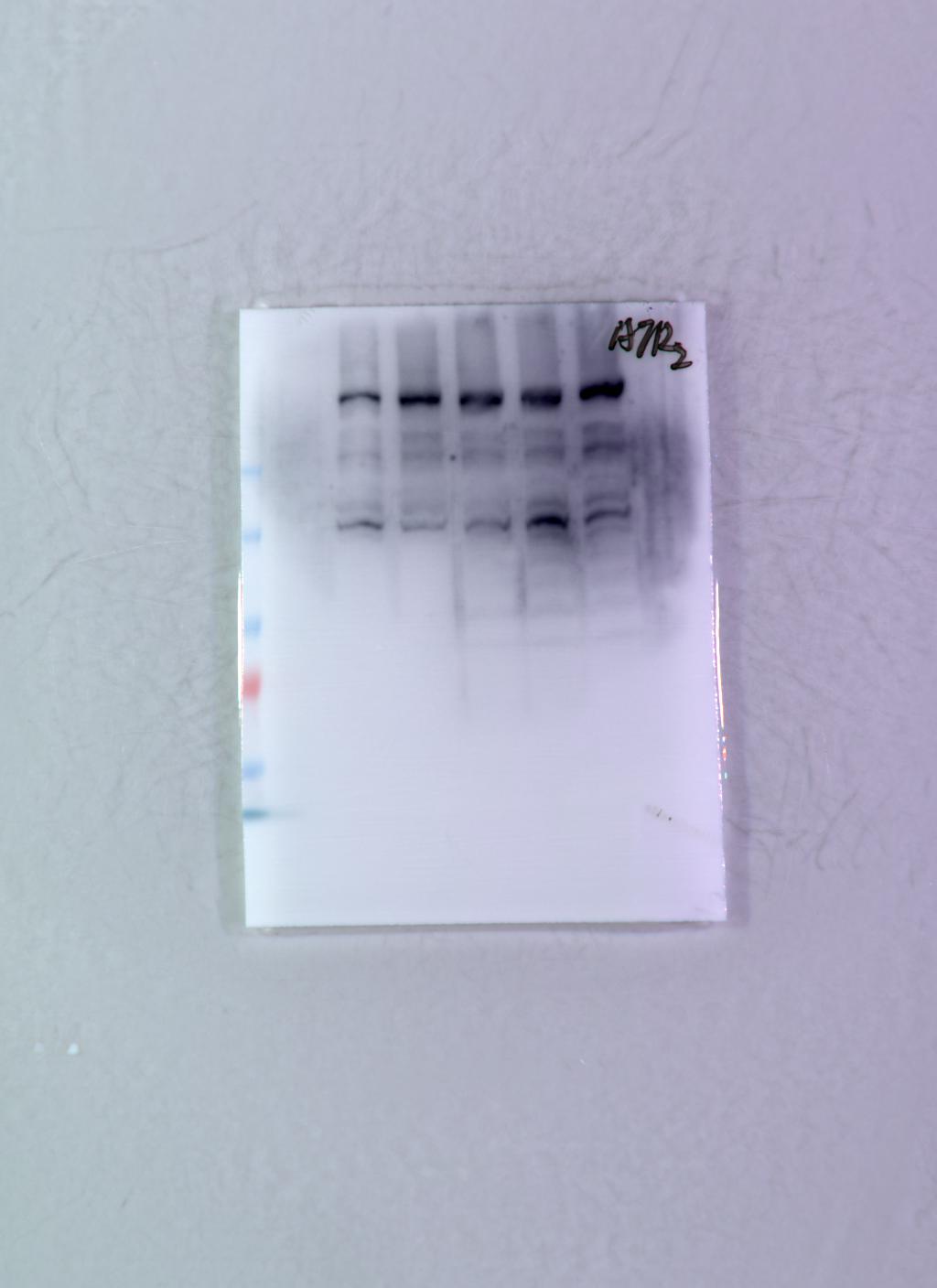

Supplement: Figure 2—source data 2. [file elife-110309-fig2-data2.zip › Figure 2-Source Data 4/P-ATR 0-2 2026.03.24_19.19.00_Ch+Marker.jpg]

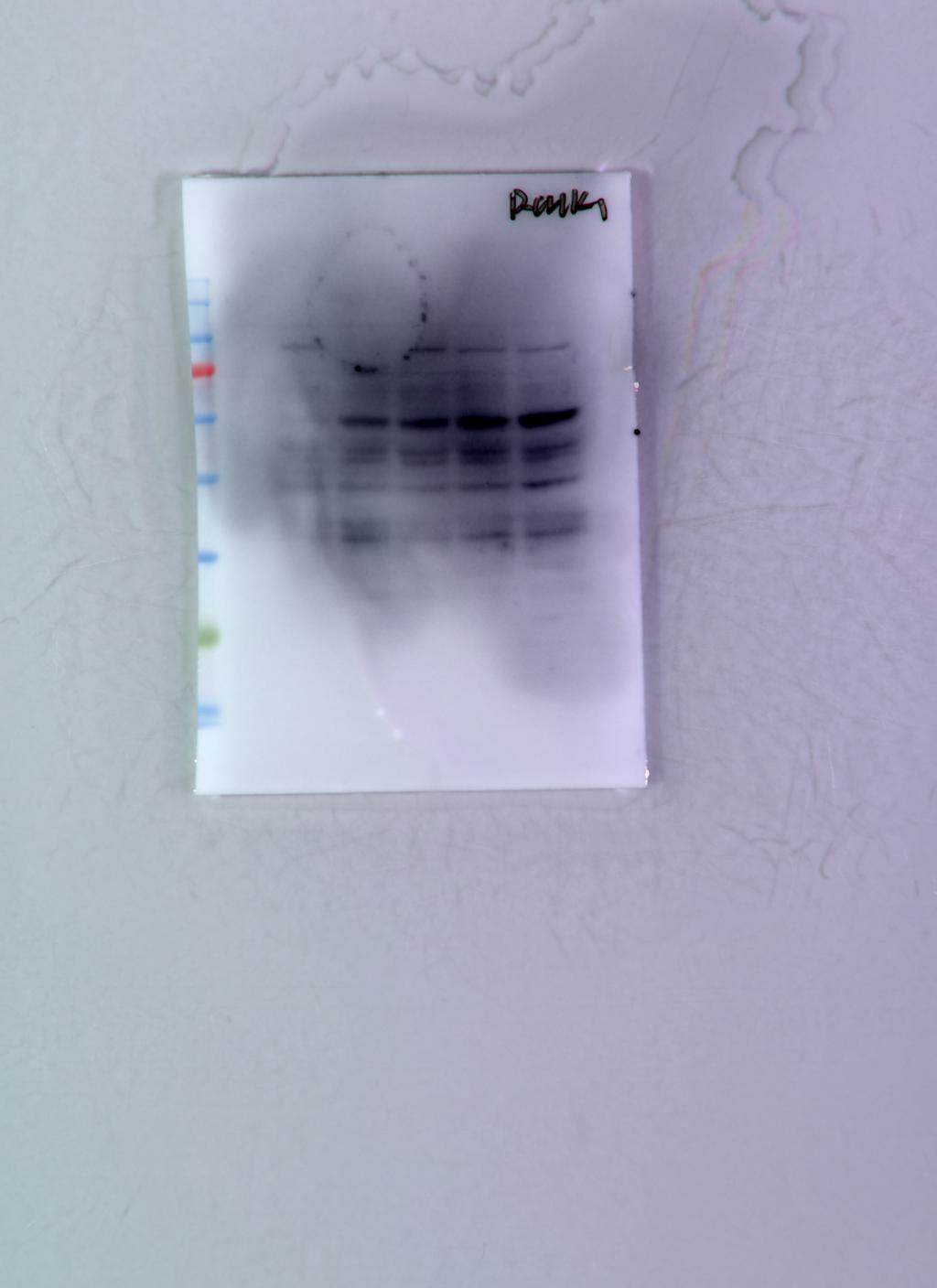

Supplement: Figure 2—source data 2. [file elife-110309-fig2-data2.zip › Figure 2-Source Data 4/P-CHK1 1-5 2023.12.03_18.52.05_Ch+Marker.jpg]

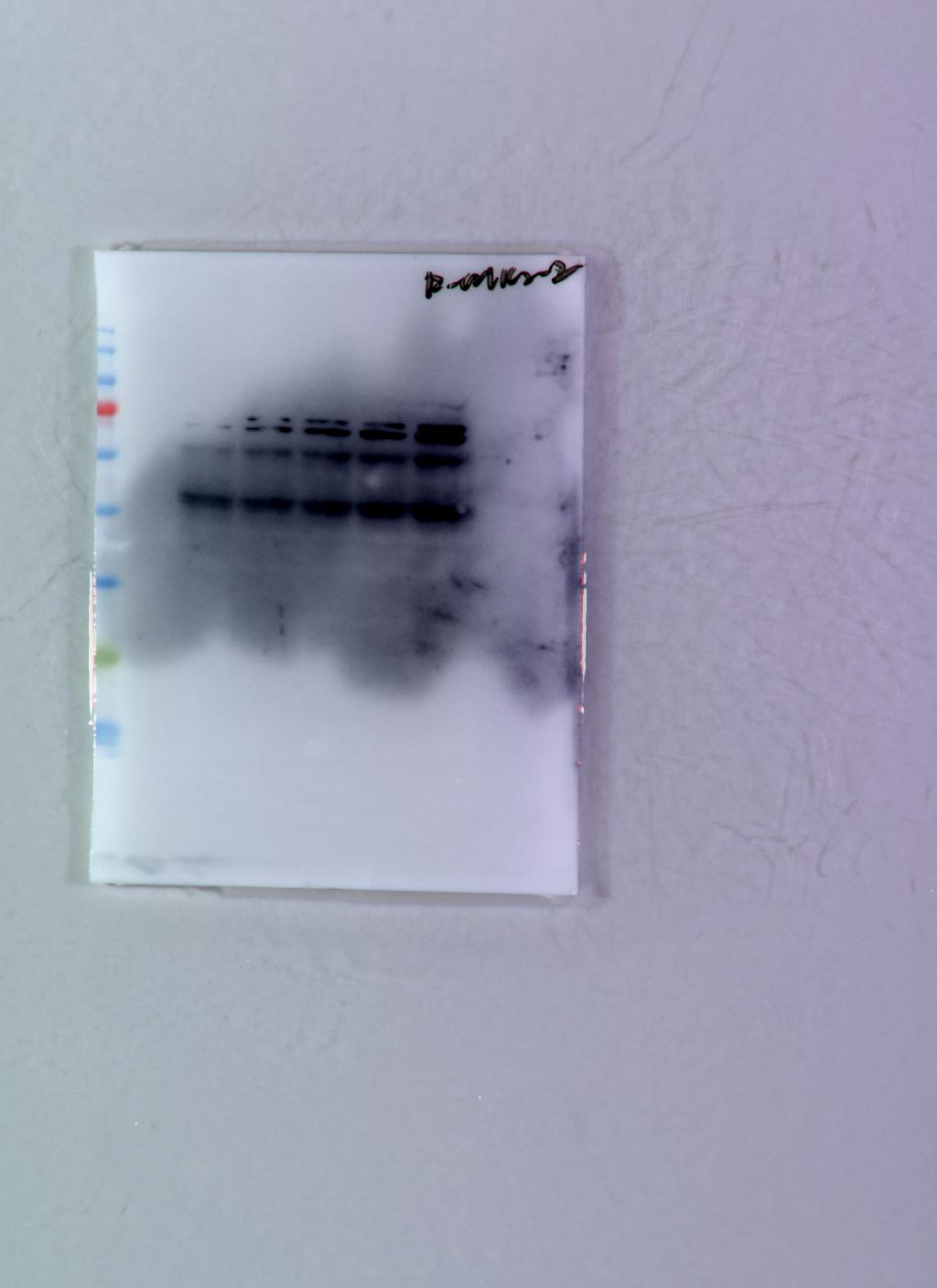

Supplement: Figure 2—source data 2. [file elife-110309-fig2-data2.zip › Figure 2-Source Data 4/P-CHK2 1-1 2023.12.03_19.54.00_Ch+Marker.jpg]

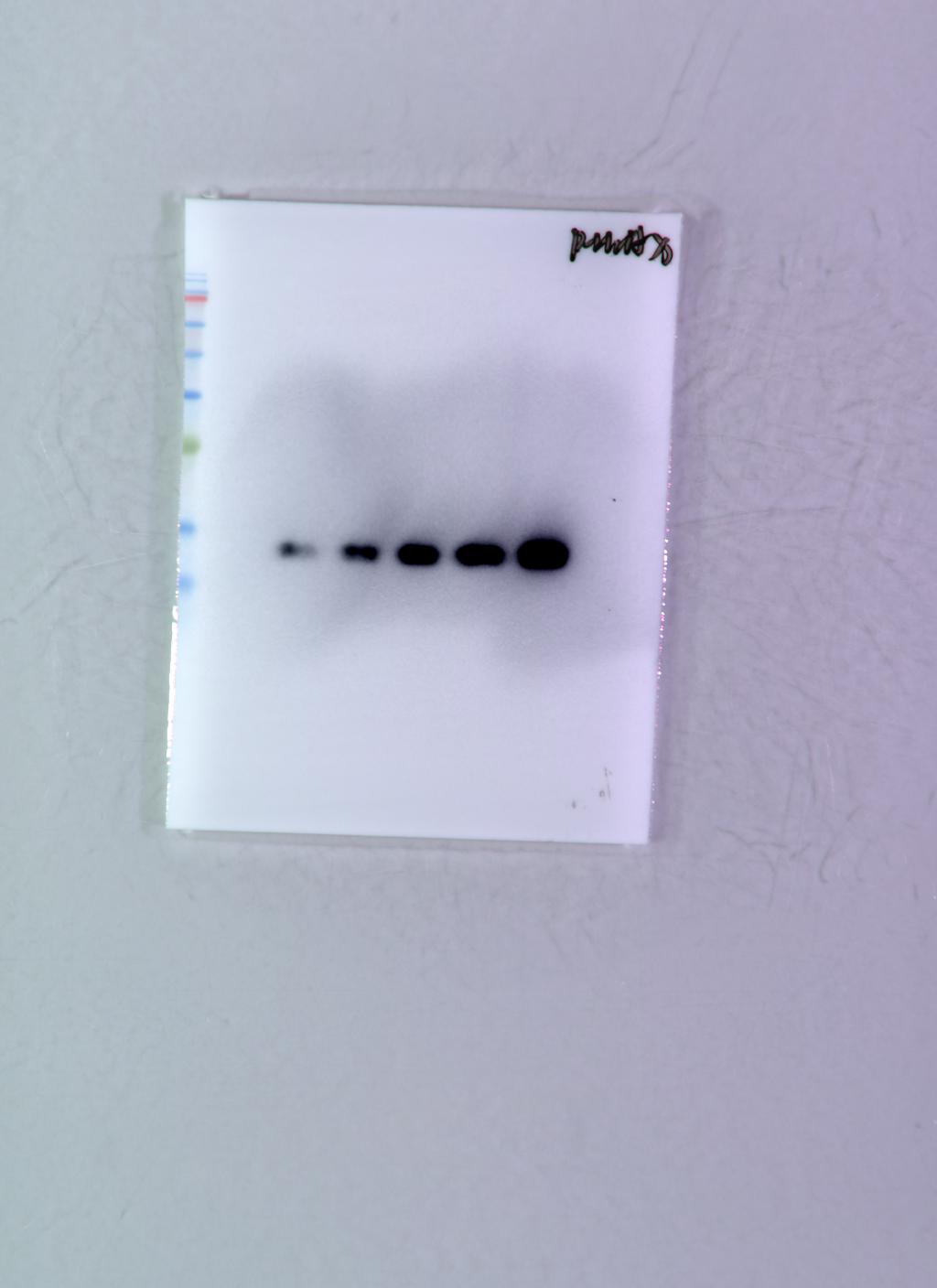

Supplement: Figure 2—source data 2. [file elife-110309-fig2-data2.zip › Figure 2-Source Data 4/p-h2ax 1-0 2023.11.25_21.28.54_Ch+Marker.jpg]

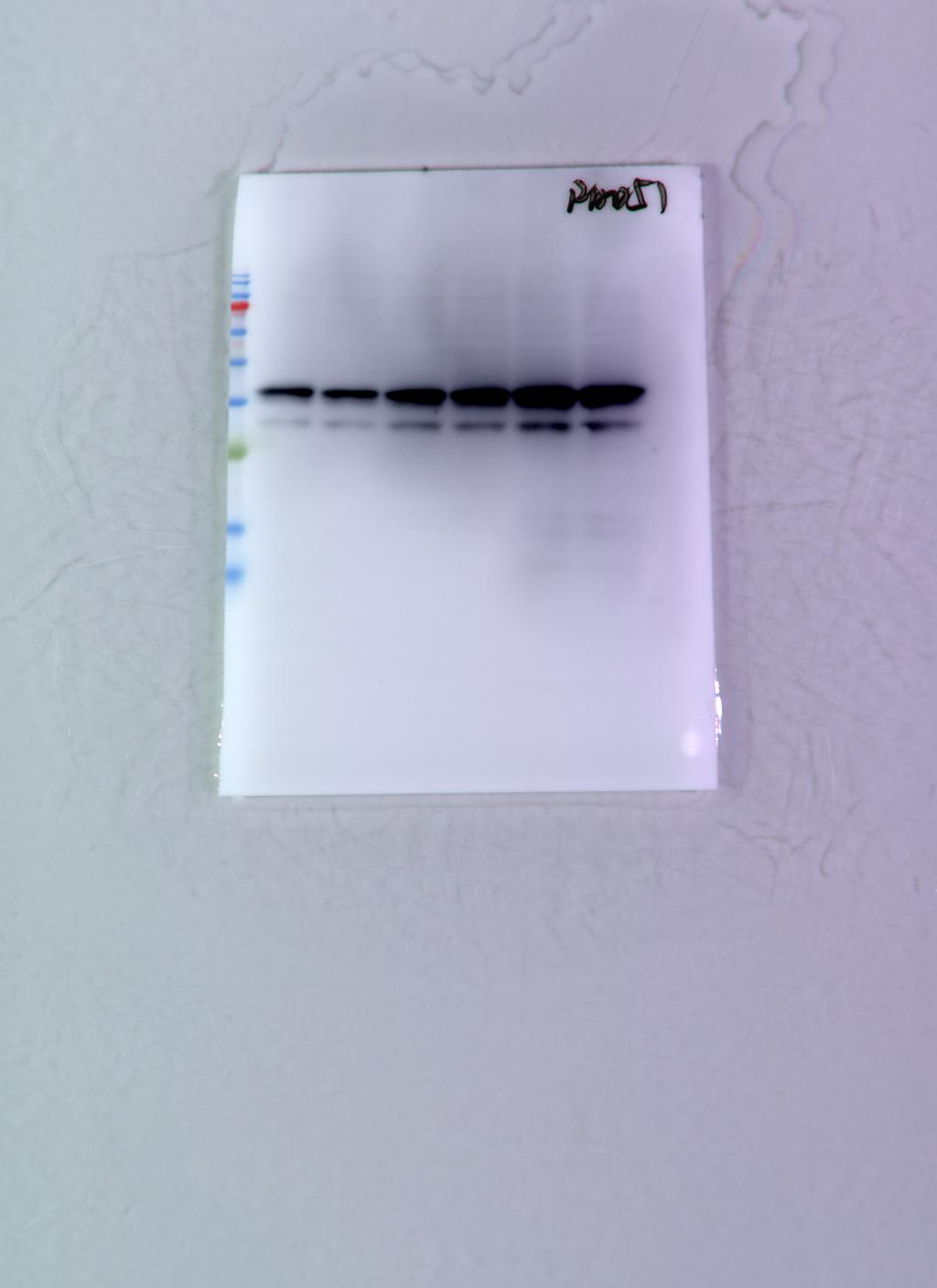

Supplement: Figure 2—source data 2. [file elife-110309-fig2-data2.zip › Figure 2-Source Data 4/RAD51 0-4 2023.12.03_18.03.41_Ch+Marker.jpg]

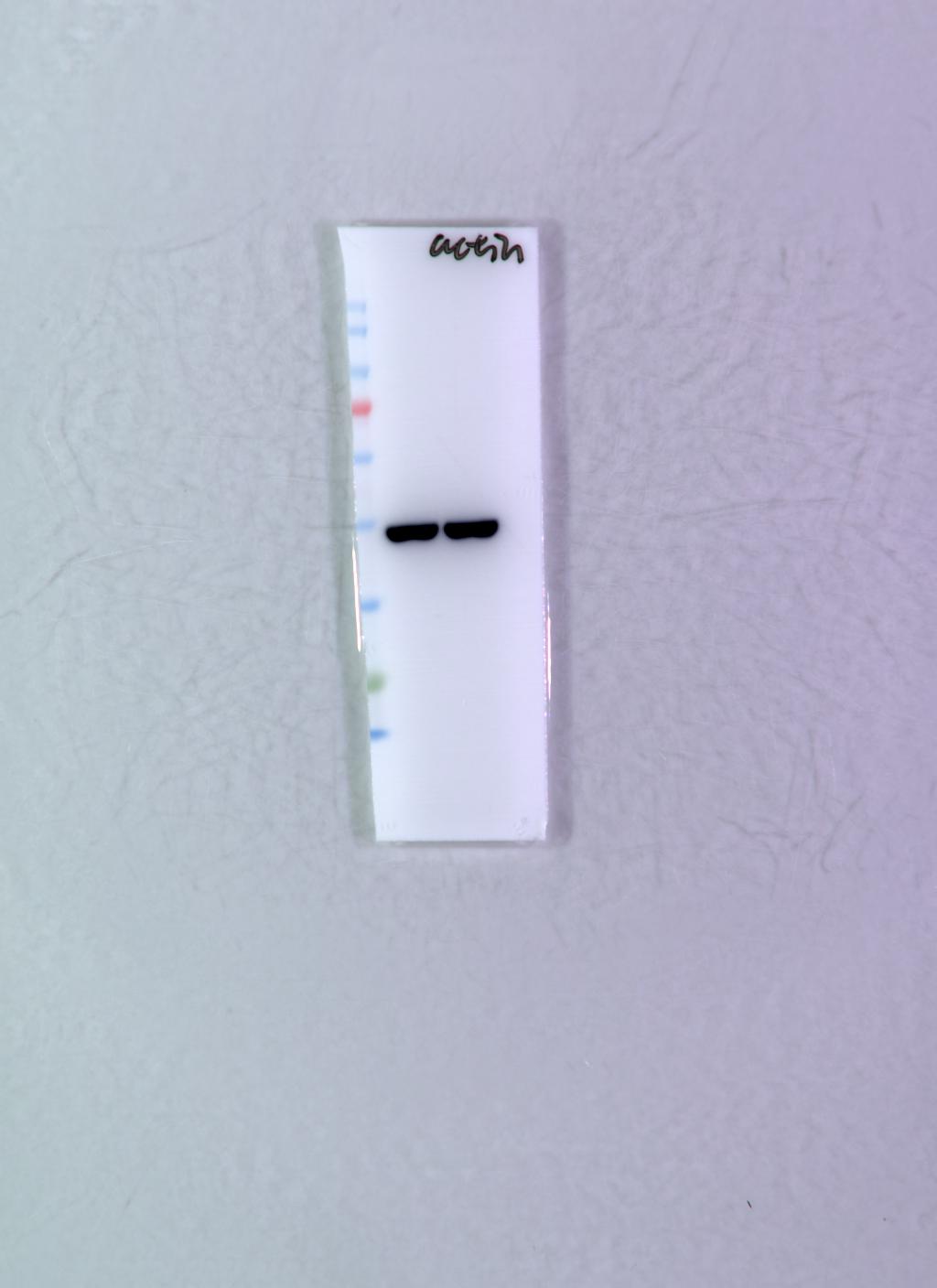

Supplement: Figure 2—source data 2. [file elife-110309-fig2-data2.zip › Figure 2-Source Data 6/ACTIN 3-3 2026.03.24_17.34.45_Ch+Marker.jpg]

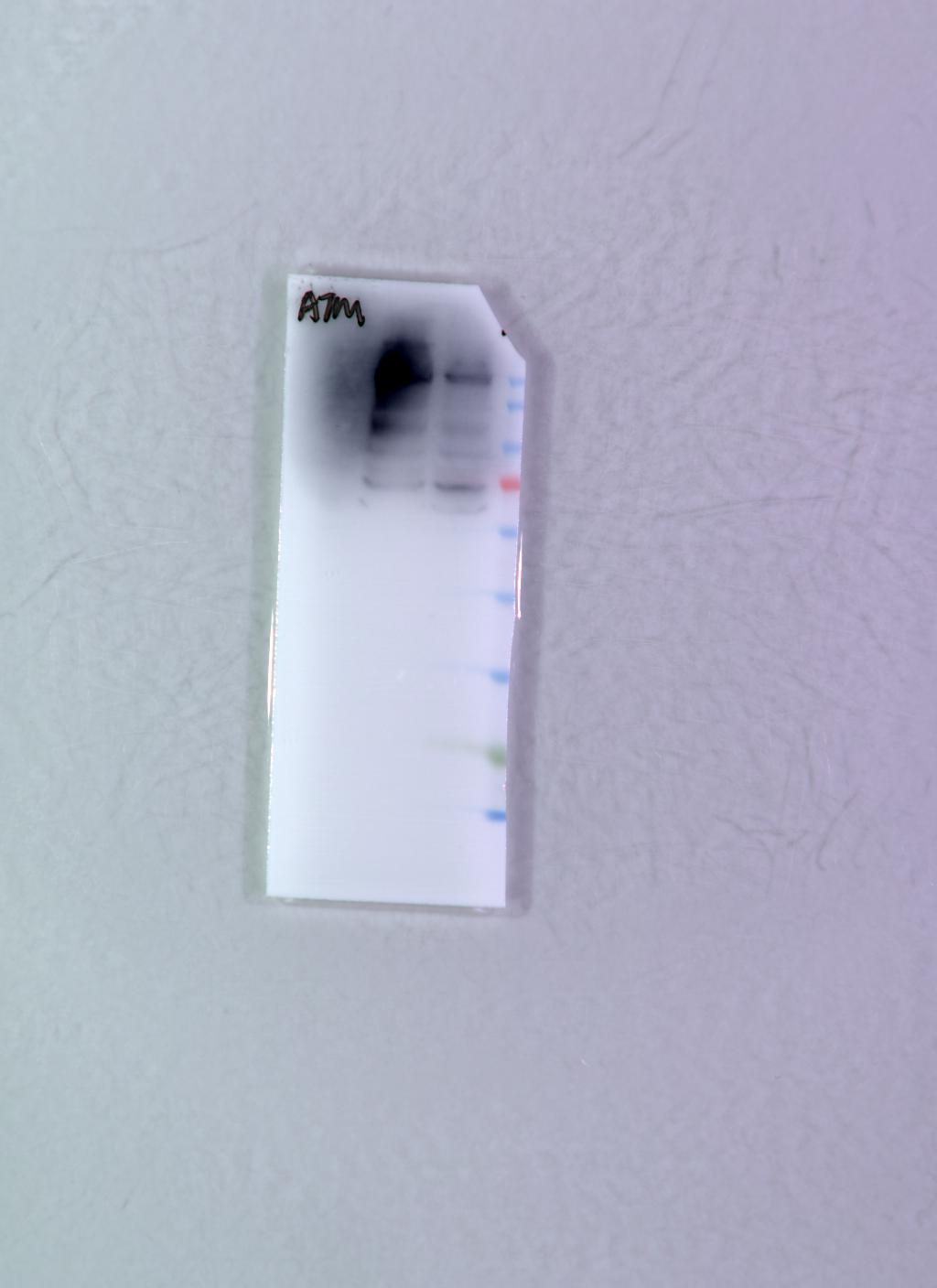

Supplement: Figure 2—source data 2. [file elife-110309-fig2-data2.zip › Figure 2-Source Data 6/ATM 1-3 2026.03.24_18.22.41_Ch+Marker.jpg]

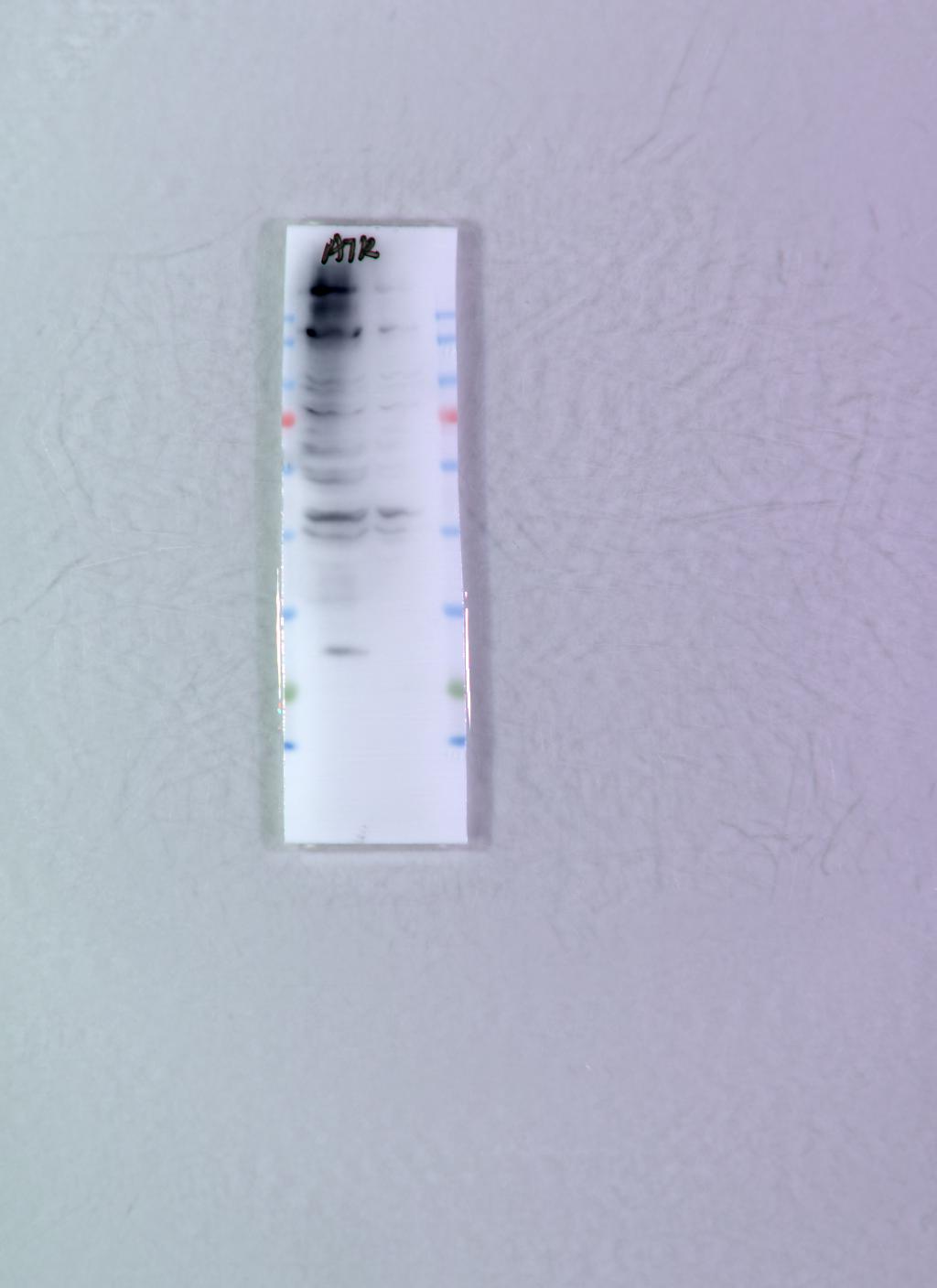

Supplement: Figure 2—source data 2. [file elife-110309-fig2-data2.zip › Figure 2-Source Data 6/ATR 5-1 2026.03.24_19.46.16_Ch+Marker.jpg]

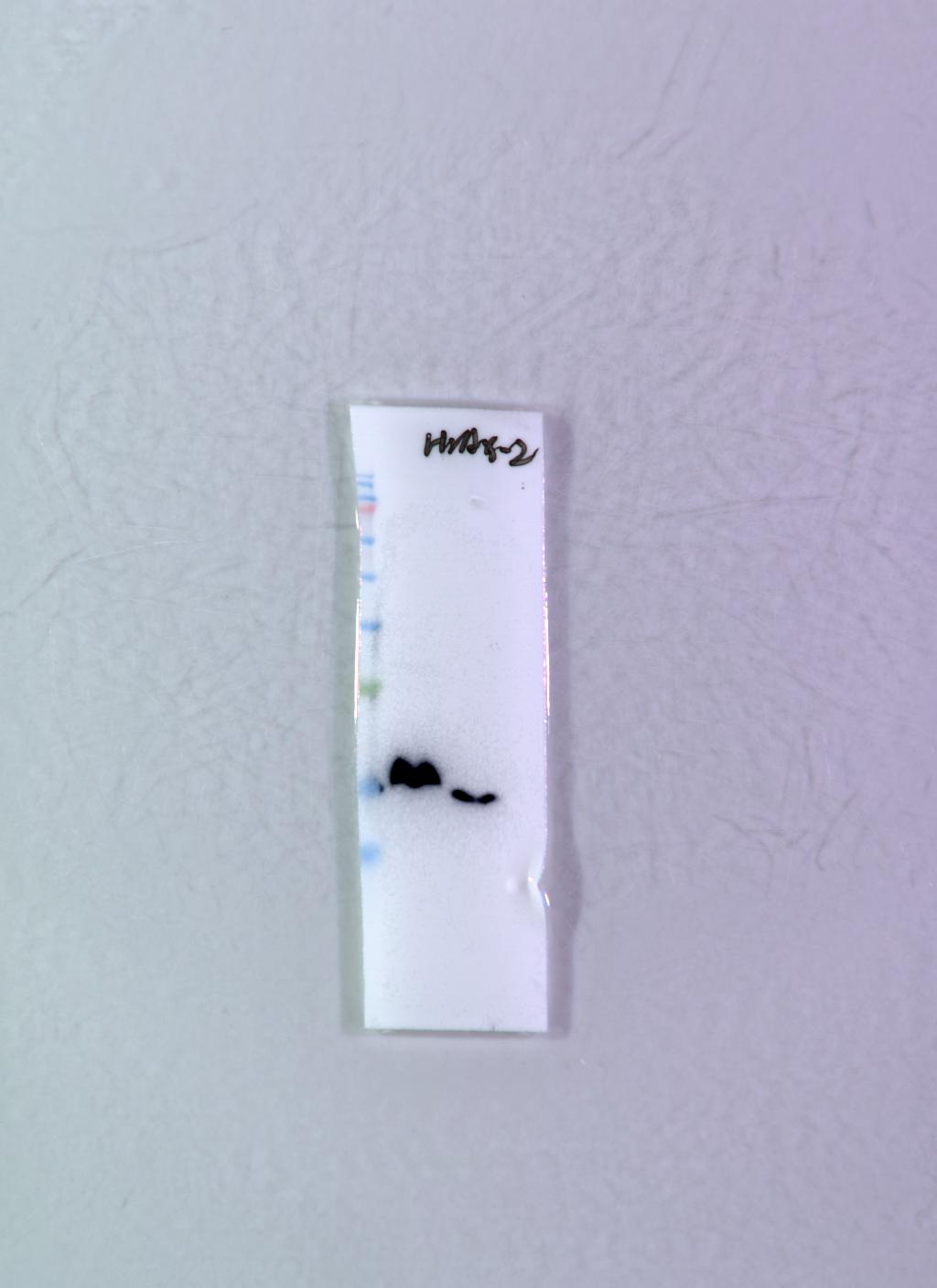

Supplement: Figure 2—source data 2. [file elife-110309-fig2-data2.zip › Figure 2-Source Data 6/H2AX 2-0 2026.03.24_19.56.21_Ch+Marker.jpg]

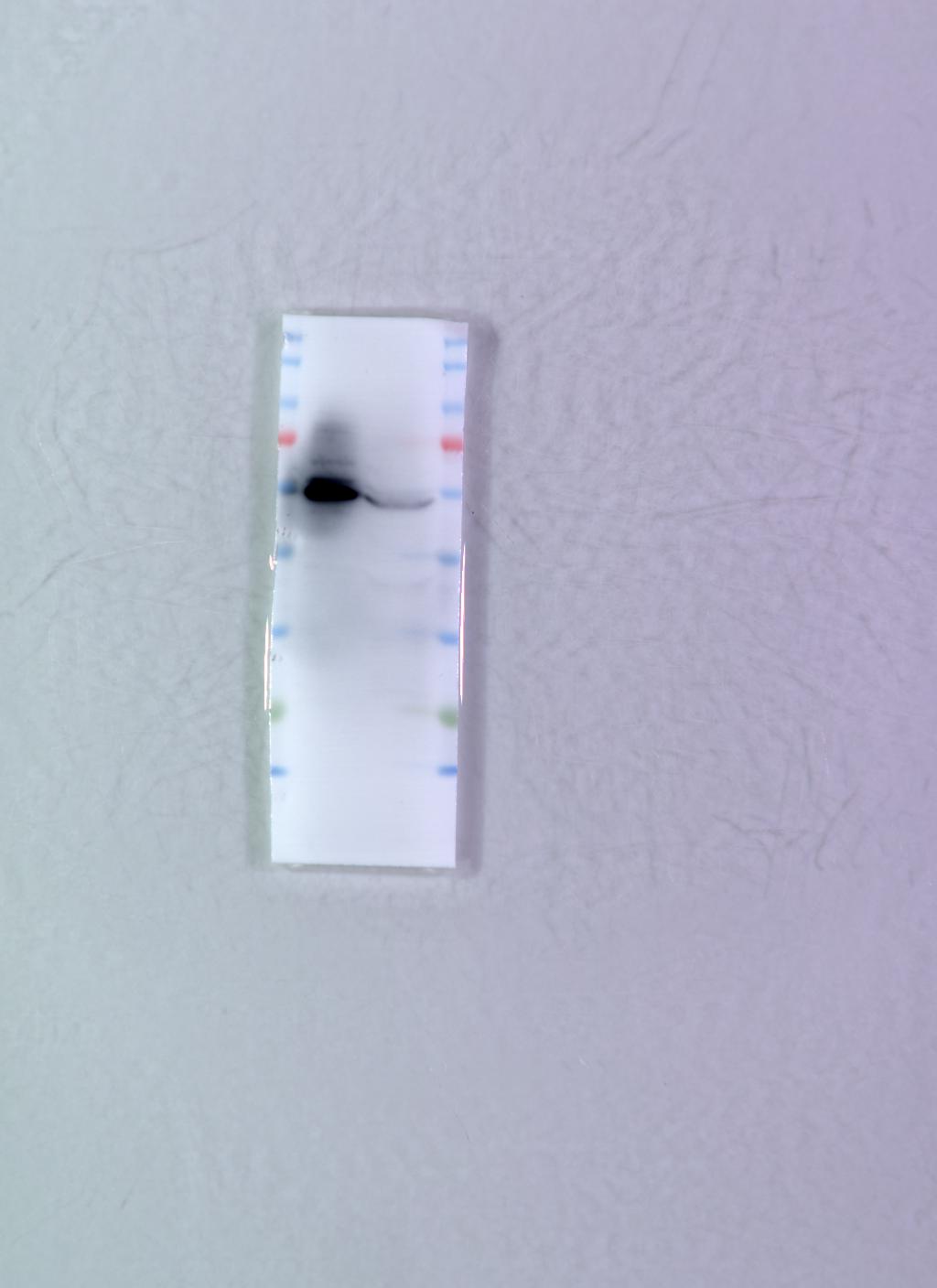

Supplement: Figure 2—source data 2. [file elife-110309-fig2-data2.zip › Figure 2-Source Data 6/HDAC1-3 2026.03.25_17.07.19_Ch+Marker.jpg]

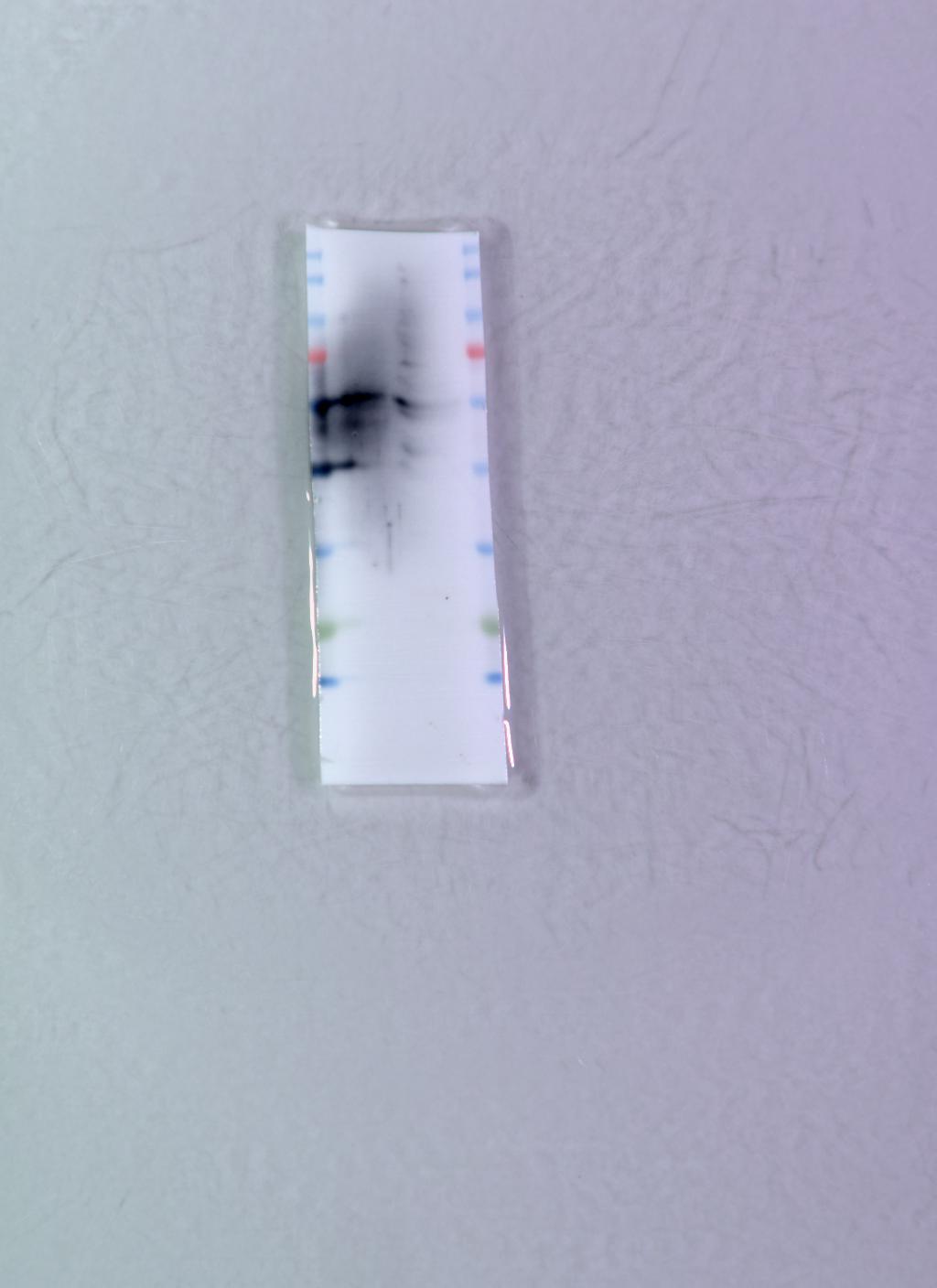

Supplement: Figure 2—source data 2. [file elife-110309-fig2-data2.zip › Figure 2-Source Data 6/HDAC2-4 2026.03.25_17.18.18_Ch+Marker.jpg]

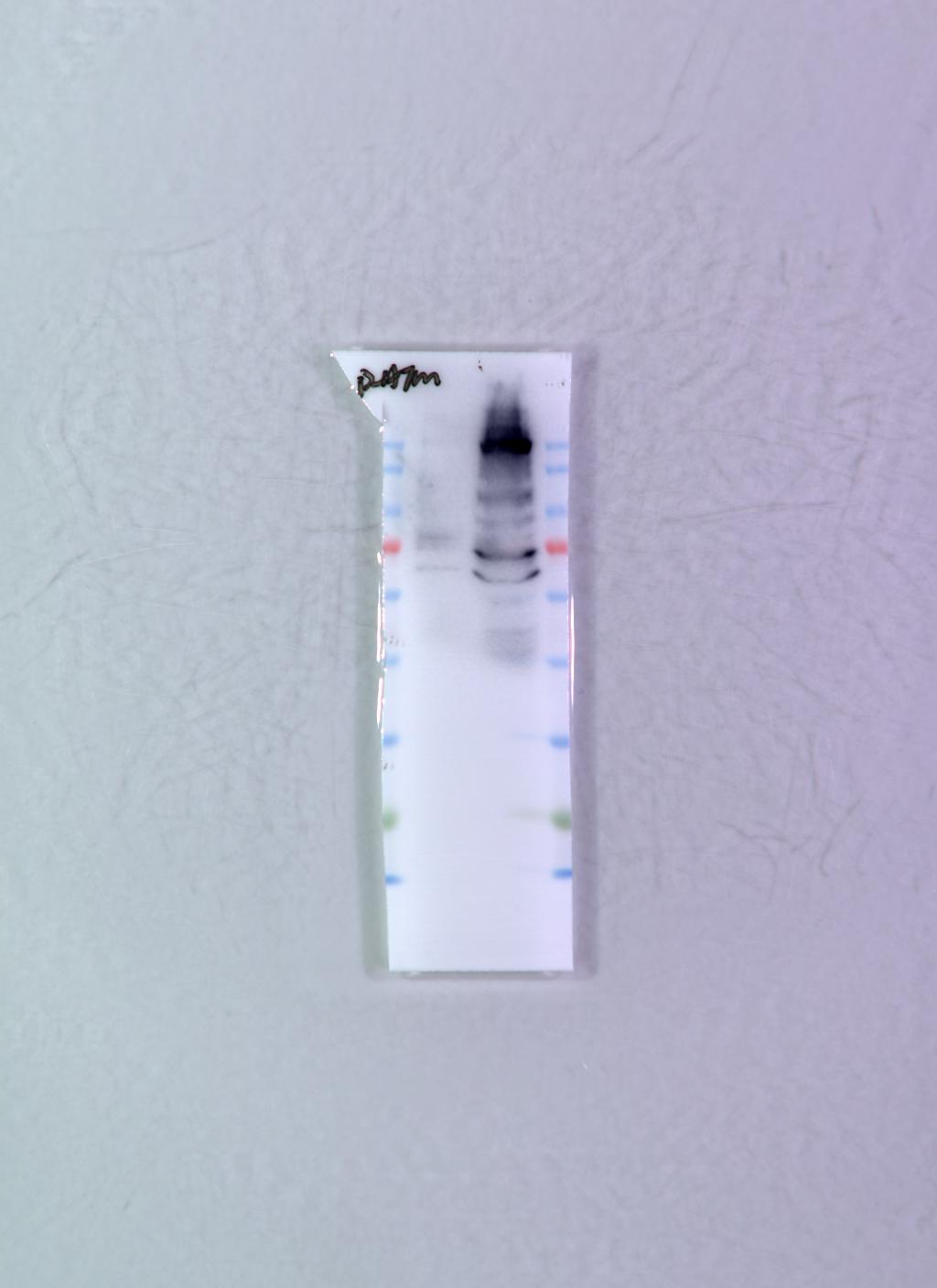

Supplement: Figure 2—source data 2. [file elife-110309-fig2-data2.zip › Figure 2-Source Data 6/P-ATM 0-4 2026.03.24_17.58.31_Ch+Marker.jpg]

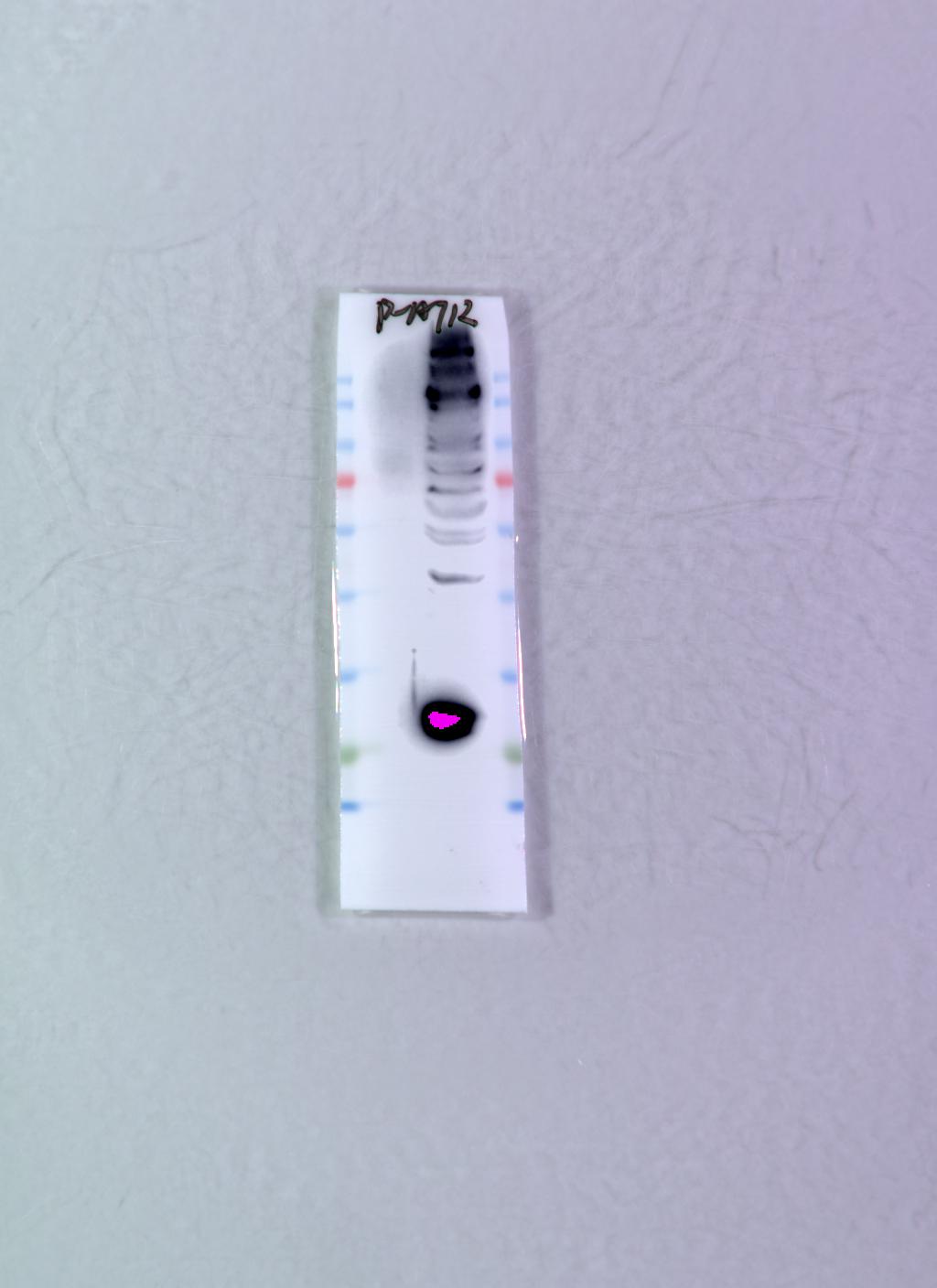

Supplement: Figure 2—source data 2. [file elife-110309-fig2-data2.zip › Figure 2-Source Data 6/P-ATR 5-2 2026.03.24_19.41.53_Ch+Marker.jpg]

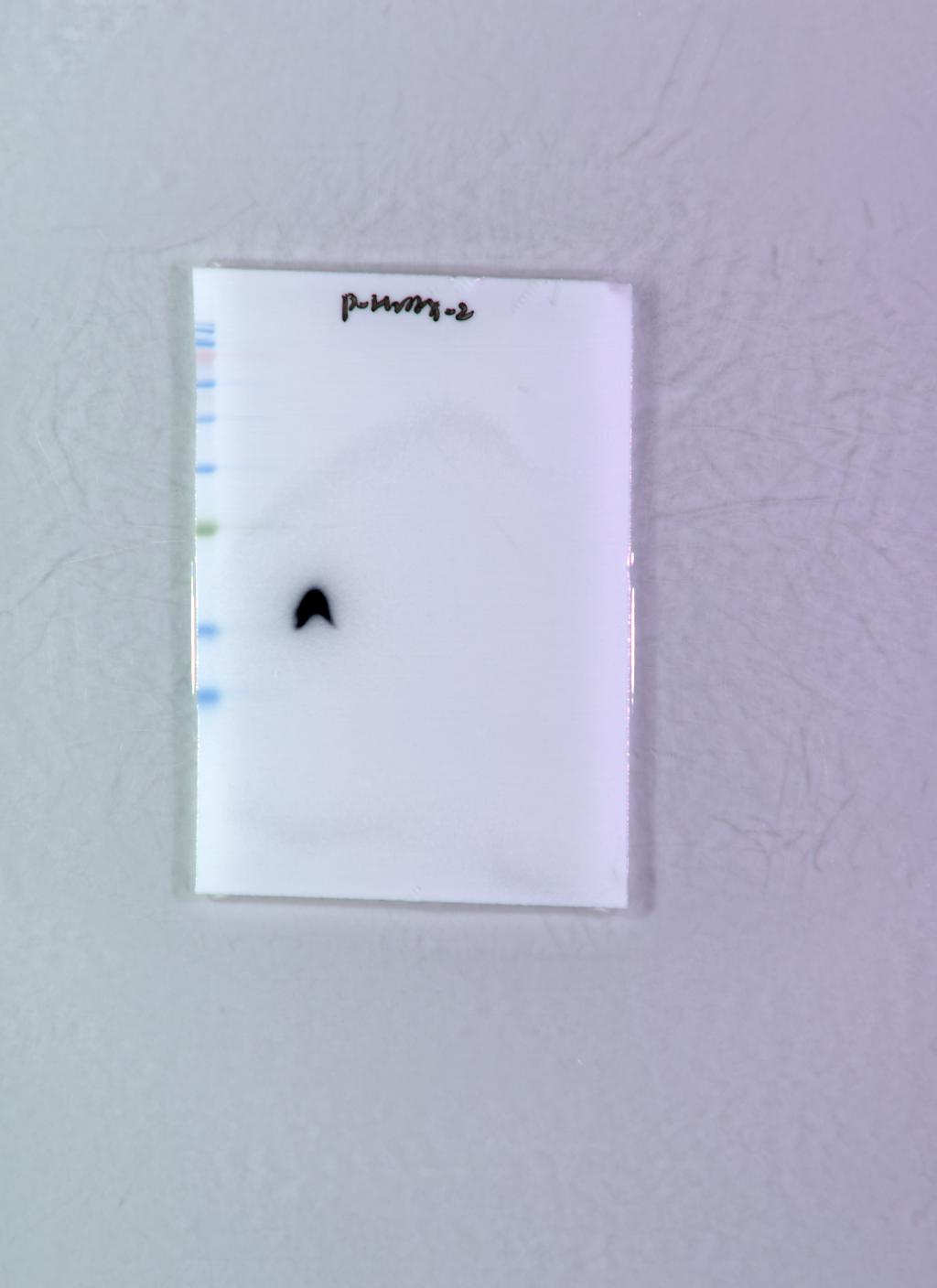

Supplement: Figure 2—source data 2. [file elife-110309-fig2-data2.zip › Figure 2-Source Data 6/P-H2AX 2-0 2026.03.24_19.53.17_Ch+Marker.jpg]

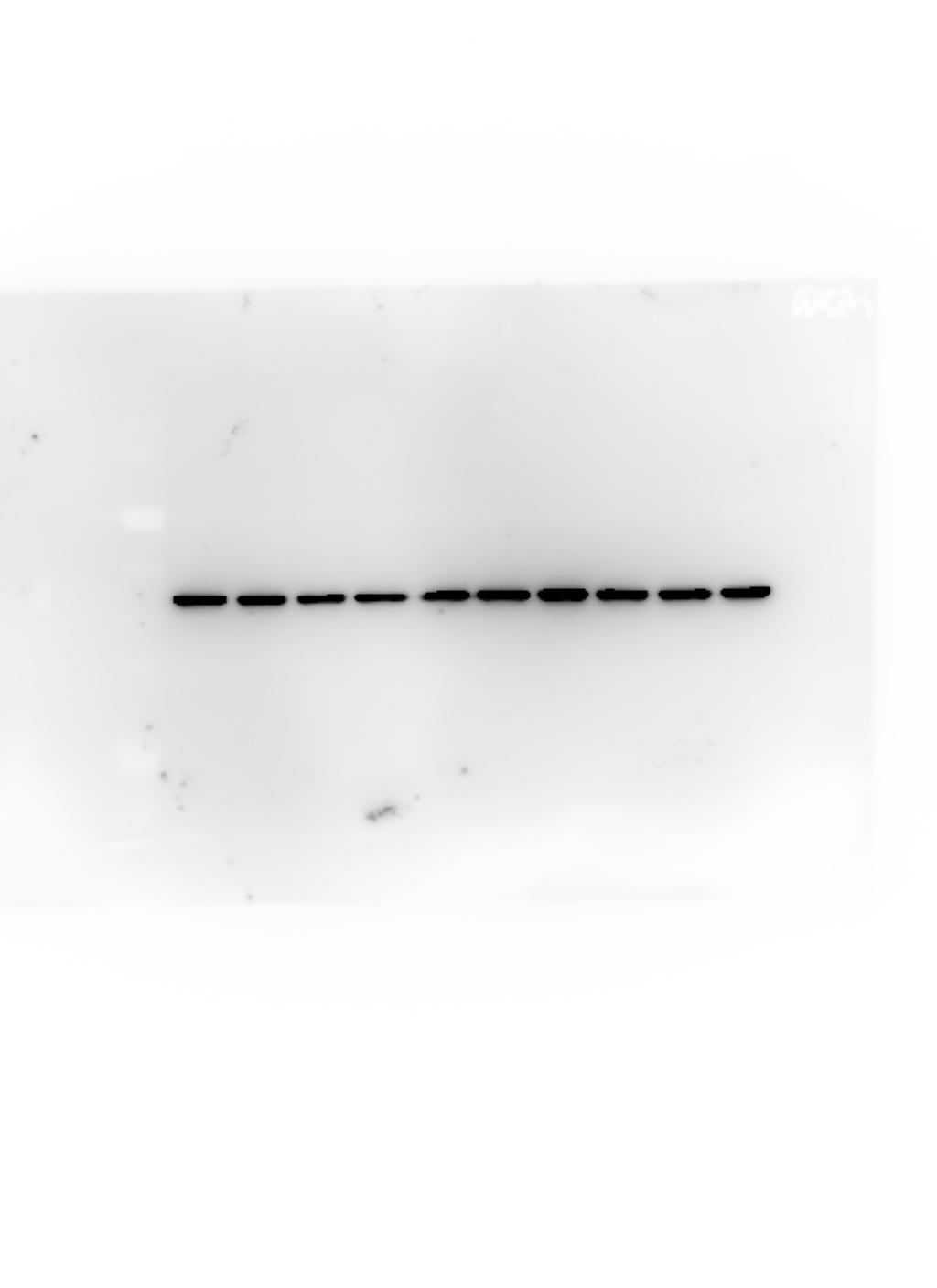

Supplement: Figure 3—source data 2. [file elife-110309-fig3-data2.zip › Figure 3-Source Data 2/ACTIN 0-2 2021.08.30_11.52.43-05_Ch.jpg]

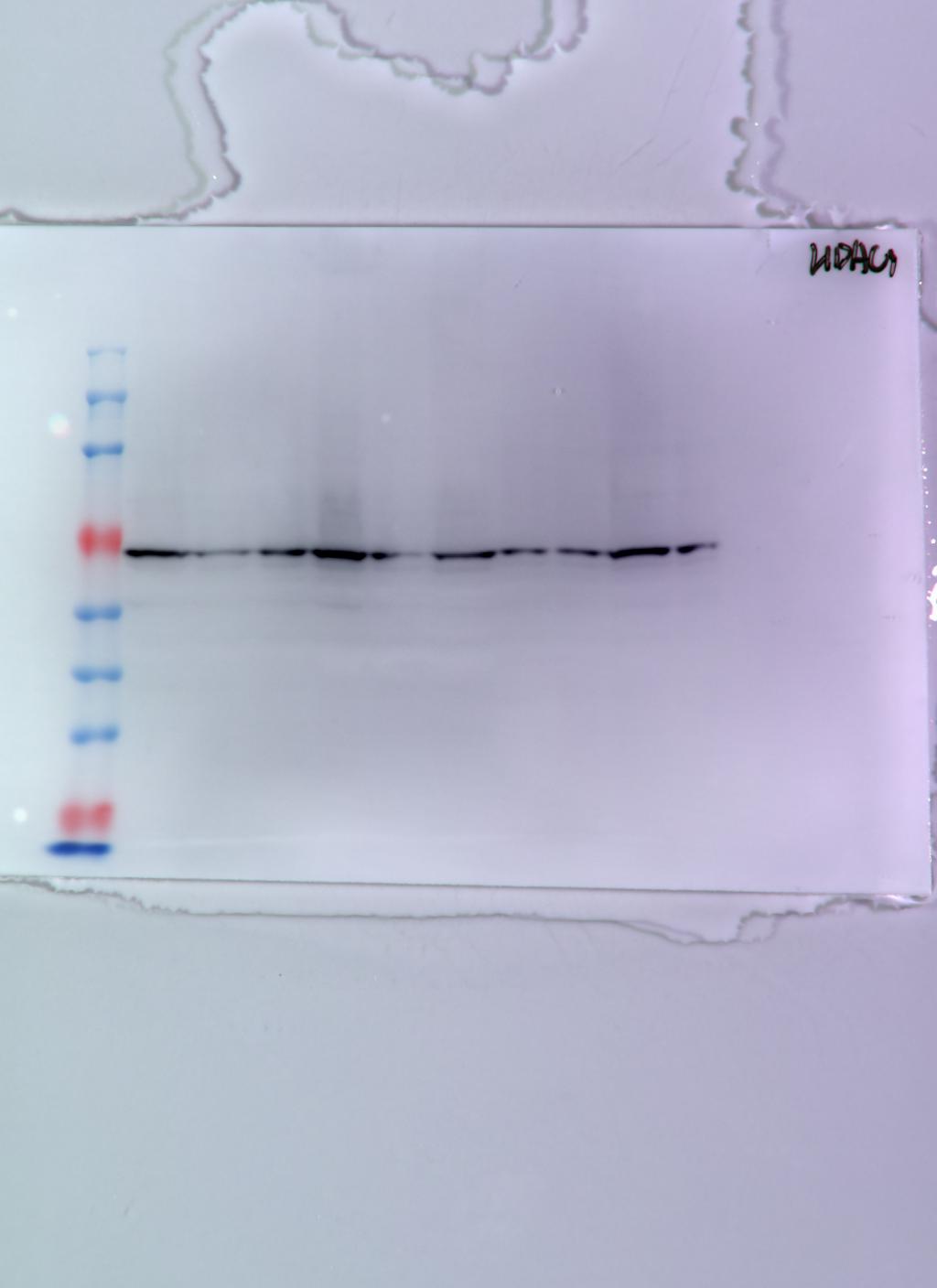

Supplement: Figure 3—source data 2. [file elife-110309-fig3-data2.zip › Figure 3-Source Data 2/HDAC1 0-5 2021.08.29_16.45.45_Ch+Marker.jpg]

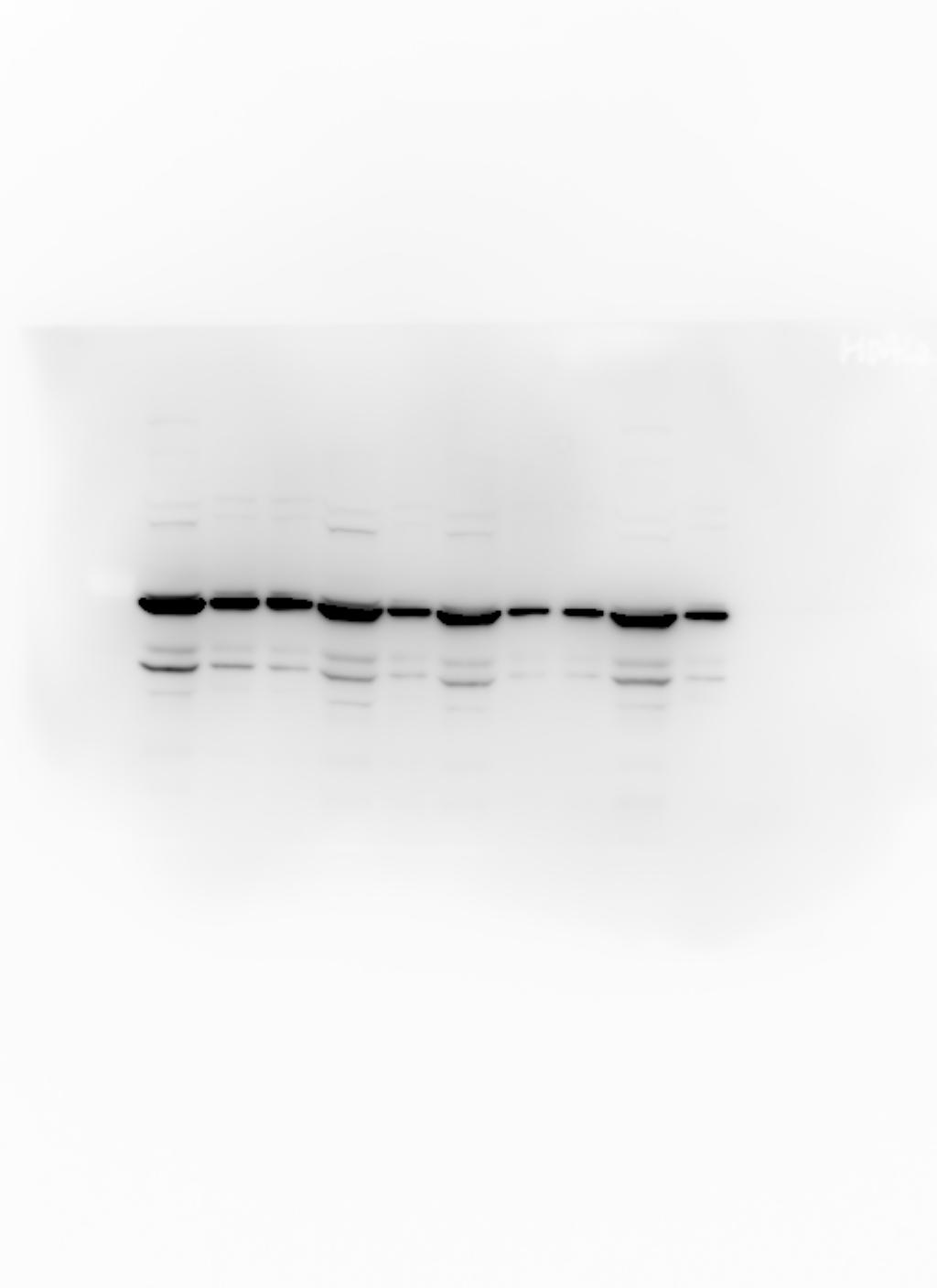

Supplement: Figure 3—source data 2. [file elife-110309-fig3-data2.zip › Figure 3-Source Data 2/HDAC2 0-9 2021.08.30_11.37.38-03_Ch.jpg]

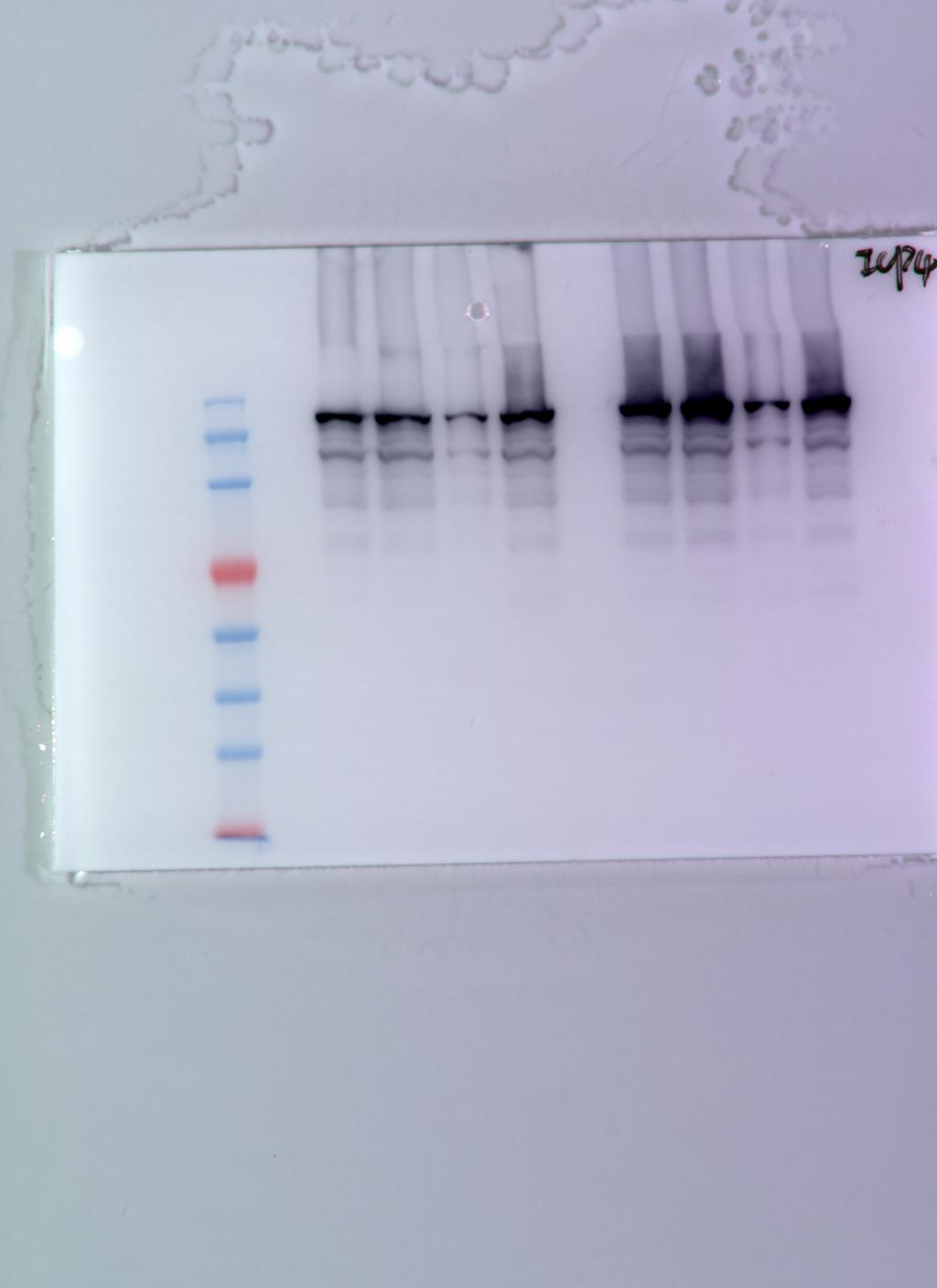

Supplement: Figure 3—source data 2. [file elife-110309-fig3-data2.zip › Figure 3-Source Data 2/ICP4 0-1 2021.08.30_11.41.22_Ch+Marker.jpg]

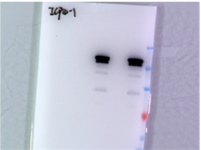

Supplement: Figure 3—source data 2. [file elife-110309-fig3-data2.zip › Figure 3-Source Data 4/ICP4.tif]

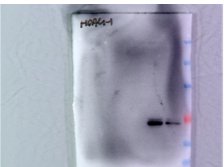

Supplement: Figure 3—source data 2. [file elife-110309-fig3-data2.zip › Figure 3-Source Data 4/IP HDAC1.tif]

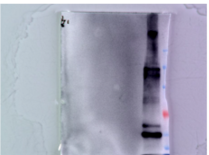

Supplement: Figure 3—source data 2. [file elife-110309-fig3-data2.zip › Figure 3-Source Data 4/IP UB.tif]

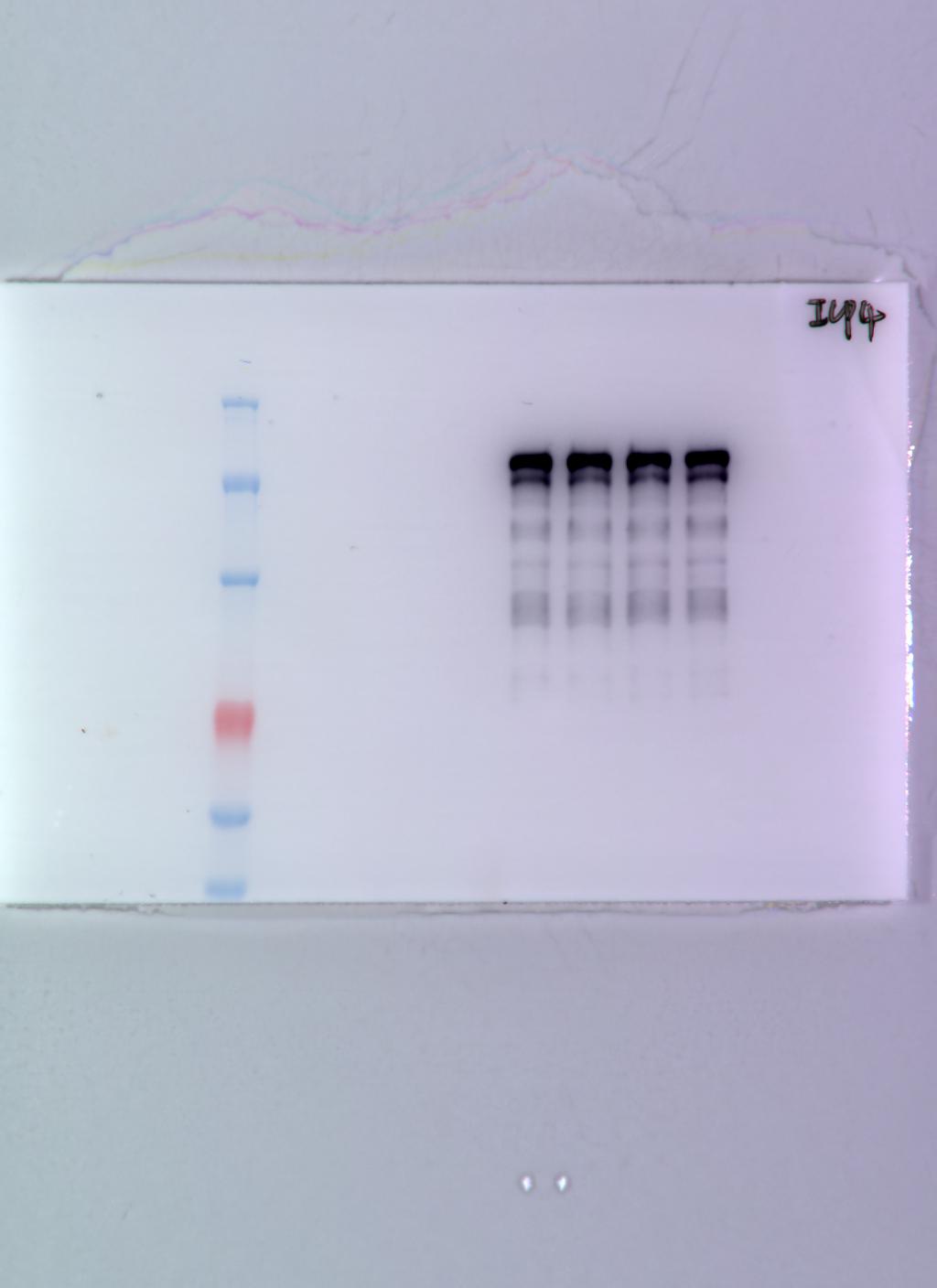

Supplement: Figure 3—source data 2. [file elife-110309-fig3-data2.zip › Figure 3-Source Data 6/INPUT ICP4 0 2021.10.18_15.20.19_Ch+Marker.jpg]
