# Supplementary figures and images for: The targeted cytosolic degradation of class I histone deacetylases is essential for efficient alphaherpesvirus replication (part 2 of 2)
Source: eLife. 2026 Jul 9;15:RP110309. doi: 10.7554/eLife.110309 (PMC13349380; doi:10.7554/eLife.110309)

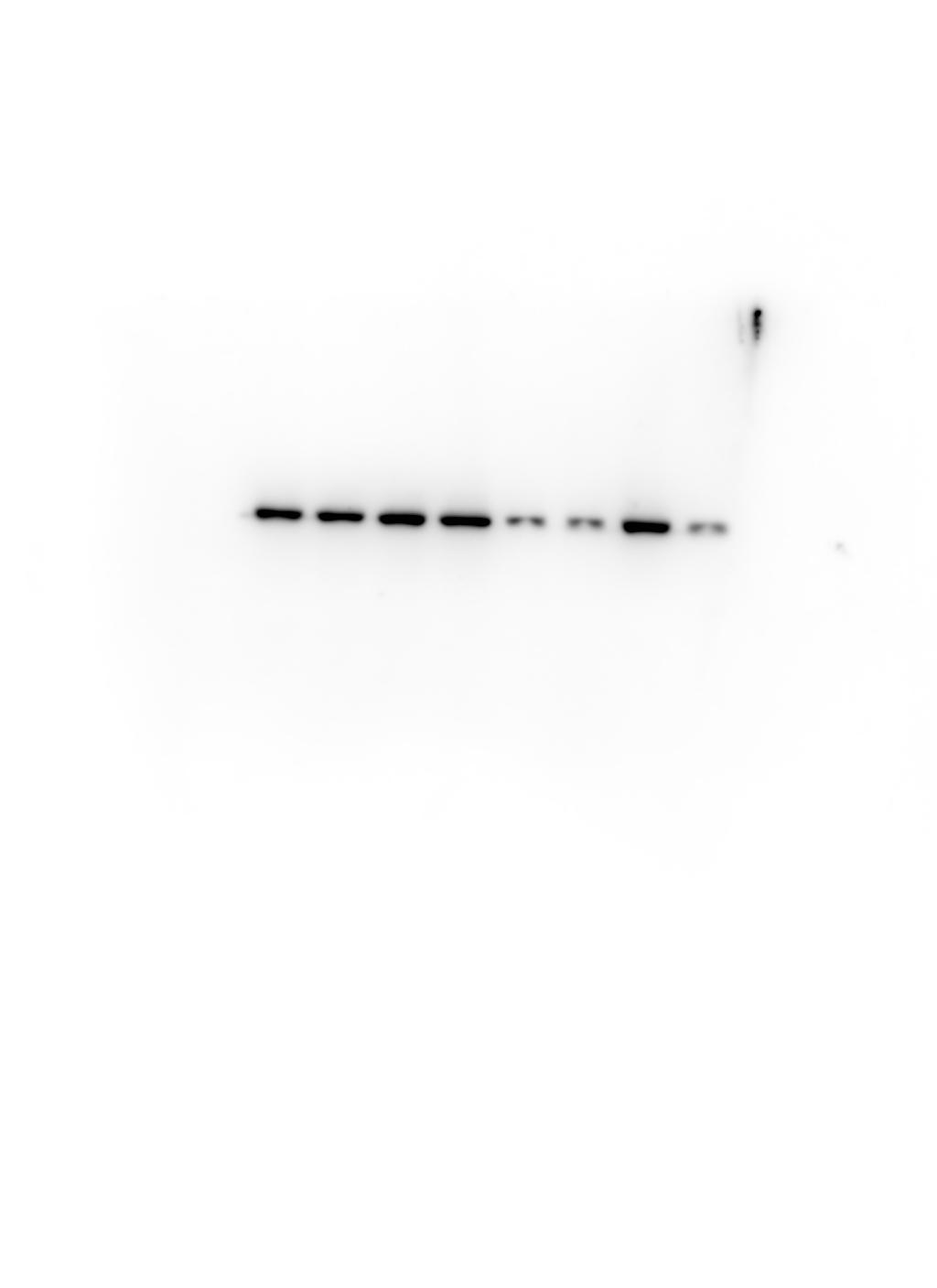

Supplement: Figure 3—source data 2. [file elife-110309-fig3-data2.zip › Figure 3-Source Data 6/IP FLAG 1-3 2021.09.23_17.21.47-05_Ch.jpg]

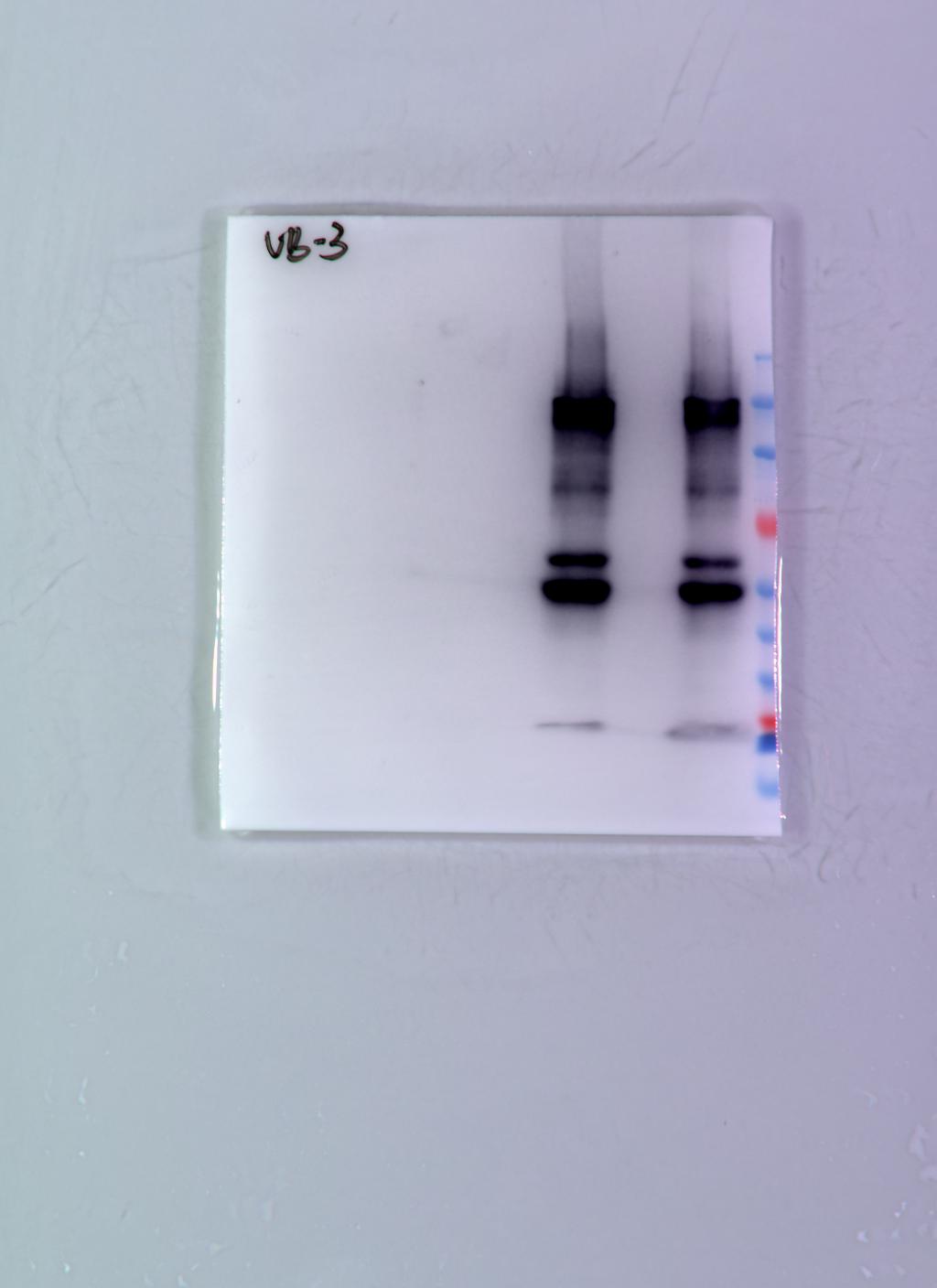

Supplement: Figure 3—source data 2. [file elife-110309-fig3-data2.zip › Figure 3-Source Data 6/UB-3 0-6 2022.04.26_17.04.55_Ch+Marker.jpg]

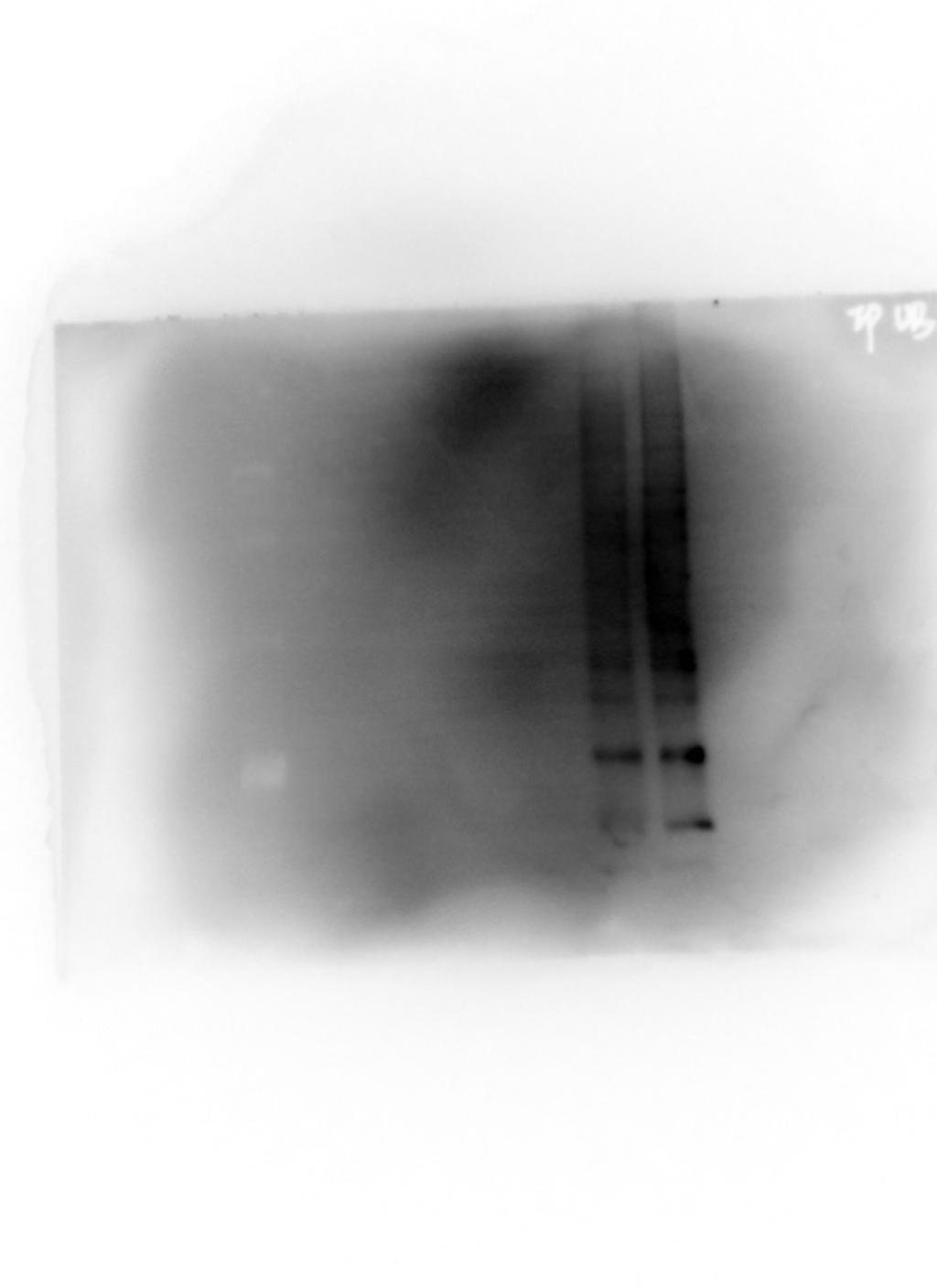

Supplement: Figure 3—source data 2. [file elife-110309-fig3-data2.zip › Figure 3-Source Data 8/hdac1 ip ha 0-6 2021.10.25_14.18.34-07_Ch.jpg]

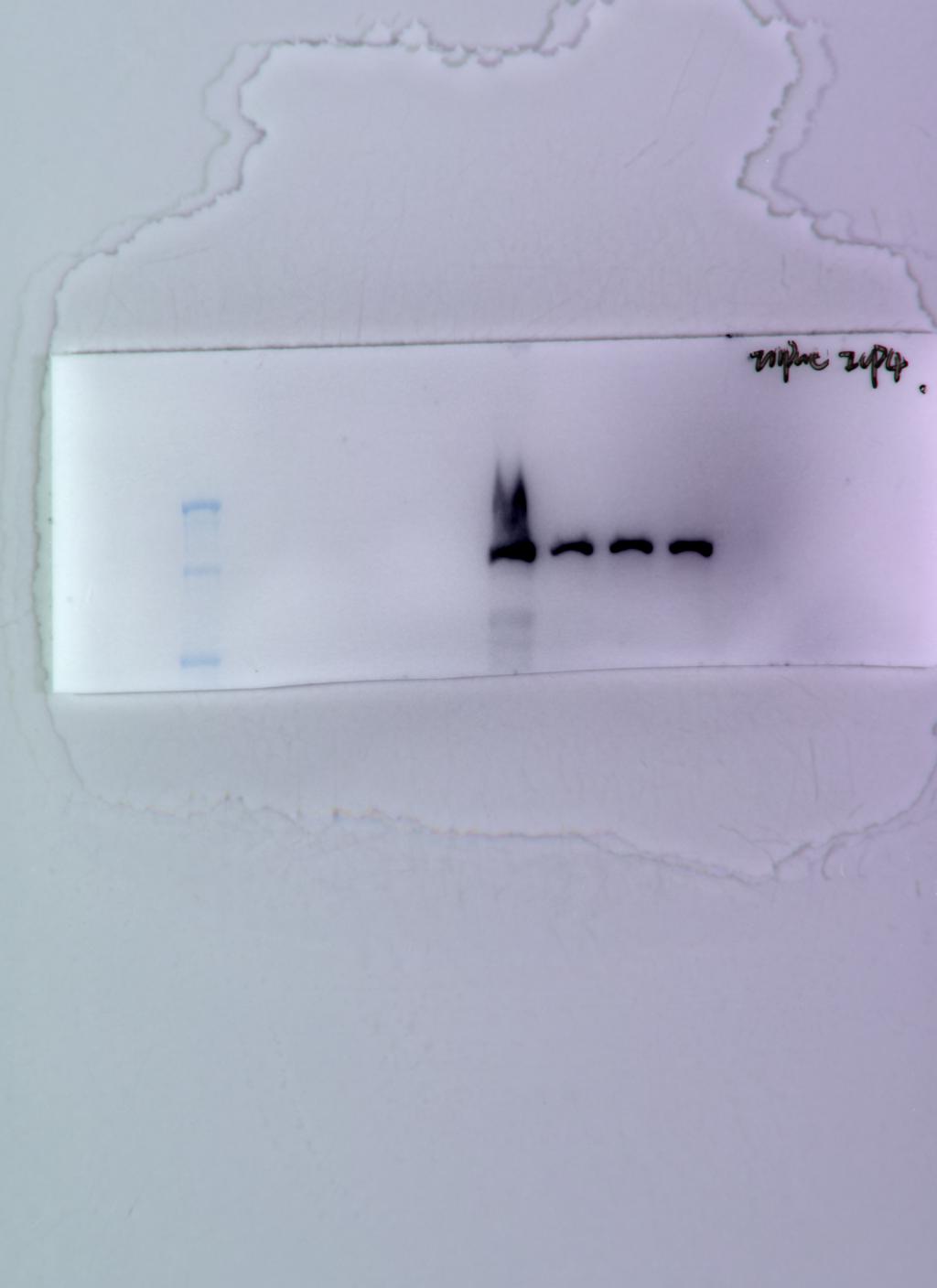

Supplement: Figure 3—source data 2. [file elife-110309-fig3-data2.zip › Figure 3-Source Data 8/icp4 0-3 2021.10.25_14.49.11_Ch+Marker.jpg]

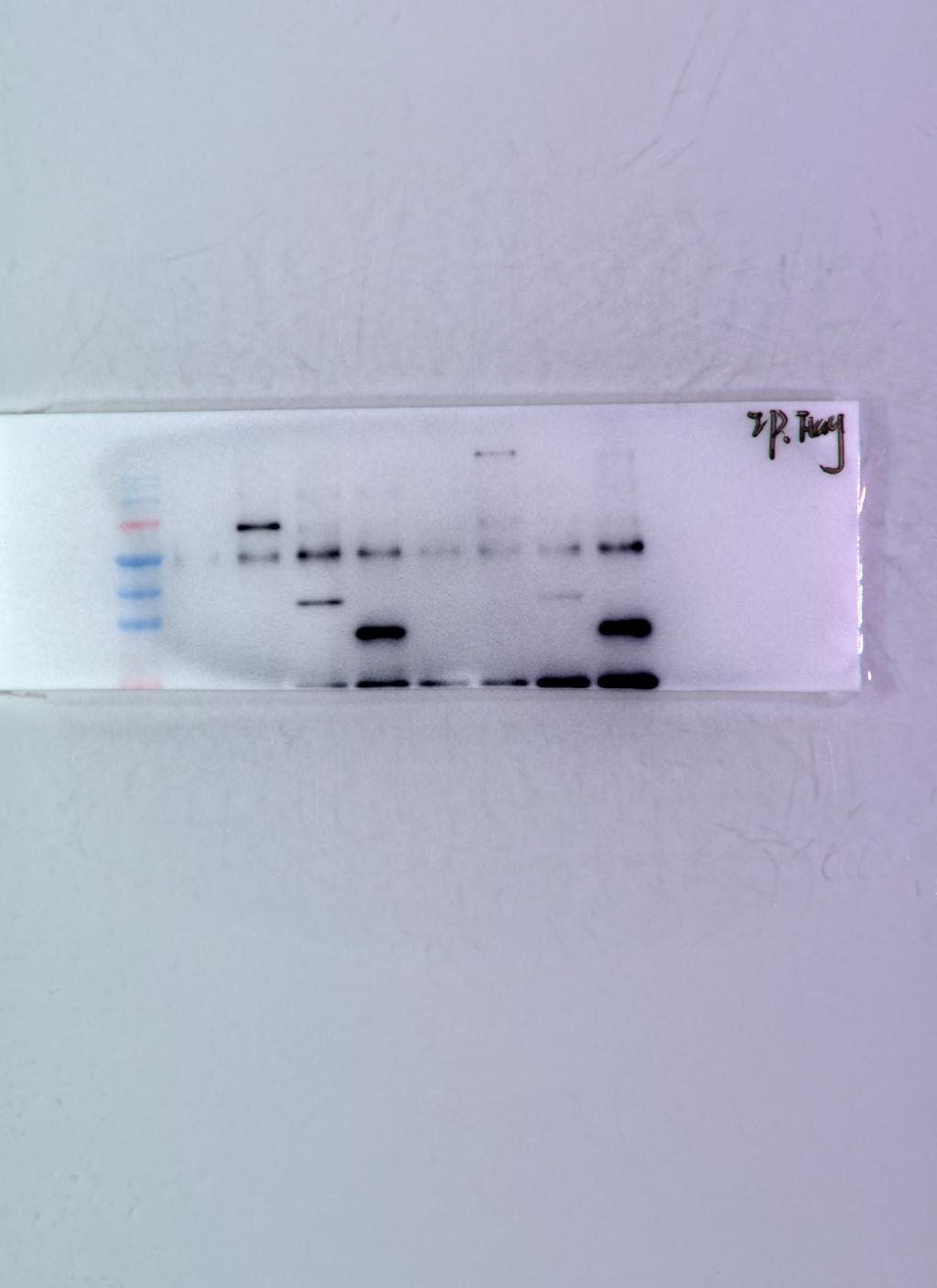

Supplement: Figure 3—source data 2. [file elife-110309-fig3-data2.zip › Figure 3-Source Data 8/ip flag 0-2 2021.10.25_15.06.47_Ch+Marker.jpg]

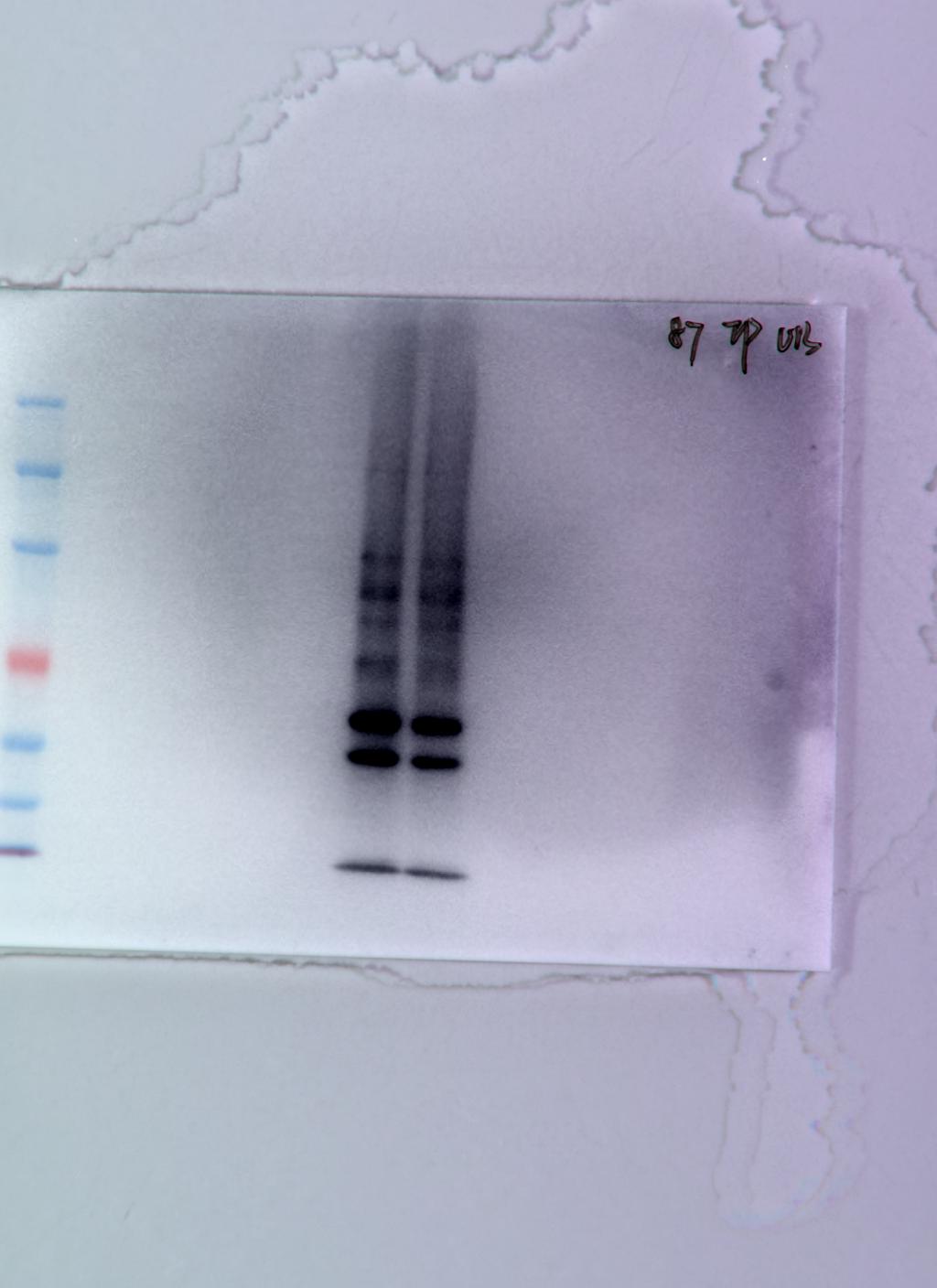

Supplement: Figure 3—source data 2. [file elife-110309-fig3-data2.zip › Figure 3-Source Data 10/87 IP UB 0-2 2021.11.29_14.20.43_Ch+Marker.jpg]

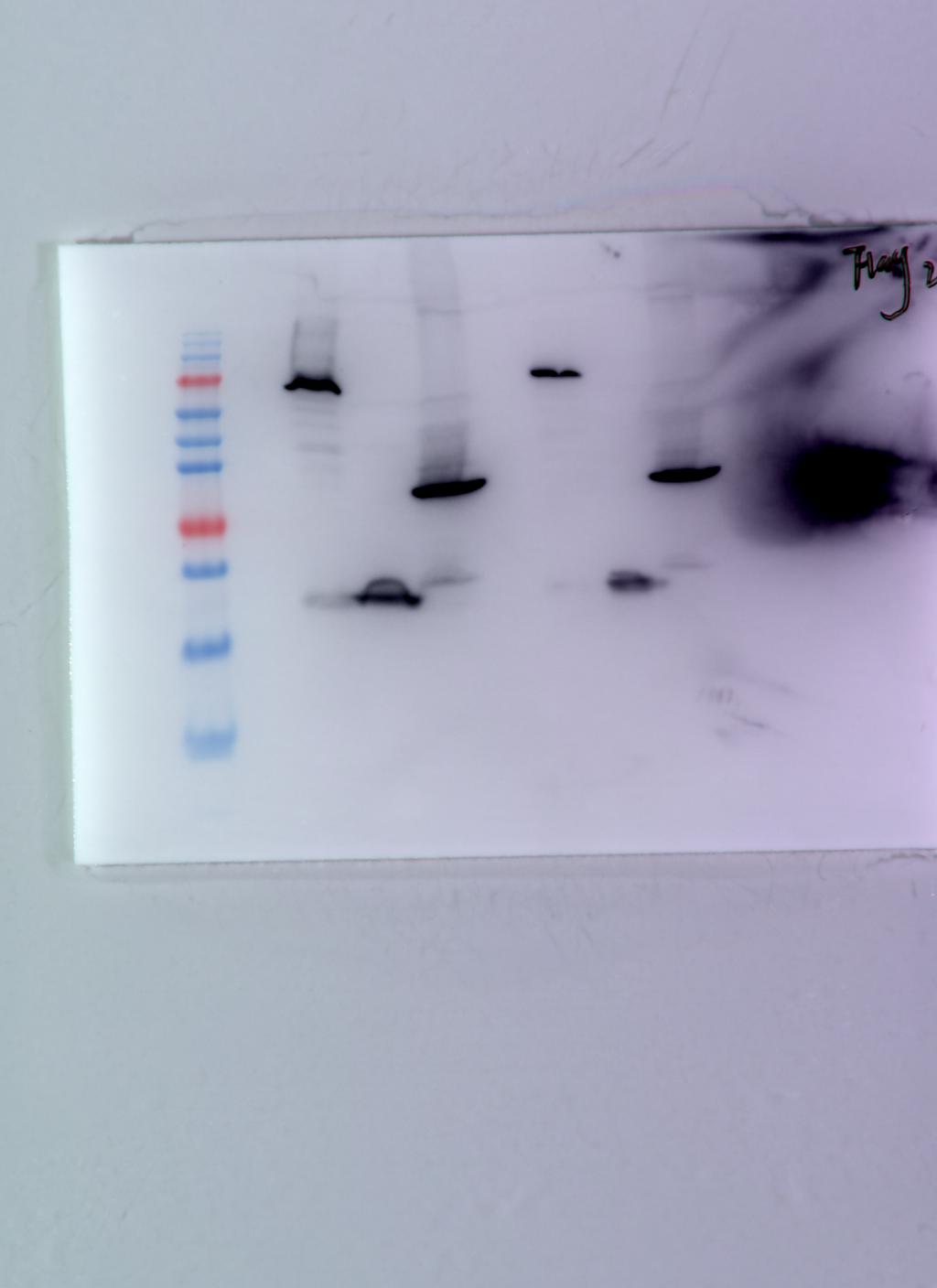

Supplement: Figure 3—source data 2. [file elife-110309-fig3-data2.zip › Figure 3-Source Data 10/flag 1-8 2022.03.11_11.06.01_Ch+Marker.jpg]

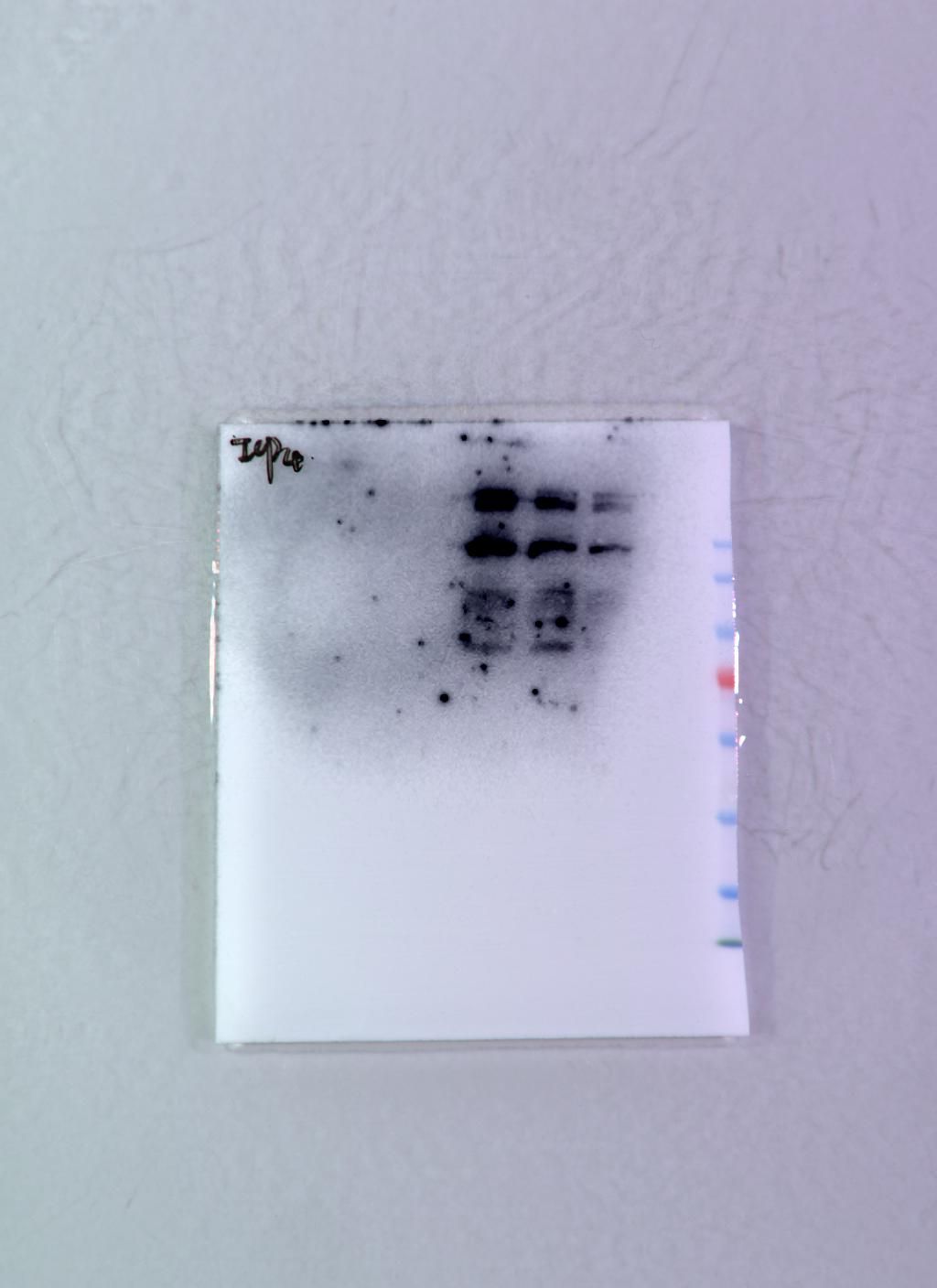

Supplement: Figure 3—source data 2. [file elife-110309-fig3-data2.zip › Figure 3-Source Data 10/ICP4 3-1 2026.03.31_18.06.51_Ch+Marker.jpg]

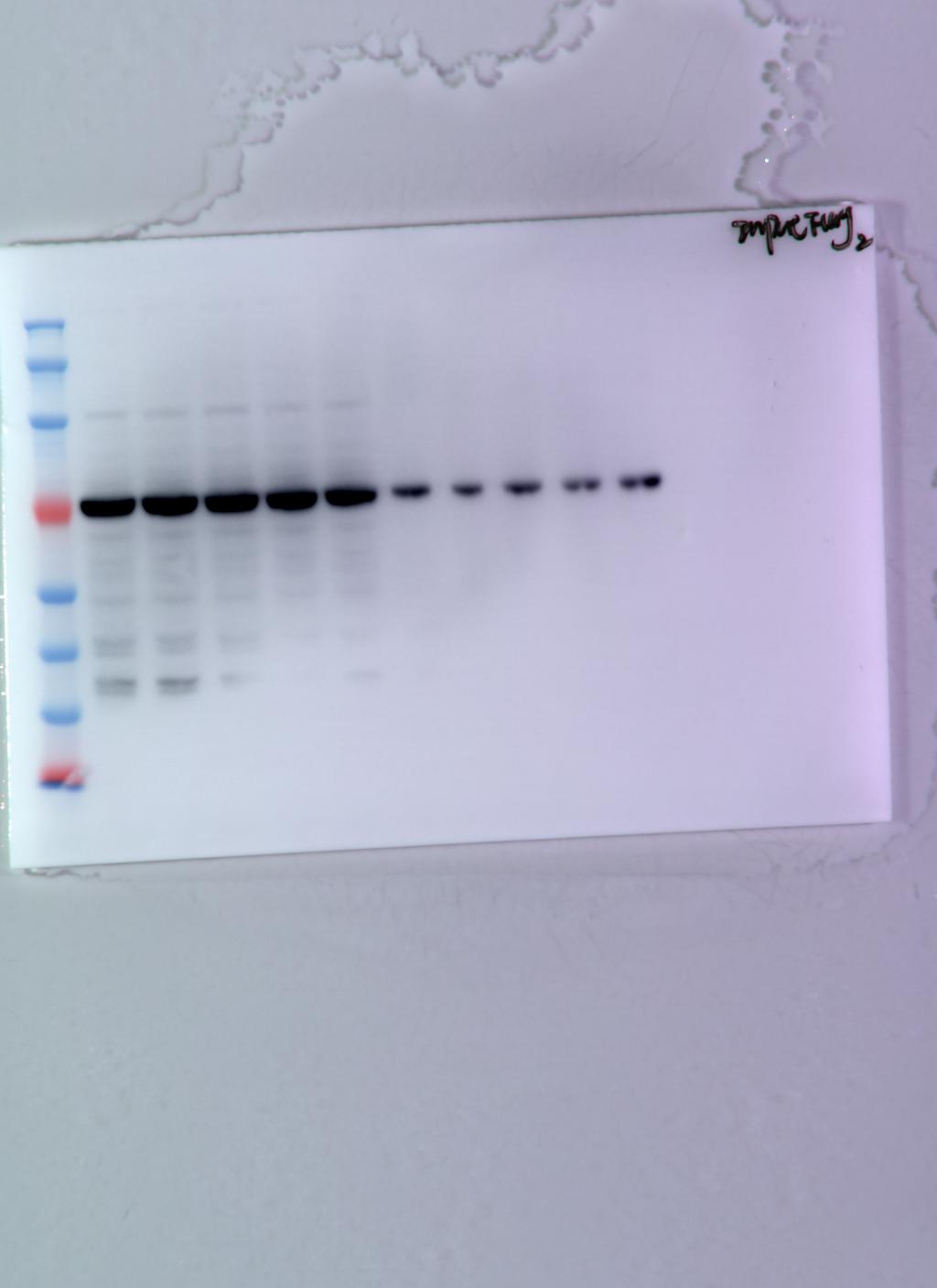

Supplement: Figure 3—source data 2. [file elife-110309-fig3-data2.zip › Figure 3-Source Data 12/input flag 0 1-4 2022.01.06_17.22.09_Ch+Marker.jpg]

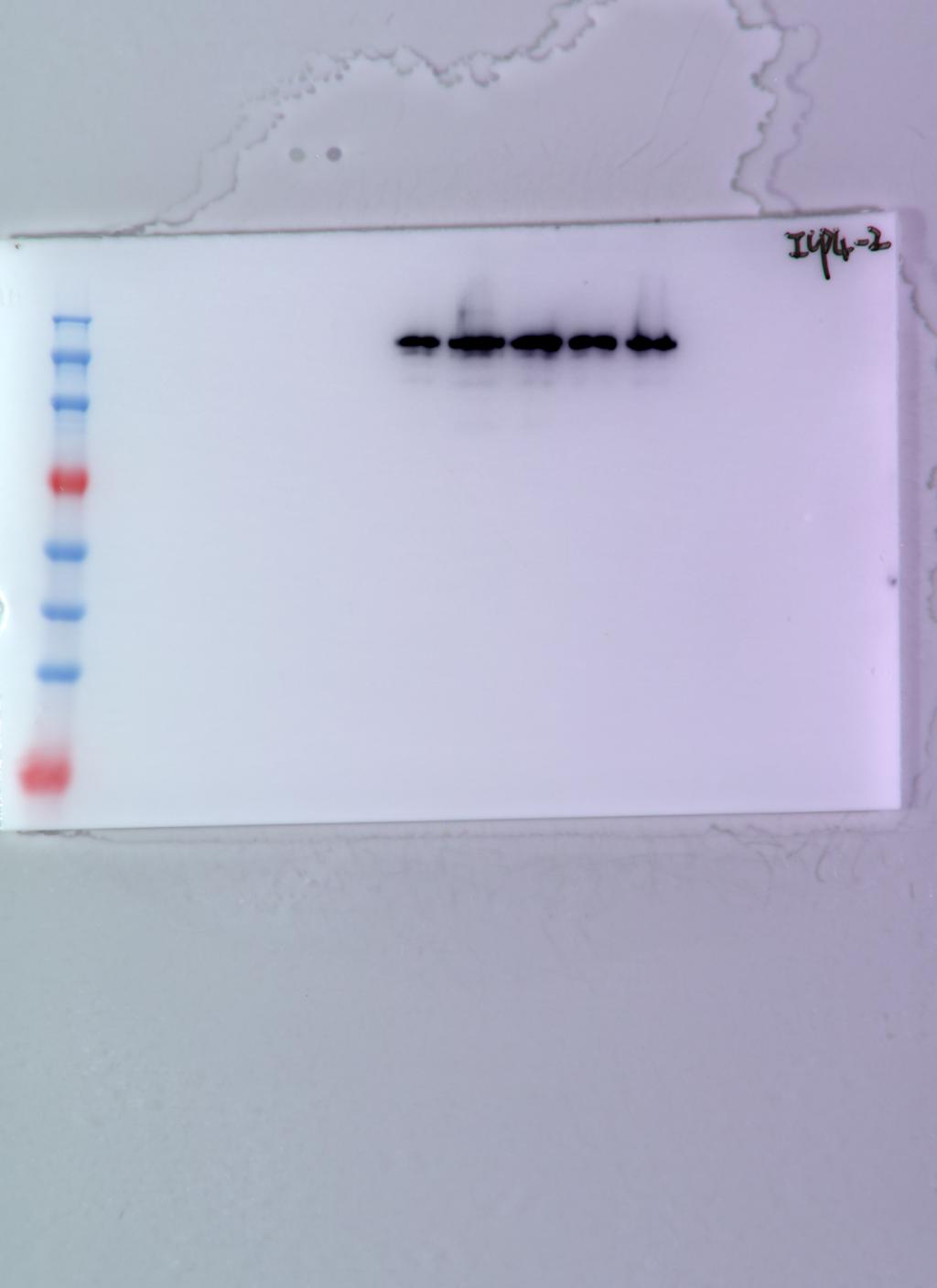

Supplement: Figure 3—source data 2. [file elife-110309-fig3-data2.zip › Figure 3-Source Data 12/INPUT ICP4-1 0 2022.01.03_00.18.56_Ch+Marker.jpg]

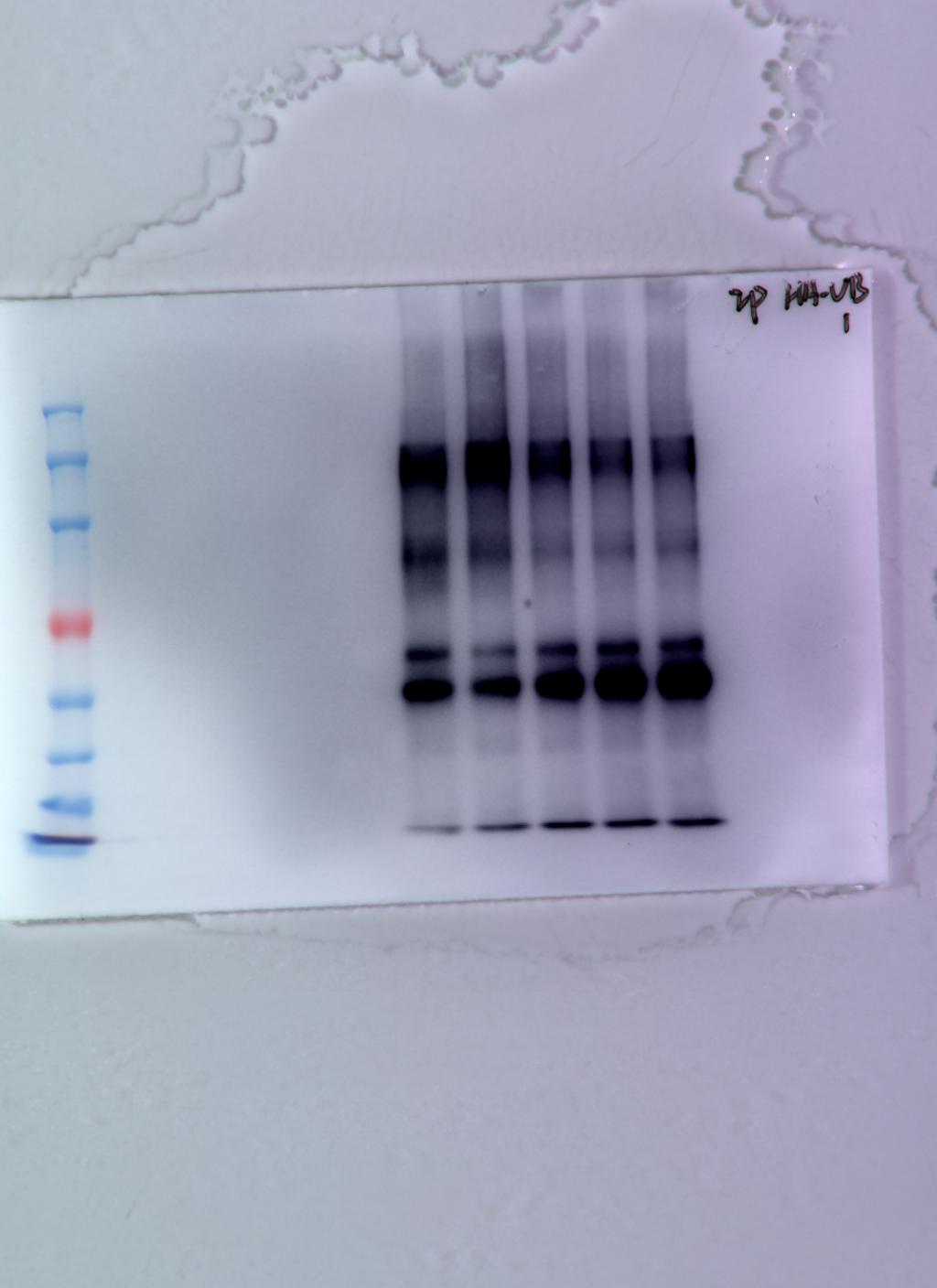

Supplement: Figure 3—source data 2. [file elife-110309-fig3-data2.zip › Figure 3-Source Data 12/IP HA UB 1 0-2 2022.01.03_21.49.33_Ch+Marker.jpg]

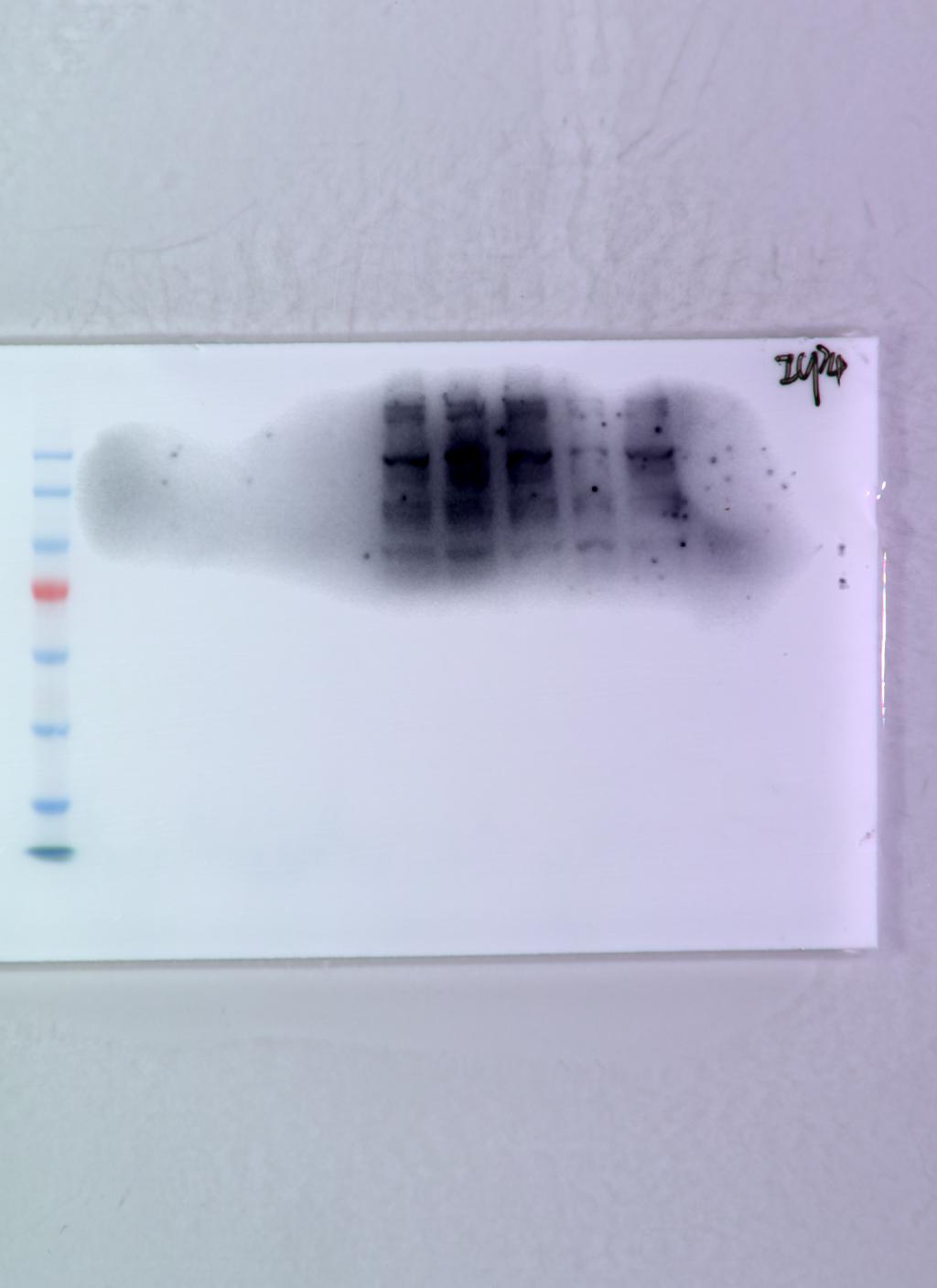

Supplement: Figure 3—source data 2. [file elife-110309-fig3-data2.zip › Figure 3-Source Data 14/ICP4 0-5 2026.03.31_17.51.47_Ch+Marker.jpg]

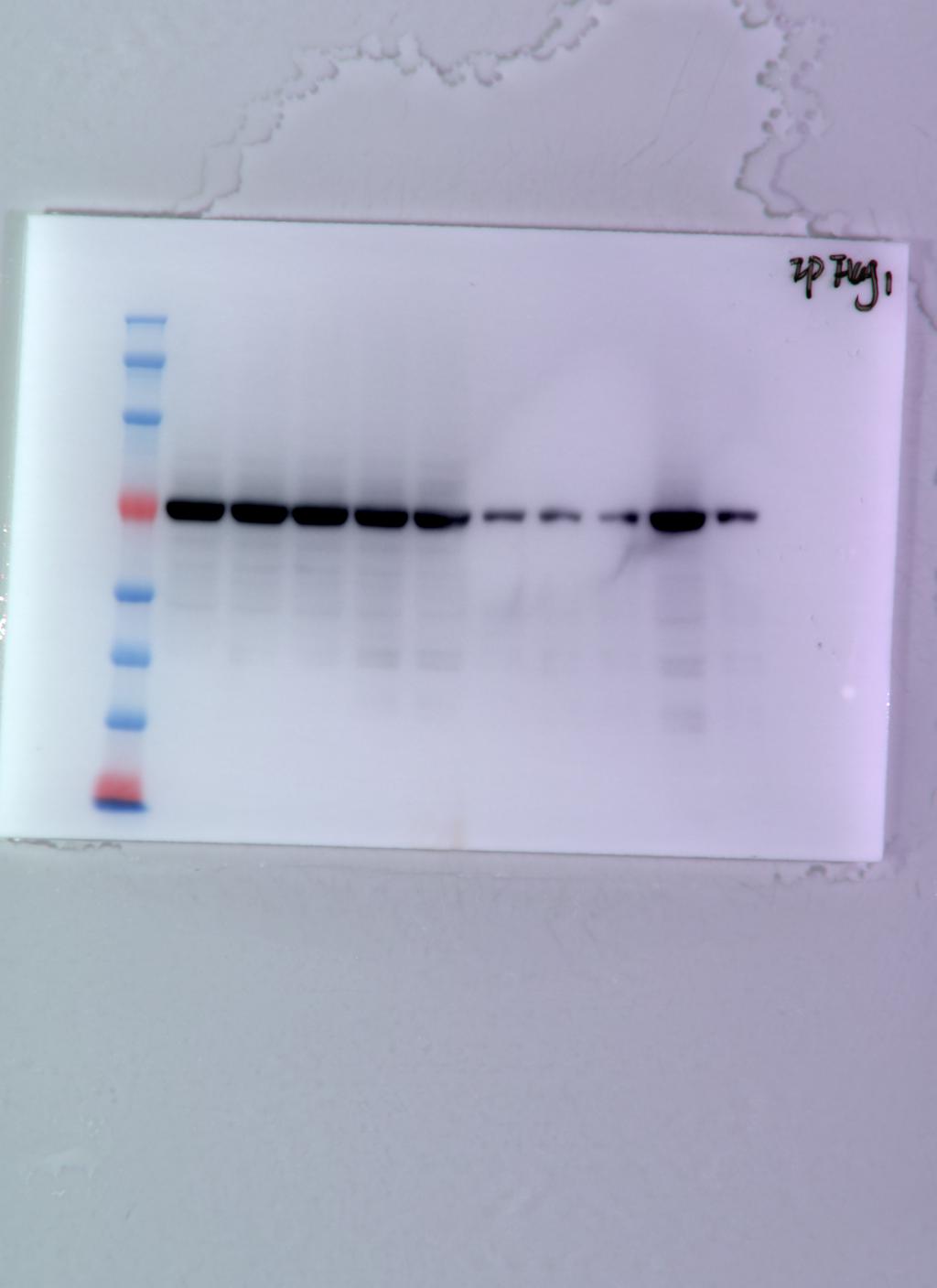

Supplement: Figure 3—source data 2. [file elife-110309-fig3-data2.zip › Figure 3-Source Data 14/ip flag 0 1-7 2022.01.06_17.01.26_Ch+Marker.jpg]

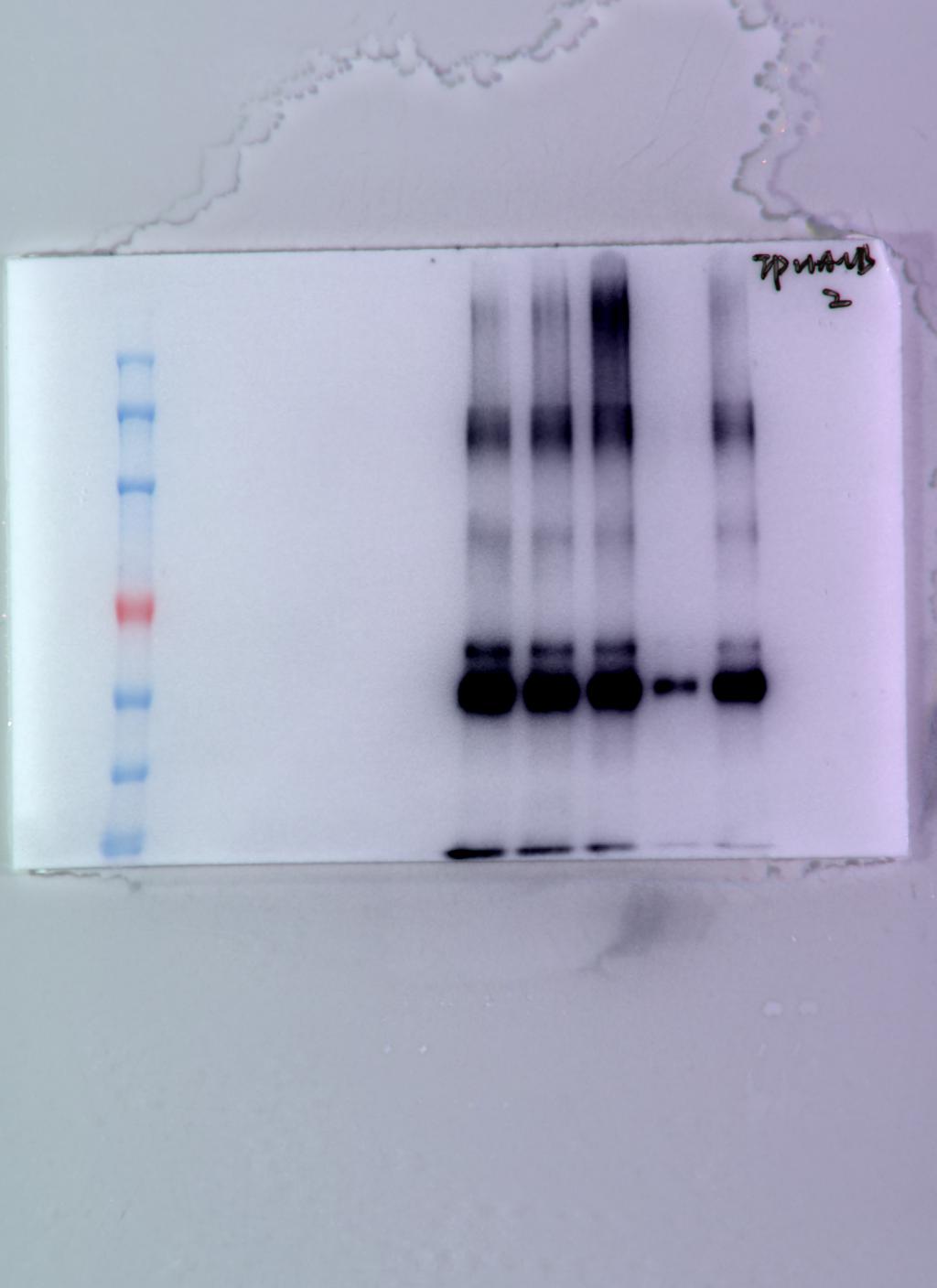

Supplement: Figure 3—source data 2. [file elife-110309-fig3-data2.zip › Figure 3-Source Data 14/IP HA UB 2 0-1 2022.01.03_21.54.37_Ch+Marker.jpg]

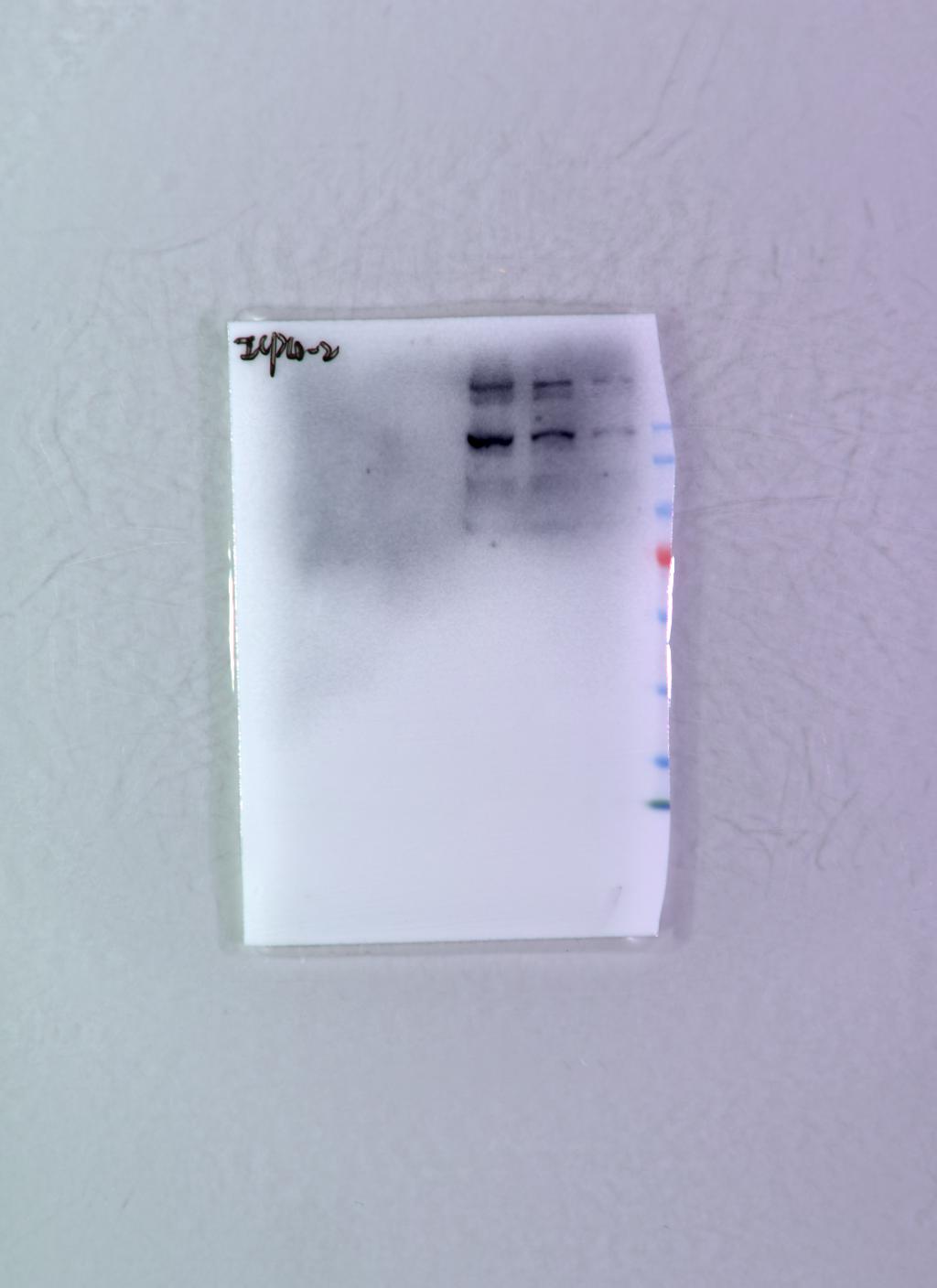

Supplement: Figure 3—source data 2. [file elife-110309-fig3-data2.zip › Figure 3-Source Data 16/ICP4 2-2 2026.03.31_18.02.41_Ch+Marker.jpg]

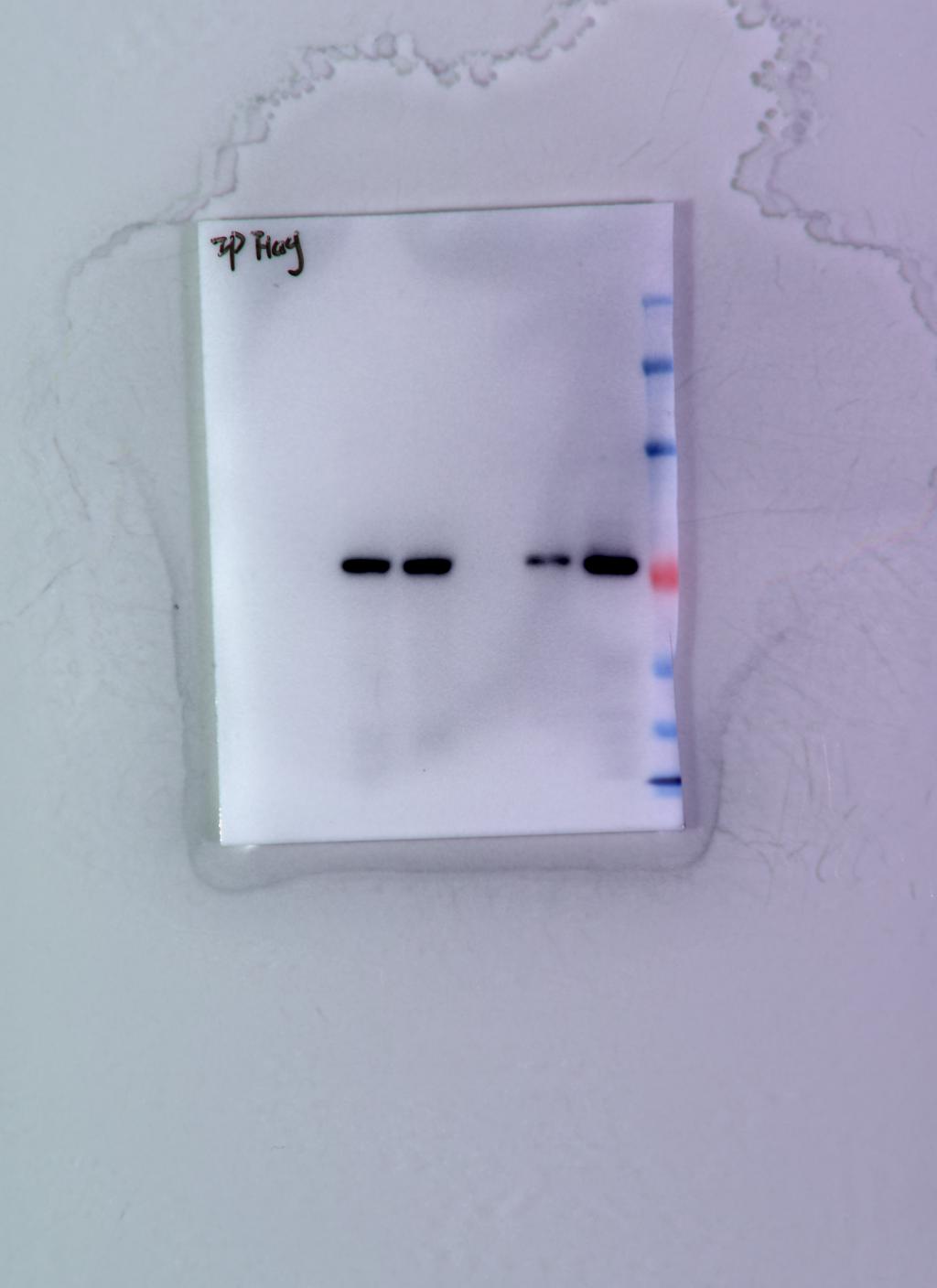

Supplement: Figure 3—source data 2. [file elife-110309-fig3-data2.zip › Figure 3-Source Data 16/IP FIAG 0-3 2022.01.09_17.12.29_Ch+Marker.jpg]

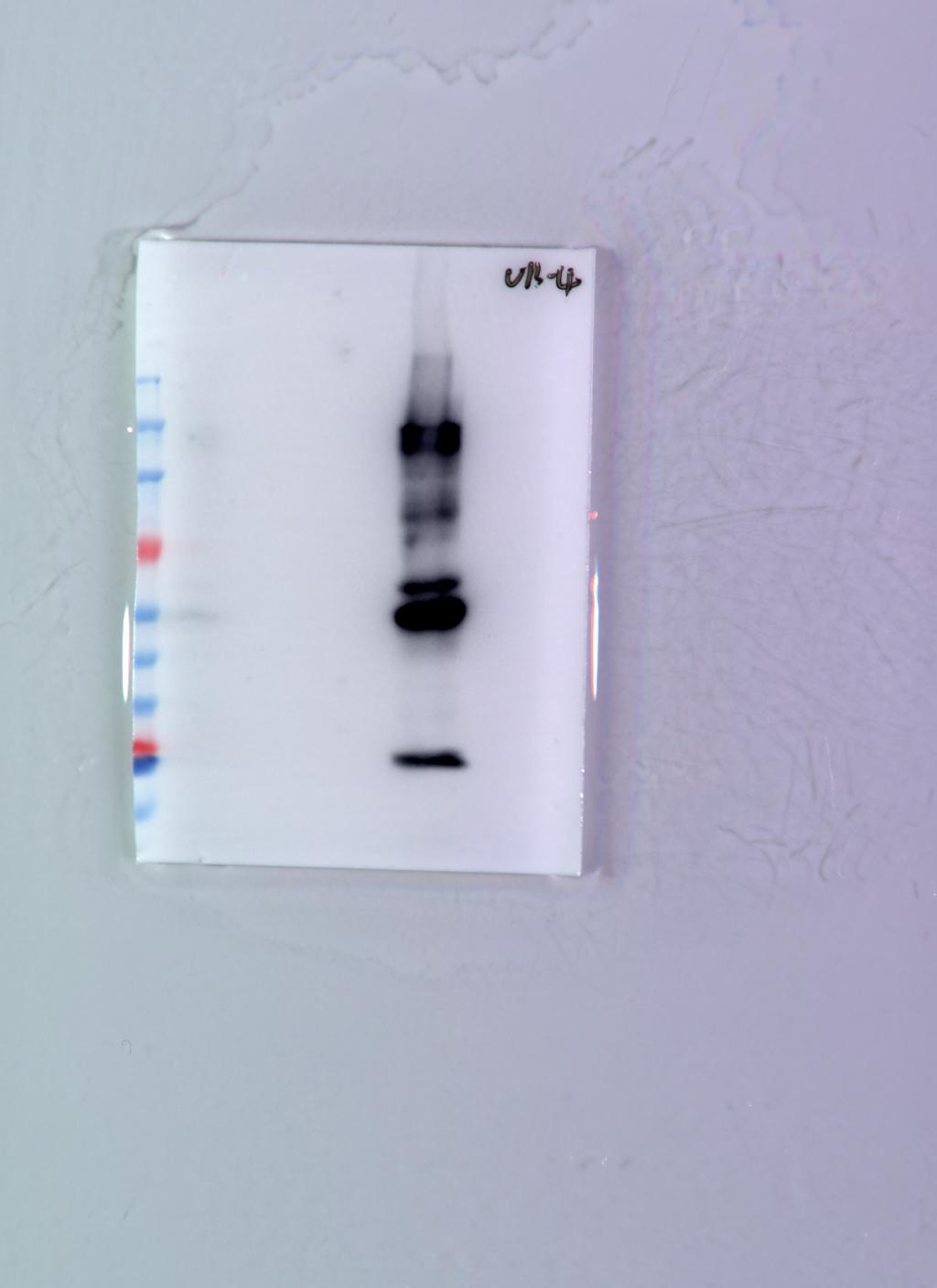

Supplement: Figure 3—source data 2. [file elife-110309-fig3-data2.zip › Figure 3-Source Data 16/UB-4 0-1 2022.04.26_17.11.19_Ch+Marker.jpg]

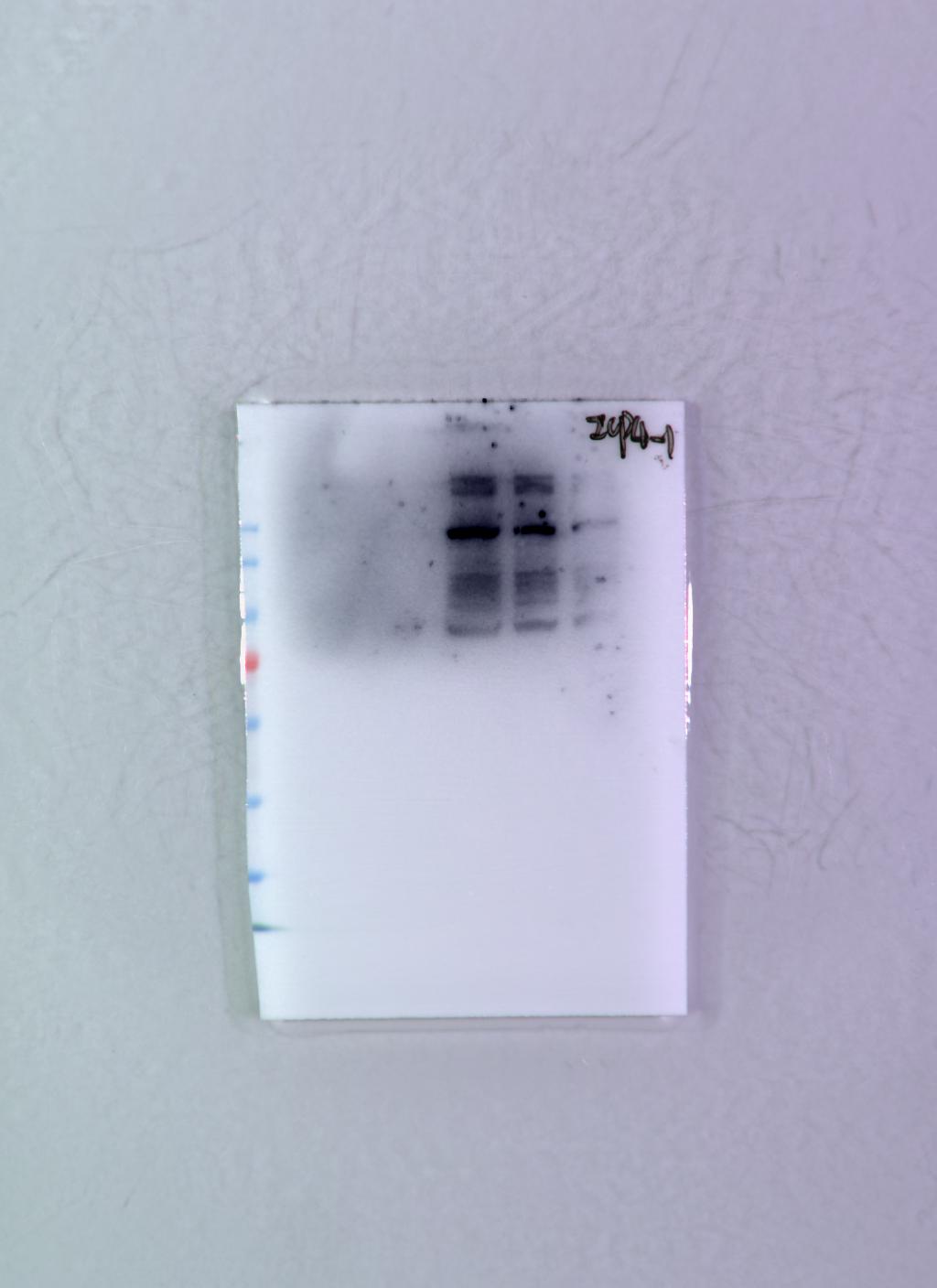

Supplement: Figure 3—source data 2. [file elife-110309-fig3-data2.zip › Figure 3-Source Data 18/ICP4 4-5 2026.03.31_18.14.27_Ch+Marker.jpg]

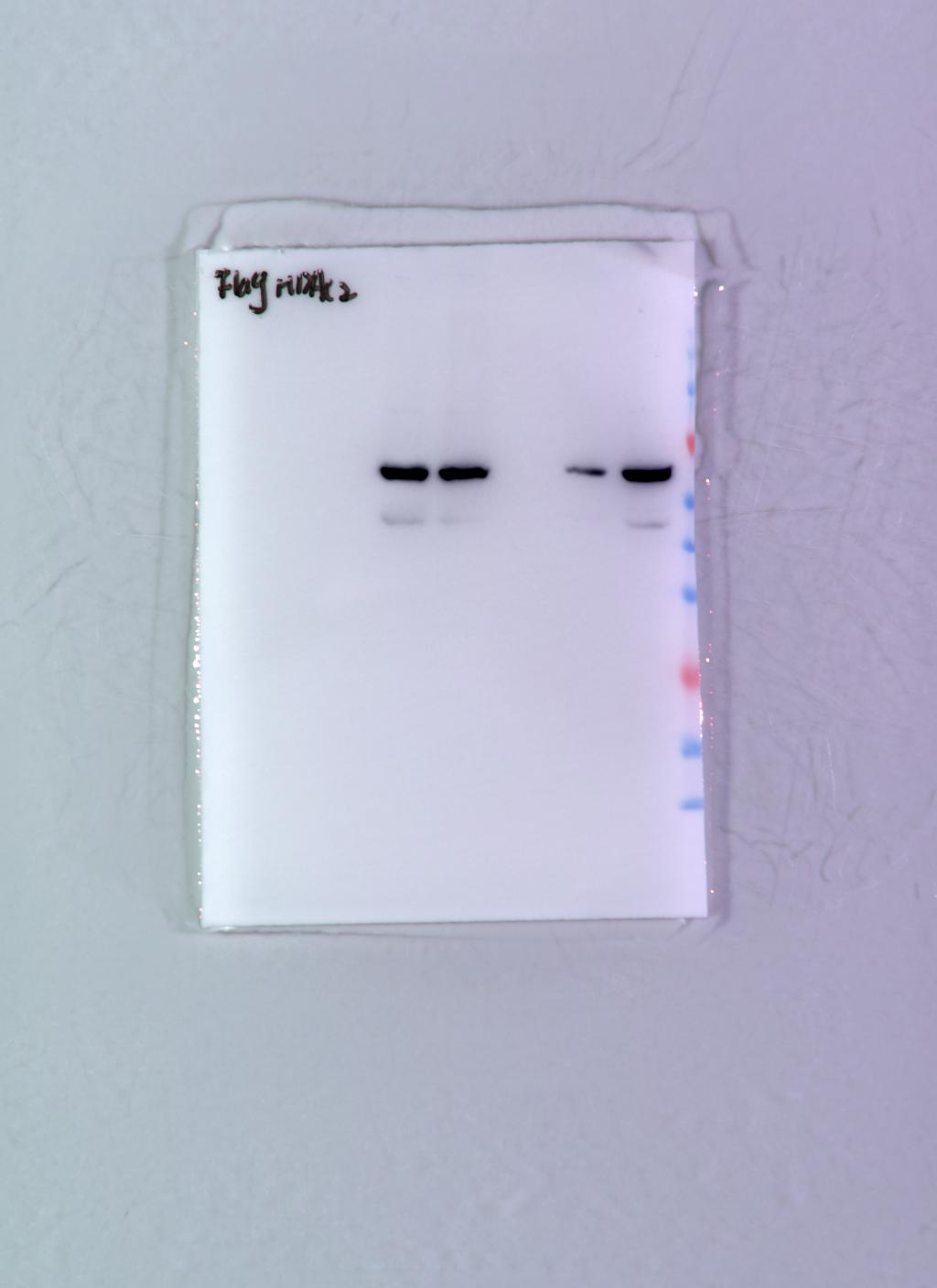

Supplement: Figure 3—source data 2. [file elife-110309-fig3-data2.zip › Figure 3-Source Data 18/ip hdac2 0-3 2022.03.07_12.30.14_Ch+Marker.jpg]

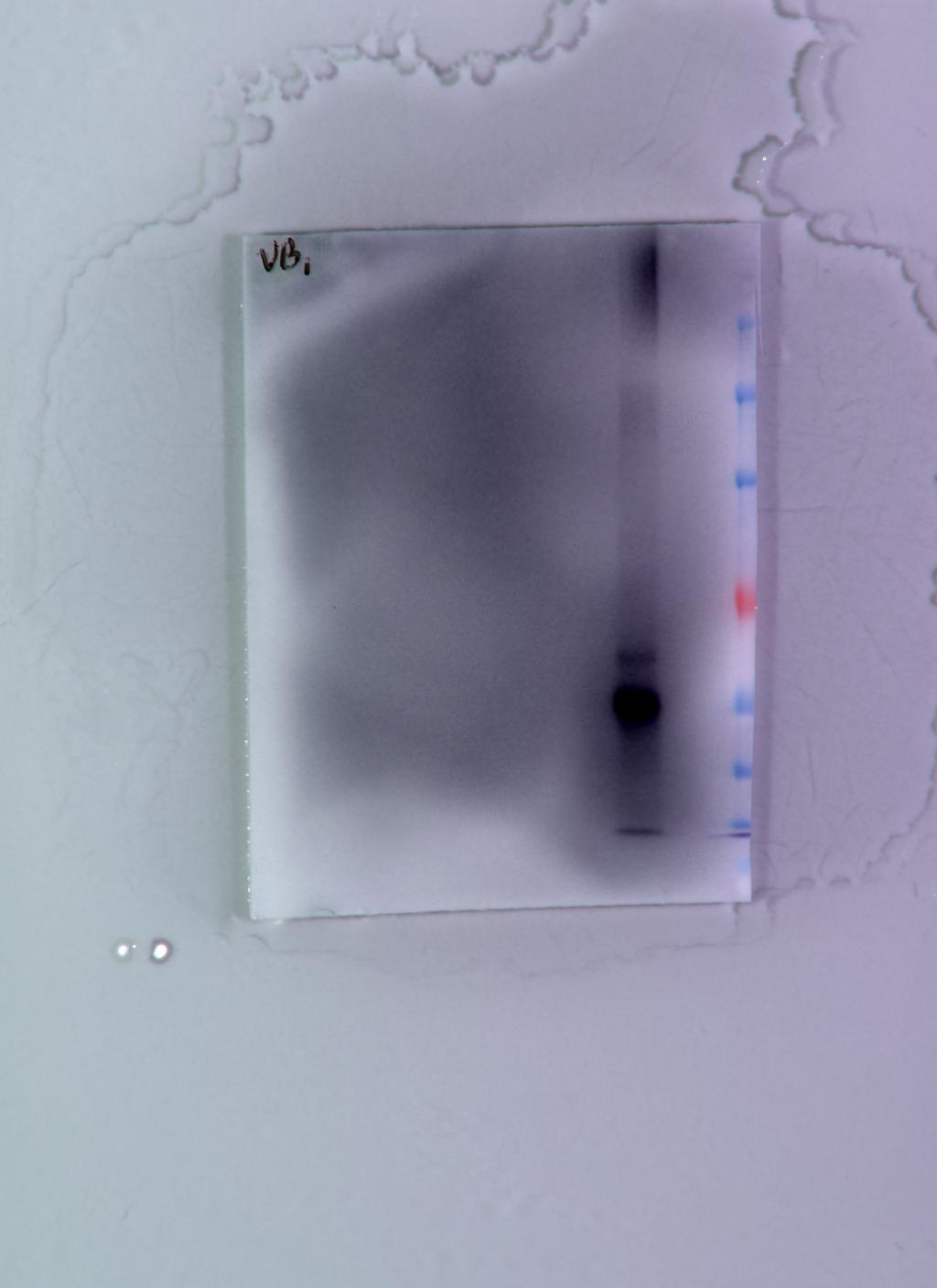

Supplement: Figure 3—source data 2. [file elife-110309-fig3-data2.zip › Figure 3-Source Data 18/UB 0-9 2022.03.06_21.26.22_Ch+Marker.jpg]

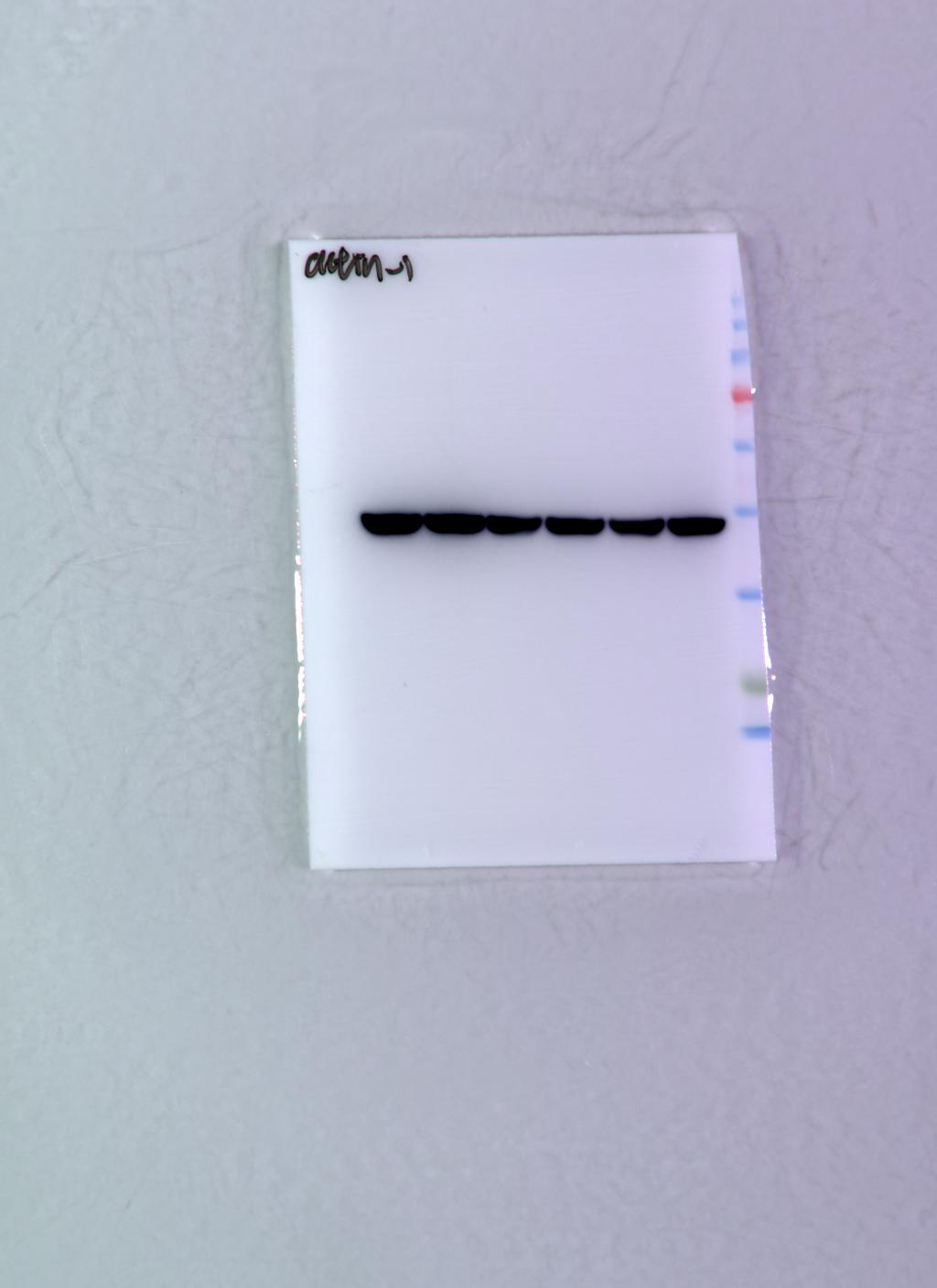

Supplement: Figure 3—source data 2. [file elife-110309-fig3-data2.zip › Figure 3-Source Data 20/ACTIN 1-4 2026.03.24_17.22.41_Ch+Marker.jpg]

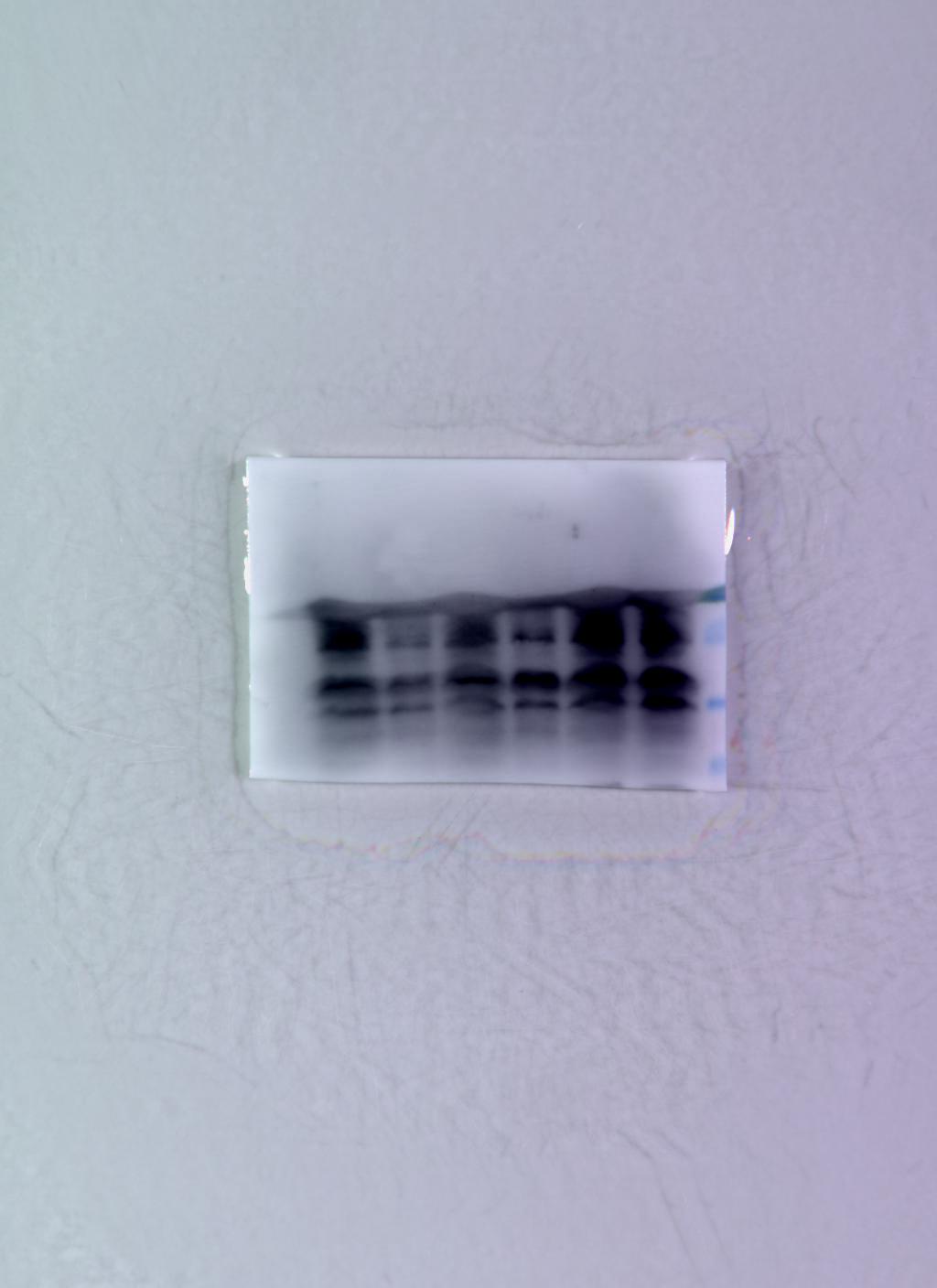

Supplement: Figure 3—source data 2. [file elife-110309-fig3-data2.zip › Figure 3-Source Data 20/ATM 1-4 2026.03.31_19.41.45_Ch+Marker.jpg]

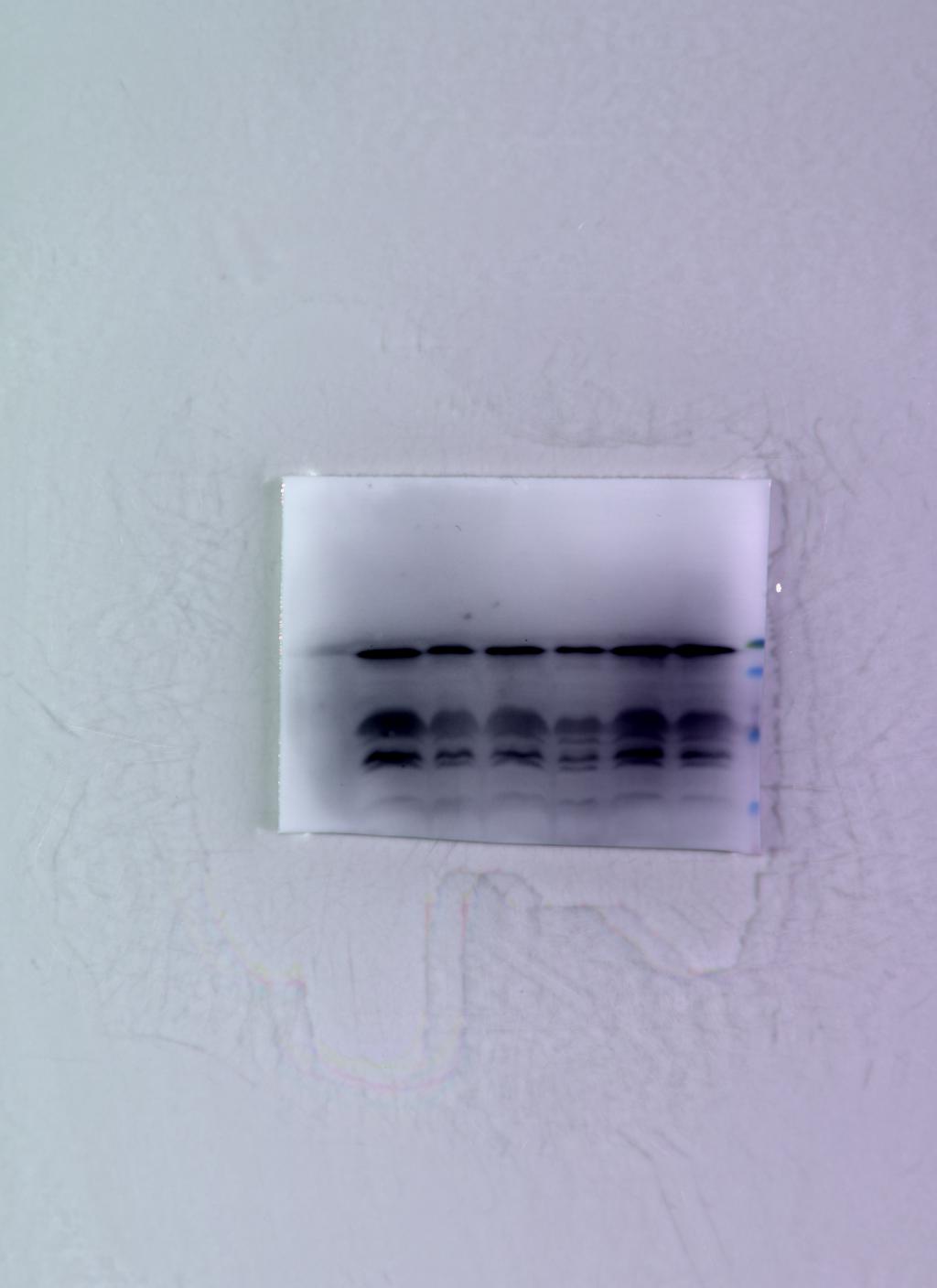

Supplement: Figure 3—source data 2. [file elife-110309-fig3-data2.zip › Figure 3-Source Data 20/ATR 1-5 2026.03.31_19.15.28_Ch+Marker.jpg]

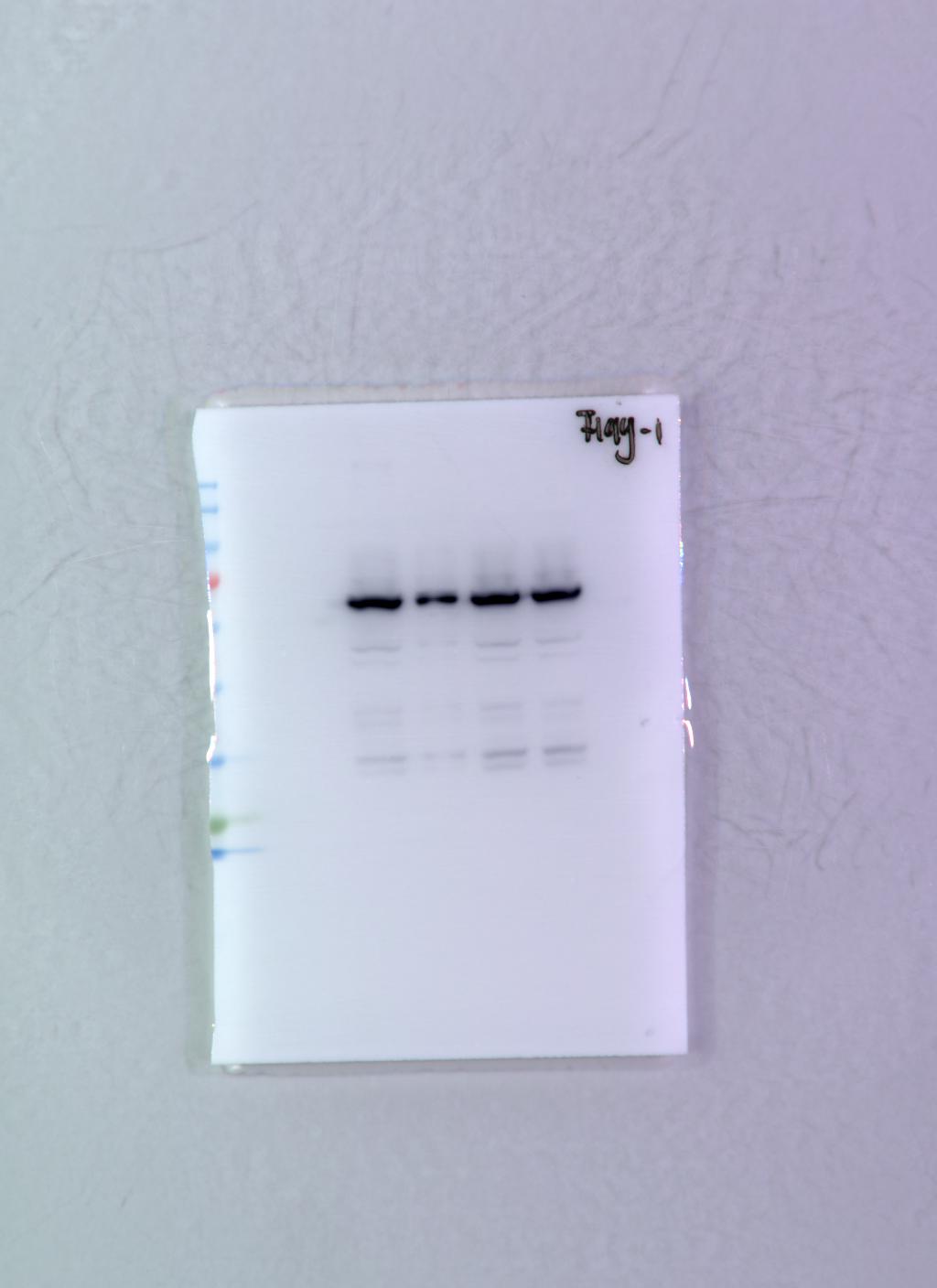

Supplement: Figure 3—source data 2. [file elife-110309-fig3-data2.zip › Figure 3-Source Data 20/FLAG 1-2 2026.03.31_18.42.29_Ch+Marker.jpg]

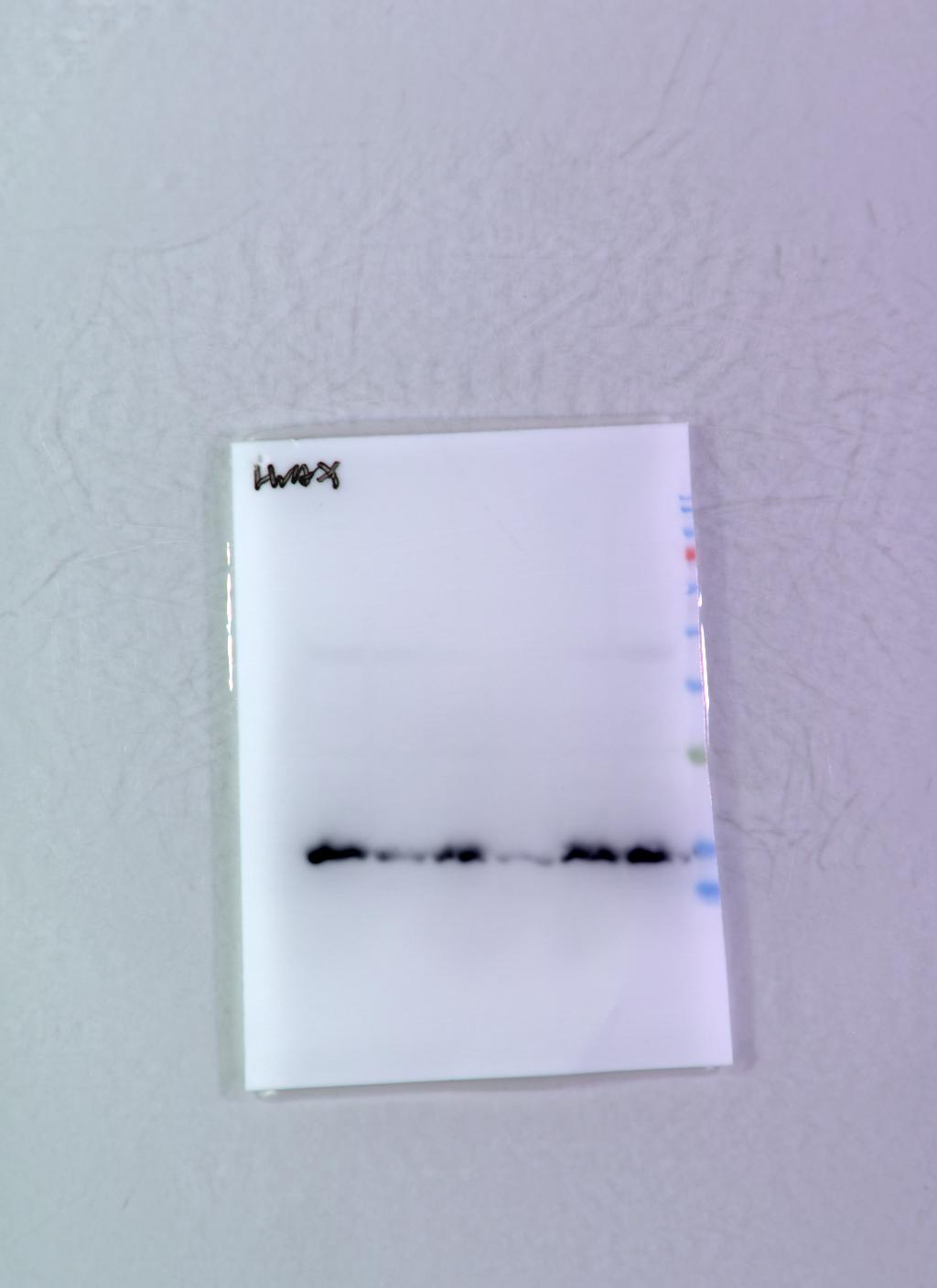

Supplement: Figure 3—source data 2. [file elife-110309-fig3-data2.zip › Figure 3-Source Data 20/H2AX 1-3 2026.03.31_19.57.41_Ch+Marker.jpg]

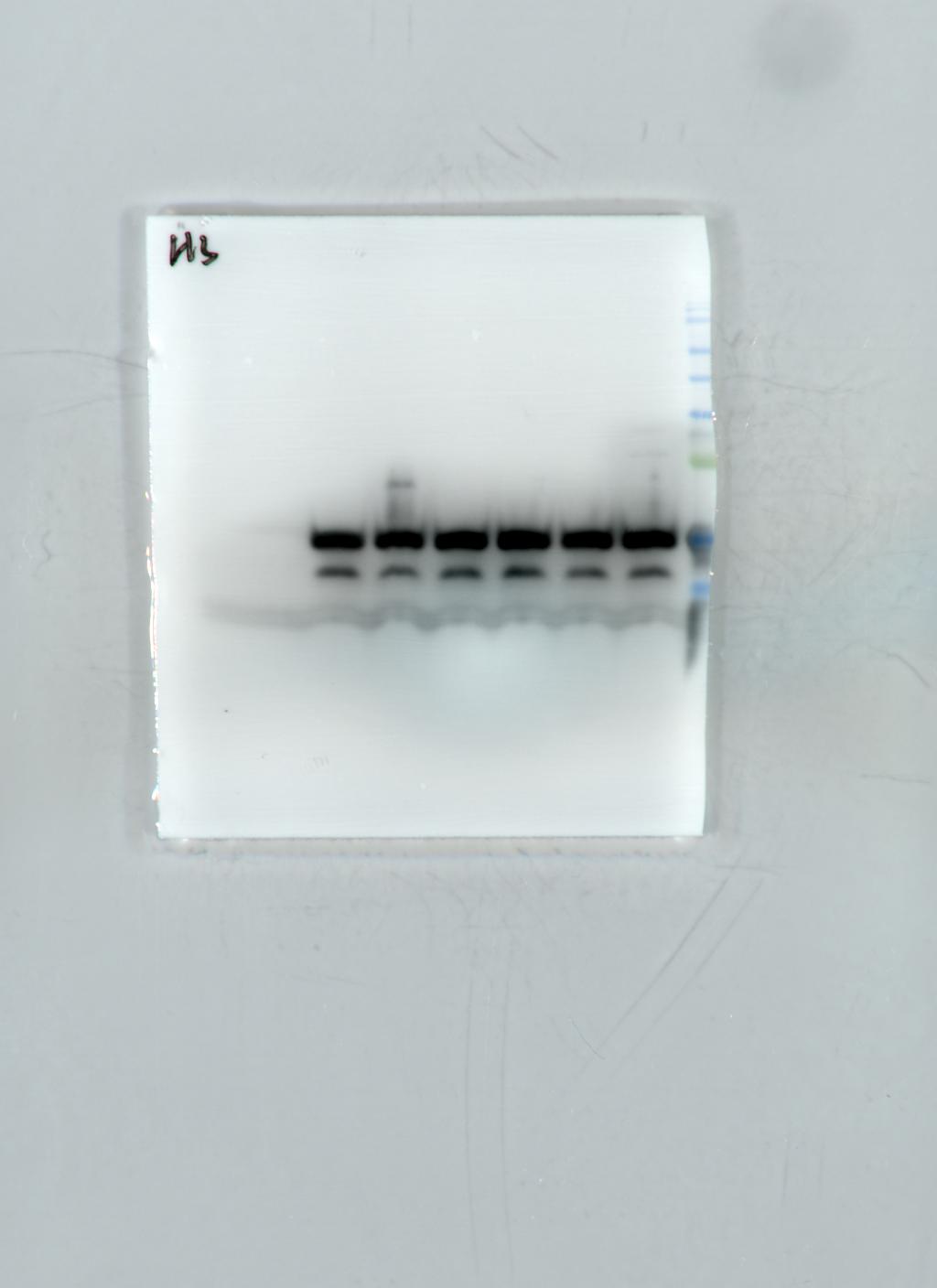

Supplement: Figure 3—source data 2. [file elife-110309-fig3-data2.zip › Figure 3-Source Data 20/H3 1-6 2026.06.05_12.21.05_Ch+Marker.jpg]

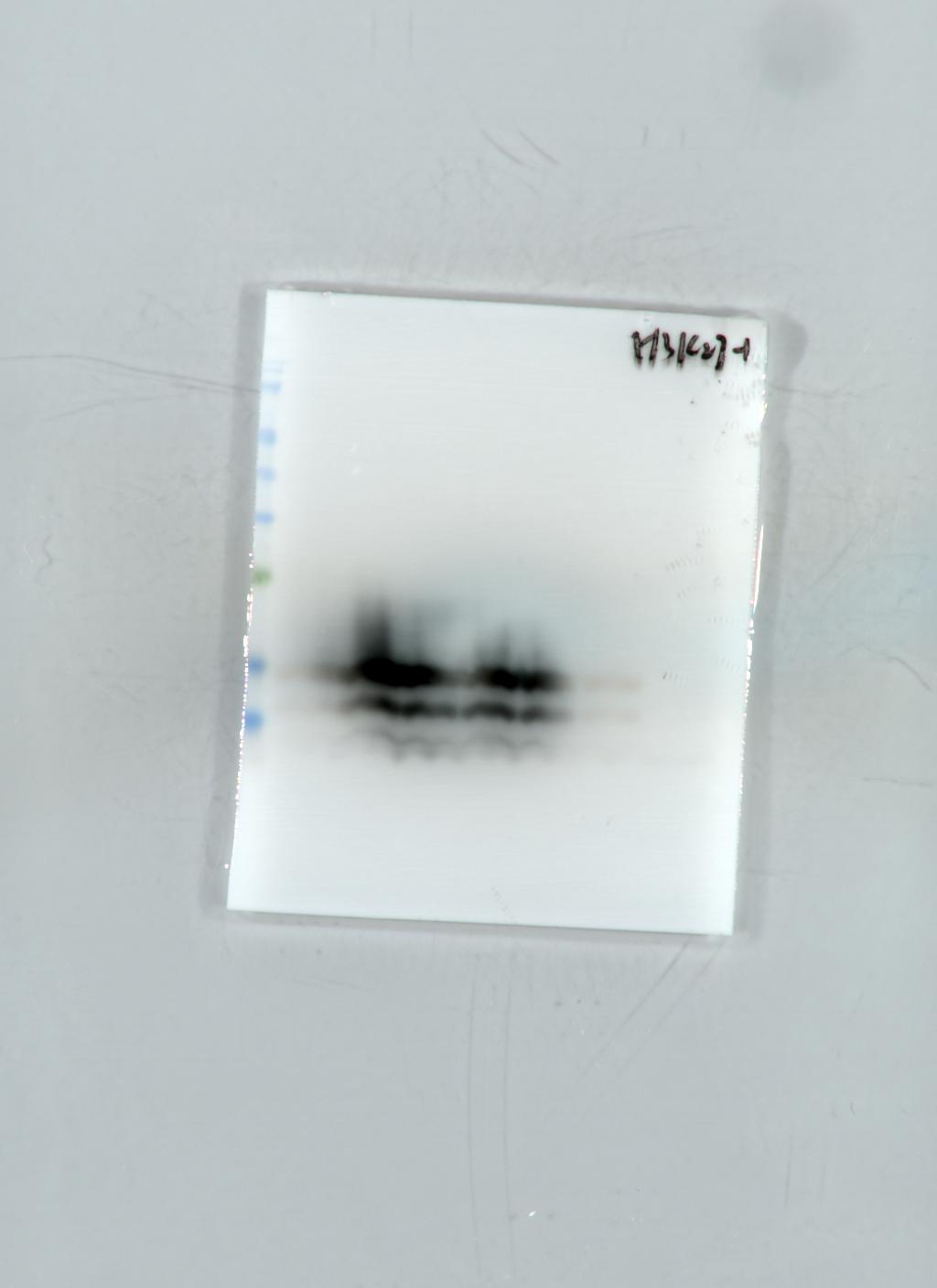

Supplement: Figure 3—source data 2. [file elife-110309-fig3-data2.zip › Figure 3-Source Data 20/H3K 56 2026.06.05_12.30.34_Ch+Marker.jpg]

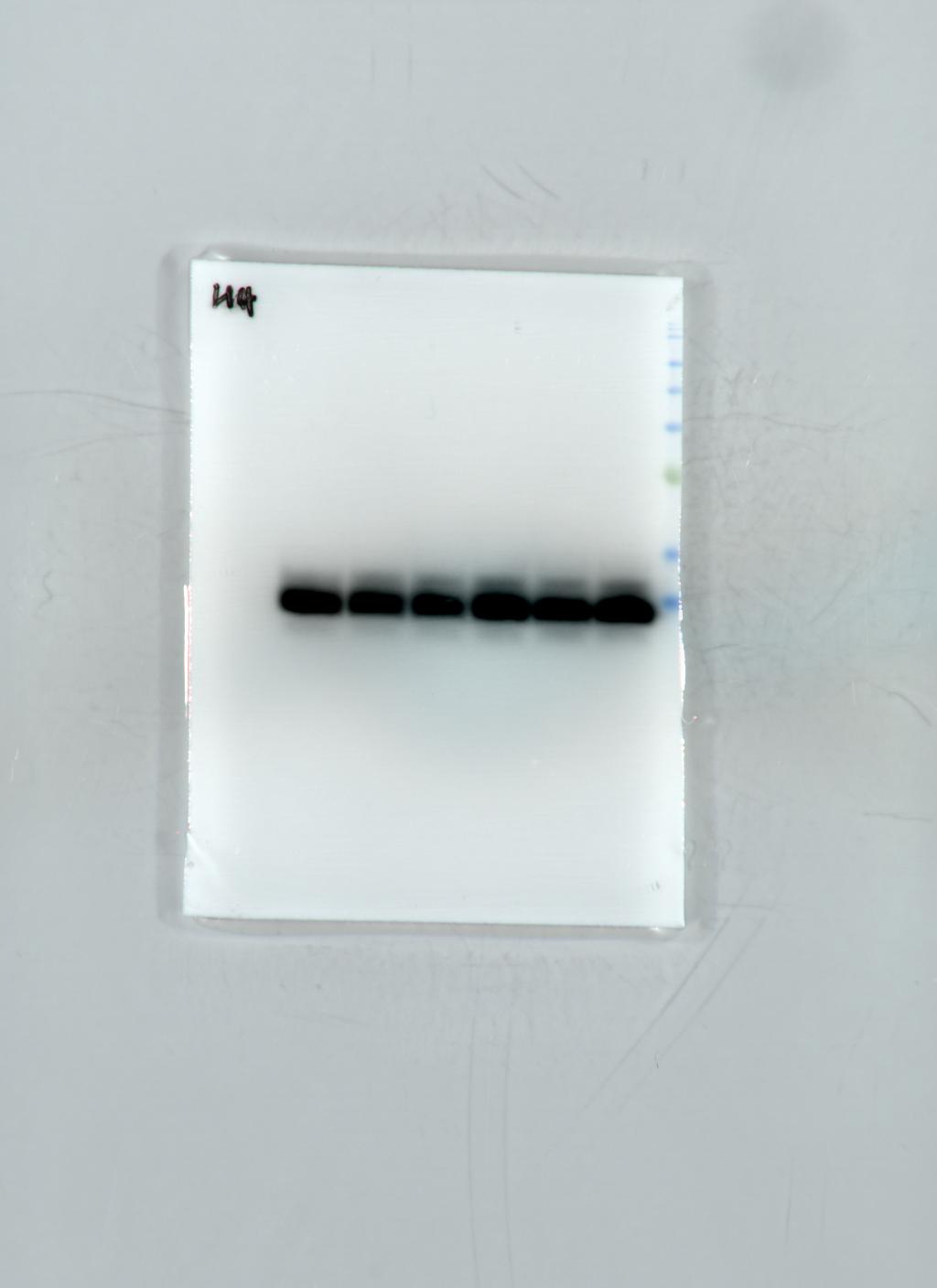

Supplement: Figure 3—source data 2. [file elife-110309-fig3-data2.zip › Figure 3-Source Data 20/h4 1-2 2026.06.04_15.48.06_Ch+Marker.jpg]

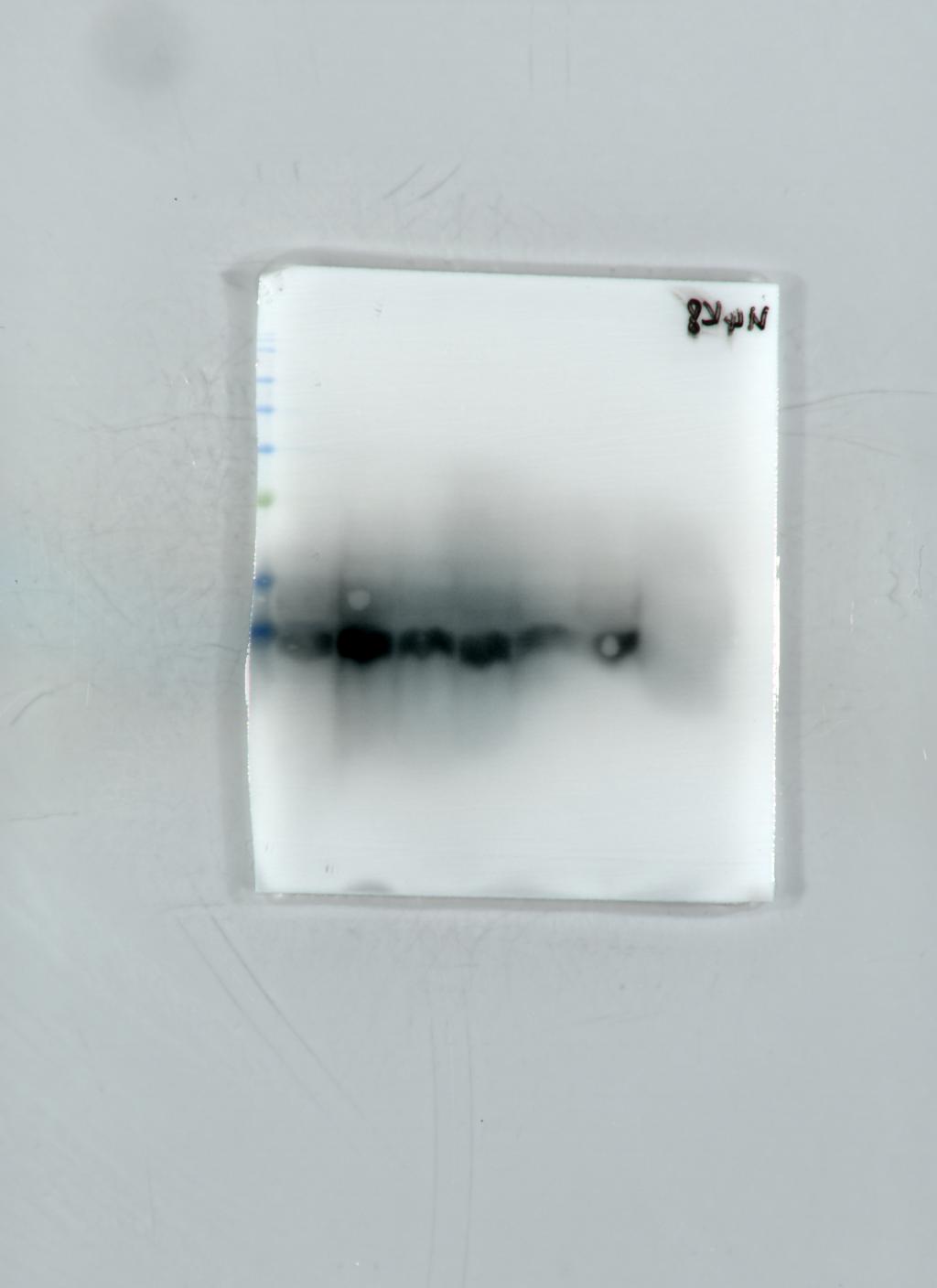

Supplement: Figure 3—source data 2. [file elife-110309-fig3-data2.zip › Figure 3-Source Data 20/h4k8 2-0 2026.06.04_16.34.12_Ch+Marker.jpg]

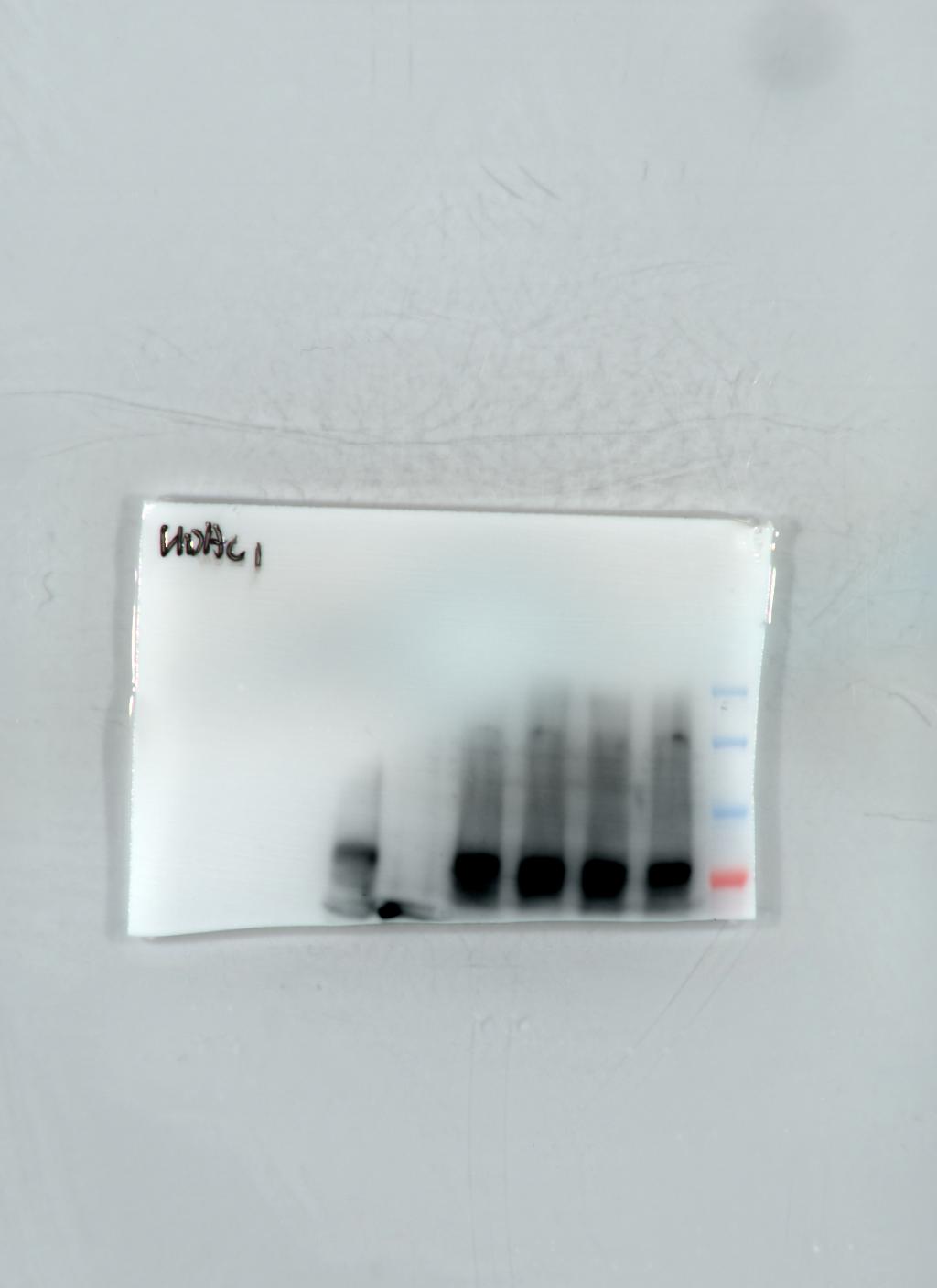

Supplement: Figure 3—source data 2. [file elife-110309-fig3-data2.zip › Figure 3-Source Data 20/hdac1 1-2 2026.06.04_16.07.49_Ch+Marker.jpg]

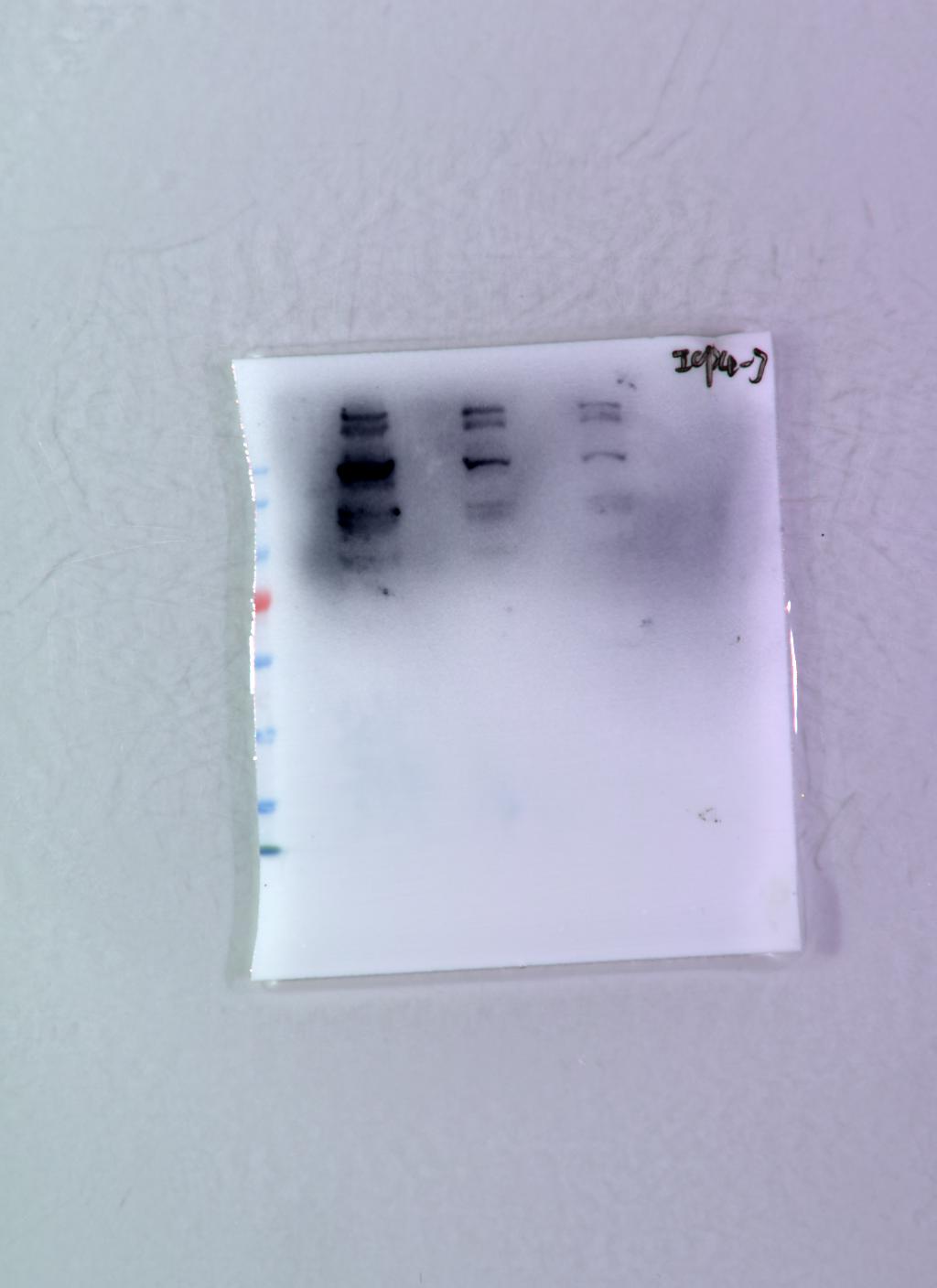

Supplement: Figure 3—source data 2. [file elife-110309-fig3-data2.zip › Figure 3-Source Data 20/ICP4 1-4 2026.03.31_17.57.19_Ch+Marker.jpg]

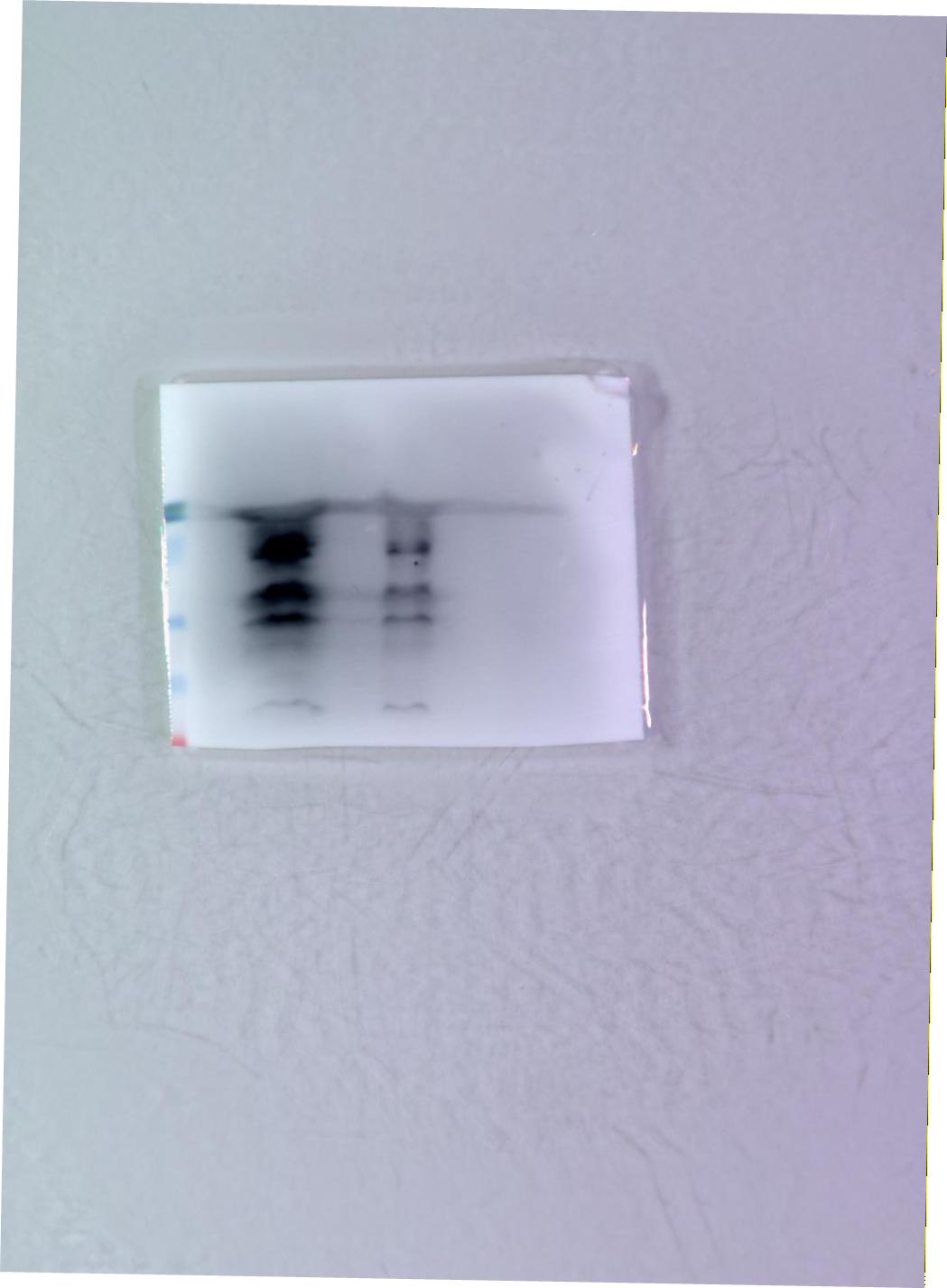

Supplement: Figure 3—source data 2. [file elife-110309-fig3-data2.zip › Figure 3-Source Data 20/P-ATM 1-7 2026.03.31_19.50.00_Ch+Marker.jpg]

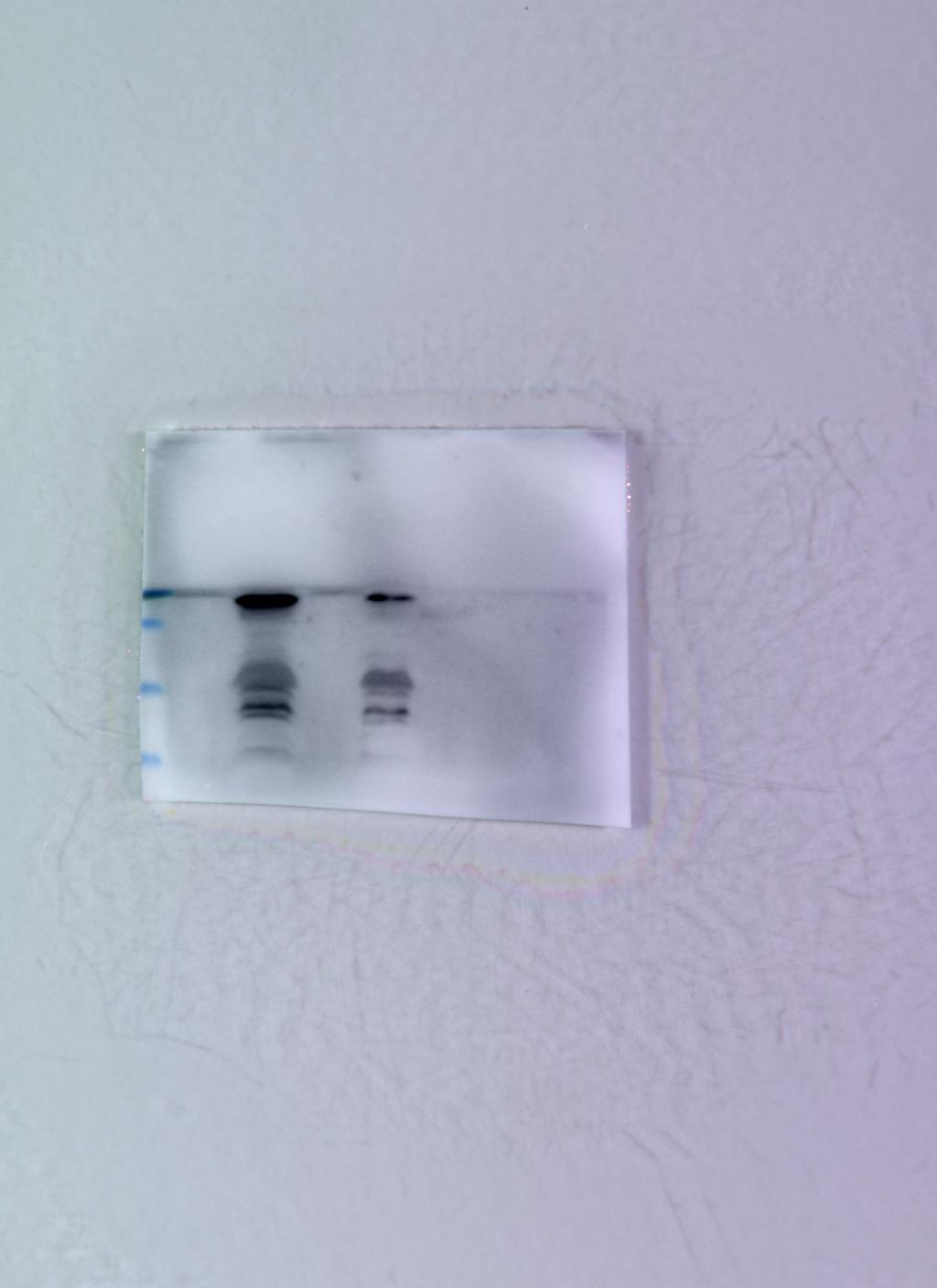

Supplement: Figure 3—source data 2. [file elife-110309-fig3-data2.zip › Figure 3-Source Data 20/P-ATR 1-1 2026.03.31_19.29.06_Ch+Marker.jpg]

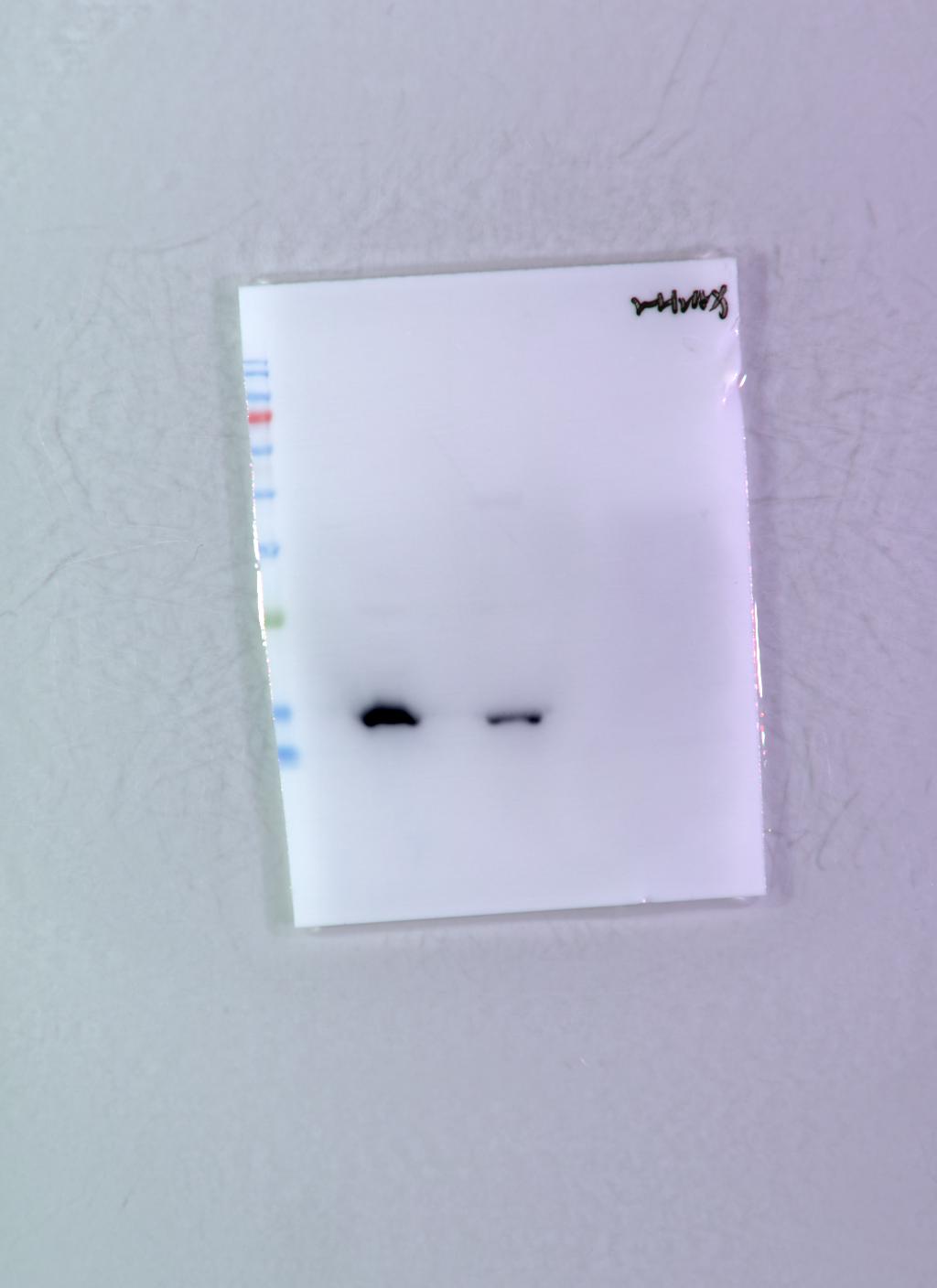

Supplement: Figure 3—source data 2. [file elife-110309-fig3-data2.zip › Figure 3-Source Data 20/P-H2AX 1-2 2026.03.31_19.52.31_Ch+Marker.jpg]

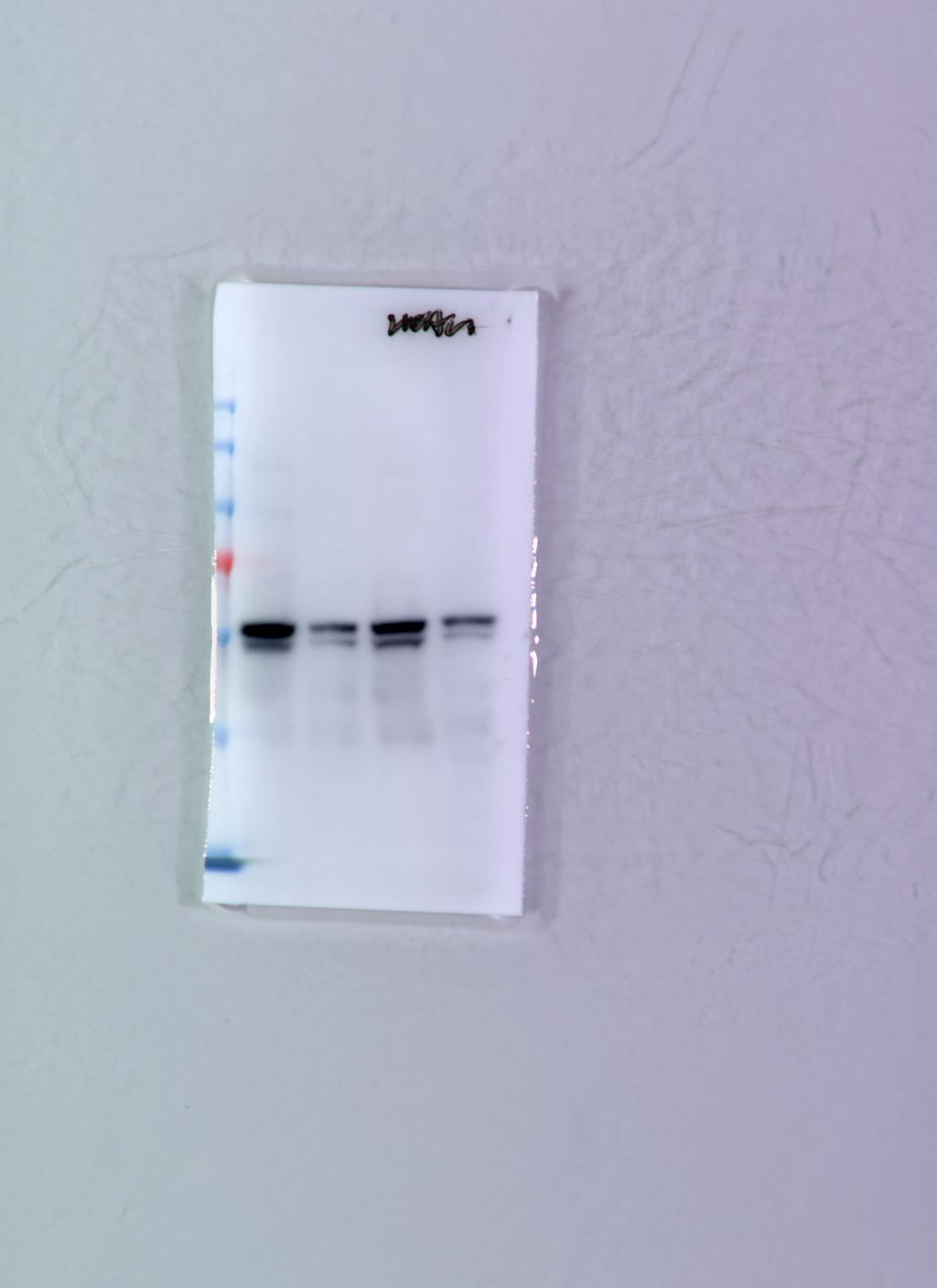

Supplement: Figure 4—source data 2. [file elife-110309-fig4-data2.zip › Figure4-Source Data4/HDAC 1-6 2023.12.03_19.34.45_Ch+Marker.jpg]

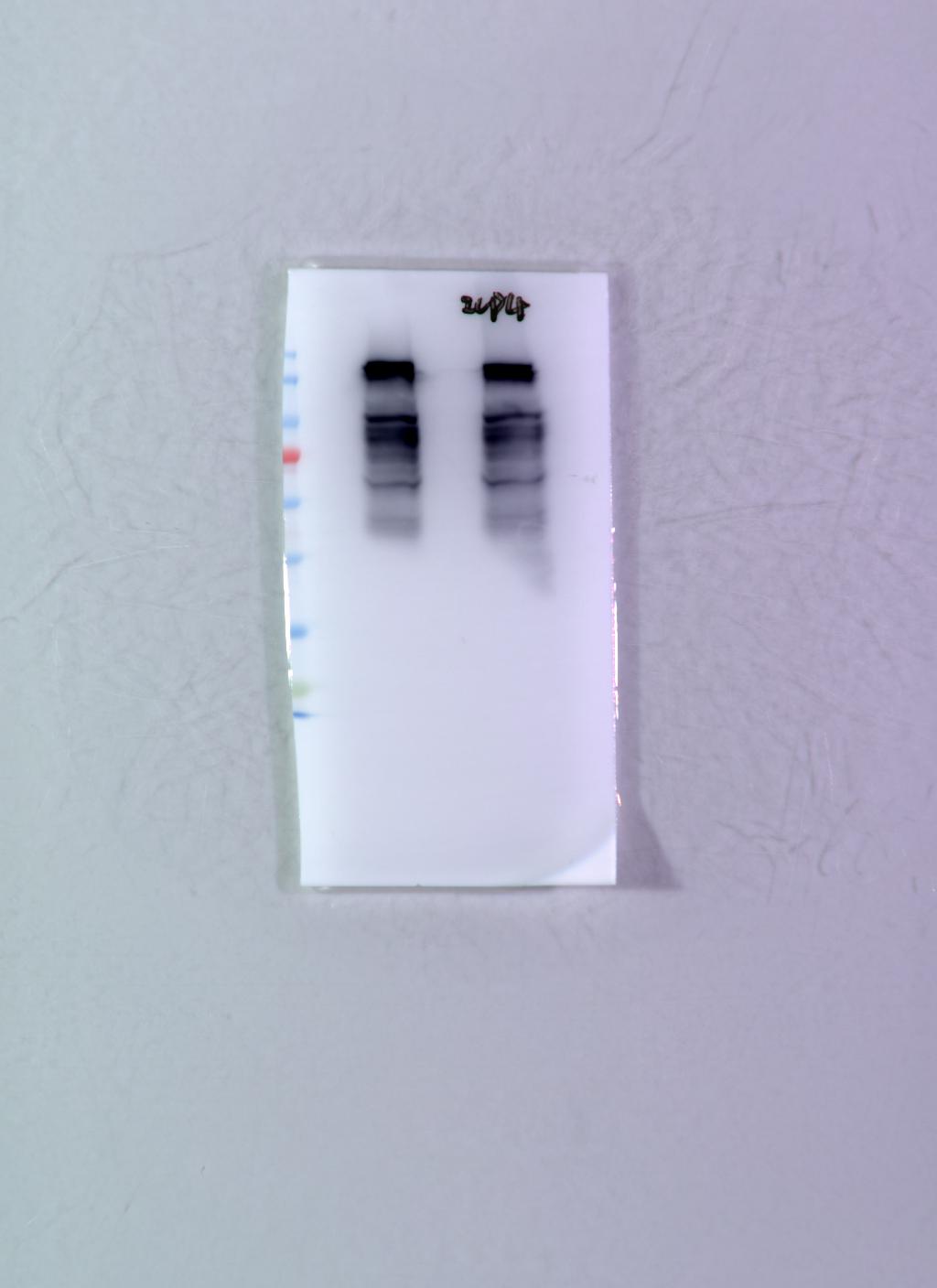

Supplement: Figure 4—source data 2. [file elife-110309-fig4-data2.zip › Figure4-Source Data4/INPUT ICP4 0-1 2023.12.02_13.13.44_Ch+Marker.jpg]

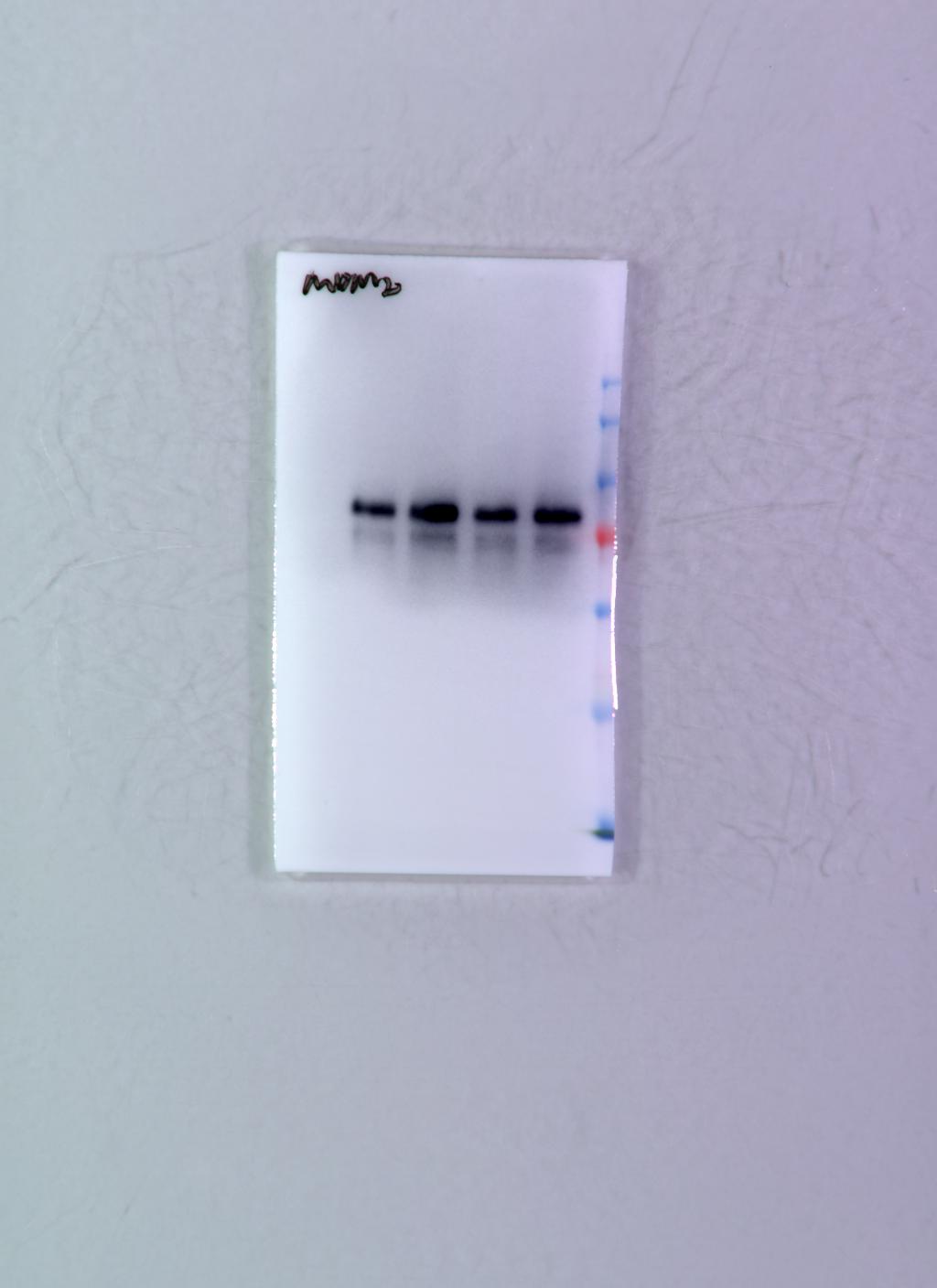

Supplement: Figure 4—source data 2. [file elife-110309-fig4-data2.zip › Figure4-Source Data4/INPUT MDM2 0-2 2023.12.03_17.53.54_Ch+Marker.jpg]

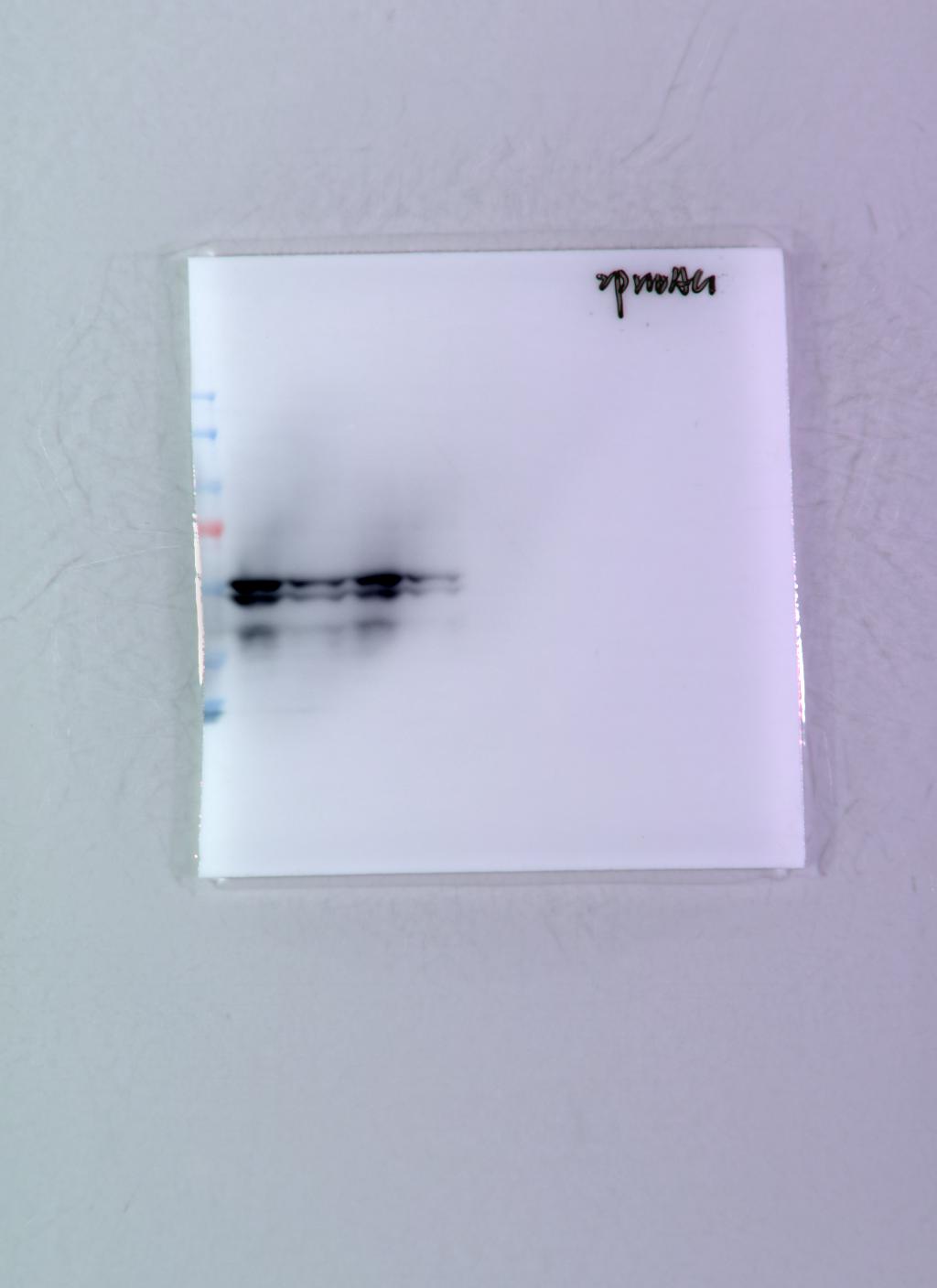

Supplement: Figure 4—source data 2. [file elife-110309-fig4-data2.zip › Figure4-Source Data4/IP HDAC 0-3 2023.12.02_13.10.46_Ch+Marker.jpg]

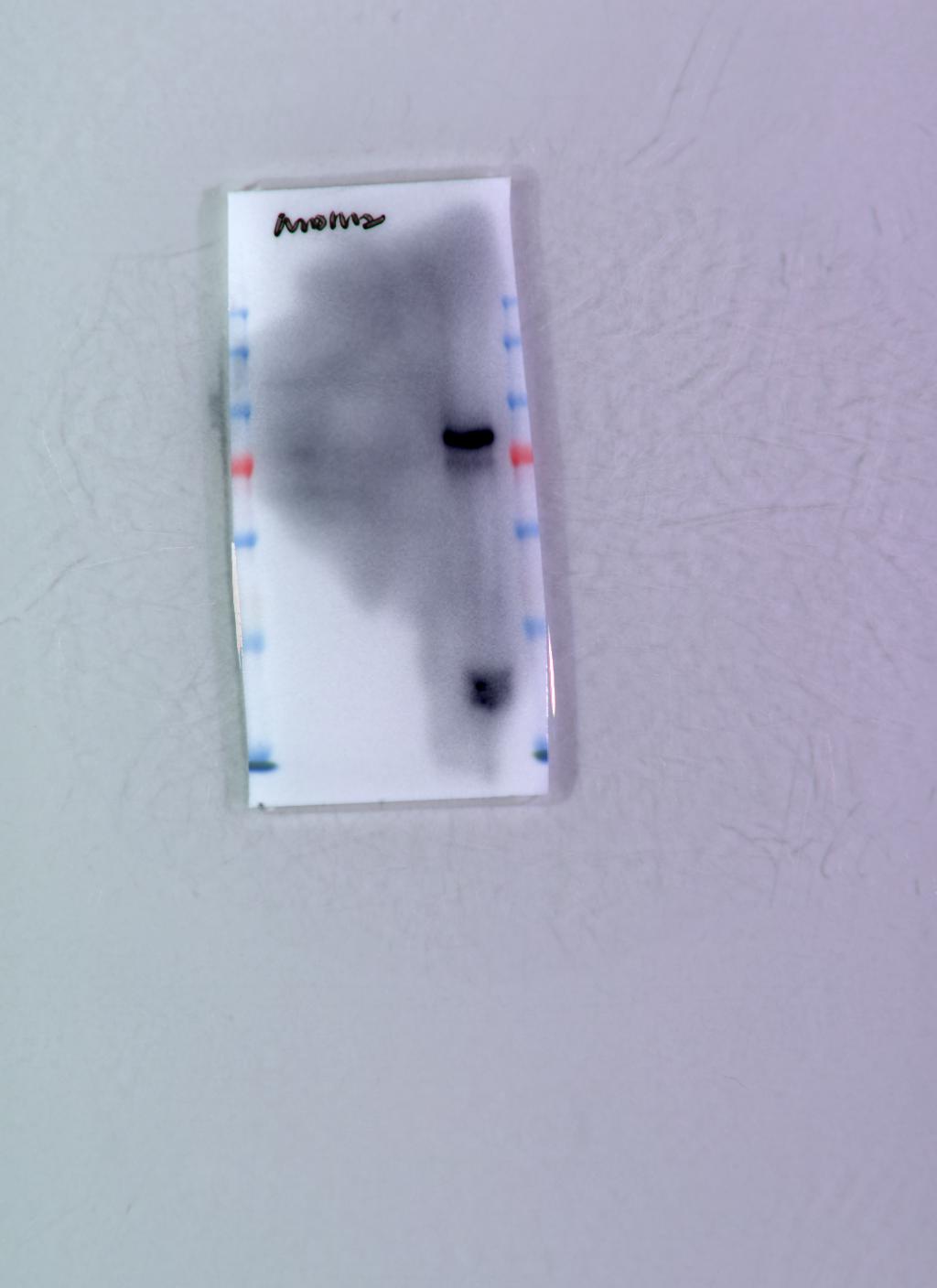

Supplement: Figure 4—source data 2. [file elife-110309-fig4-data2.zip › Figure4-Source Data4/IP MDM2 0-3 2023.12.03_17.57.31_Ch+Marker.jpg]

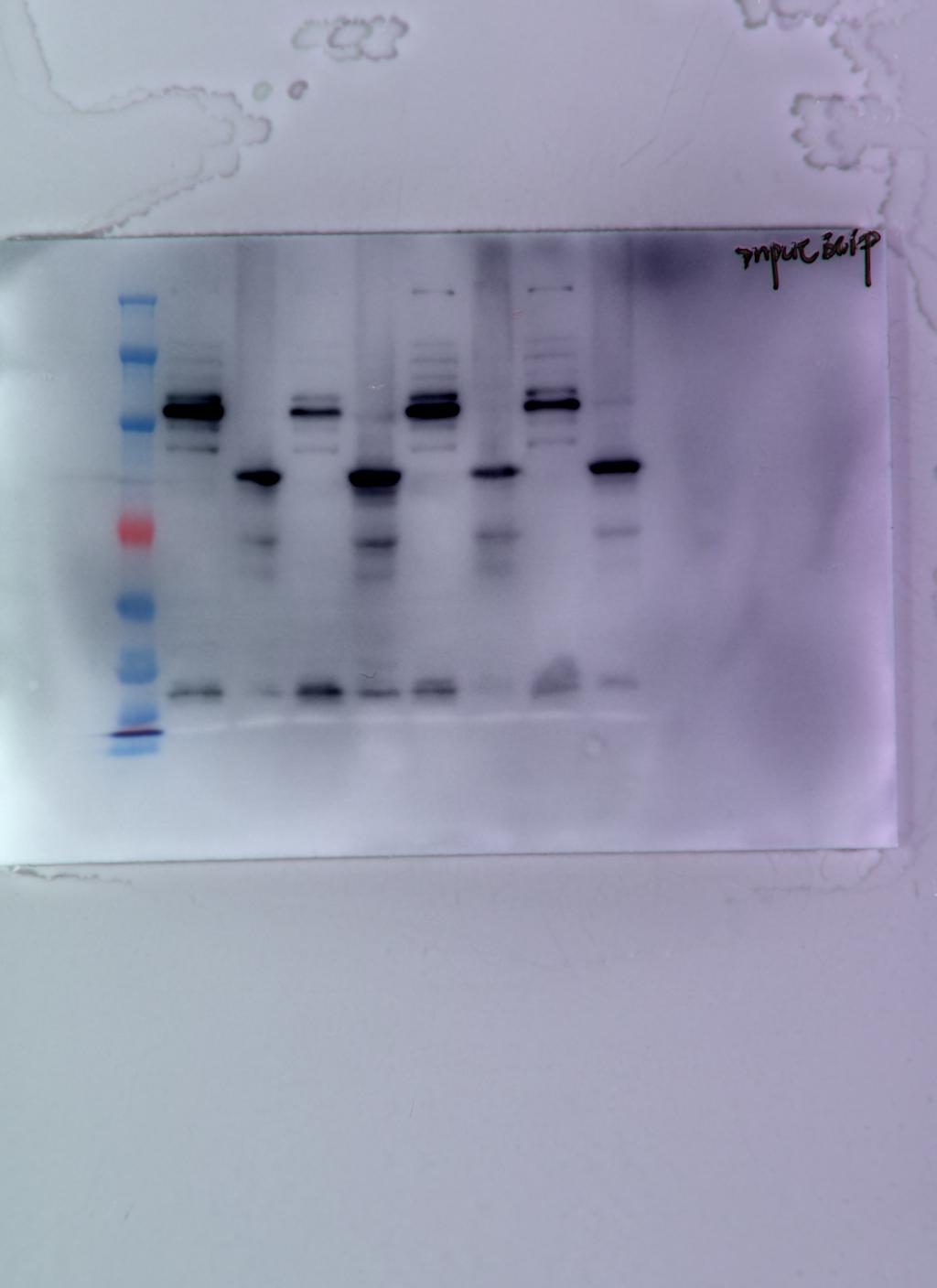

Supplement: Figure 4—source data 2. [file elife-110309-fig4-data2.zip › Figure4-Source Data6/INPUT EGFP 0-6 2022.01.10_14.45.55_Ch+Marker.jpg]

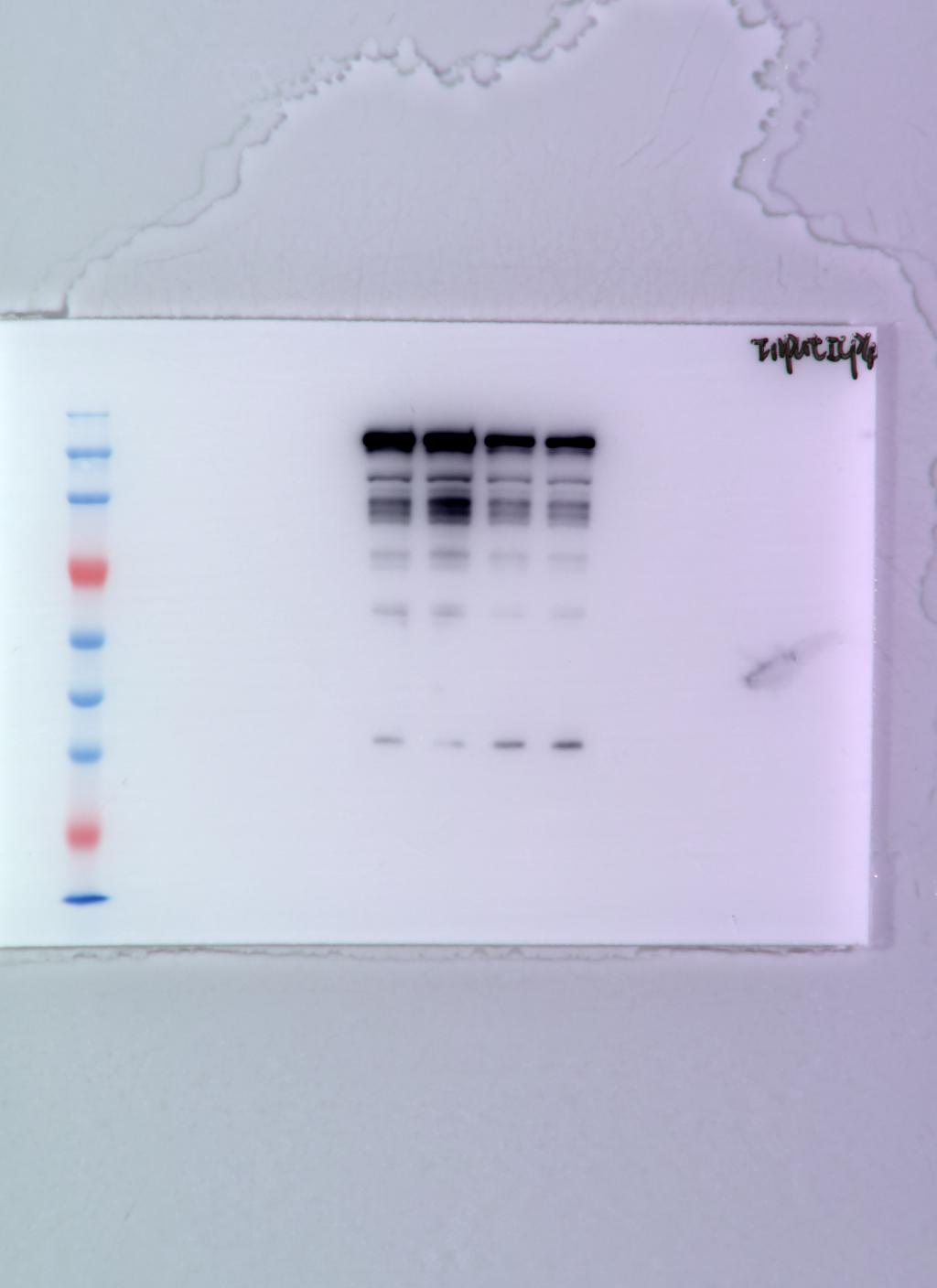

Supplement: Figure 4—source data 2. [file elife-110309-fig4-data2.zip › Figure4-Source Data6/INPUT ICP4 0-1 2021.12.18_16.07.17_Ch+Marker.jpg]

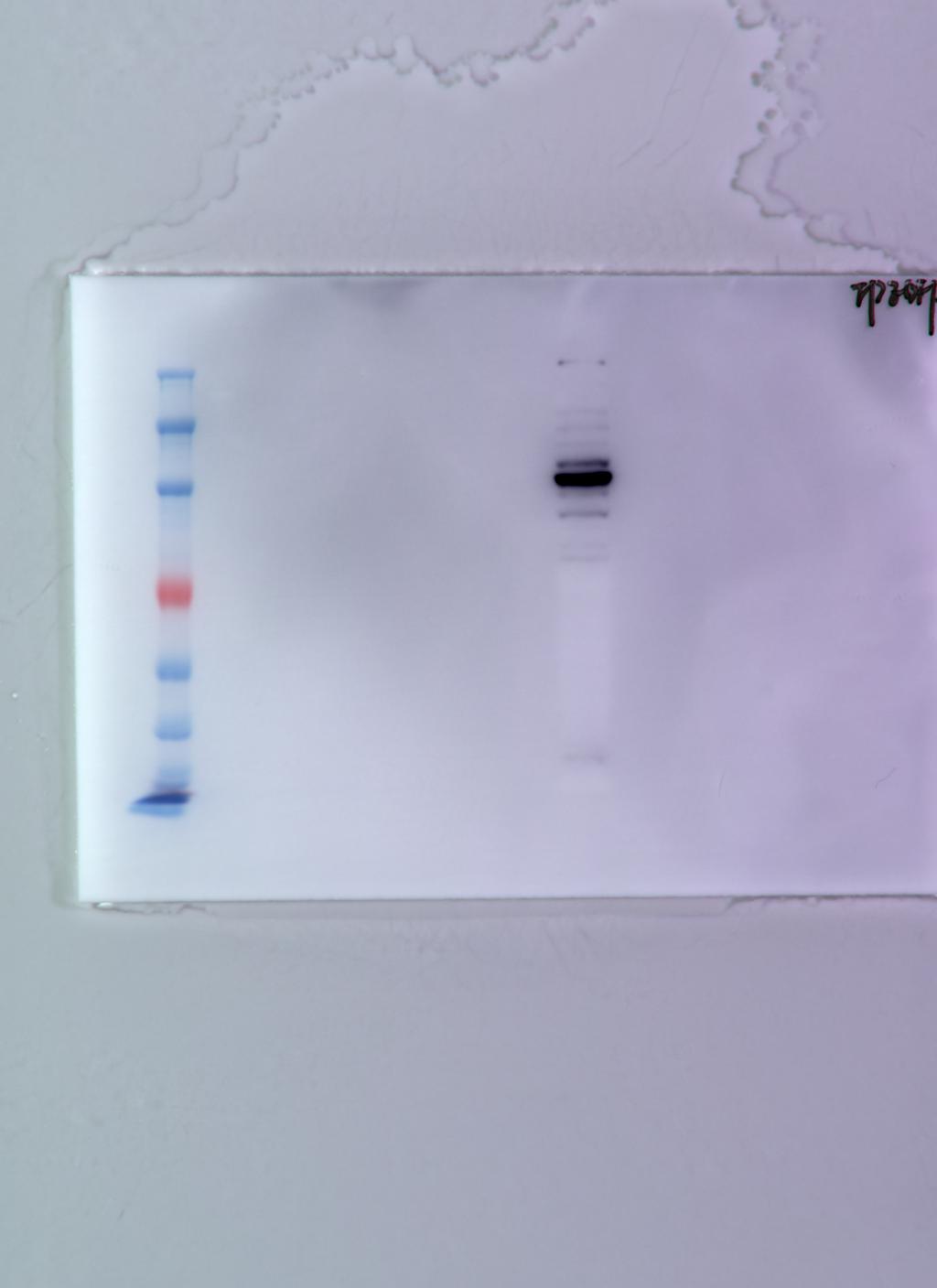

Supplement: Figure 4—source data 2. [file elife-110309-fig4-data2.zip › Figure4-Source Data6/IP EGFP 0 2022.01.10_14.24.16_Ch+Marker.jpg]

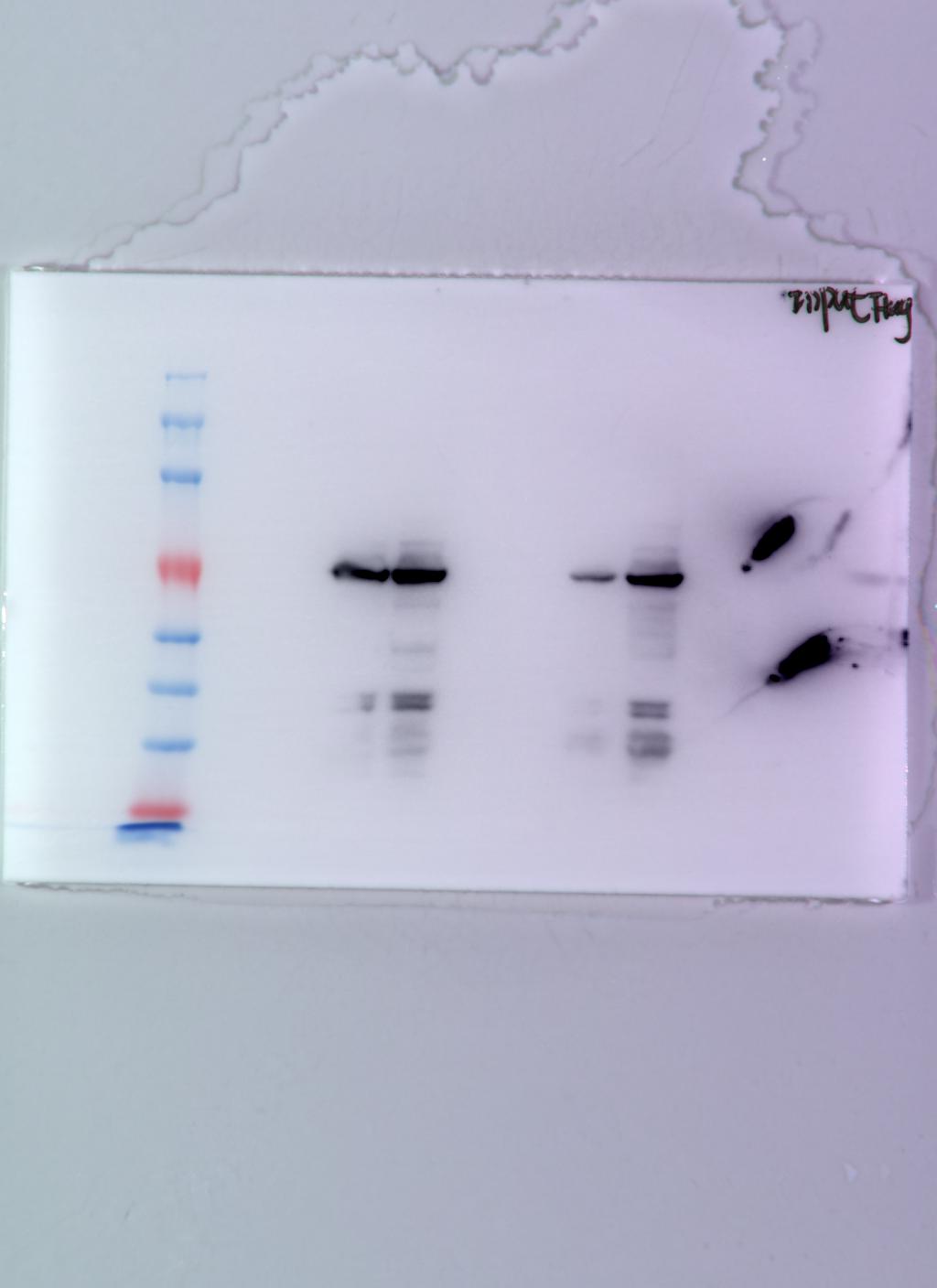

Supplement: Figure 4—source data 2. [file elife-110309-fig4-data2.zip › Figure4-Source Data6/IP FLAG 0-2 2021.12.18_15.47.58_Ch+Marker.jpg]

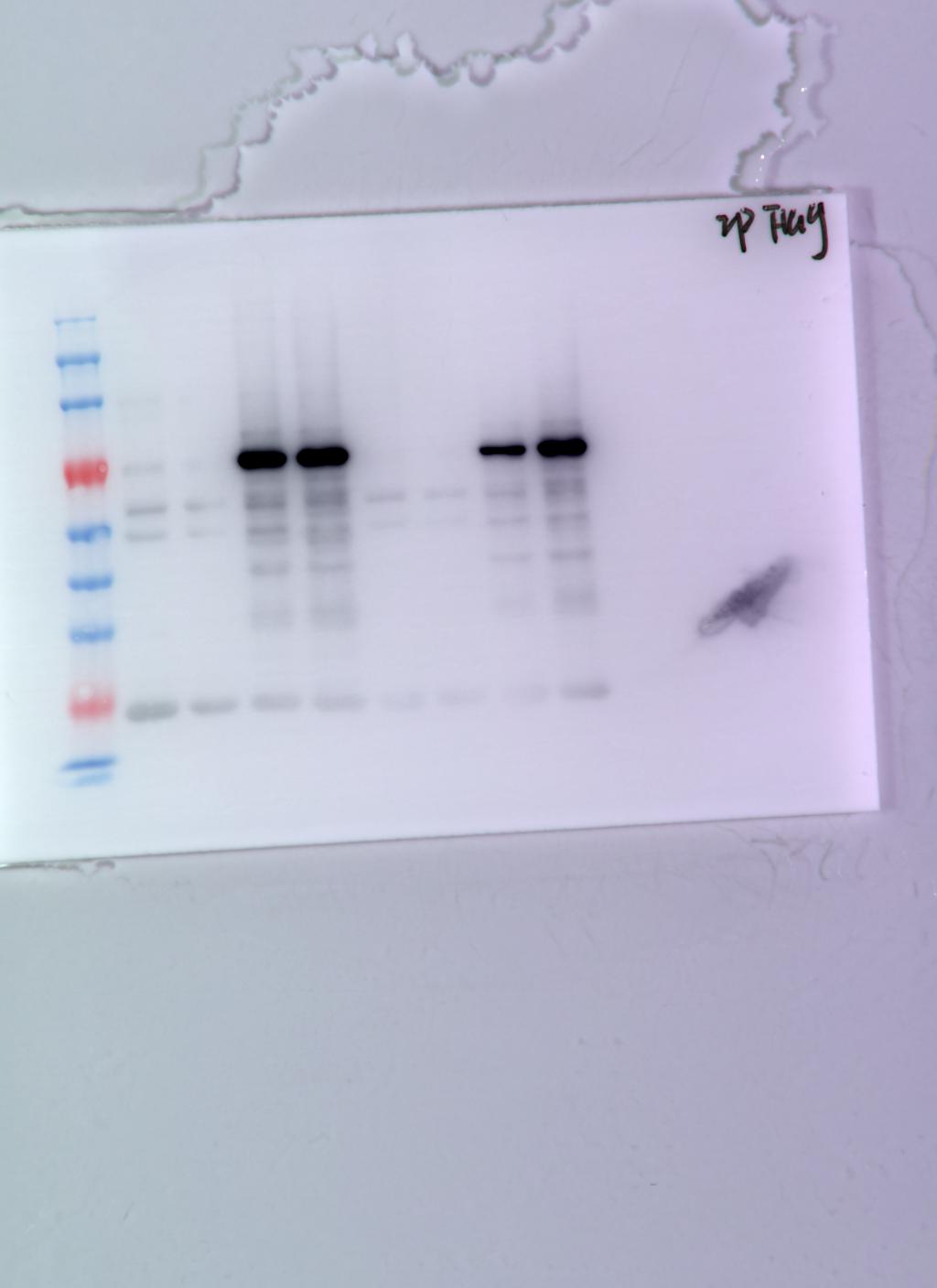

Supplement: Figure 4—source data 2. [file elife-110309-fig4-data2.zip › Figure4-Source Data6/IP FLAG 1-5 2021.12.18_16.02.41_Ch+Marker.jpg]

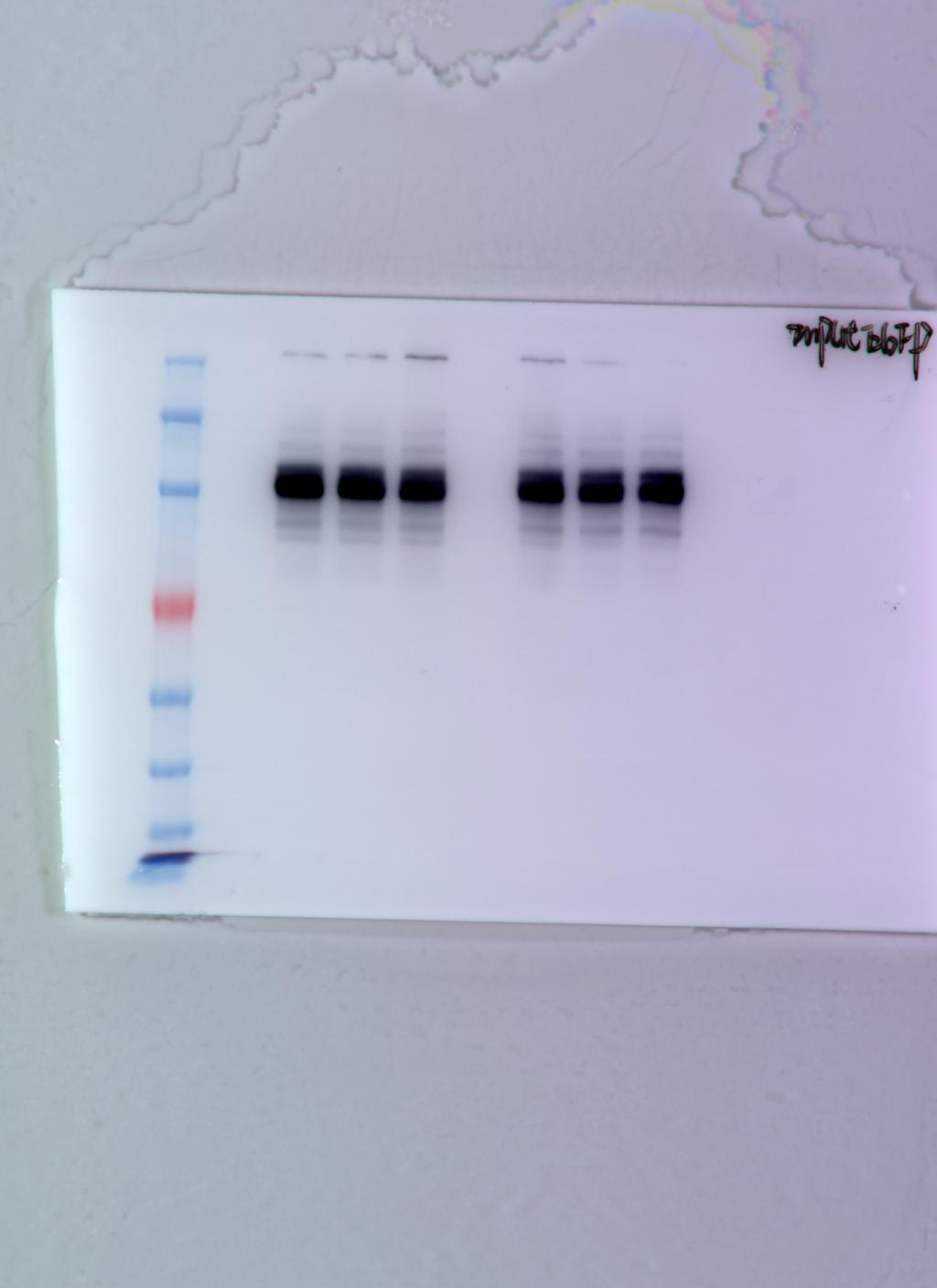

Supplement: Figure 4—source data 2. [file elife-110309-fig4-data2.zip › Figure4-Source Data8/INPUT EGFP 0-2 2022.01.15_16.56.29_Ch+Marker.jpg]

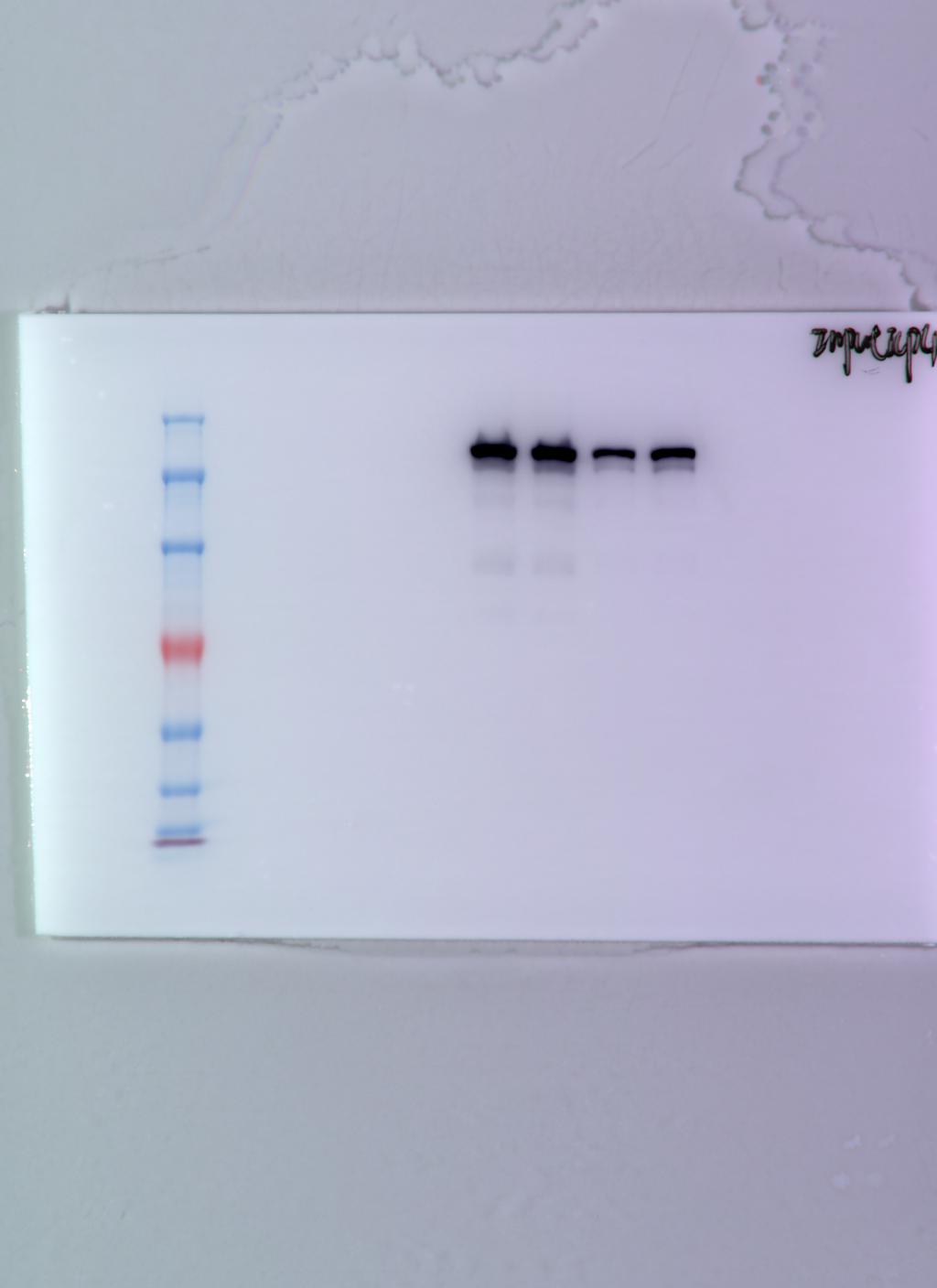

Supplement: Figure 4—source data 2. [file elife-110309-fig4-data2.zip › Figure4-Source Data8/INPUT ICP0 -2 2022.01.15_17.20.00_Ch+Marker.jpg]

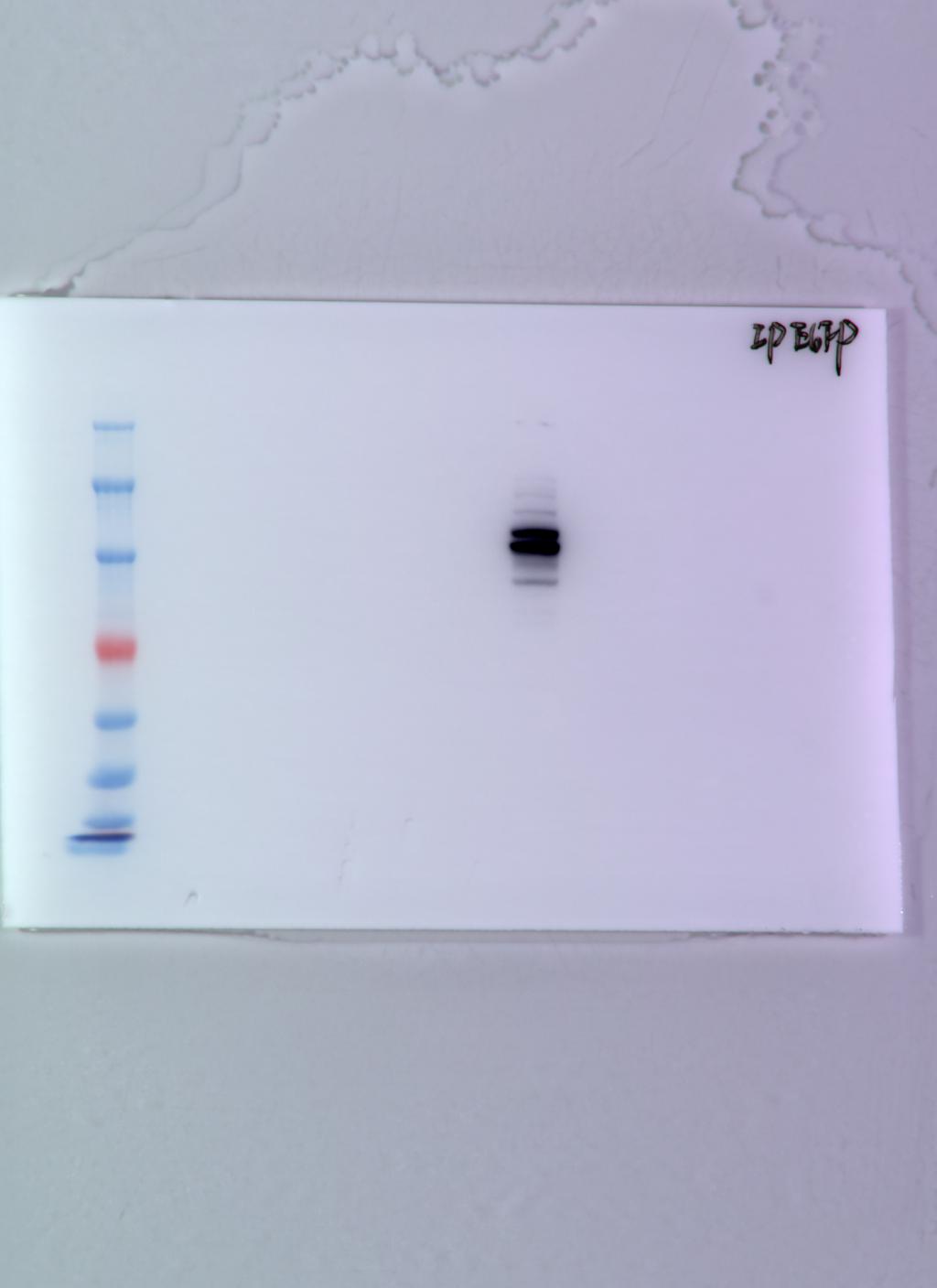

Supplement: Figure 4—source data 2. [file elife-110309-fig4-data2.zip › Figure4-Source Data8/IP EGFP 0-1 2022.01.15_17.01.17_Ch+Marker.jpg]

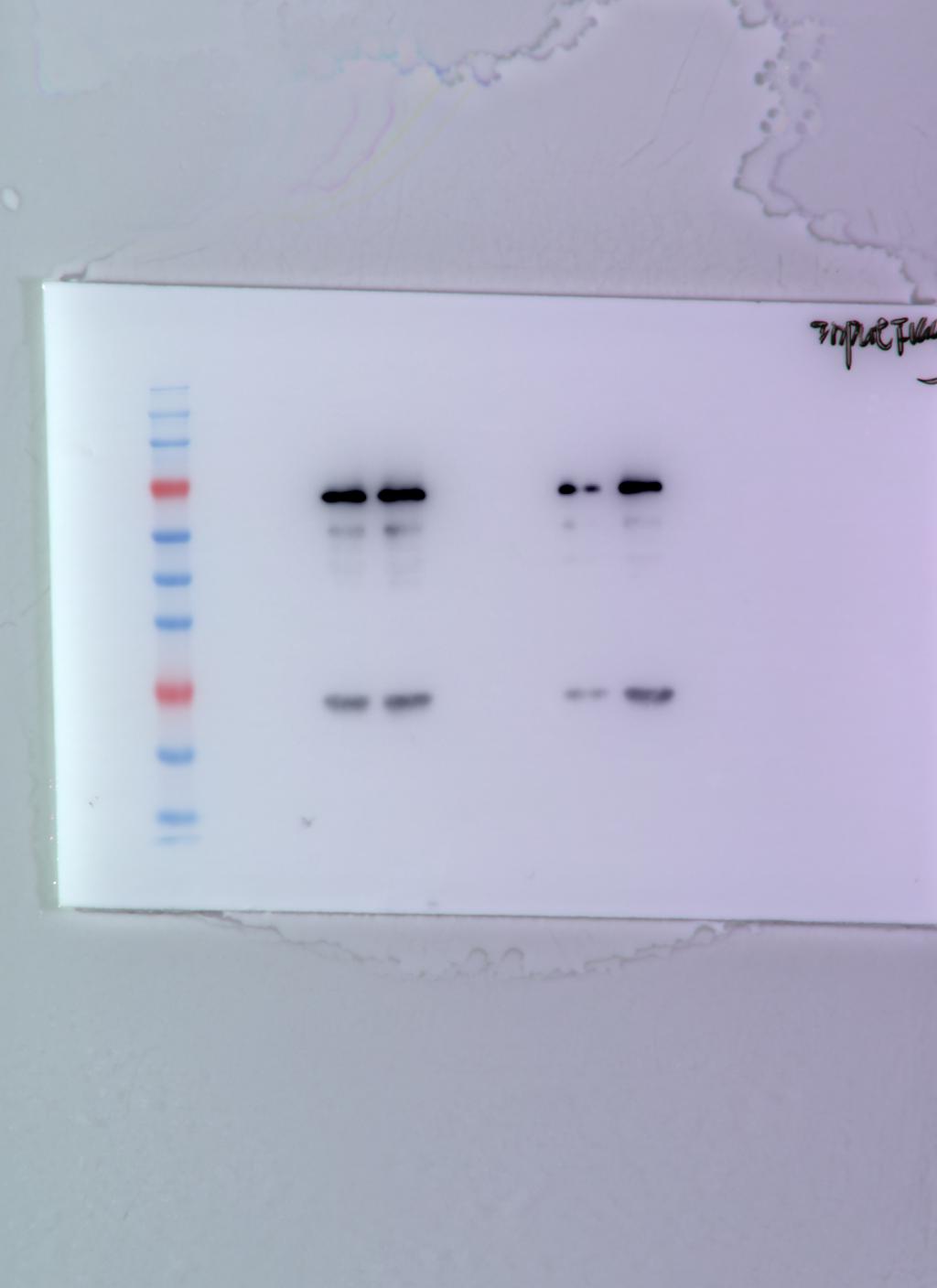

Supplement: Figure 4—source data 2. [file elife-110309-fig4-data2.zip › Figure4-Source Data8/IP FLAG 0-2 2022.01.15_17.06.16_Ch+Marker.jpg]

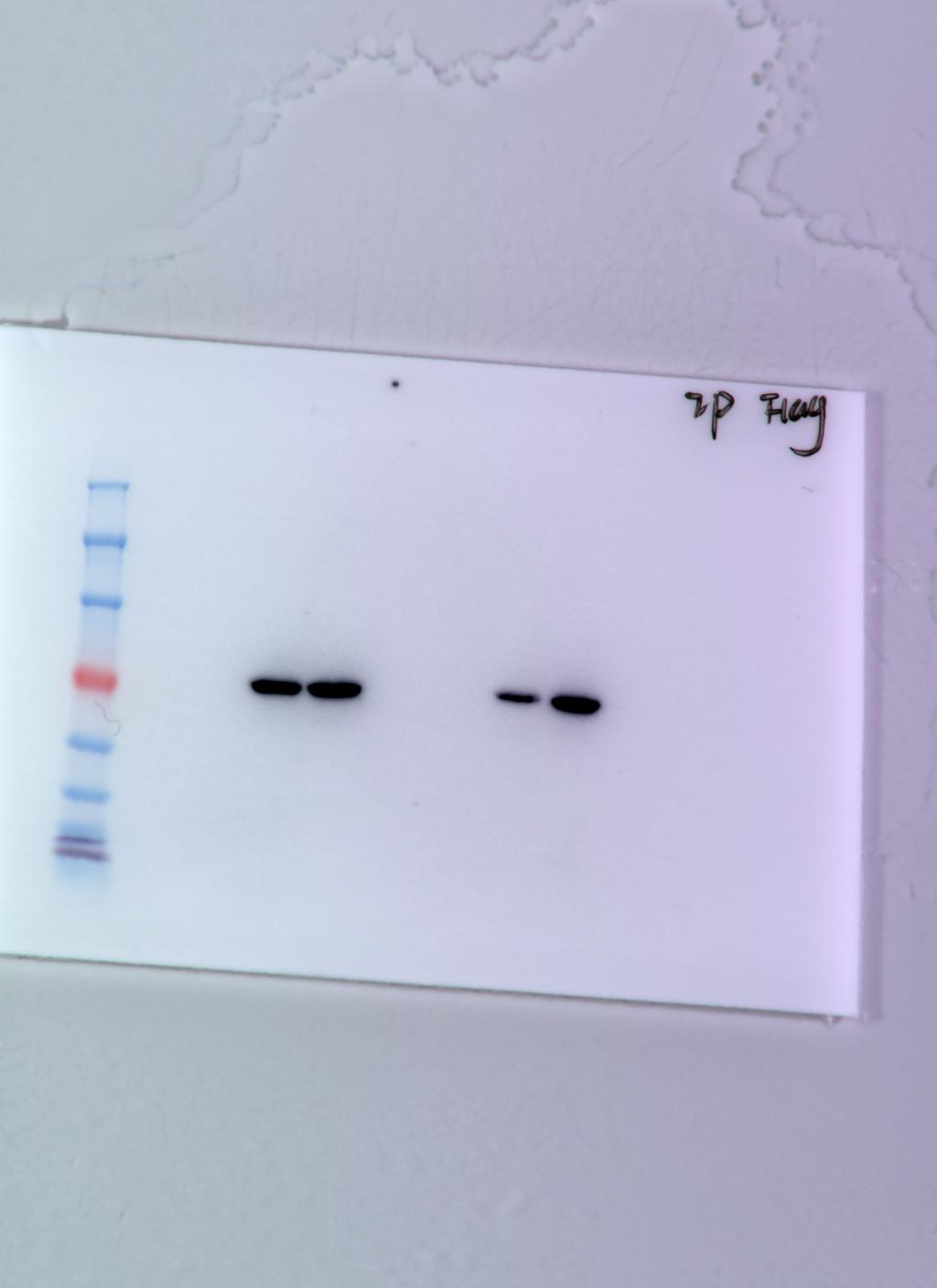

Supplement: Figure 4—source data 2. [file elife-110309-fig4-data2.zip › Figure4-Source Data8/IP-2 FLAG 0-1 2022.01.15_17.13.34_Ch+Marker.jpg]

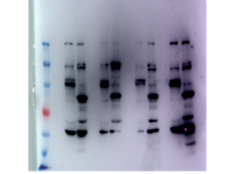

Supplement: Figure 4—source data 2. [file elife-110309-fig4-data2.zip › Figure4-Source Data10/INPUT GFP.tif]

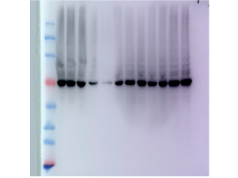

Supplement: Figure 4—source data 2. [file elife-110309-fig4-data2.zip › Figure4-Source Data10/INPUT HDAC1.tif]

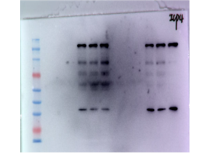

Supplement: Figure 4—source data 2. [file elife-110309-fig4-data2.zip › Figure4-Source Data10/INPUT ICP4.tif]

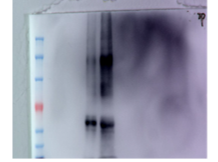

Supplement: Figure 4—source data 2. [file elife-110309-fig4-data2.zip › Figure4-Source Data10/IP UB.tif]

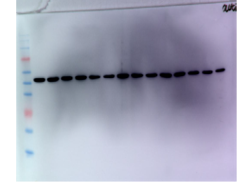

Supplement: Figure 4—source data 2. [file elife-110309-fig4-data2.zip › Figure4-Source Data2/ACTIN.tif]

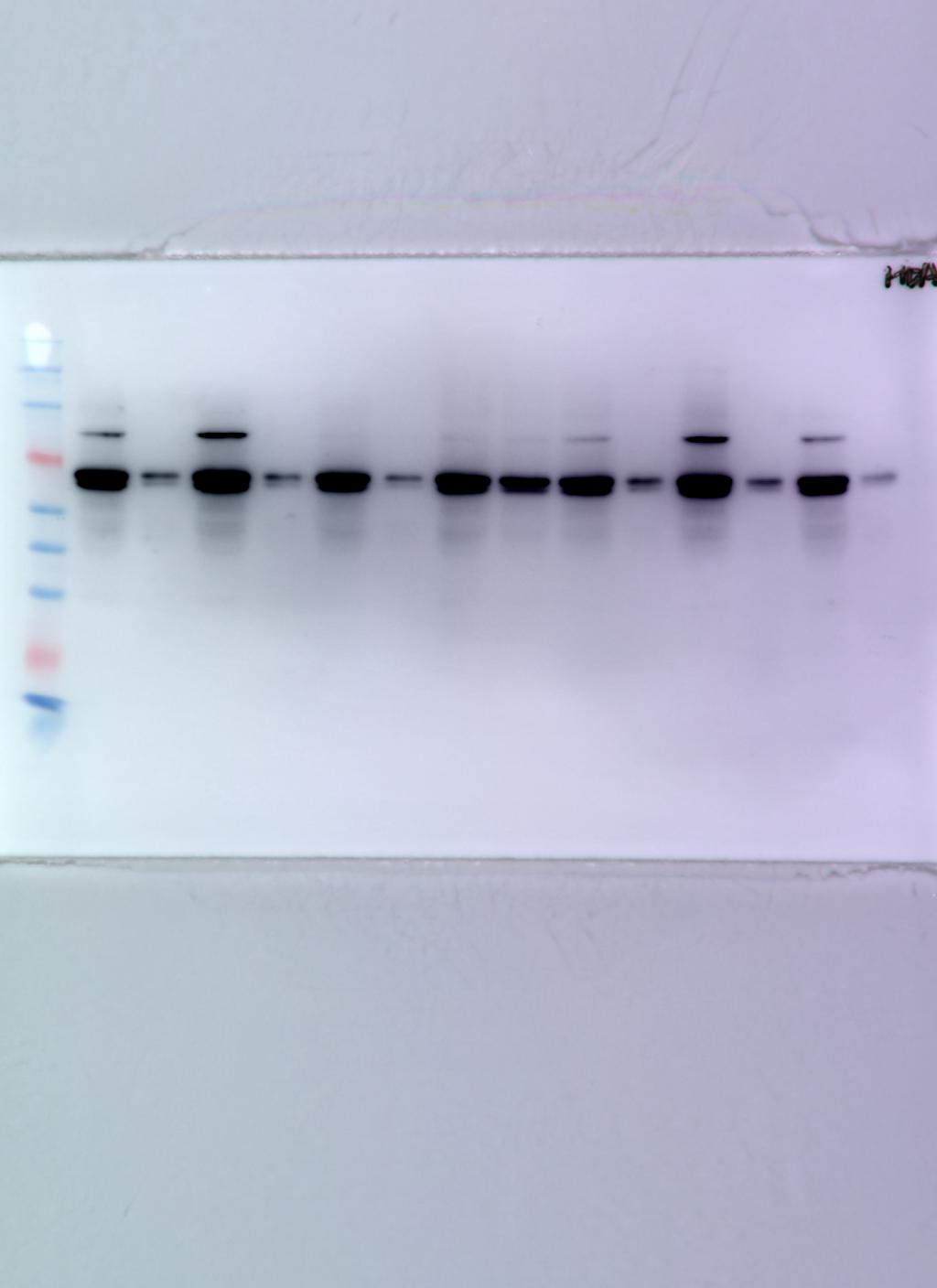

Supplement: Figure 4—source data 2. [file elife-110309-fig4-data2.zip › Figure4-Source Data2/HDAC1 0-2 2022.08.05_16.15.37_Ch+Marker.jpg]

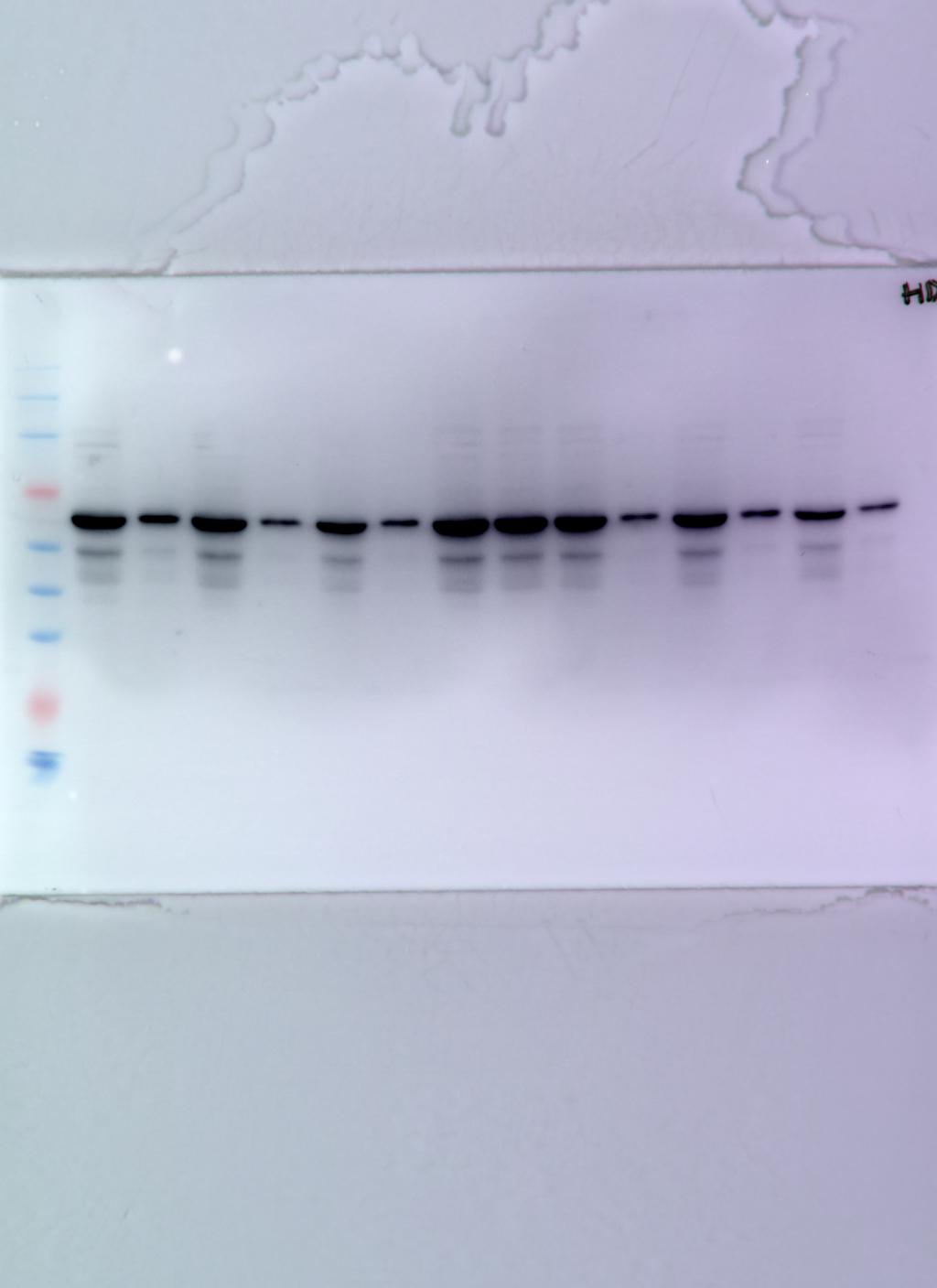

Supplement: Figure 4—source data 2. [file elife-110309-fig4-data2.zip › Figure4-Source Data2/HDAC2 0-4 2022.08.05_16.31.31_Ch+Marker.jpg]

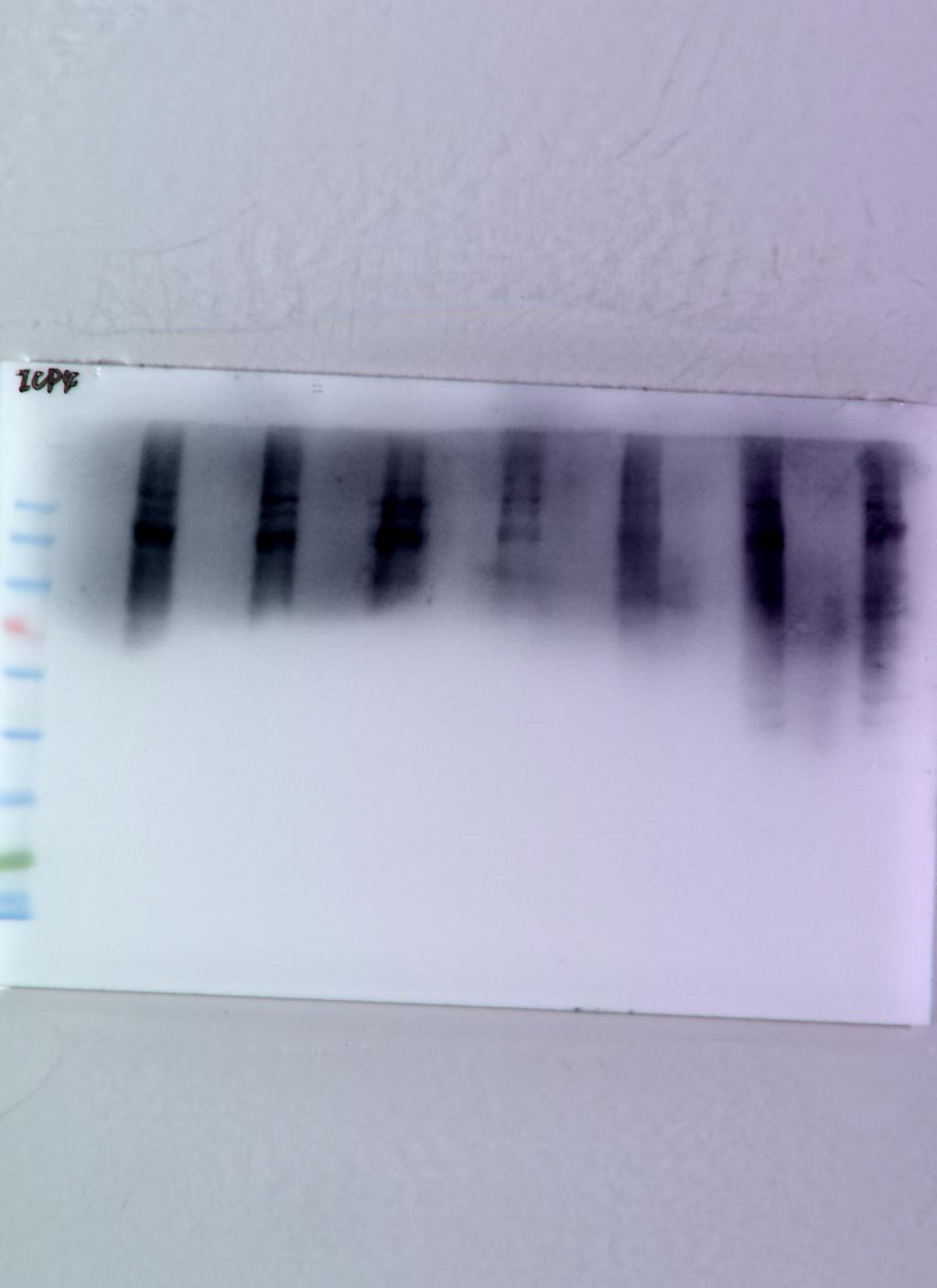

Supplement: Figure 4—source data 2. [file elife-110309-fig4-data2.zip › Figure4-Source Data2/ICP4 0-4 2026.04.07_09.25.38_Ch+Marker.jpg]

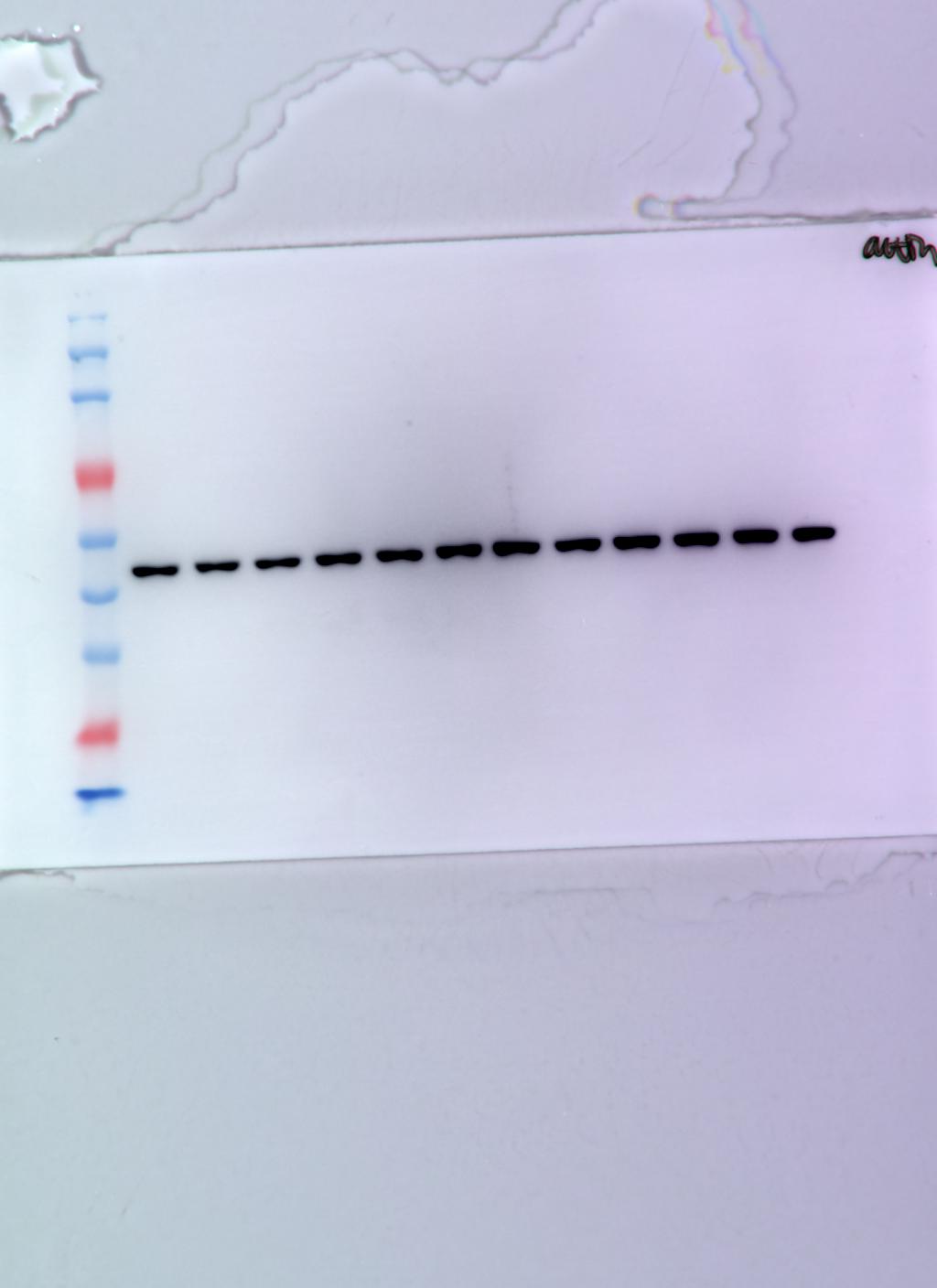

Supplement: Figure 5—source data 2. [file elife-110309-fig5-data2.zip › Figure5-Source Data2/ACTIN 0-4 2021.10.21_15.24.09_Ch+Marker.jpg]

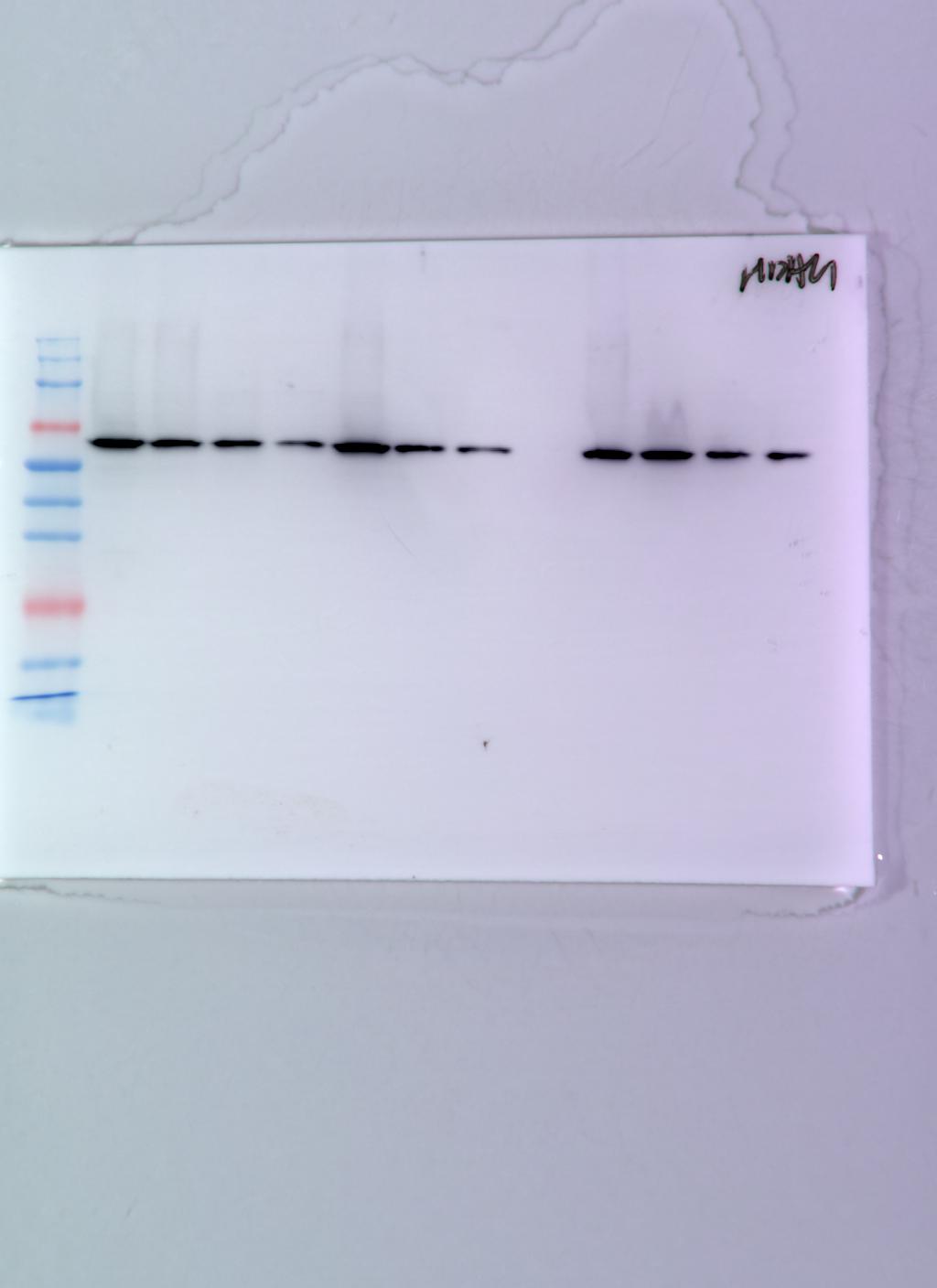

Supplement: Figure 5—source data 2. [file elife-110309-fig5-data2.zip › Figure5-Source Data2/HDAC1 0-6 2021.10.22_15.49.23_Ch+Marker.jpg]

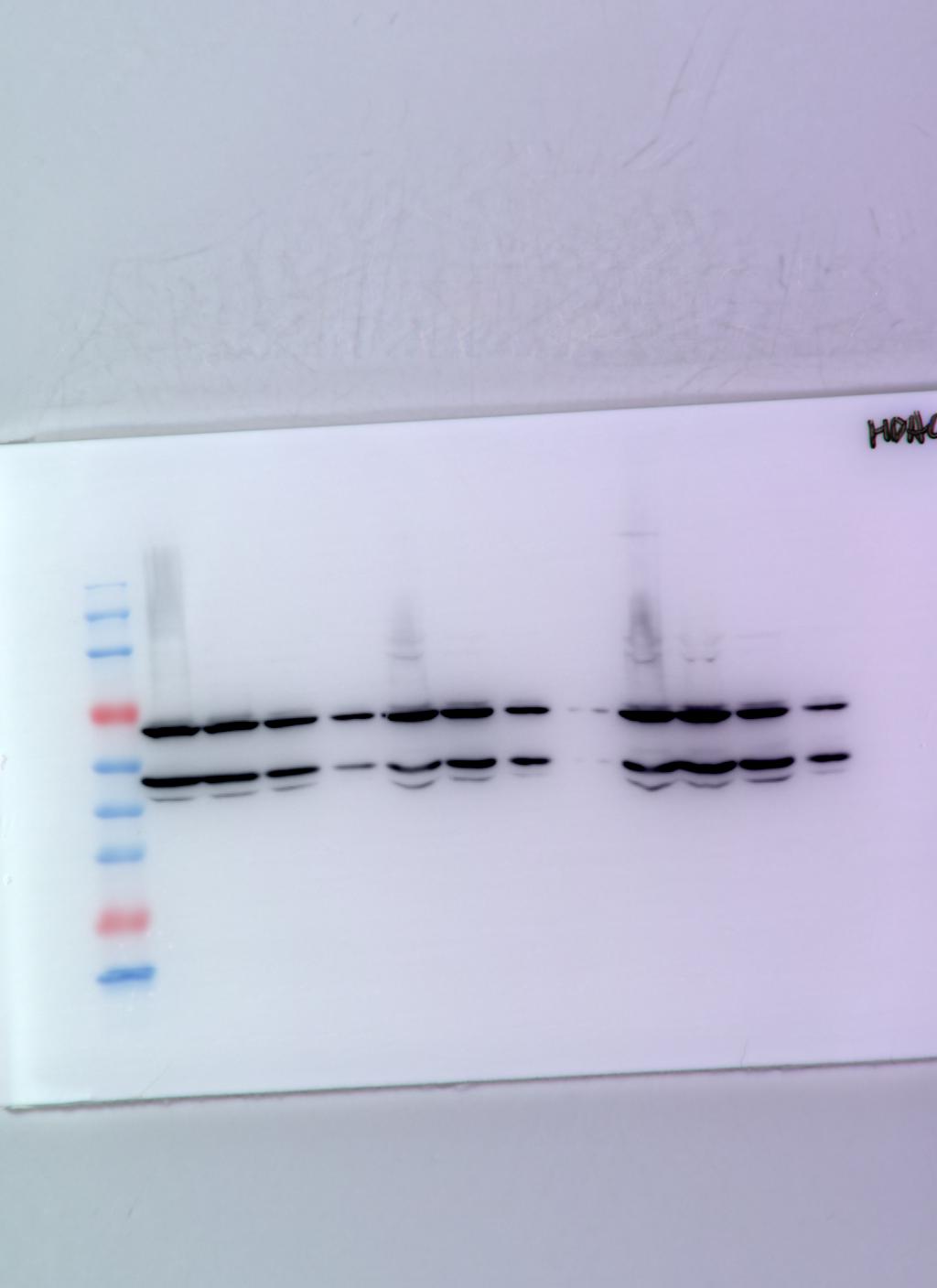

Supplement: Figure 5—source data 2. [file elife-110309-fig5-data2.zip › Figure5-Source Data2/HDAC2 0 2021.10.21_15.51.57_Ch+Marker.jpg]

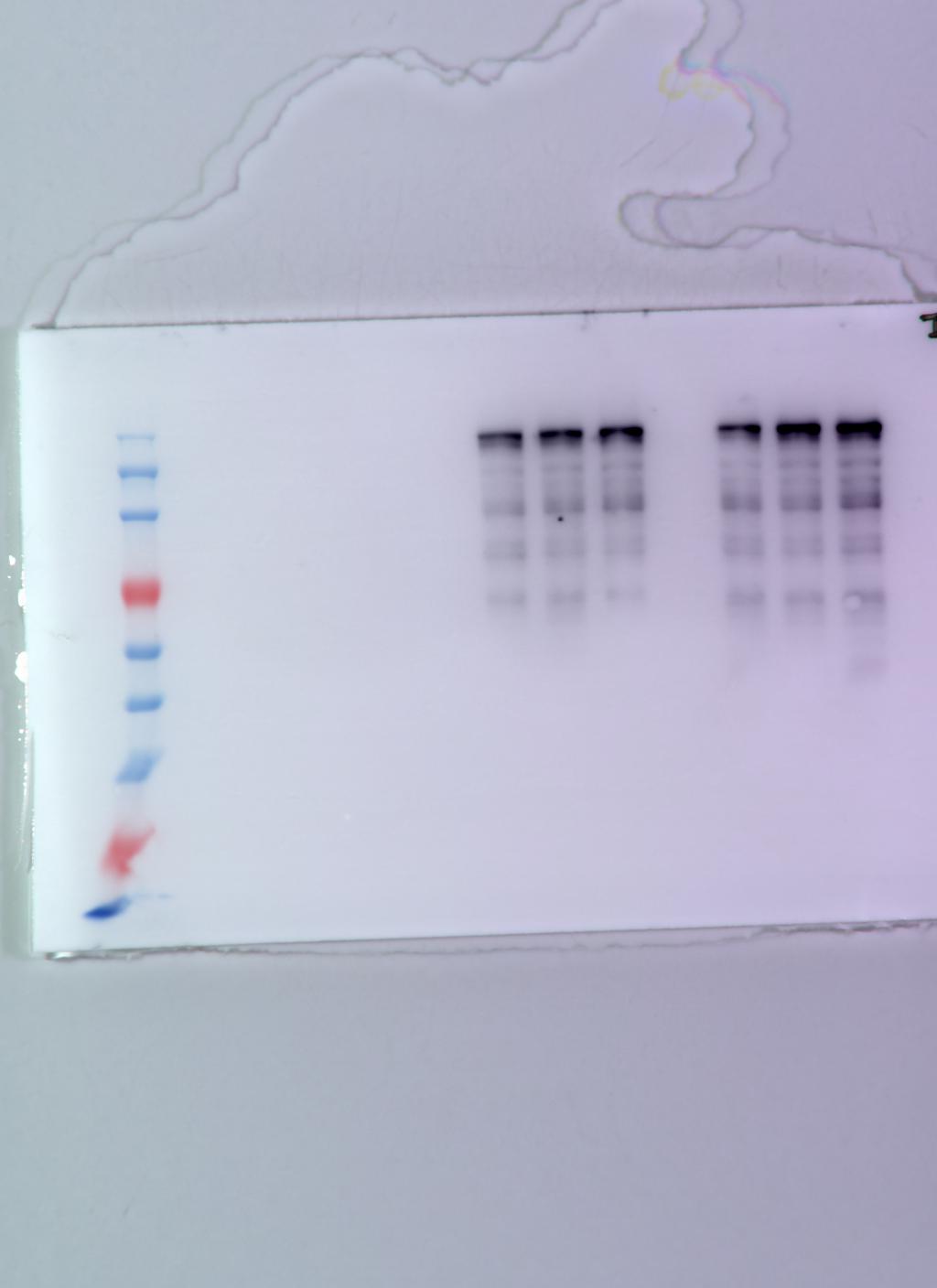

Supplement: Figure 5—source data 2. [file elife-110309-fig5-data2.zip › Figure5-Source Data2/ICP4 0-4 2021.10.21_15.40.31_Ch+Marker.jpg]

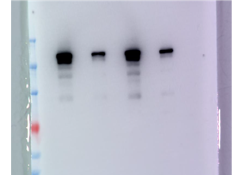

Supplement: Figure 5—source data 2. [file elife-110309-fig5-data2.zip › Figure5-Source Data4/INPUT ICP4.tif]

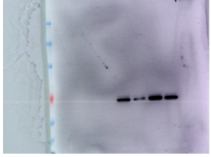

Supplement: Figure 5—source data 2. [file elife-110309-fig5-data2.zip › Figure5-Source Data4/IP FLAG.tif]

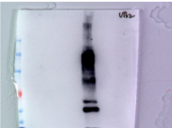

Supplement: Figure 5—source data 2. [file elife-110309-fig5-data2.zip › Figure5-Source Data4/IP UB.tif]

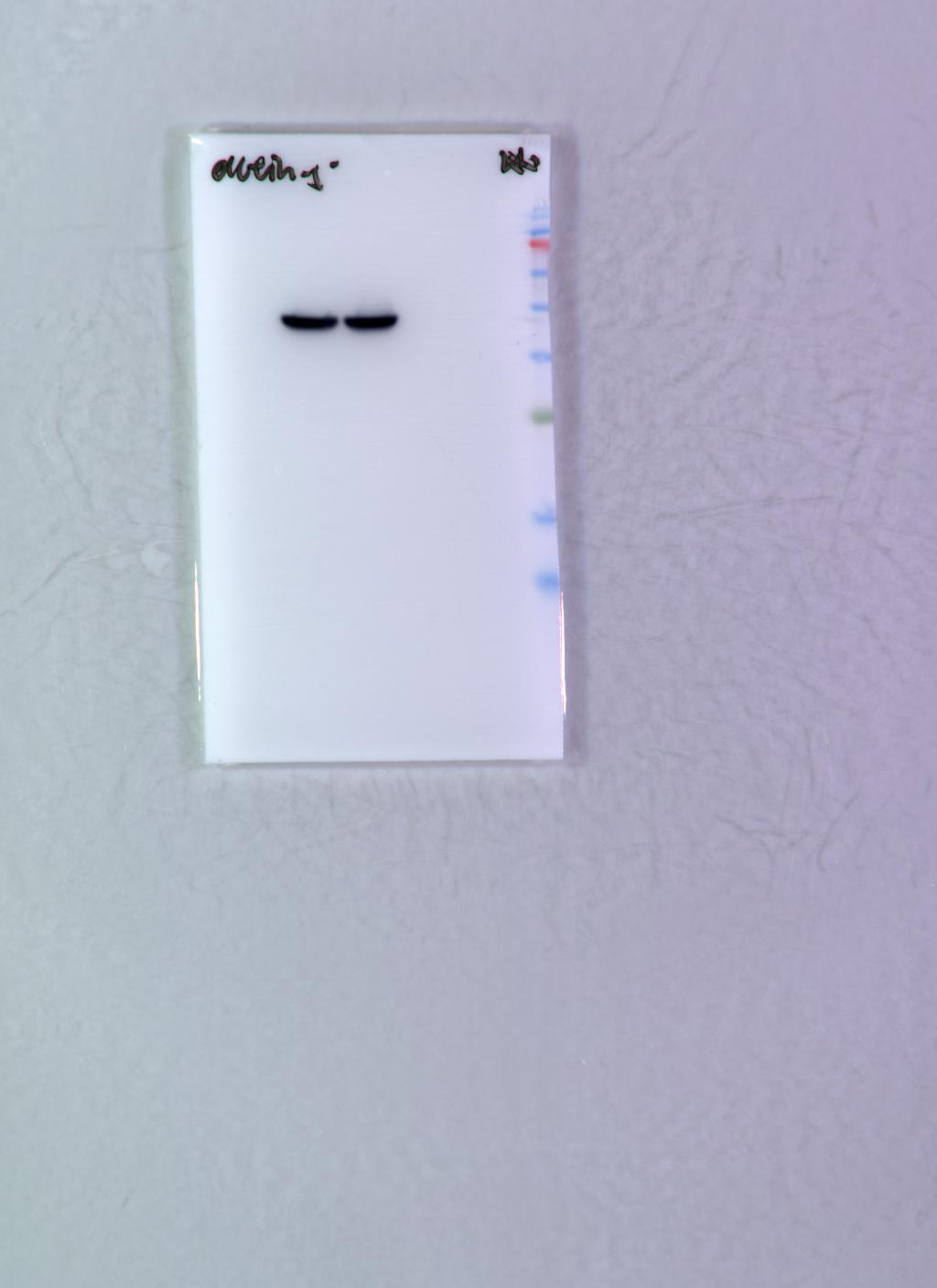

Supplement: Figure 5—source data 2. [file elife-110309-fig5-data2.zip › Figure5-Source Data6/ACTIN 4-1 2026.03.24_17.37.54_Ch+Marker.jpg]

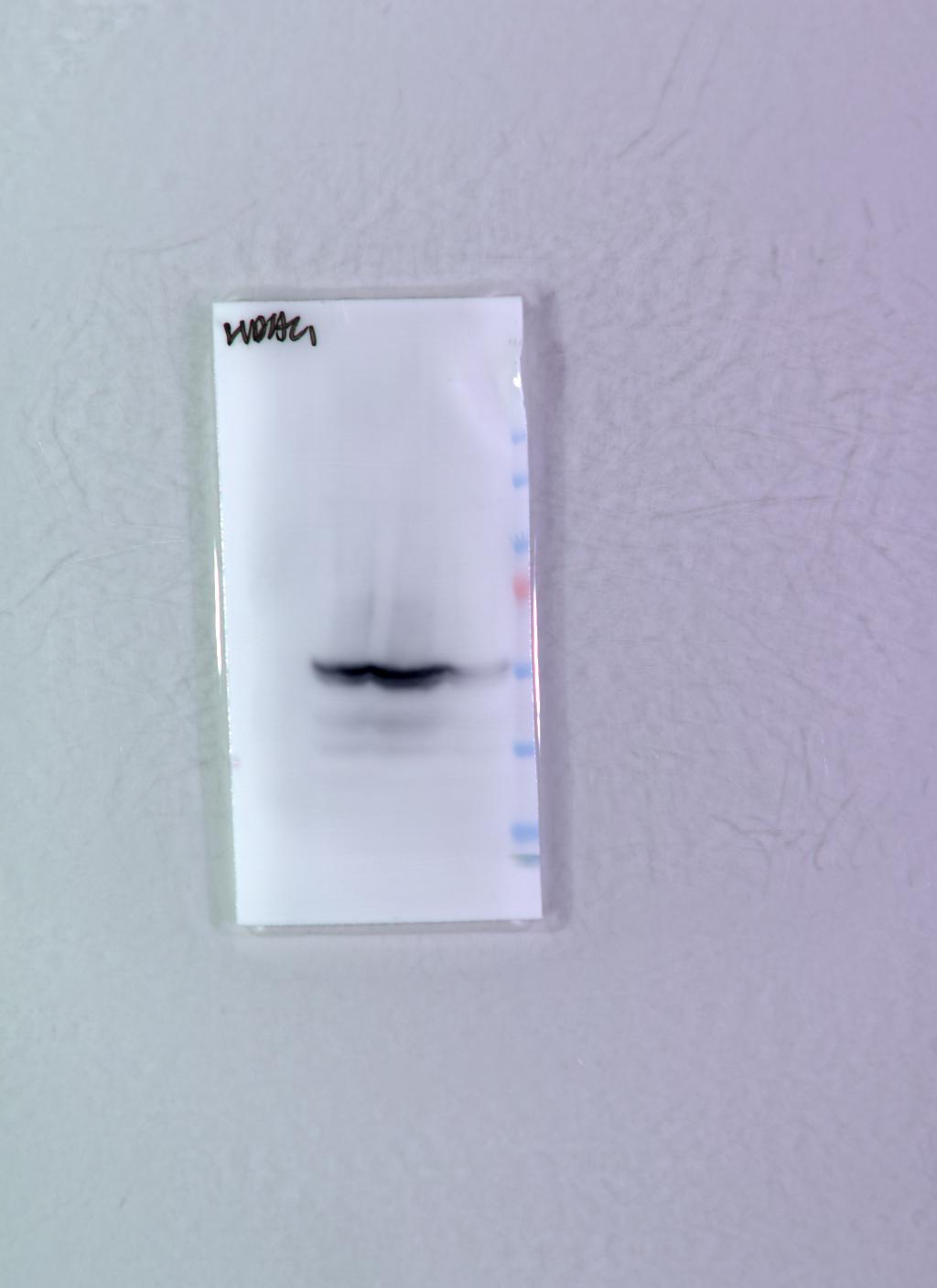

Supplement: Figure 5—source data 2. [file elife-110309-fig5-data2.zip › Figure5-Source Data6/HDAC1 1-4 2026.03.24_19.04.23_Ch+Marker.jpg]

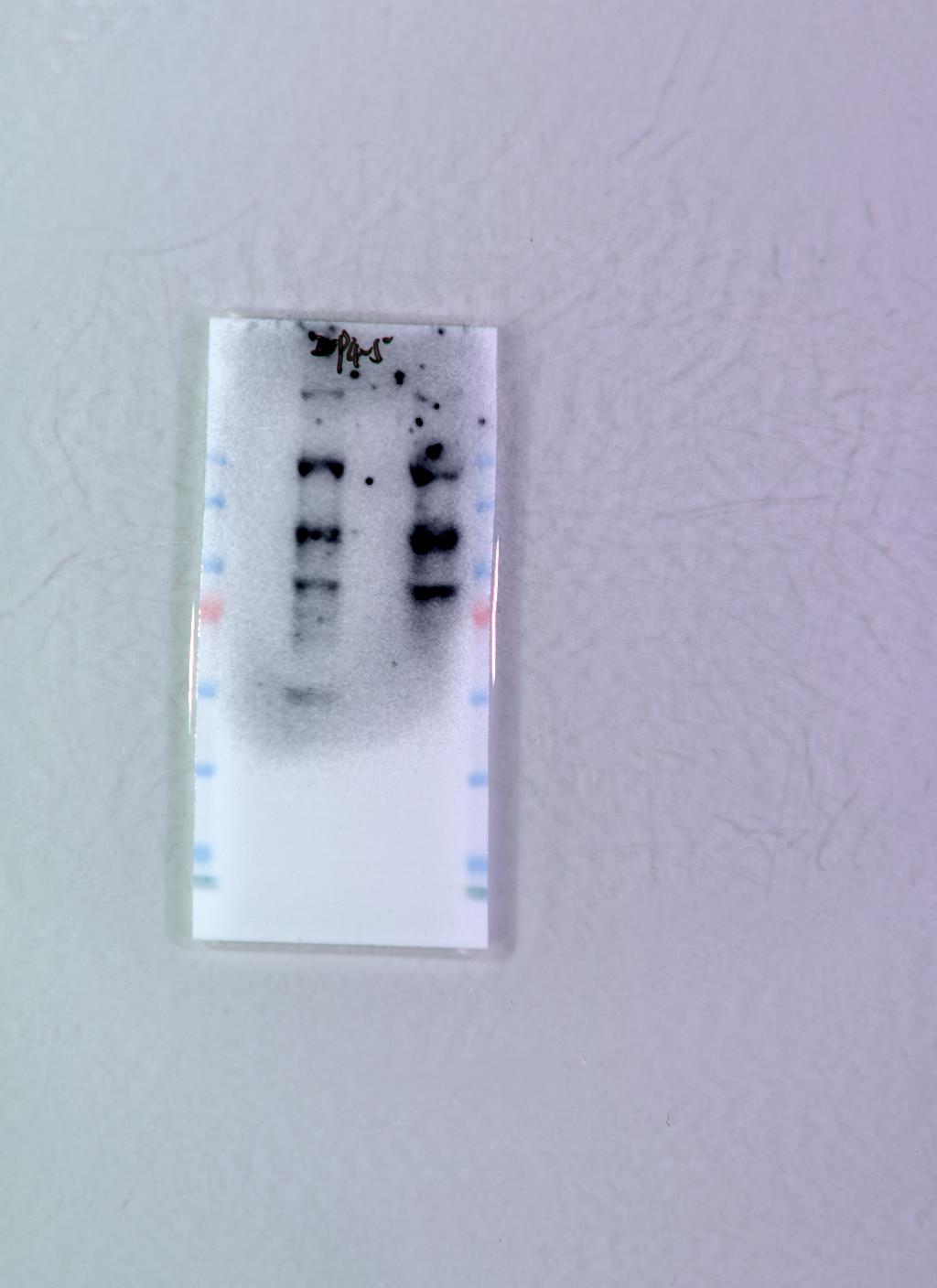

Supplement: Figure 5—source data 2. [file elife-110309-fig5-data2.zip › Figure5-Source Data6/ICP4 6-4 2026.03.24_21.24.46_Ch+Marker.jpg]

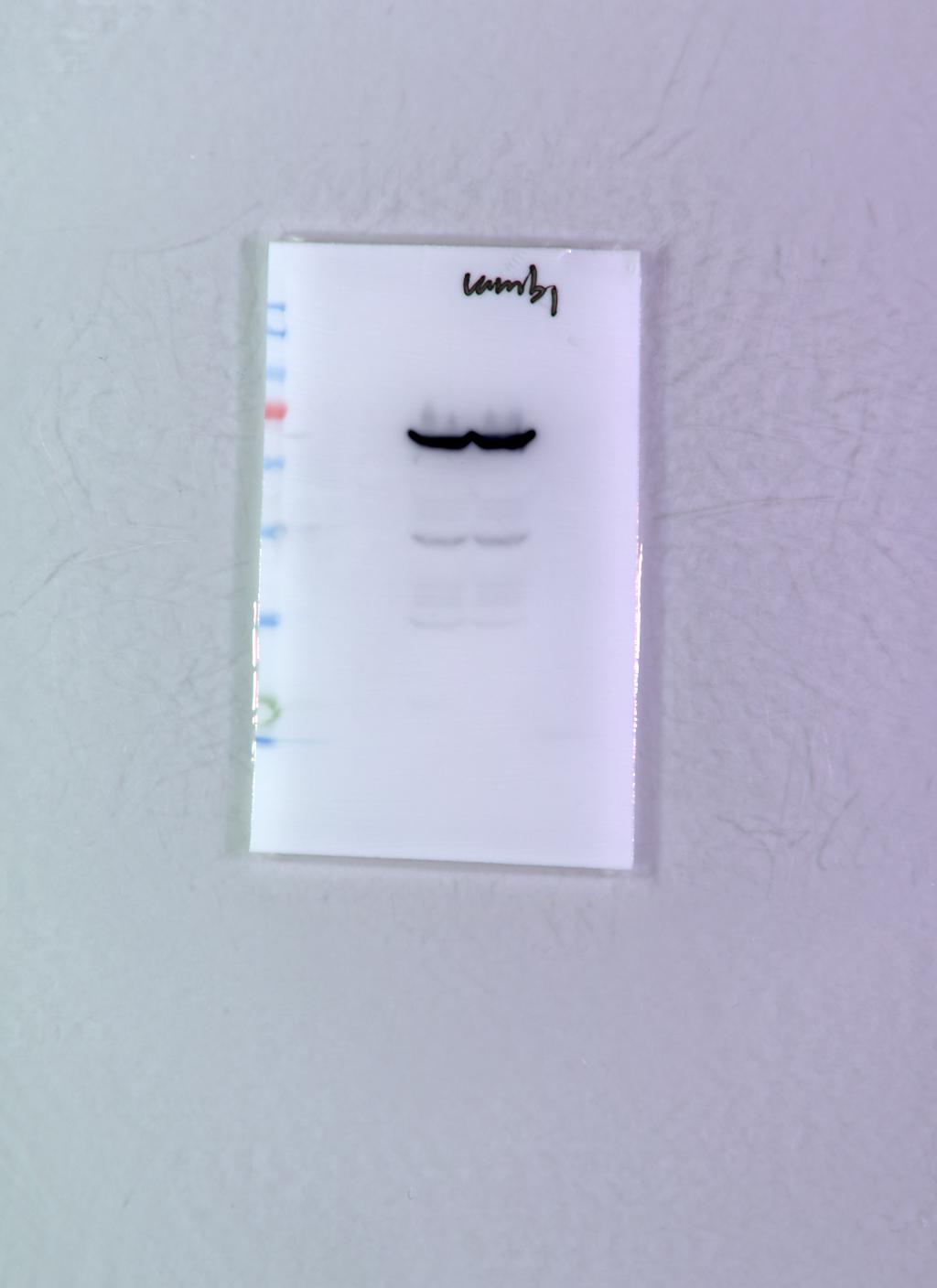

Supplement: Figure 5—source data 2. [file elife-110309-fig5-data2.zip › Figure5-Source Data6/LAMIB1 0-2 2026.03.24_17.04.58_Ch+Marker.jpg]

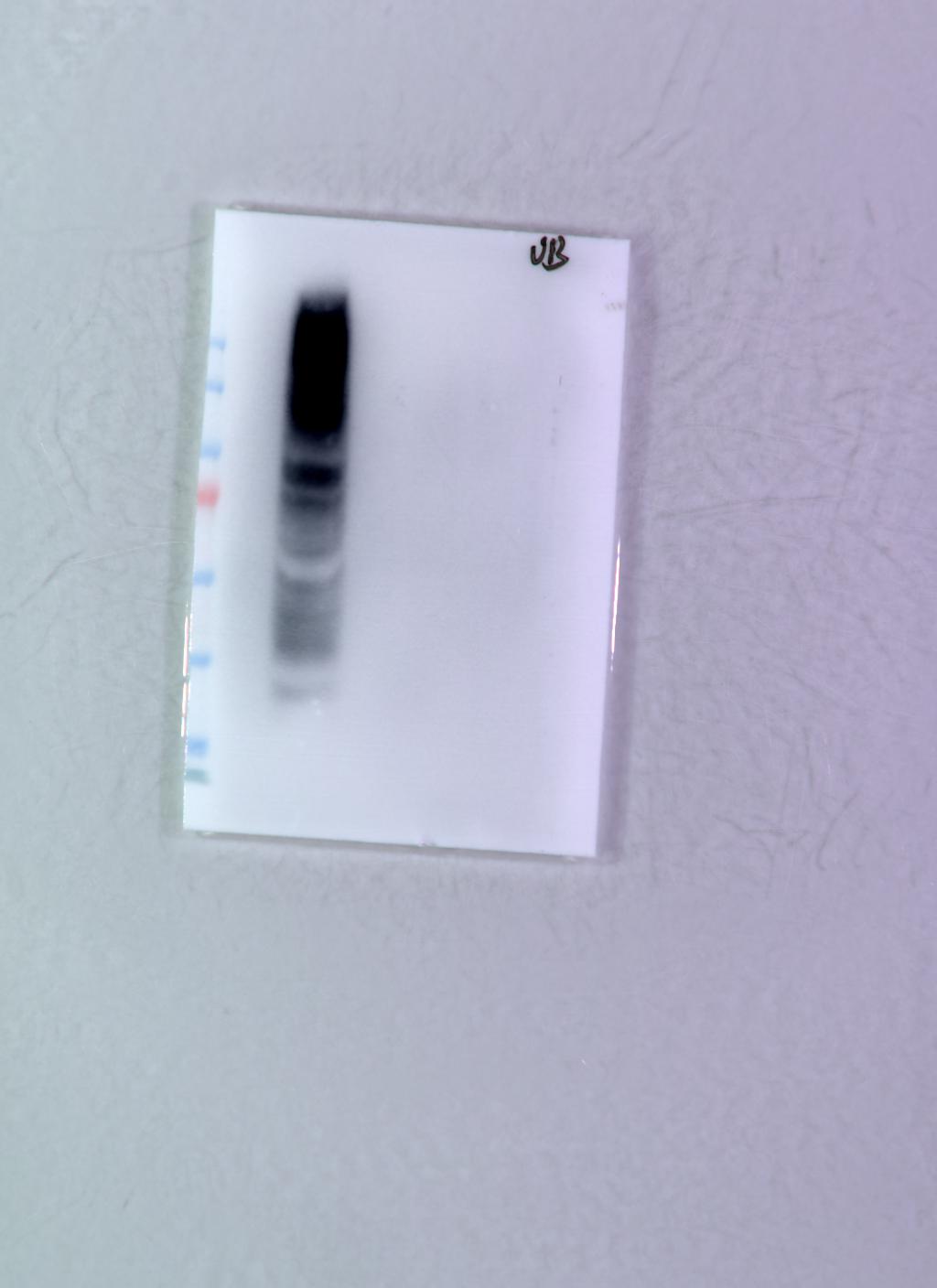

Supplement: Figure 5—source data 2. [file elife-110309-fig5-data2.zip › Figure5-Source Data6/UB 0-3 2026.03.24_18.44.16_Ch+Marker.jpg]

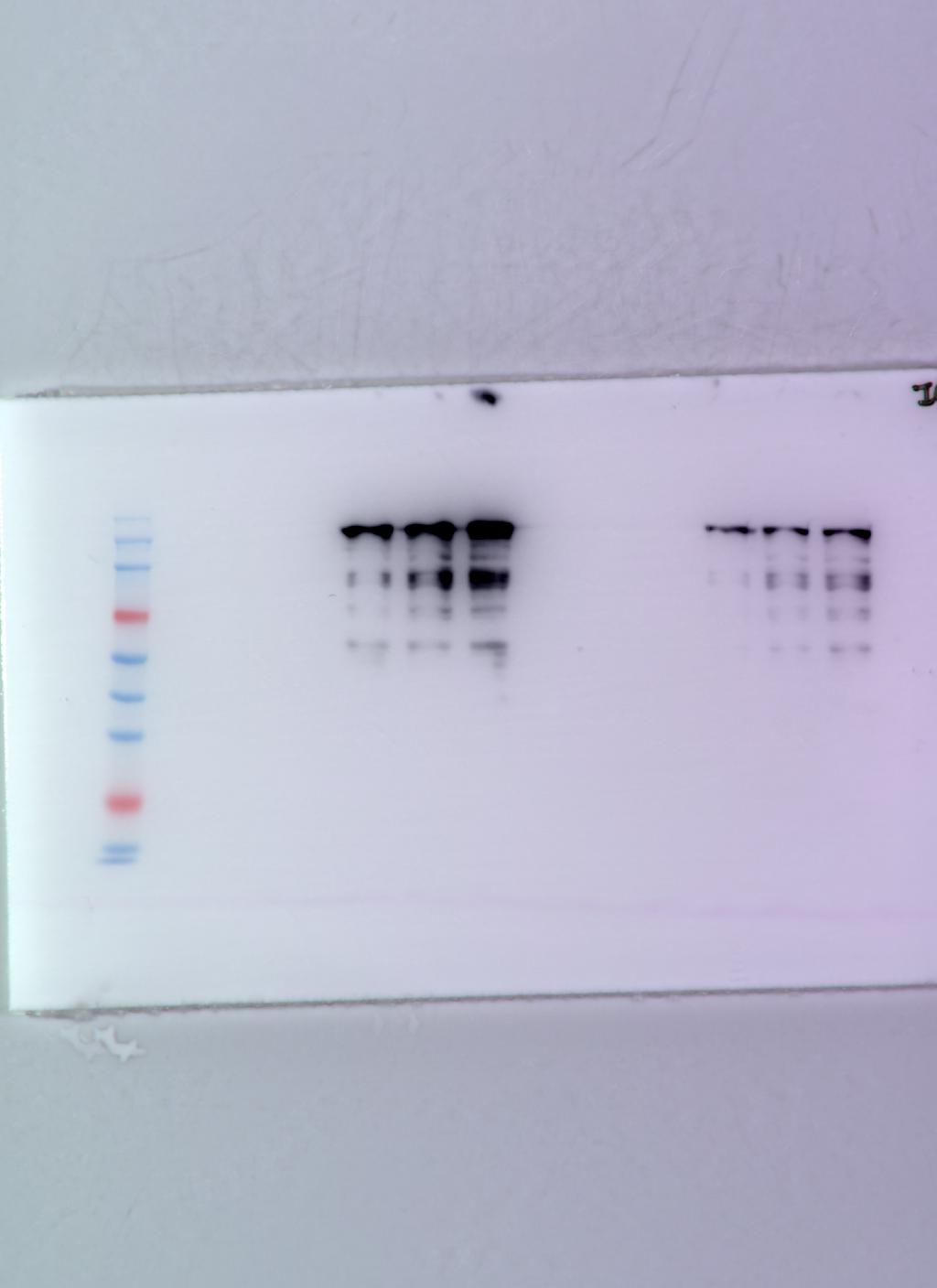

Supplement: Figure 5—source data 2. [file elife-110309-fig5-data2.zip › Figure5-Source Data8/ICP4 0 2021.12.13_17.07.52_Ch+Marker.jpg]

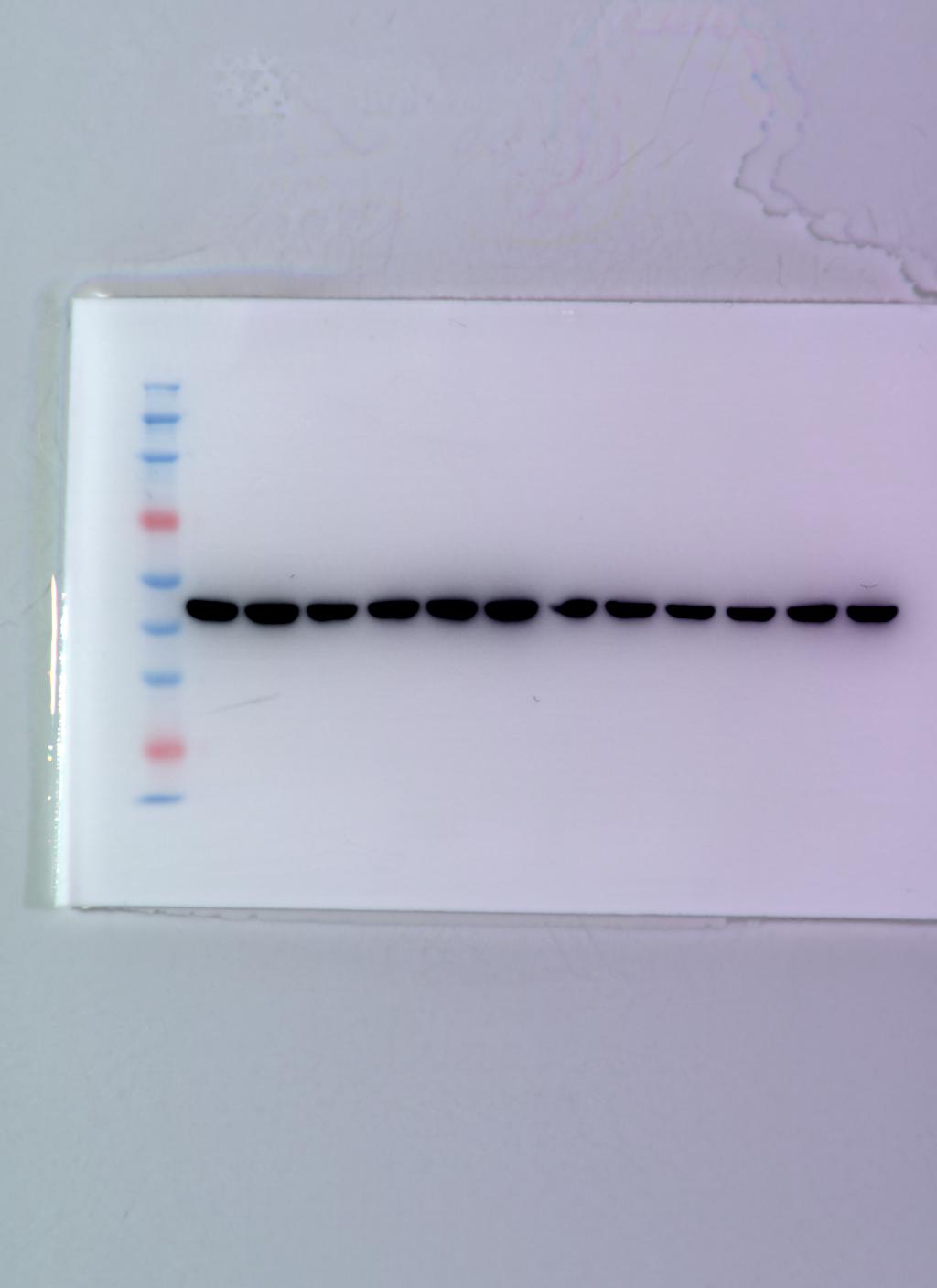

Supplement: Figure 5—source data 2. [file elife-110309-fig5-data2.zip › Figure5-Source Data8/LMB ACTIN 0-2 2021.12.06_23.37.28_Ch+Marker.jpg]

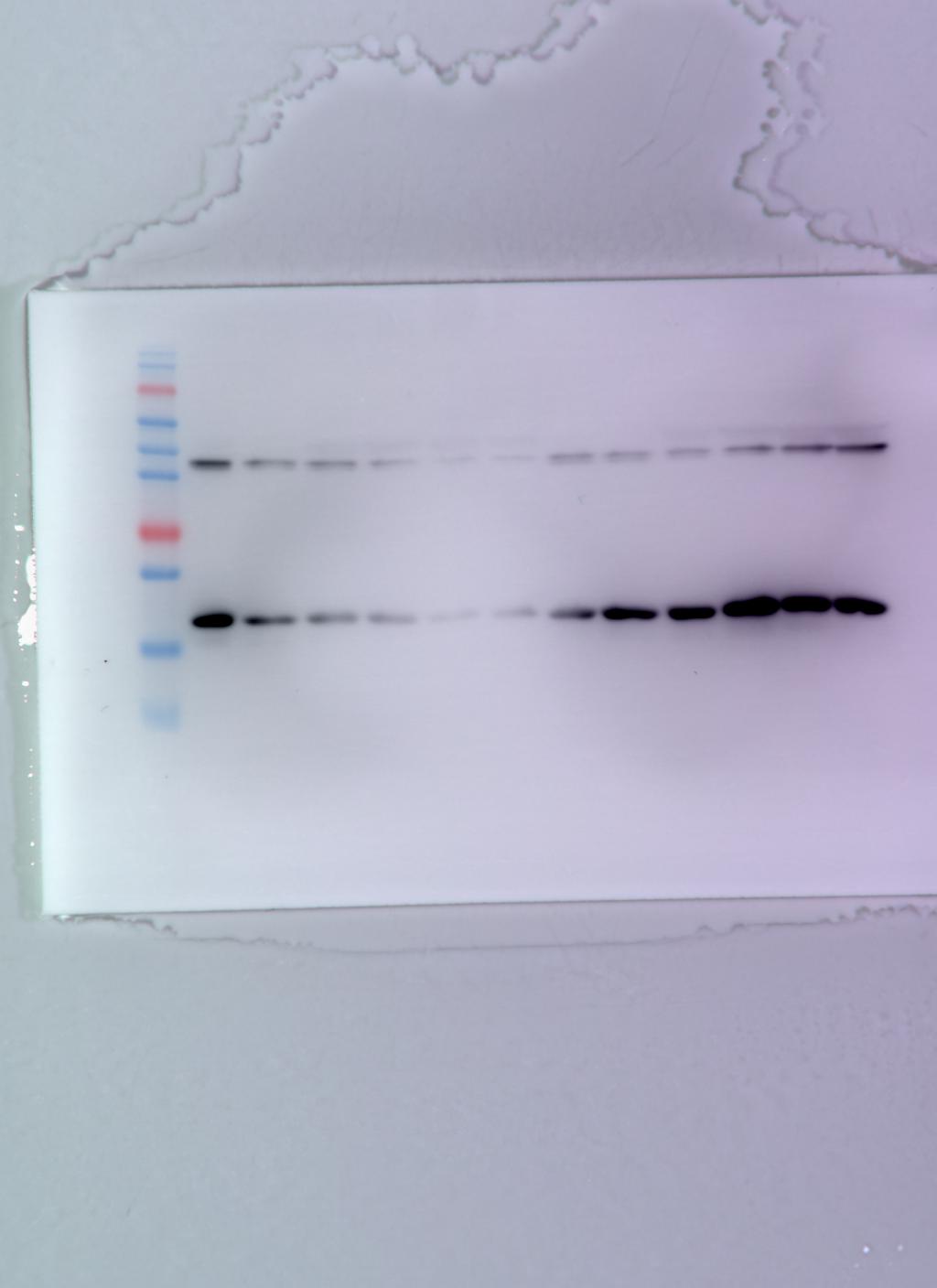

Supplement: Figure 5—source data 2. [file elife-110309-fig5-data2.zip › Figure5-Source Data8/LMB H2AX 0-5 2021.12.06_22.35.55_Ch+Marker.jpg]

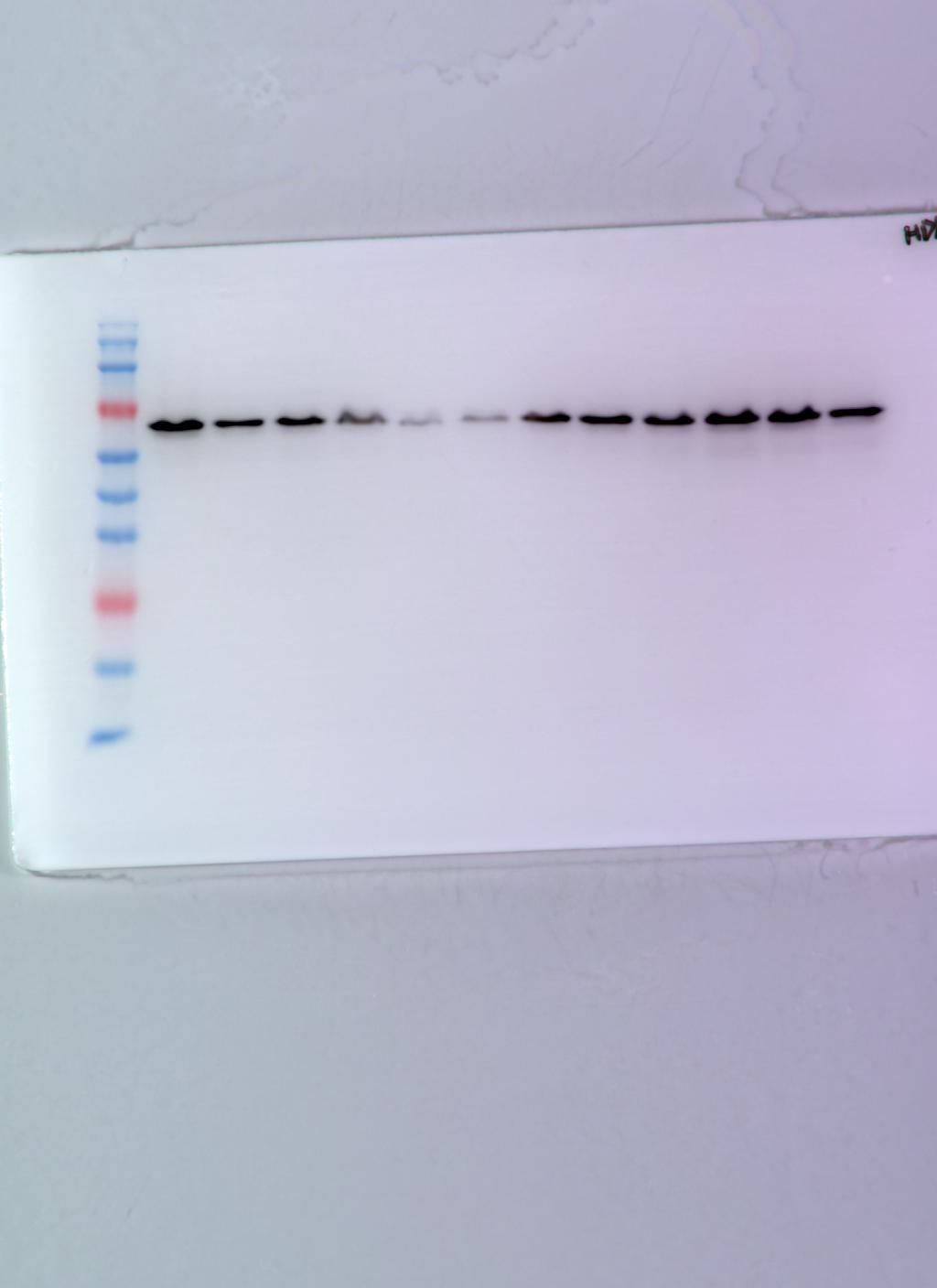

Supplement: Figure 5—source data 2. [file elife-110309-fig5-data2.zip › Figure5-Source Data8/LMB HDAC 0-3 2021.12.06_21.46.53_Ch+Marker.jpg]

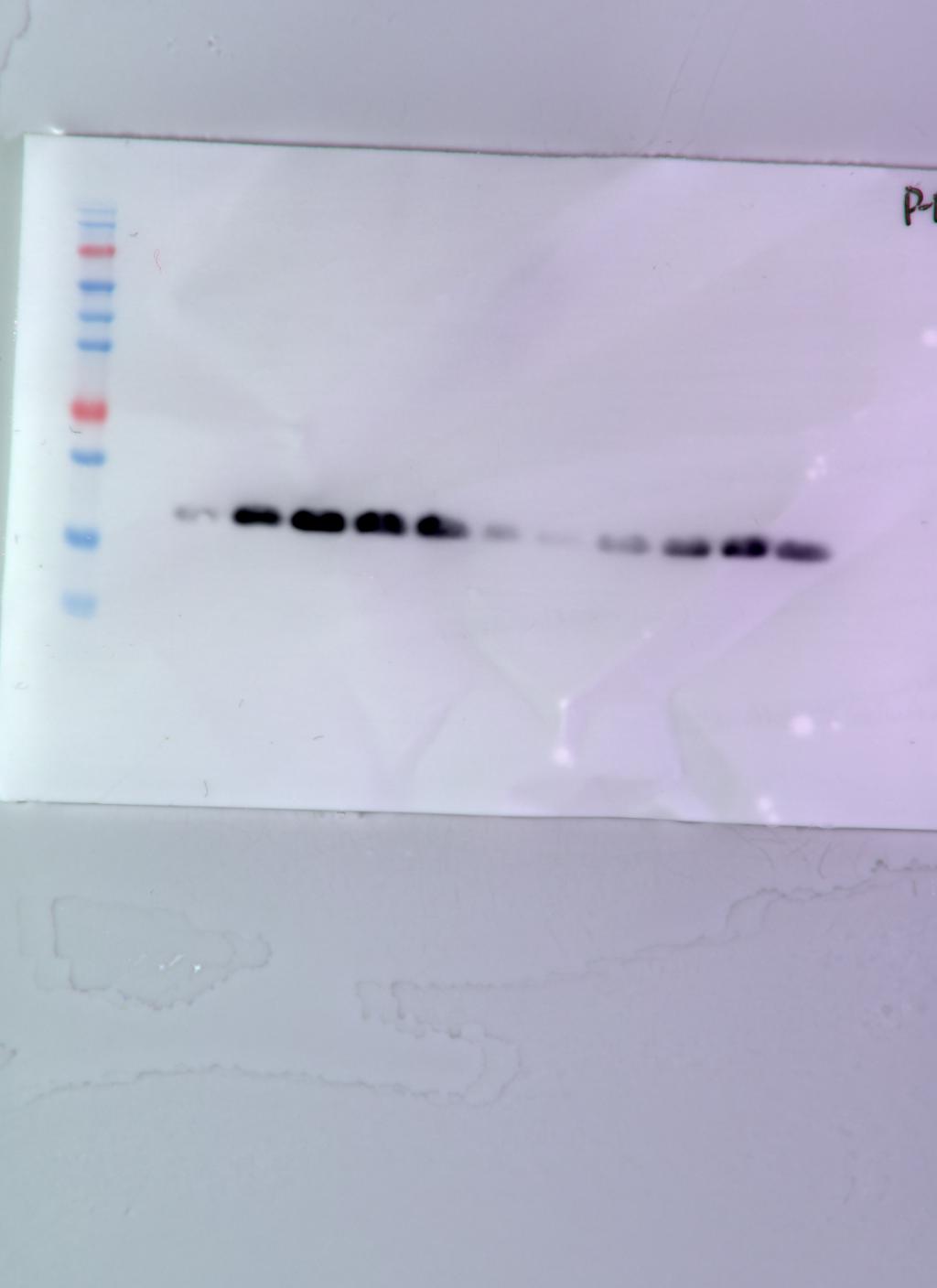

Supplement: Figure 5—source data 2. [file elife-110309-fig5-data2.zip › Figure5-Source Data8/LMB P-H2AX 0-2 2021.12.06_21.13.34_Ch+Marker.jpg]
