# Supplementary material for: The targeted cytosolic degradation of class I histone deacetylases is essential for efficient alphaherpesvirus replication
Source: eLife. 2026 Jul 9;15:RP110309. doi: 10.7554/eLife.110309 (PMC13349380; doi:10.7554/eLife.110309)

Figure 4, Source Data 3. Original membranes corresponding to Figure4, panel C. Rainbow pre-stained protein molecular weight standards were used as size references. Each immunoblot clearly displayed the marker bands with their assigned molecular weights, and the expected molecular weight of the target protein—as well as the catalog number and source of the corresponding primary antibody—was annotated on the respe ctive blot.

Fig. 4C

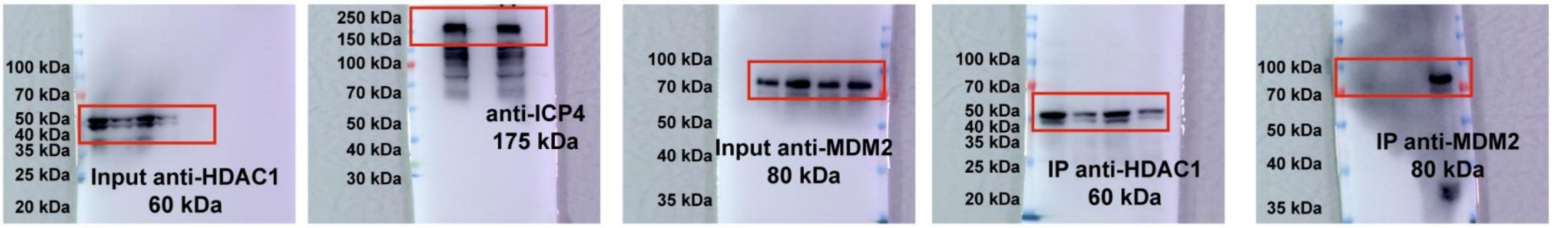

Supplement: Figure 4—source data 1. [file elife-110309-fig4-data1.zip › Figure4-SourceData3.pdf]
