# Supplementary material for: The targeted cytosolic degradation of class I histone deacetylases is essential for efficient alphaherpesvirus replication
Source: eLife. 2026 Jul 9;15:RP110309. doi: 10.7554/eLife.110309 (PMC13349380; doi:10.7554/eLife.110309)
Supplement: Supplementary file 1. [file elife-110309-supp1.docx]

**Supplementary File 1. List of shRNAs and primers used in this study.**

| **Genes** | **Forward (**5′-3′**)** | **Reverse (**5′-3′**)** |
| --- | --- | --- |
| Human Q-*HDAC1* | CATCGCTGTGAATTGGGCTG | CCCTCTGGTGATACTTTAGCAGT |
| Human Q-*HDAC2* | TGGCCTTTCTGAGCTGATTT | AGCCACTGAAACAAGACTTCA |
| Human Q-*β-actin* | CCTTGCACATGCCGGAG | GCACAGAGCCTCGCCTT |
| Sus Q-*β-actin* | CTGAACCCCAAAGCCAACCGT | TTCTCCTTGATGTCCCGCACG |
| HSV-1-*gB* | GGACATCAAGGCGGAGAACA | TTCTCCTTGAAGACCACCGC |
| HSV-1-*ICP0* | TCTGCATCCCGTGCATGAAAAC | TCACGCCCACTATCAGGTACAC |
| HSV-1-*ICP4* | ATCGCATCGGAA AGGGACACG | CCAAGGTGCTTACCCGTGCAA A |
| siHDAC1 | GGCUAGUGUUAUAGAUUAAGC | UUAAUCUAUAACACUAGCCUU |
| siHDAC2 | GGUCAAUAAGACCAGAUAACA | UUAUCUGGUCUUAUUGACCGU |
| siControl | UUCUCCGAACGUGUCACGUTT | ACGUGACACGUUCGGAGAATT |
| PRV-*gB* | CTCGCCATCGTCAGCAA | GCTGCTCCTCCATGTCCTT |
| Sus Q-*HDAC1* | TGACGAGTCCTATGAGGCCA | CAAACTCCACACACTTGGCG |
| Sus Q-*HDAC2* | CCCCATAAAGCCACTGCTGA | AGCCACCAGTTGAAAGCTGA |
| Human Q-*PIRH2* | TATCCTGGGGGAAGGATCGG | TCATGACACAAGCGGCAAGT |
| Human sh-PIRH2 | AATGTAACTTATGCCTAGCTA | TAGCTAGGCATAAGTTACATT |
| Human sh-KCTD11 | GGCACATCCTCAATTTCCTGA | TCAGGAAATTGAGGATGTGCC |
| Human Q-*KCTD11* | GATGTAGATGTCAGCCCCCG | GTGCAGAAAAGGTTGGCTCG |
| Human sh-MDM2 | GCCAGAGTGAGTCAGACAAGT | ACTTGTCTGACTCACTCTGGC |
| Human Q-*MDM2* | ACCCTGGTTAGACCAAAGCC | TGGCACGCCAAACAAATCTC |
| Human sh-CHFR | GCAACCAGAGGTTTGACATGG | CCATGTCAAACCTCTGGTTGC |
| Human Q-*CHFR* | TGTGTTCCATGGGACCAAAGAT | ACGTCGATGTTGATGGCTGT |
| Human sh-UHRF1 | GCCAGAGTGAGTCAGACAAGT | ACTTGTCTGACTCACTCTGGC |
| Human Q-*UHRF1* | AACAGCTCCTGGATCTTCCG | GGTTTCATCGCCATCCCCA |
| Human sh-TRIM46 | GGTGAGGATATGCAGACCTTC | GAAGTCTGCATATCCTCACC |
| Human Q-*TRIM46* | ACGGCGAATACAGTGAAG | GCTGGTCCTTGCTGATAG |
