# Supplementary material for: The targeted cytosolic degradation of class I histone deacetylases is essential for efficient alphaherpesvirus replication
Source: eLife. 2026 Jul 9;15:RP110309. doi: 10.7554/eLife.110309 (PMC13349380; doi:10.7554/eLife.110309)
Supplement: Peer review history

# Editor report: eLife Assessment

**DOI:** 10.7554/eLife.110309.4.sa0

**Authors:** John W Schoggins

**Affiliations:** The University of Texas Southwestern Medical Center United States

**Keywords:** Convincing, Valuable

This **valuable** study presents **convincing** data demonstrating that alpha herpesvirus triggers nuclear export of HDACs, which are then degraded in an MDM2-dependent manner. This virus-driven process leads to histone hyperacetylation and activation of the DNA damage response, which promotes viral replication.

# Referee report: Reviewer #1 (Public review)

**DOI:** 10.7554/eLife.110309.4.sa1

[Editors' note: this version has been assessed by the Reviewing Editor without further input from the original reviewers. The authors have addressed the comments raised in the previous round of review.]

Summary:

In this study, the authors propose that HSV-1 infection degrades the class I histone deacetylases HDAC1 and HDAC2. The MDM2 E3 ubiquitin ligase from the DNA damage response pathway is responsible for ubiquitinating these HDACs that are subsequently degraded via proteasomes. The authors hypothesize that HDAC degradation will cause hyperacetylation of viral chromatin and enable viral gene transcription.

Strengths:

The ubiquitination of HDAC1 and HDAC2 by Mdm2 and the mapping studies are clear.

# Referee report: Reviewer #2 (Public review)

**DOI:** 10.7554/eLife.110309.4.sa2

Summary:

The authors discovered that HDAC1/2 are degraded in HSV-1 and PRV infections. They attempted to establish a new mechanism by which HDAC1/2 are translocated to the cytoplasm to be degraded in HSV-1 infection, and the degradation causes changes in histone acetylation to affect the DDR pathway.

Strengths:

(1) Interesting findings of HDAC1/2 degradation during HSV-1 and PRV infection, and it may impact more than the virology field.

(2) Significant work to identify the ubiquitin site in HDAC1/2 and K63 linkage.

# Author comment: Author response

**DOI:** 10.7554/eLife.110309.4.sa3

**Authors:** Shengli Ming, Meng-Hua Du, Jia-Ming Yang, Ya-Di Guo, Jia-Jia Pan, Wei-Fei Lu, Jiang Wang, Lei Zeng, Bei-Bei Chu

**Affiliations:** Henan Agricultural University 郑州市 China; Henan Agricultural University Zhengzhou China; Tsinghua University beijing China; Henan Agricultural University Zhengzhou China; Henan Agricultural University Zhengzhou China; Henan Agricultural University Zhengzhou China; Henan Agricultural University Zhengzhou China; Henan Agricultural University Zhengzhou China; Henan Agricultural University Zhengzhou China

The following is the authors’ response to the previous reviews

**Public Reviews:**

**Reviewer #1 (Public review):**

Summary:

In this study, the authors propose that HSV-1 infection degrades the class I histone deacetylases HDAC1 and HDAC2. The MDM2 E3 ubiquitin ligase from the DNA damage response pathway is responsible for ubiquitinating these HDACs that are subsequently degraded via proteasomes. The authors hypothesize that HDAC degradation will cause hyperacetylation of viral chromatin and enable viral gene transcription.

Strengths:

The ubiquitination of HDAC1 & HDAC2 by Mdm2 and the mapping studies are clear.

Comments on revised version:

The authors enhanced their manuscript by more supportive data and providing clarification and the necessary corrections. However, a few more issues pertain:

(1) In Figure 4j at 2 h post-infection we typically see the input virus and not progeny virus production. The input seems to have about 1-log difference that is expected to impact the results.

We sincerely appreciate the reviewer's valuable comments regarding the timing notation. It should be noted that the "2 h" indicated in Figure 4j does not refer to two hours after the start of viral infection, but rather to two hours following medium replacement—after the virus has completed adsorption and internalization at 37°C (typically taking 2 hours), with this moment defined as the new time zero point (t = 0 h). Thus, this corresponds to approximately 4 hours post-infection (4 hpi). All subsequent sampling time points (4, 6, 12, and 24 h) are consistently defined according to this same system. This temporal framework aligns with previous studies: Nobe et al. (mBio 2025; DOI: 10.1128/mbio.00280-25) have clearly demonstrated that newly generated viral particles can be detected as early as 4 hours after HSV-1 infection, supporting the possibility of early progeny virus production at this time point in our experiment. We have accordingly revised the figure legend for Figure 4j to explicitly state the time reference ("t = 0 h defined as time of medium replacement post-adsorption") and added detailed procedural descriptions in the Methods section regarding adsorption, medium change, and sample collection time points to ensure clarity and reproducibility of the timing protocol.

(2) Figs 1A, 1E, 2H it seems unclear why ICP4 becomes detectable at 12 h post-infection in HeLa cells? How about other a-genes? How about other cells? ICP4 is typically detectable within 2-3 h post-infection.

We sincerely appreciate the valuable comments provided by the reviewers. Regarding the observation that ICP4 was detected only after 12 hours post-infection in HeLa cells, we re-evaluated our experimental conditions and reviewed relevant literature. The results indicate that at a higher multiplicity of infection (MOI = 5), ICP4 can indeed be reliably detected in HeLa cells as early as 2 hours post-infection (Author response image 1). Notably, Fouad S. El-Mayet et al. reported that under MOI = 1, ICP4 could not be detected until 8 hours after HSV-1 infection of mouse neuroblastoma Neuro-2A cells Figure 5A (Fouad S. El-Mayet et al., Antiviral Research, 2024, DOI: 10.1016/j.antiviral.2024.105870), although their early protein VP16 showed positive expression as early as 4 hours post-infection. This time difference is closely related to cell type: Neuro-2A is a highly susceptible neuronal cell line for HSV-1, exhibiting significantly faster viral gene expression kinetics compared to epithelial-derived HeLa cells. In contrast, HeLa cells are human cervical cancer epithelial cells with relatively low efficiency in initial transcriptional activation of HSV-1 and higher baseline expression levels of endogenous antiviral factors (such as interferon-stimulated genes), which may lead to a marked delay in the expression of early immediate-early genes like ICP4.

**Author response image 1.**

(3) In responses 2-2, Fig 5K: An infection without transfection has not been included. This is important to understand kinetics of infection in transfected cells.

We sincerely appreciate the reviewer's insightful identification of this critical oversight. In all relevant experiments, we have strictly included empty vector transfection controls—serving as a baseline reference for each transfection group to eliminate potential influences from the transfection procedure itself and the vector background on viral replication, gene expression, and signaling pathways. The failure to clearly label this control in previous figure legends and main figures was indeed an omission in our presentation; we have now fully addressed this in the revised manuscript: all figures involving transfections (including Figures 3L, 3M, 5K, etc.) now explicitly indicate the "empty vector" control, and we have added detailed explanations in the figure legends and methods section regarding its role as an internal transfection control and procedural comparator. Once again, we thank the reviewer for their high level of professionalism in helping us enhance the completeness and scientific rigor of our data presentation.

(4) Why HDAC1 with deleted NES does not accumulate or looks like it is degraded? Why then ICP4 does not accumulate?

We sincerely apologize for the lack of clear labeling of the FLAG-HDAC1 ΔNES protein band in Author response image 2. This omission may have led reviewers to misinterpret its expression level as abnormal. After re-evaluation and improved annotation, Author response image 2 now clearly indicates the FLAG-HDAC1 ΔNES band its migration position corresponds to the expected molecular weight (slightly smaller than wild-type FLAG-HDAC1), and the band intensity is comparable to that of the empty vector and wild-type groups, indicating stable intracellular expression of this mutant protein without significant degradation. Therefore, its inhibitory effect on HSV-1 replication is not due to protein instability, but rather results from subcellular localization defects caused by the loss of nuclear export signal (NES): the ΔNES mutation causes HDAC1 to abnormally retain within the nucleus, ultimately leading to significant downregulation of ICP4 transcription and impaired protein accumulation.

**Author response image 2.**

**Reviewer #2 (Public review):**

Summary:

The authors discovered that HDAC1/2 are degraded in HSV-1 and PRV infections. They attempted to establish a new mechanism by which HDAC1/2 are translocated to the cytoplasm to be degraded in HSV-1 infection, and the degradation causes changes in histone acetylation to affect the DDR pathway.

Strengths:

(1) Interesting findings of HDAC1/2 degradation during HSV-1 and PRV infection, and it may impact more than the virology field.

(2) Significant work to identify the ubiquitin site in HDAC1/2 and K63 linkage.

Comments on revised version:

The authors added experiments to address the previous comments. The added knockdown and overexpression experiments provided sufficient support for the proposed mechanism. The conclusions are now strengthened. However, a few essential controls are still missing.

(1) Figure 3K: How does the expression level of Flag-HDAC1 variants compare to the endogenous HDAC1 level? The stripe probed by Flag antibody should be reprobed by HDAC1 antibody. Also, how does the K74R mutant affect histone acetylation? Moreover, the numbers between the panels are hard to read and have not been explained.

We sincerely thank the reviewers for their insightful and constructive feedback. In response to the comment on Figure 3K, we performed antibody re-probing of the Flag-immunoprecipitated or Flag-immunoblotted membranes with a validated HDAC1-specific antibody. Consistent with robust transfection and expression, both wild-type Flag-HDAC1 and its mutants including K74R exhibited markedly elevated total HDAC1 protein levels relative to vector control, confirming efficient exogenous expression and protein stability. To directly assess functional consequences, we evaluated global histone acetylation status in parallel samples and found that the K74R mutant induces significantly greater deacetylation than wild-type Flag-HDAC1, as demonstrated by pronounced reductions in H3K56ac and H4K8 acetylation levels. Finally, to improve clarity and readability, we have revised the lane annotations in Figure 3K—increasing font size, enhancing contrast, and ensuring consistent alignment—and fully documented these modifications in the updated figure legend.

(2) Figure 3M and 3L: DNA transfection per se frequently stimulates cell reactions that inhibit HSV-1 replication. Is the HSV-1 only sample transfected by empty vector or untransfected?

We sincerely appreciate the reviewer's insightful identification of this critical oversight. In all relevant experiments, we have strictly included empty vector transfection controls serving as a baseline reference for each transfection group to eliminate potential influences from the transfection procedure itself and the vector background on viral replication, gene expression, and signaling pathways. The failure to clearly label this control in previous figure legends and main figures was indeed an omission in our presentation; we have now fully addressed this in the revised manuscript: all figures involving transfections (including Figures 3L, 3M, 5K, etc.) now explicitly indicate the "empty vector" control, and we have added detailed explanations in the figure legends and methods section regarding its role as an internal transfection control and procedural comparator. Once again, we thank the reviewer for their high level of professionalism in helping us enhance the completeness and scientific rigor of our data presentation.

(3) Figure 4G-4J: What is the MDM2 knockdown efficiency?

During the construction of the MDM2 knockdown cell lines, we first systematically validated the knockdown efficiency by qRT-PCR. As shown in Figure 4A, compared to the control group (shCtrl), MDM2 mRNA levels were reduced by approximately 60% in shMDM2 cells, and protein expression also showed a corresponding significant decrease, confirming that the cell line had been successfully established and exhibited stable gene silencing effects.

(4) Figure 5F and line 400-401: "thereby preventing HDAC1 degradation-markedly impaired HSV-1 replication (Fig. 5F)." However, viral replication is not demonstrated in Figure 5F.

We sincerely appreciate the reviewer for pointing out the error in the figure legend numbering. Upon verification, the experimental data referred to in lines 400–401 of the original text and in Figure 5F actually correspond to the revised new Figure 5J. We apologize for failing to update the figure references in the main text during the revision process due to an oversight. We have now uniformly corrected all relevant descriptions in the text to "Figure 5J" and conducted a comprehensive review of all figure numbers, table numbers, and cross-references throughout the manuscript to confirm there are no other similar errors.

(5) Figure 5K: also need a control of empty vector. Furthermore, how does the HDAC1 ΔNES expression affect histone acetylation and DDR responses?

We sincerely thank the reviewers for their thoughtful and constructive feedback on Figure 5K. With regard to the empty vector control: all pertinent experiments in this study were performed with rigorous inclusion of an appropriate empty vector control (pCMV-Flag or its isogenic backbone), serving as the definitive negative control. The prior absence of this control in the figure representation was unintentional and reflects an oversight in data presentation—not in experimental design—and we offer our sincere apologies. We have now incorporated the empty vector control bands into Figure 5K and revised the figure legend to explicitly identify and describe this control. In addition, per the reviewers’ recommendation, we conducted a comprehensive assessment of HDAC1 ΔNES function, specifically examining its impact on global histone acetylation and canonical DNA damage response (DDR) activation. Quantitative immunoblotting and immunofluorescence analyses revealed that HDAC1 ΔNES expression leads to significantly greater reduction in H3K56ac and H4K8 acetylation compared with wild-type HDAC1. Moreover, upon induction of DNA damage, HDAC1 ΔNES-expressing cells exhibit attenuated DDR signaling, evidenced by diminished γH2AX focus formation, reduced CHK2 phosphorylation (p-CHK2), and blunted p53 stabilization and activation consistent with impaired DDR initiation or propagation (see Author response image 3). Collectively, these data indicate that nuclear retention of HDAC1 due to NES deletion not only potentiates its chromatin-targeted deacetylase activity but also contributes to suppression of DDR signaling, likely through epigenetic modulation of damage-sensing chromatin domains.

**Author response image 3.**

(6) Statements listed below are better moved to discussion after all data being presented. They are quite a stretch when looking at each figure by itself.

(i) Line 268-270: "Together, these findings indicate that HSV-1 selectively degrades class I HDACs, resulting in widespread histone hyperacetylation that fosters a chromatin state conducive to viral replication". ----may be okay for a statement.

(ii) Line 291-292: "providing initial evidence that HSV-1 infection promotes DDR activation through downregulation of HDAC1 expression"

(iii) Line 331-333: "Together, these results indicate that HSV-1 infection promotes K63-linked polyubiquitination of HDAC1/2 at conserved lysine residues, ultimately leading to their proteasomal degradation."

(iv) Line 334-336 is a repeated sentence.

We sincerely thank the reviewers for their thoughtful and constructive feedback. As noted, statements of mechanistic interpretation are not appropriate in the Results section; accordingly, we have relocated all such statements to the Discussion section. Furthermore, we have conducted a comprehensive line-by-line review of the manuscript to ensure that (i) every mechanistic inference is directly supported by experimental data presented in the Results, and (ii) integrative interpretations particularly those linking molecular observations to broader biological implications are confined exclusively to the Discussion.
